# Synthesis, Inhibitory Activity, and *In Silico* Modeling of Selective COX-1 Inhibitors with a Quinazoline Core

**DOI:** 10.1021/acsmedchemlett.1c00004

**Published:** 2021-03-12

**Authors:** Marcela Dvorakova, Lenka Langhansova, Veronika Temml, Antonio Pavicic, Tomas Vanek, Premysl Landa

**Affiliations:** † Laboratory of Plant Biotechnologies, Czech Academy of Sciences, Institute of Experimental Botany, Rozvojova 263, 165 02 Prague 6 - Lysolaje, Czech Republic; ‡ Department of Pharmaceutical and Medicinal Chemistry, Paracelsus Medical University of Salzburg, Strubergasse 21, 5020 Salzburg, Austria

**Keywords:** Cyclooxygenase, inhibitor, quinazoline, selectivity, docking

## Abstract

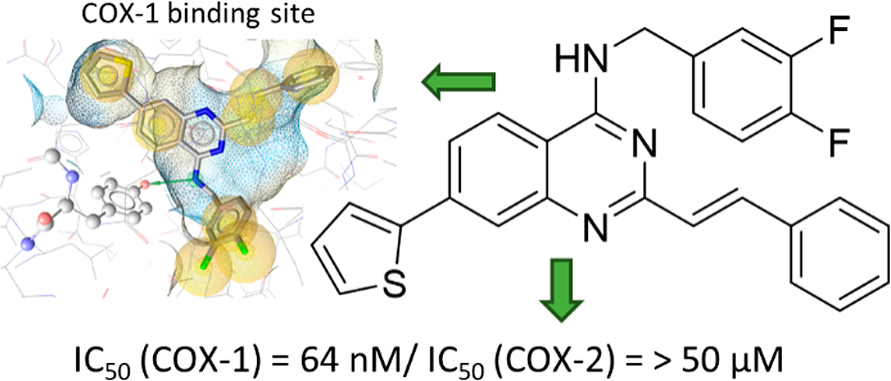

Selective cyclooxygenase-1 (COX-1) inhibition has got into the spotlight with the discovery of COX-1 upregulation in various cancers and the cardioprotective role of COX-1 in control of thrombocyte aggregation. Yet, COX-1-selective inhibitors are poorly explored. Thus, three series of quinazoline derivatives were prepared and tested for their potential inhibitory activity toward COX-1 and COX-2. Of the prepared compounds, 11 exhibited interesting COX-1 selectivity, with 8 compounds being totally COX-1-selective. The IC_50_ value of the best quinazoline inhibitor was 64 nM. The structural features ensuring COX-1 selectivity were elucidated using *in silico* modeling.

Cyclooxygenase (COX) is a bifunctional, membrane-bound homodimer with fatty acid oxygenase and peroxidase function. It is a key enzyme of the inflammation process and the main target of nonsteroidal anti-inflammatory drugs (NSAIDs). Two catalytically functional COX isoforms are present in mammals, COX-1 and COX-2. These isoforms share a considerable protein sequence, and their catalytic mechanism is very similar. Both are activated by peroxides; however, COX-1 requires much higher concentrations for activation than COX-2.^1^ Both also consist of three domains, one of which is the catalytic domain that contains both the fatty acid oxygenase and peroxidase active sites. The oxygenase site is buried deep in the catalytic domain, and its entrance is governed by Arg120, Tyr355, and Glu524 amino acids. The volume of the COX-1 oxygenase site is about 20% smaller than that of COX-2. In addition, the COX-2 oxygenase site contains an additional hydrophilic side-pocket in the proximity of Phe518. In COX-1, an access to this pocket is hindered by bulky amino acids, Ile523 and Ile434, which in COX-2 are replaced by smaller valines.^2^

The oxygenase site catalyzes the synthesis of prostaglandins from arachidonic acid (AA). COX-1 is a primary contributor to the synthesis of thromboxane A2 (TXA2), while COX-2 furnishes predominantly prostaglandin E2 (PGE2) and prostacyclin (PGI2).^3^ Thus, deletion of the COX-1 gene causes predominantly impaired platelet aggregation, whereas COX-2 gene deletion seems to be detrimental to an organism.^4^ Moreover, because COX-2 cannot be usually detected under normal conditions and is rapidly induced during inflammation, it was long believed that COX-2 is the main proinflammatory isoform and COX-1 provides gastrointestinal tract protection and modulates platelet function.^4,5^ This oversimplified view has been lately challenged by a number of studies.^6−9^ Today, it is believed that COX-1 is responsible for the primary prostanoid response to inflammatory stimuli, especially in these cells and tissues, where it is constitutively expressed, whereas COX-2 contributes to prostanoid synthesis later in the inflammation process.^4−6,10−12^ Moreover, COX-1 upregulation was found in various cancers, such as skin, breast, colorectal, or epithelial ovarian cancer^10,11^ as well as during atherosclerosis,^13^ tocolysis,^14^ or neuroinflammation.^12,15,16^ Consequently, selective COX-1 inhibition is a promising target for the treatment of cancers and neurodegenerative disorders. Furthermore, the cardioprotective role of COX-1 in control of thrombocyte aggregation has been reported.^17,18^ On the top of that, COX-1 has been identified as one of two main enzymes involved in the synthesis of chemoprotective factors derived from mesenchymal stem cells, which are able to modulate the response to chemotherapy, suggesting that the inhibition of COX-1 may reverse drug resistance.^19^ Therefore, selective COX-1 inhibitors may provide a new direction in the development of anti-inflammatory compounds with potential activity toward diseases other than those treated with NSAIDs.

Relatively few selective COX-1 inhibitors have been developed so far, such as SC-560, mofezolac, or FR122047 (Figure 1). From these, only mofezolac is used clinically as an analgesic drug and only in Japan.^4,5^ Other compounds, often abundantly used as reference compounds in *in vitro* and *in vivo* studies, have poor pharmacodynamic or pharmacokinetic profiles to enter clinical trials.^4,5^ Cingolani et al. studied the structural basis for COX-1 selectivity of two inhibitors, P6 and mofezolac (Figure 1).^2^ They concluded that the COX-1 selectivity is due to their tighter fit within the COX-1 binding site caused by the nature of substituents on heterocycle core, while carboxylic acid and chlorine in mofezolac and P6, respectively, made inevitable contact with Arg120 and Tyr355 at the entrance to the catalytic domain.

**Figure 1:**
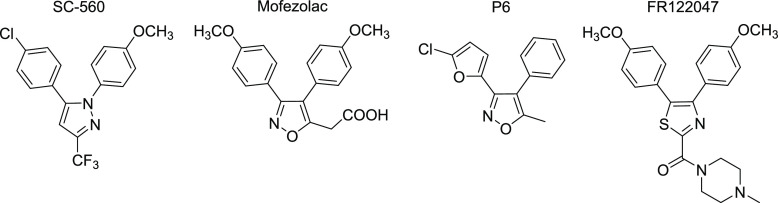
Examples of selective COX-1 inhibitors.

Quinazolines are heterocyclic compounds with numerous therapeutic applications such as anti-inflammatory, analgesic, anticonvulsant, or anticancer applications. There are a few quinazoline compounds on the market used as anti-inflammatory drugs, for example, tryptanthrin, proquazone, or fluproquazone (Figure 2). Proquazone and fluproquazone were developed as third-generation NSAIDs with outstanding safety and efficacy.^20^ Still, the potential of other quinazolines to inhibit COXs is weakly explored. Only several quinazolinones were identified as selective COX-2 inhibitors.^21−26^

**Figure 2:**
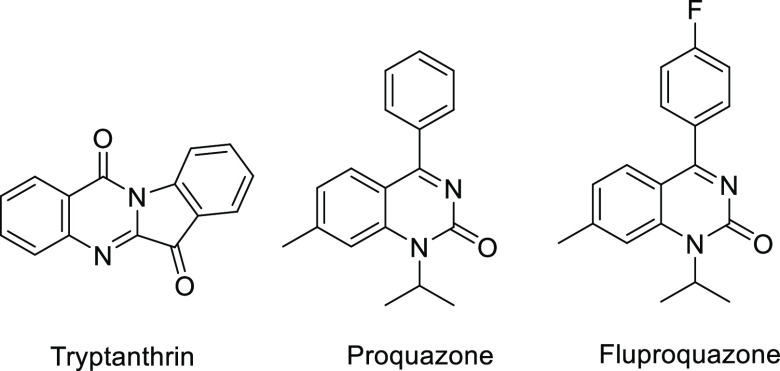
Quinazoline anti-inflammatory drugs on market.

Therefore, three series of 2,4,7-substituted quinazolines as potential COX-1 inhibitors were designed and synthesized. The derivatization of the quinazoline core in positions 2, 4, and 7 was chosen in order to resemble the shape of a letter “V”, which is typical for numerous diarylheterocyclic COX-1 inhibitors. Although several of the selective COX-2 inhibitors (coxibs) also possess the diarylheterocyclic moiety, they always contain either a sulfonamide or methylsulfamoyl group. Instead, we decided to bet on the substitution with a styryl group to partly resemble the structure of stilbenes, a class of selective COX-1 inhibitors.^27^ Other substituents were chosen with the aim to offer distinct electronic and steric properties of the final compounds as well as potential variability in H-bonding with the amino acids within the COX-1 binding site. The synthesized compounds were evaluated *in vitro* for their ability to inhibit COX-1 and COX-2 isoenzymes. The results were also supported by *in silico* modeling.

The synthesis of the first series started with the preparation of (*E*)-4-chloro-2-styrylquinazoline (**2a**) from the commercially available anthranilic acid (**1a**), following a literature procedure.^28^ Shortly, the treatment of **1a** with acetic anhydride, followed by the reaction with benzaldehyde in acetic acid gave an intermediate, which upon exposure to phosphoryl chloride (POCl_3_) afforded (*E*)-4-chloro-2-styrylquinazoline (**2a**) (Scheme 1). Final compounds (**3a**–**v**) were prepared *via* amination of quinazoline **2a** with variously substituted anilines or amines (Table 1).

**Scheme 1:**
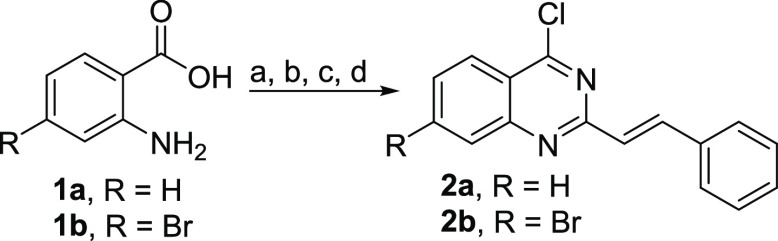
Synthesis of Quinazolines **2a** and **2b** Reagents and conditions: (a) acetic anhydride, 120 °C, 3 h; (b) aqueous ammonia, reflux, 3 h; (c) benzaldehyde, acetic acid, reflux, 12 h; (d) POCl_3_, 4-(dimethylamino)pyridine (DMAP), toluene, reflux, 5 h.

**Table 1:**
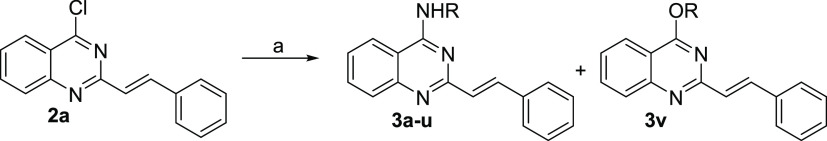
First Series of Quinazoline Derivatives^a^

entry	reagent: RNH_2_	product (yield %)	entry	reagent: RNH_2_	product (yield %)
1	3,4-dimethylaniline	**3a** (73.5)	12	methyl 3-aminopropanoate	**3l** (78.0)
2	*p*-toluidine	**3b** (53.0)	13	4-*tert*-butylaniline	**3m** (62.0)
3	4-methoxyaniline	**3c** (53.8)	14	4-butylaniline	**3n** (72.3)
4	piperazine	**3d** (82.1)	15	4-propoxyaniline	**3o** (63.8)
5	ethyl 4-aminobenzoate	**3e** (60.4)	16	2,4,6-trimethylaniline	**3p** (79.7)
6	3,4-difluoroaniline	**3f** (86.3)	17	2,4,6-trifluoroaniline	**3q** (75.1)
7	4-nitroaniline	**3g** (28.5)	18	2-bromo-4-fluoro-6-methylaniline	**3r** (67.4)
8	8-aminoquinoline	**3h** (84.9)	19	3,5-dimethoxyaniline	**3s** (68.2)
9	2-aminopyridine	**3i** (88.8)	20	3,5-bis(trifluoromethyl)aniline	**3t** (37.7)
10	3-amino-5-methylpyrazole	**3j** (44.0)	21	4-(trifluoromethyl)aniline	**3u** (54.1)
11	3,4-difluorobenzylamine	**3k** (61.6)	22	4-aminophenol	**3v** (70.7)

a Reagents and conditions: (a) corresponding amine or aniline, DMAP, triethylamine, THF, or toluene, reflux, 20 h.

The synthesis of the second series began with the preparation of 7-bromo-2,4-dichloroquinazoline (**4**) from the commercially available 2-amino-4-bromobenzoic acid (**1b**), by the reaction with urea followed by a treatment with POCl_3_, according to published procedure (Scheme 2).^29^ The step-by-step modification of three functional groups in quinazoline **4** afforded the desired final quinazolines. Shortly, the 4-chloro position was aminated using two different benzylamines to give the intermediates **5a**,**b** (Scheme 2), which underwent a Suzuki cross-coupling reaction^30^ of the 7-bromo position with three aromatic boronic acids to afford intermediates **6a**–**e**. Finally, the 2-chloro position was aminated using four different secondary amines to give the final quinazoline derivatives **7a**–**o** (Table 2).

**Scheme 2:**
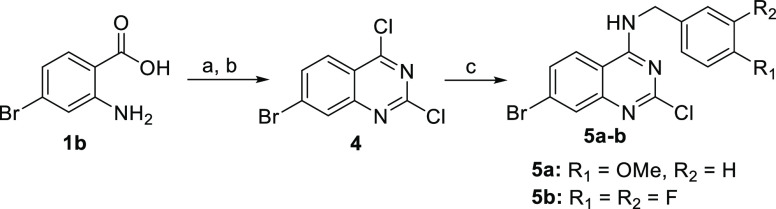
Synthesis of Quinazoline Intermediates **5a**,**b***via* Quinazoline **4** Reagents and conditions: (a) urea, 200 °C, 3 h; (b) POCl_3_, *N*,*N*-dimethylaniline, 120 °C, 4 h; (c) 4-methoxybenzylamine or 3,4-difluorobenzylamine, sodium acetate, THF, 65 °C, 3 h.

**Table 2:**
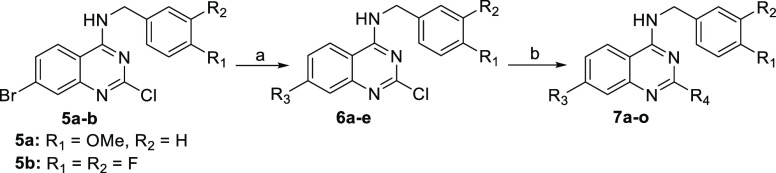
Second Series of Quinazoline Derivatives^a^

entry	reactant	R_3_ (intermediate)	R_4_	product (yield %)
1	**5a**	4-methoxyphenyl (**6a**)	morpholin-1-yl	**7a** (89.8)
2	**5a**	4-methoxyphenyl (**6a**)	pyrrolidin-1-yl	**7b** (80.2)
3	**5a**	4-methoxyphenyl (**6a**)	piperazin-1-yl	**7c** (79.6)
4	**5a**	4-methoxyphenyl (**6a**)	diethylamino	**7d** (40.7)
5	**5a**	4-fluorophenyl (**6b**)	morpholin-1-yl	**7e** (94.9)
6	**5a**	4-fluorophenyl (**6b**)	pyrrolidin-1-yl	**7f** (96.6)
7	**5a**	4-fluorophenyl (**6b**)	piperazin-1-yl	**7g** (90.8)
8	**5a**	4-fluorophenyl (**6b**)	diethylamino	**7h** (34.7)
9	**5a**	thiophen-2-yl (**6c**)	morpholin-1-yl	**7i** (82.6)
10	**5a**	thiophen-2-yl (**6c**)	pyrrolidin-1-yl	**7j** (87.6)
11	**5b**	4-methoxyphenyl (**6d**)	morpholin-1-yl	**7k** (90.1)
12	**5b**	4-methoxyphenyl (**6d**)	pyrrolidin-1-yl	**7l** (91.8)
13	**5b**	4-methoxyphenyl (**6d**)	piperazin-1-yl	**7m** (84.9)
14	**5b**	4-methoxyphenyl (**6d**)	diethylamino	**7n** (54.4)
15	**5b**	thiophen-2-yl (**6e**)	morpholin-1-yl	**7o** (90.5)

a Reagents and conditions: (a) corresponding boronic acid or ester, K_2_CO_3_, PdCl_2_(PPh_3_)_2_, toluene/dioxane/water (10:5:8, v/v/v), 90 °C, overnight; (b) corresponding secondary amine, KI, K_2_CO_3_, *N*,*N*-diisopropylethylamine, dimethylformamide, 110 °C, overnight.

Out of the synthesized compounds, seven exerted potent activity toward COX-1. For these, IC_50_ values were determined on both COX isoforms, while compounds exhibiting less than 50% inhibition on both COX isoforms were considered inactive (for percent inhibition, see Table 1S in the Supporting Information B). From the first series, three compounds (**3b**, **3c**, **3k**) exerted single-digit micromolar inhibitory activity toward COX-1, which was comparable with the inhibitory activity of the reference inhibitor ibuprofen, while their inhibitory activity toward COX-2 was rather moderate (Table 4). Their selectivity index was between 5 and 27. On the other hand, one compound from the first series (**3v**) and three compounds from the second series (**6c**, **6e**, and **7o**) were totally COX-1-selective, as they did not inhibit COX-2 even at 50 μM. It should be noted that two of these COX-1-selective compounds, **6c** and **6e**, were just intermediates, and only one compound, **7o**, belonged to the final derivatives. In addition, these two intermediates exhibited IC_50_ values in the nanomolar range (1 order of magnitude better than ibuprofen), while the IC_50_ value of the final compound **7o** was in micromolar range, and two other corresponding final derivatives, **7i** and **7j**, were found inactive. From these results, it is obvious that the presence of an amine moiety in position 2 decreases substantially the inhibitory activity toward COX-1.

Structurally, all active compounds possessed either a chlorine or styryl group in position 2 and in position 4 was a phenyl or benzyl ring substituted in the *para*-position with an electron-donating group (F, CH_3_, NH_2_, OCH_3_). Except for fluorine, which was tolerated also in the *meta*-position, the presence of other substituents, either an electron-donating (EDG) or electron-withdrawing (EWG), in the *meta*- or *ortho*-position caused inactivity in COX assays. Similarly, with aliphatic or heterocyclic substitution in position 4, the inhibitory activity vanished. Thiophene ring in position 7 significantly enhanced COX-1-selective inhibitory activity and was even indispensable for this activity when the styryl group in position 2 was missing.

Based on these results, two other quinazoline derivatives were prepared as examples of a third series. A combination of the substituents from the active compounds of both previous series was employed. Thus, the styryl group was placed in position 2, a thiophene ring was placed in position 7, and position 4 was substituted with 3,4-difluorobenzylamine or 4-methylaniline (Table 3). The synthesis followed the procedure for the first series,^28^ while the starting material from the second series, 2-amino-4-bromobenzoic acid (**1b**), was used. Thus, (*E*)-7-bromo-4-chloro-2-styrylquinazoline **2b** was formed (Scheme 1). The reaction of **2b** with 3,4-difluorobenzylamine or 4-methylaniline, respectively, afforded intermediates **8a**,**b**, which upon Suzuki cross-coupling^30^ with thiophene-2-boronic acid pinacol ester gave the final quinazolines **9a**,**b** (Table 3).

**Table 3:**
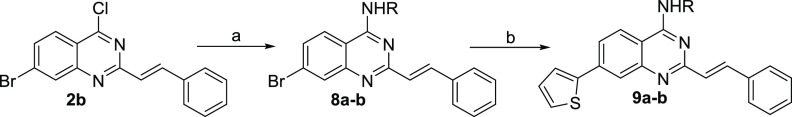
Third Series of Quinazoline Derivatives^a^

entry	reagent 1: RNH_2_	product (yield %)	reagent 2: R_2_B(OH)_2_	product (yield %)
1	4-methylaniline	**8a** (53.4)	thiophene-2-boronic acid pinacol ester	**9a** (77.0)
2	3,4-difluorobenzylamine	**8b** (93.2)		**9b** (85.3)

a Reagents and conditions: (a) Corresponding aniline or benzylamine, DMAP, Et_3_N, toluene, reflux, 3 h; (b) thiophene-2-boronic acid pinacol ester, K_2_CO_3_, PdCl_2_(PPh_3_)_2_, toluene/dioxane/water (10:5:8, v/v/v), 90 °C, overnight.

Both intermediates **8a**,**b** as well as both final compounds **9a**,**b** exerted selective COX-1 inhibitory activity. As expected, the IC_50_ values of intermediates **8a**,**b** were comparable with ibuprofen, as their structure resembled that of the final compounds of the first series, **3b** and **3k** (Table 4). Moreover, the comparison of the IC_50_ values of the final compounds **9a**,**b** (141/64 nM, respectively) with **6c** and **6e** (376 and 142 nM, respectively) demonstrated the importance of the 2-styryl group for the inhibitory activity toward COX-1. Furthermore, similarly to compounds of the second series, the presence of the thiophene ring in compounds **9a**,**b** significantly augmented the COX-1 inhibitory activity, causing a decrease in IC_50_ values by 1 to 2 orders of magnitude, with the best value observed for compound **9b** (IC_50_ = 64 nM). Even though this value was 1 order of magnitude higher than the IC_50_ value determined for the reference COX-1-selective inhibitor, SC-560, such activity together with total COX-1 selectivity renders quinazoline **9b** as a promising compound for further evaluation of its biological activity and pharmacological profile.

**Table 4:** IC_50_ Values for COX-1 and COX-2 Enzymes

compound	IC_50_ COX-1 (μM)	IC_50_ COX-2 (μM)	selectivity index (COX-2/COX-1)
**3b**	1.57 ± 0.54	43.0 ± 10.3	27.4
**3c**	1.89 ± 0.63	37.3 ± 9.36	19.7
**3k**	1.90 ± 0.69	10.1 ± 1.38	5.32
**3v**	3.14 ± 0.99	>50	COX-1-selective
**6c**	0.376 ± 0.189	>50	COX-1-selective
**6e**	0.142 ± 0.014	>50	COX-1-selective
**7o**	1.39 ± 0.72	>50	COX-1-selective
**8a**	0.78 ± 0.64	>50	COX-1-selective
**8b**	1.58 ± 0.89	>50	COX-1-selective
**9a**	0.141 ± 0.045	>50	COX-1-selective
**9b**	0.064 ± 0.044	>50	COX-1-selective
ibuprofen	2.19 ± 0.78	3.30 ± 0.96	1.51
SC-560	0.006 ± 0.003	1.03 ± 0.40	179.5

To determine whether the synthesized compounds compete with the substrate (AA) in binding to the binding site of the COX-1 enzyme, **9b** was selected as a representative compound and tested in COX-1 assays with different concentrations of AA. The ability of compound **9b** to inhibit COX-1 was reduced when the concentration of AA increased in the reaction. IC_50_ values increased from 21.1 nM in the presence of 250 nM AA, through 72.8 nM in the presence of 1250 nM AA, to 254 nM in the presence of 6250 nM AA. It suggests that compound **9b** competed with AA in binding to COX-1. Ibuprofen, which is a known competitive COX-1 inhibitor,^31^ also exhibited such a tendency, as its IC_50_ values increased from 4.00 μM with 250 nM AA to 70.1 μM with 6250 nM AA (Table 2S in the Supporting Information B).

In order to elucidate the structural causes of the selective binding mechanism, all compounds (**3**–**9**) were docked into the binding sites of COX-1 using the pdb entry 3n8w, cocrystallized with flurbiprofen,^32^ and COX-2 using the pdb entry 6COX, cocrystallized with SC-558.^33^ An analysis of the resulting docking poses revealed a hydrogen bridge between the secondary amine functionality and Tyr355, a key residue for electron transfer in the COX mechanism of action, as the primary indicator of activity within the compound class. The most active quinazoline derivative **9b** is placed in the COX-1 binding site so that the amine functionality is located within binding vicinity of Tyr355. The three ring substituents fill different hydrophobic parts of the pocket. The fluorinated benzyl fills a pocket leading up to Met113, and the styryl moiety rests in a pocket leading up to Tyr385. The thiophene moiety is oriented toward Phe518 (Figure 3). In the binding site of COX-2, compound **9b** is flipped and can no longer interact with Tyr355 (Figure 3). The loss of this key interaction could explain the loss of *in vitro* activity.

**Figure 3:**
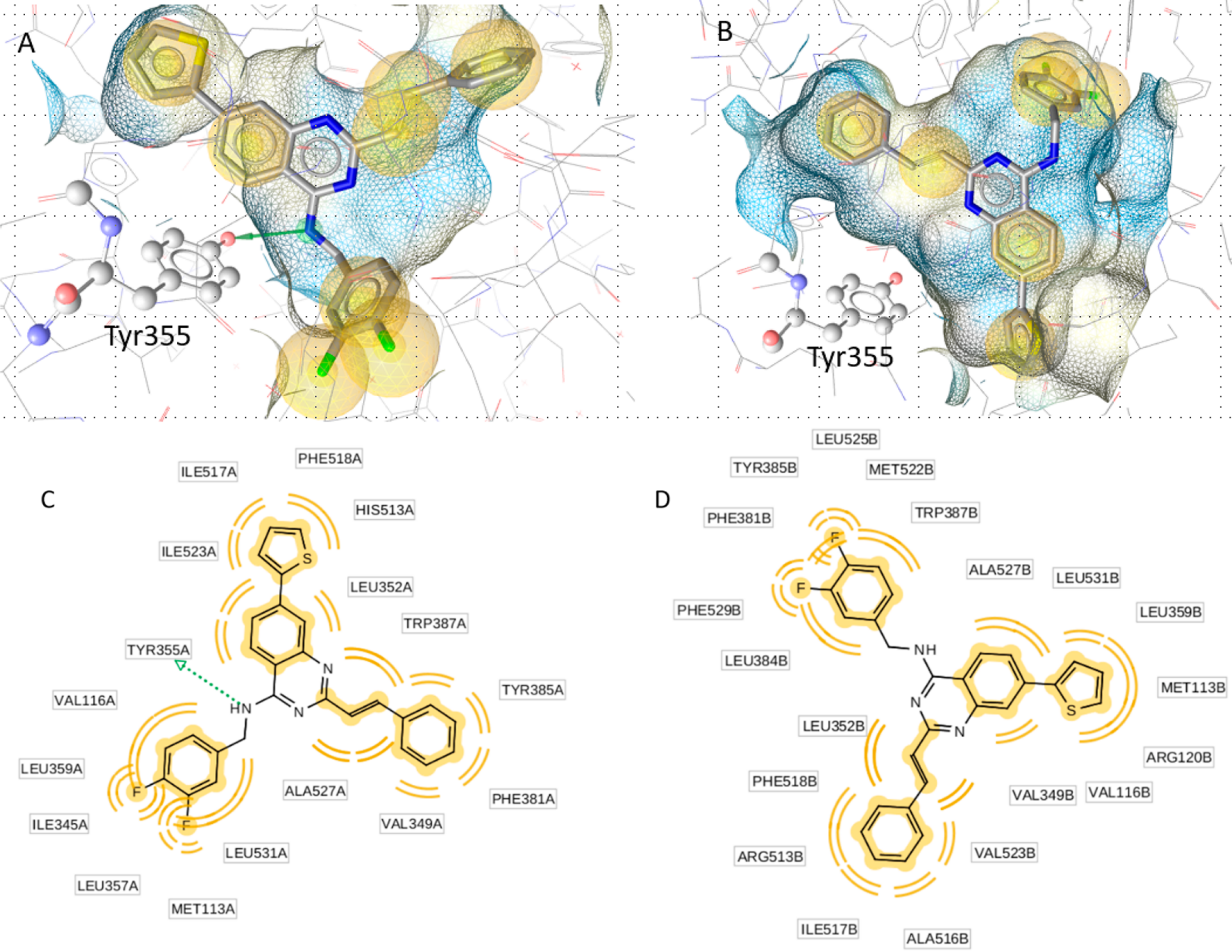
(A,C) **9b** in the binding site of COX-1. The amine functionality acts as a hydrogen bond donor (green arrow) to Tyr355. The yellow spheres represent hydrophobic contacts within the binding pocket. Within COX-1, **9b** assumes an orientation, where the fluorinated benzyl fills a pocket leading up to Met113 and the styryl moiety rests in a pocket leading up to Tyr385. The thiophene moiety adds additional stability by filling a channel leading to Phe518. (B,D) The key hydrogen bond interaction is lost in COX-2, where **9b** assumes a flipped orientation.

A similar effect, but less pronounced, occurs for the smaller molecules from the first series, which still display dual activity. Compound **3k** binds to COX-1 in the same orientation as **9b**, also forming the hydrogen bond with Tyr355, but as it lacks the thiophene ring, the channel leading to Phe518 is empty causing a reduced activity. In COX-2, the molecule is again flipped, but due to the smaller size, the pyrimidine ring can act as an interaction partner for Tyr355 (Figure 4).

**Figure 4:**
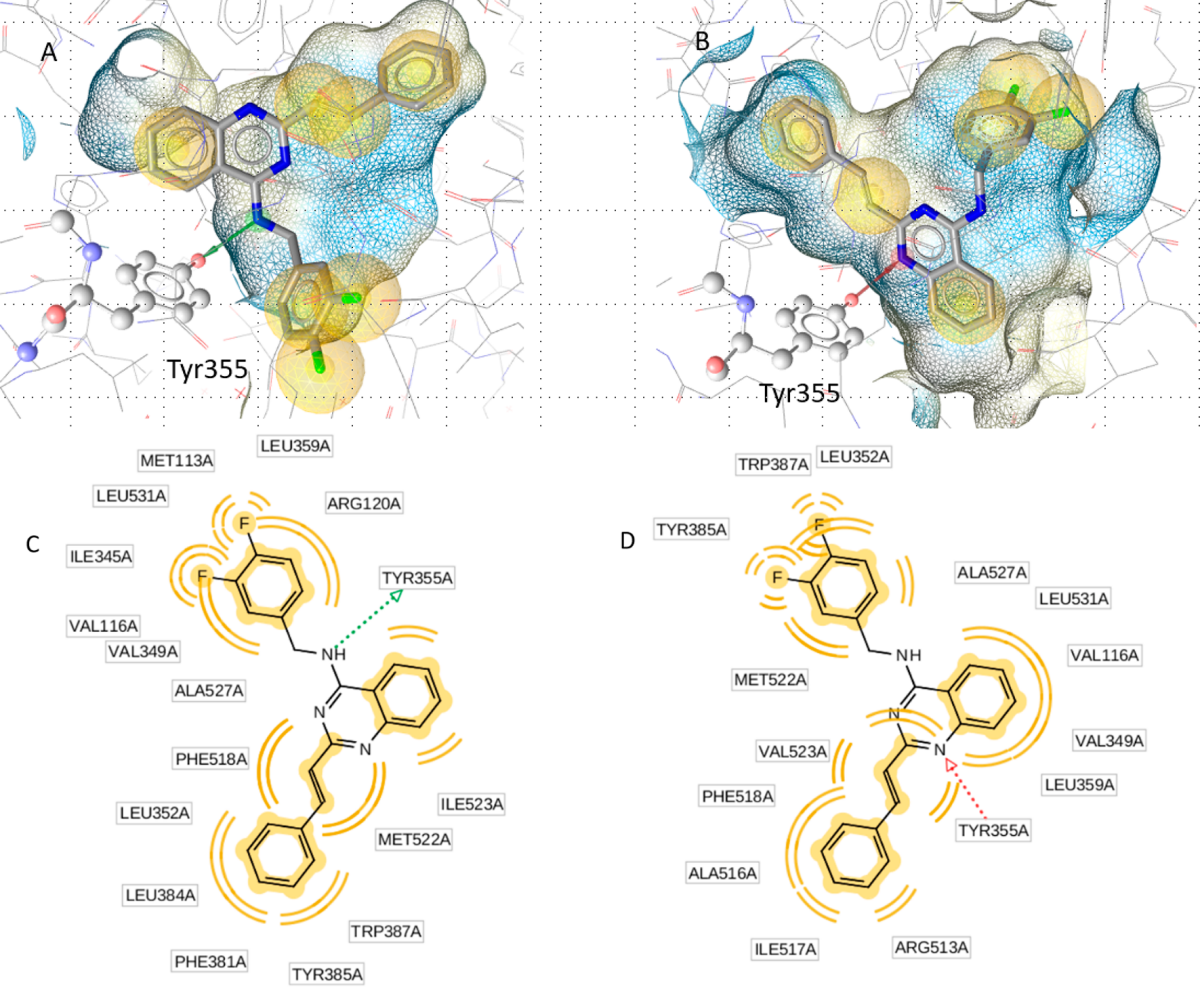
(A,C) **3k** also forms the key hydrogen bond with Tyr355 in COX-1. The fluorinated benzyl group is oriented toward Met113, while the styryl moiety fills the channel leading up to Tyr385. (B,D) In COX-2, the molecule is flipped, but the pyrimidine ring can act as a hydrogen bond acceptor to Tyr355, explaining the residual COX-2 activity in the smaller molecules of the first series. Here, the fluorinated benzyl group fills the channel leading up to Tyr385, while the styryl is oriented toward Phe518. The yellow spheres represent hydrophobic contacts with the binding pocket.

In summary, three series of quinazoline derivatives were synthesized, and their inhibitory activity toward COX-1 and COX-2 isoenzymes was evaluated *in vitro*. From the synthesized compounds, 11 quinazoline derivatives exhibited good to excellent inhibitory activity toward COX-1 (IC_50_ = 0.064–3.14 μM). Out of these, seven compounds did not inhibit a COX-2 isoform even at 50 μM, making these compounds totally COX-1-selective. The IC_50_ values of the most active compounds were 1 to 2 orders of magnitude lower than the inhibitory activity of the reference compound, ibuprofen (IC_50_ = 2.19 μM). According to our results, the compounds compete with the substrate in the binding to COX-1 binding site. All the active compounds possessed in the 4-position of the quinazoline ring either para-EDG-substituted aniline or benzylamine and in the 2-position a chlorine or styryl group. The presence of a thiophene ring in the 7-position markedly enhanced the inhibitory activity as well as COX-1 selectivity. In the docking study, it was shown that the activity hinges on the formation of a hydrogen bond between the secondary amine and key enzyme residue Tyr355. This interaction is only formed in the binding site of COX-1 and thus may be the cause of the selectivity.

## Supporting Information Available

Supporting Information A: Detailed synthetic procedures, complete spectroscopic data of synthesized compounds, experimental procedure of docking experiments and presentation of docking scores, and experimental procedure of the determination of COX inhibition (PDF)
Supporting Information B: ^1^H and ^13^C NMR spectra of all newly synthesized compounds, percentual inhibition of COX-1 and COX-2 isoenzymes by tested compounds at 20 μM, COX-1 inhibition by compound **9b** and ibuprofen at different substrate concentrations, percentual inhibition of COX-1 by active compounds at different concentrations graphically depicted as % inhibition vs log(concentration), UPLC/UV–vis chromatograms of the active compounds showing their purity (PDF)

## Abbreviations

AA: arachidonic acid
COX: cyclooxygenase
DMAP: 4-(dimethylamino)pyridine
EDG: electron-donating group
EWG: electron-withdrawing group
NSAIDs: nonsteroidal anti-inflammatory drugs
PDB: protein data bank
PdCl_2_(PPh_3_)_2_: bis(triphenylphosphine)palladium(II) dichloride
PGE2: prostaglandin E2
PGI2: prostacyclin
POCl_3_: phosphoryl chloride
THF: tetrahydrofuran
TXA2: thromboxane A2
