# Supplementary material for: Synthesis, Inhibitory Activity, and In Silico Modeling of Selective COX-1 Inhibitors with a Quinazoline Core
Source: ACS Med Chem Lett. 2021 Mar 12;12(4):610–6. doi: 10.1021/acsmedchemlett.1c00004 (PMC8040043; doi:10.1021/acsmedchemlett.1c00004)
Supplement: Supplementary file 1 — ml1c00004_si_001.pdf [file ml1c00004_si_001.pdf]

Synthesis, Inhibitory Activity, and *In Silico* Modeling of Selective COX-1 Inhibitors with a Quinazoline Core

Marcela Dvorakova[a,*], Lenka Langhansova[a], Veronika Temml[b], Antonio Pavicic[a], Tomas Vanek[a], Premysl Landa[a]

[a] Laboratory of Plant Biotechnologies, Czech Academy of Sciences, Institute of Experimental Botany, Rozvojova 263, 165 02 Prague 6 - Lysolaje, Czech Republic

[b] Department of Pharmaceutical and Medicinal Chemistry, Paracelsus Medical University of Salzburg, Strubergasse 21, 5020 Salzburg, Austria

Experimental Procedures

Table of Contents

Material and Methods....EP2

Docking Experiments and Scores....EP2

Chemistry....EP3

*Synthesis of (E)-4-chloro-2-styrylquinazoline (**2a**) and (E)-7-bromo-4-chloro-2-styrylquinazoline (**2b**)*....EP3

*General procedure for the first series of quinazoline derivatives (**3a**-**v**)*....EP3

*Synthesis of 7-bromo-2,4-dichloroquinazoline (**4**)*....EP7

*General procedure for quinazoline-4-amines (**5a**-**b**)*....EP8

*General procedure for Suzuki coupling to obtain compounds (**6a-e**)*....EP8

*General procedure for the second series of quinazoline derivatives (**7a-o**)*....EP9

*General procedure for 4-aminoquinazolines (**8a**-**b**)*....EP12

*General procedure for Suzuki coupling to obtain final quinazoline derivatives of the third series (**9a-b**)*....EP13

COX-1 and COX-2 assay....EP13

References....EP14

Material and methods

All reactions requiring anhydrous or inert conditions were carried out under a positive atmosphere of argon in oven-dried glassware. Solutions or liquids were introduced in round bottom flasks using oven-dried syringes through rubber septa. All reactions were stirred magnetically using Teflon-coated stirring bars. If needed, reactions were warmed using an electrically-heated silicon oil bath, and the stated temperature corresponded to the temperature of the bath. Organic solutions obtained after aqueous work-up were dried over $MgSO_4$. The removal of solvents was accomplished using a rotary evaporator at water aspirator pressure. Chemicals for the syntheses were purchased from Sigma-Aldrich (Prague, Czech Republic). Solvents for extractions and chromatography were of technical grade and purchased from Penta Chemicals s.r.o. (Prague, Czech Republic) or from VWR (Stribrna Skalice, Czech Republic). Solvents used in reactions were distilled from appropriate drying agents and stored under argon over activated Linde 4Å molecular sieves. Column and flash chromatography was carried out using Merck silica gel (60-200 µm). Analytical TLC was performed with Merck silica gel 60 F_{254} plates. Visualization was accomplished by UV-light (254 nm) and staining with a vanillin solution, followed by heating. ^{1}H, ^{13}C, and 2D (H-COSY, HMQC) NMR spectra were recorded on a Bruker Avance III™ HD 400 MHz spectrometer equipped with a Prodigy cryo-probe. $CDCl_3$ (7.26 ppm in ^{1}H NMR and 77.0 ppm in ^{13}C NMR), DMSO-d6 (2.50 ppm in ^{1}H NMR and 39.52 ppm in ^{13}C NMR), or methanol-d4 (3.31 ppm in ^{1}H NMR and 49.0 ppm in ^{13}C NMR) were used as internal references. The chemical shifts (δ) are reported in ppm and the coupling constants are recorded in Hz. The hydrogen and carbon assignments were done according to ^{1}H-^{1}H COSY and ^{1}H-^{13}C HMQC experiments. Mass spectra were recorded on an LTQ Orbitrap XL spectrometer. The purity of the products tested for bioactivity was estimated 95 % or higher according to NMR spectra. The purity of the active compounds was also analyzed by UPLC/UV-VIS analysis on Acquity UPLC-MS system equipped with Acquity QDa Mass detector and Acquity UPLC photodiode array detector (from Waters, Prague, Czech Republic). The analysis was done using C18 column (130Å, 1.7 µm, 2.1 mm × 100 mm), C18 VanGuard pre-column (130Å, 1.7 µm, 2.1 mm × 5 mm), with 0.5 mL/min flow, and gradient from 0.1 % HCOOH/H_2O to 0.1 % HCOOH/acetonitrile during 6 min. The purity of the active was 96 % or higher except for compound **6c**, which contained impurity overlapping with blank impurity, thus lowering its estimated purity to 92 %.The chromatograms are provided in Supporting Information B.

Docking experiments

All compounds (**3**-**9**) were docked into the binding sites of COX-1 (using the pdb entry 3n8w, site A, cocrystallized with flurbiprofen)[1] and COX-2 (using the pdb entry 6COX, cocrystallized with SC-558).[2] The docking was performed in GOLD 5.2 running on a Windows 7 machine.[3] The binding sites were defined by the location of the co-crystallized ligands in a radius of 12 Å. All water molecules were deleted from the binding site. Goldscore fitness was selected as a scoring function to evaluate the quality of the individual poses. Both docking workflows were validated by conducting a redocking that resulted in RMSD values to the co-crystallized ligands of the top ranked pose < 1,5 Å. Interaction patterns were analyzed and visualized with LigandScout 4.4. (www.inteligand.com). A set of ten poses per ligand was calculated per structure.

Docking scores table

Compound	IC_{50} COX-1 (µM)	IC_{50} COX-2 (µM)	Selectivity index (COX-2/COX-1)	Goldscore COX-1	Goldscore COX-2
**3b**	1.57±0.54	43.0±10.3	27.4	54.91	66.92
**3c**	1.89±0.63	37.3±9.36	19.7	59.37	64.14

**3k**	1.90±0.69	10.1±1.38	5.32	50.63	60.29
**3v**	3.14±0.99	> 50	COX-1 selective	49.87	64.84
**6c**	0.376±0.189	> 50	COX-1 selective	58.15	51.85
**6e**	0.142±0.014	> 50	COX-1 selective	54.74	54.69
**7o**	1.39±0.72	> 50	COX-1 selective	51.74	56.66
**8a**	0.78±0.64	> 50	COX-1 selective	51.39	66.18
**8b**	1.58±0.89	> 50	COX-1 selective	54.40	61.14
**9a**	0.141±0.045	> 50	COX-1 selective	39.49	62.26
**9b**	0.064±0.044	> 50	COX-1 selective	30.84	61.95
**Ibuprofen**	2.19±0.78	3.30±0.96	1.51	52.47	50.47
**SC-560**	0.006±0.003	1.03±0.40	179.5	54.54	61.94

Chemistry

*Synthesis of (E)-4-chloro-2-styrylquinazoline (**2a**) and (E)-7-bromo-4-chloro-2-styrylquinazoline (**2b**)*

(*E*)-4-Chloro-2-styrylquinazoline **2a** and (*E*)-7-bromo-4-chloro-2-styrylquinazoline **2b**, respectively, were synthesized according to a reported procedure.[4] To anthranilic acid (**1a**, 5.00 g, 36.5 mmol) or 2-amino-4-bromobenzoic acid (**1b**, 7.89g, 36.5 mmol) was added acetic anhydride (Ac_2O, 20 mL, 36.5 mmol) and the resulting mixture was stirred for 3 h at 120°C. Ac_2O was then evaporated and to the residue was added aqueous ammonia (NH_4OH, 28%, 40 mL). The reaction mixture was heated under reflux at 120°C for 3 h, cooled to room temperature (r.t.), filtered through Büchner funnel, washed with water and methanol and the solids were dried on rotary evaporator. To the residue (31.2 mmol) was added acetic acid (AcOH, 30 mL) and benzaldehyde (3.60 mL, 32.5 mmol) and the resulting mixture was stirred at 120°C overnight. Then, it was cooled down to r.t., which led to the precipitation of solids, which were filtered through Büchner funnel, washed with methanol and dried on rotary evaporator. To the residue (18.7 mmol) dissolved in toluene was added $POCl_3$ (3.40 mL, 37.4 mmol) and *N*,*N*-dimethylaniline (DMA, 4.70 mL, 37.4 mmol) and the resulting mixture was stirred at 120°C for 5 h. Then, the solvent was evaporated, the residue was dissolved in chloroform and washed with water. Chloroform was evaporated and the residue was purified by column chromatography in chloroform, which afforded pure products, (*E*)-4-chloro-2-styrylquinazoline **2a**, or (*E*)-7-bromo-4-chloro-2-styrylquinazoline **2b**, respectively.

(*E*)-4-chloro-2-styrylquinazoline (**2a**, orange solid, 88.0 %): ^{1}H NMR ($CDCl_3$): δ 8.22 (1H, ddd, *J* = 0.7, 1.4, 8.3 Hz, Ar); 8.14 (1H, d, *J* = 15.9 Hz, C*H*); 8.00 (1H, ddd, *J* = 0.6, 1.2, 8.4 Hz, Ar); 7.91 (1H, ddd, *J* = 1.4, 7.0, 8.4 Hz, Ar); 7.70 - 7.59 (3H, m, Ar); 7.45 - 7.38 (2H, m, Ar); 7.38 - 7.28 (2H, m, Ar, C*H*). ^{13}C NMR ($CDCl_3$): δ 162.1, 160.3, 151.7, 139.8, 135.8, 134.9, 129.3, 129.0, 128.8, 128.4, 128.1, 127.8, 126.5, 125.9, 122.2. HRMS(ESI) m/z: calcd for $C_{16}H_{12}N_2Cl$ $(M + H)^+$: 267.06835. Found: 267.06825.

(*E*)-7-bromo-4-chloro-2-styrylquinazoline (**2b**, yellow solid, 79.4 %): ^{1}H NMR ($CDCl_3$): δ 8.17 (1H, dd, *J* = 0.5, 1.8 Hz, Ar); 8.14 (1H, d, *J* = 15.9 Hz, C*H*); 8.06 (1H, dd, *J* = 0.5, 8.8 Hz, Ar); 7.71 (1H, dd, *J* = 1.9, 8.8 Hz, Ar); 7.69 - 7.64 (2H, m, Ar); 7.45 - 7.34 (3H, m, Ar); 7.28 (1H, d, *J* = 16.0 Hz, C*H*). ^{13}C NMR ($CDCl_3$): δ 162.0, 161.3, 152.3, 140.6, 135.6, 131.6, 130.8, 129.9, 129.6, 128.9, 127.9, 127.2, 126.2, 120.9. HRMS(ESI) m/z: calcd for $C_{16}H_{11}N_2BrCl$ $(M + H)^+$: 344.97887. Found: 344.97890.

*General procedure for the first series of quinazoline derivatives (**3a**-**v**)*

The synthesis followed a reported procedure.[4] (*E*)-4-Chloro-2-styrylquinazoline **2a** (1×n) was dissolved in THF (or toluene) and corresponding aniline (2×n), triethylamine (2×n) and dimethyl aminopyridine (DMAP, 0.2×n) were added. The reaction mixture was stirred at 80°C (or 120°C in case of toluene) for 20 h. Then, it was evaporated, the residue dissolved in chloroform and washed with water. The chloroform was then evaporated and the residue was purified by column chromatography to give the final quinazolines of the first series, **3a**-**v**. Es an eluent a mixture of hexane/ethyl acetate between 3/1 and 1/1 (v/v) was used.

(*E*)-*N*-(3,4-dimethylphenyl)-2-styryl-4-aminoquinazoline (**3a**, yellowish solid, 73.5 %): ^{1}H NMR (Methanol-d4) δ 8.27 (1H, dt, *J* = 1.0, 8.3 Hz, Ar), 7.96 (1H, d, *J* = 15.8 Hz, C*H*), 7.81 (1H, d, *J* = 2.3 Hz, Ar), 7.76 – 7.67 (2H, m, Ar), 7.61 – 7.54 (3H, m, Ar), 7.50 (1H, ddd, *J* = 3.1, 5.1, 8.3 Hz, Ar), 7.42 – 7.34 (2H, m, Ar), 7.34 – 7.27 (1H, m, Ar), 7.18 (1H, d, *J* = 8.2 Hz, Ar), 7.13 (1H, d, *J* = 15.7 Hz, C*H*), 2.36 (3H, s, C*H₃*), 2.30 (3H, s, C*H₃*). ^{13}C NMR (Methanol-d4): *δ* 161.5, 158.6, 150.8, 139.4, 137.6, 137.3, 137.1, 133.8, 133.1, 130.3, 129.7, 129.5, 128.2, 128.1, 127.5, 126.8, 124.4, 123.0, 120.5, 115.3, 20.3, 19.4. HRMS(ESI) m/z: calcd for $C_{24}H_{22}N_3$ (M + H)$^+$: 352.18082. Found: 352.18090.

(*E*)-2-styryl-*N*-(p-tolyl)-4-aminoquinazoline (**3b**, yellowish solid, 53.0 %): ^{1}H NMR ($CDCl_3$): *δ* 8.00 (1H, d, *J* = 15.8 Hz, C*H*); 7.91 - 7.88 (1H, m, Ar); 7.85 - 7.82 (1H, m, Ar); 7.80 - 7.73 (3H, m, Ar); 7.65 - 7.61 (2H, m, Ar); 7.49 (1H, ddd, *J* = 1.2, 7.0, 8.2 Hz, Ar); 7.42 - 7.37 (3H, m, Ar, N*H*); 7.35 - 7.30 (1H, m, Ar); 7.29 - 7.23 (3H, m, Ar, C*H*); 2.41 (3H, s, C*H₃*). ^{13}C NMR ($CDCl_3$): *δ* 160.7, 156.8, 150.9, 137.9, 136.5, 135.9, 133.8, 132.9, 129.5, 129.4, 128.8, 128.7, 127.6, 125.9, 121.4, 120.3, 113.9, 21.0. HRMS(EI) m/z: calcd for $C_{23}H_{19}N_3$: 337.1579. Found: 337.1580.

(*E*)-*N*-(4-methoxyphenyl)-2-styryl-4-aminoquinazoline (**3c**, yellow solid, 53.8 %): ^{1}H NMR ($CDCl_3$): *δ* 7.96 (1H, d, *J* = 15.8 Hz, C*H*); 7.89 (1H, dq, *J* = 0.6, 8.4 Hz, Ar); 7.84 - 7.81 (1H, m, Ar); 7.80 - 7.73 (3H, m, Ar); 7.64 - 7.60 (2H, m, Ar); 7.49 (1H, ddd, *J* = 1.2, 7.0, 8.2 Hz, Ar); 7.41 - 7.29 (4H, m, Ar, N*H*); 7.24 (1H, d, *J* = 15.8 Hz, C*H*); 7.03 - 6.98 (2H, m, Ar); 3.88 (3H, s, C*H₃*). ^{13}C NMR ($CDCl_3$): *δ* 160.7, 157.0, 156.5, 150.8, 137.8, 136.5, 132.9, 131.5, 128.7, 127.6, 125.9, 123.3, 120.3, 114.1, 113.9, 55.5. HRMS(EI) m/z: calcd for $C_{23}H_{19}N_3O$: 353.1528. Found: 353.1531.

(*E*)-4-(piperazin-1-yl)-2-styrylquinazoline (**3d**, yellowish solid, 82.1 %): ^{1}H NMR ($CDCl_3$): *δ* 8.01 (1H, d, *J* = 15.8 Hz, C*H*); 7.90 - 7.85 (2H, m, Ar); 7.70 (1H, ddd, *J* = 1.3, 6.9, 8.4 Hz, Ar); 7.67 - 7.62 (2H, m, Ar); 7.42 - 7.36 (3H, m, Ar); 7.34 - 7.29 (1H, m, Ar); 7.29 - 7.23 (1H, m, C*H*); 3.82 - 3.76 (4H, m, 2×C*H₂*); 3.16 - 3.10 (4H, m, 2×C*H₂*); 1.86 (1H, s, NH). ^{13}C NMR ($CDCl_3$): *δ* 164.7, 159.8, 152.6, 137.3, 136.5, 132.4, 128.8, 128.7, 128.6, 128.5, 127.5, 125.0, 124.8, 115.5, 51.1, 46.1. HRMS(CI) m/z: calcd for $C_{20}H_{21}N_4$ (M + H)$^+$: 317.1766. Found: 317.1764.

Ethyl (*E*)-4-((2-styrylquinazolin-4-yl)amino)benzoate (**3e**, yellowish solid, 60.4 %): ^{1}H NMR ($CDCl_3$): *δ* 8.18 - 8.14 (2H, m, Ar); 8.05 - 8.00 (3H, m, Ar, C*H*); 7.95 - 7.86 (2H, m, Ar, N*H*); 7.81 (1H, ddd, *J* = 1.3, 7.0, 8.4 Hz, Ar); 7.68 - 7.62 (3H, m, Ar); 7.53 (1H, ddd, *J* = 1.2, 7.0, 8.2 Hz, Ar); 7.44 - 7.39 (2H, m, Ar); 7.37 - 7.32 (1H, m, Ar); 7.29 (1H, d, *J* = 15.8 Hz, C*H*); 4.41 (2H, q, J = 7.1 Hz, C*H₂*); 1.44 (3H, t, *J* = 7.1 Hz, C*H₃*). ^{13}C NMR ($CDCl_3$): *δ* 166.2, 160.5, 156.4, 151.0, 142.8, 138.2, 136.2, 133.3, 130.8, 129.0, 128.9, 128.8, 128.4, 127.7, 126.4, 125.5, 120.1, 119.4, 113.9, 60.9, 14.4. HRMS(EI) m/z: calcd for $C_{25}H_{21}N_3O_2$: 395.1634. Found: 395.1634.

(*E*)-*N*-(3,4-difluorophenyl)-2-styryl-4-aminoquinazoline (**3f**, yellowish solid, 86.3 %): ^{1}H NMR ($CDCl_3$): *δ* 8.2 (1H, ddd, *J* = 2.6, 7.2, 12.6 Hz, Ar); 7.98 (1H, d, *J* = 15.8 Hz, C*H*); 7.91 (1H, dq, *J* = 0.6, 8.3 Hz, Ar); 7.85 - 7.80 (1H, m, Ar); 7.79 (1H, ddd, *J* = 1.3, 7.0, 8.4 Hz, Ar); 7.66 - 7.61 (2H, m, Ar); 7.51 (1H, ddd, *J* = 1.2, 7.0, 8.2 Hz, Ar); 7.45 (1H, br. s, N*H*); 7.43 - 7.38 (2H, m, Ar); 7.36 - 7.30 (2H, m, Ar); 7.29 - 7.17 (2H, m, Ar, C*H*). ^{13}C NMR ($CDCl_3$): *δ* 160.5, 156.5, 151.3 (d, *J* = 13.0 Hz), 150.9, 148.8 (d, *J* = 13.1 Hz), 148.0 (d, *J* = 12.7 Hz), 145.6 (d, *J* = 12.8 Hz), 138.2, 136.2, 135.1 (dd, *J* = 3.3, 9.0 Hz), 133.2, 128.9,

128.8, 128.3, 127.7, 126.3, 120.1, 117.1 (dd, J = 4.8, 17.8 Hz), 116.6 (dd, J = 3.4, 5.8 Hz), 113.6, 111.3, 111.1. HRMS(EI) m/z: calcd for $C_{22}H_{15}N_3F_2$: 359.1234. Found: 359.1232.

(*E*)-*N*-(4-nitrophenyl)-2-styryl-4-aminoquinazoline (**3g**, yellow solid, 28.5 %): ^{1}H NMR ($CDCl_3$): *δ* 8.39 - 8.34 (2H, m, Ar); 8.16 - 8.11 (2H, m, Ar); 8.01 (1H, d, *J* = 15.8 Hz, C*H*); 7.98 - 7.89 (2H, m, Ar); 7.85 (1H, ddd, *J* = 1.3, 6.9, 8.4 Hz, Ar); 7.74 (1H, br. s, N*H*); 7.69 - 7.65 (2H, m, Ar); 7.58 (1H, ddd, *J* = 1.2, 7.0, 8.2 Hz, Ar); 7.46 - 7.41 (2H, m, Ar); 7.39 - 7.34 (1H, m, Ar); 7.31 (1H, d, *J* = 15.8 Hz, C*H*). ^{13}C NMR ($CDCl_3$): *δ* 160.3, 156.2, 151.2, 144.7, 143.0, 138.5, 136.0, 133.6, 129.2, 129.1, 128.9, 128.1, 127.7, 126.7, 125.1, 120.0, 119.9, 113.8. HRMS(ESI) m/z: calcd for $C_{22}H_{17}N_4O_2$ $(M + H)^+$: 369.13460. Found: 369.13424.

(*E*)-*N*-(quinolin-8-yl)-2-styryl-4-aminoquinazoline (**3h**, yellow solid, 84.9 %): ^{1}H NMR ($CDCl_3$): *δ* 10.8 (1H, s, N*H*); 9.45 (1H, dd, *J* = 1.2, 7.8 Hz, Ar); 8.92 (1H, dd, *J* = 1.6, 4.2 Hz, Ar); 8.25 - 8.20 (2H, m, Ar); 8.13 (1H, d, *J* = 15.8 Hz, C*H*); 7.92 (1H, dq, *J* = 0.6, 8.4 Hz, Ar); 7.81 (1H, ddd, *J* = 1.3, 6.9, 8.4 Hz, Ar); 7.74 - 7.69 (3H, m, Ar); 7.60 (1H, ddd, *J* = 1.3, 6.9, 8.2 Hz, Ar); 7.56 - 7.49 (2H, m, Ar); 7.46 - 7.41 (2H, m, Ar); 7.40 - 7.33 (2H, m, Ar, C*H*). ^{13}C NMR ($CDCl_3$): *δ* 160.8, 156.6, 150.7, 148.1, 139.2, 137.6, 136.5, 135.1, 132.9, 129.1, 128.8, 128.7, 128.1, 127.7, 127.5, 126.2, 121.7, 121.1, 120.6, 116.6, 115.1. HRMS(ESI) m/z: calcd for $C_{25}H_{19}N_4$ $(M + H)^+$: 375.16042. Found: 375.16005.

(*E*)-*N*-(pyridin-2-yl)-2-styryl-4-aminoquinazoline (**3i**, yellow solid, 88.8 %): ^{1}H NMR ($CDCl_3$): *δ* 8.89 (1H, dt, *J* = 1.0, 8.4 Hz, Ar); 8.37 (1H, dq, *J* = 1.0, 5.0 Hz, Ar); 8.34 (1H, br. s, N*H*); 8.04 (1H, d, *J* = 15.8 Hz, C*H*); 7.97 (1H, d, *J* = 8.2 Hz, Ar); 7.92 (1H, d, *J* = 8.2 Hz, Ar); 7.90 - 7.84 (1H, m, Ar); 7.83 - 7.78 (1H, m, Ar); 7.69 - 7.65 (2H, m, Ar); 7.54 (1H, ddd, *J* = 1.2, 6.9, 8.3 Hz, Ar); 7.45 - 7.39 (2H, m, Ar); 7.38 - 7.29 (2H, m, Ar, C*H*); 7.09 (1H, ddd, *J* = 1.0, 4.8, 7.2 Hz, Ar). ^{13}C NMR ($CDCl_3$): *δ* 160.3, 156.1, 152.1, 150.9, 148.0, 138.1, 137.9, 136.3, 133.3, 128.9, 128.8, 128.7, 127.6, 126.5, 120.6, 119.1, 114.7, 113.2. HRMS(ESI) m/z: calcd for $C_{21}H_{17}N_4$ $(M + H)^+$: 325.14477. Found: 325.14446.

(*E*)-*N*-(5-methyl-1H-pyrazol-3-yl)-2-styryl-4-aminoquinazoline (**3j**, yellowish solid, 44.0 %): ^{1}H NMR ($CDCl_3$): *δ* 9.38 (1H, ddd, *J* = 0.7, 1.5, 8.6 Hz, Ar); 7.97 (1H, dq, *J* = 0.7, 8.4 Hz, Ar); 7.92 (1H, d, *J* = 15.8 Hz, C*H*); 7.86 (1H, ddd, *J* = 1.4, 6.8, 8.4 Hz, Ar); 7.77 - 7.62 (2H, m, Ar); 7.59 (1H, ddd, *J* = 1.3, 6.8, 8.6 Hz, Ar); 7.44 - 7.39 (2H, m, Ar); 7.38 - 7.32 (2H, m, Ar, C*H*); 5.8 (2H, br. s, 2xN*H*); 5.52 (1H, s, C*H*); 2.31 (3H, s, C*H3*). ^{13}C NMR ($CDCl_3$): *δ* 158.6, 157.4, 153.7, 152.6, 150.5, 137.9, 136.0, 134.0, 129.4, 129.2, 128.9, 128.1, 127.7, 127.6, 127.1, 116.3, 90.8, 14.3). HRMS(ESI) m/z: calcd for $C_{20}H_{18}N_5$ $(M + H)^+$: 328.15567. Found: 328.15520.

(*E*)-*N*-(3,4-difluorobenzyl)-2-styryl-4-aminoquinazoline (**3k**, white solid, 61.6 %): ^{1}H NMR ($CDCl_3$): *δ* 7.97 (1H, d, *J* = 15.8 Hz, C*H*); 7.86 (1H, dq, *J* = 0.6, 8.4 Hz, Ar); 7.76 - 7.69 (2H, m, Ar); 7.64 - 7.60 (2H, m, Ar); 7.45 - 7.36 (3H, m, Ar); 7.34 - 7.26 (2H, m, Ar); 7.23 (1H, d, *J* = 15.8 Hz, C*H*); 7.20 - 7.11 (2H, m, Ar); 6.02 (1H, t, *J* = 5.6 Hz, N*H*); 4.94 (2H, d, *J* = 5.6 Hz, C*H2*). ^{13}C NMR ($CDCl_3$): *δ* 160.7, 158.7, 151.6 (d, *J* = 12.8 Hz), 151.0 (d, *J* = 12.6 Hz), 150.4, 149.2 (d, *J* = 12.7 Hz), 148.5 (d, *J* = 12.4 Hz), 137.5, 136.4, 135.8 (dd, *J* = 3.8, 5.1 Hz), 132.9, 128.8, 128.7, 128.7, 128.5, 127.5, 125.6, 123.8 (dd, *J* = 3.6, 6.3 Hz), 120.4, 117.4 (d, *J* = 17.0 Hz), 116.9 (d, *J* = 17.3 Hz), 113.6, 44.3. HRMS(EI) m/z: calcd for $C_{23}H_{17}N_3F_2$: 373.1391. Found: 373.1392.

Methyl (*E*)-3-((2-styrylquinazolin-4-yl)amino)propanoate (**3l**, white solid, 78.0 %): ^{1}H NMR ($CDCl_3$): *δ* 8.00 (1H, d, *J* = 15.8 Hz, C*H*); 7.82 (1H, dq, *J* = 0.6, 8.3 Hz, Ar); 7.72 - 7.63 (4H, m, Ar); 7.43 - 7.36 (3H, m, Ar); 7.34 - 7.29 (1H, m, Ar); 7.22 (1H, d, *J* = 15.8 Hz, C*H*); 6.44 (1H, t, *J* = 6.0 Hz, N*H*); 4.08 (2H, q, *J* = 6.0 Hz, C*H2*); 3.73 (3H, s, C*H3*); 2.84 (2H, t, *J* = 6.0 Hz, C*H2*). ^{13}C NMR ($CDCl_3$): *δ* 173.6, 160.6, 158.8, 150.3, 137.1, 136.6, 132.6, 129.1, 128.7, 128.6, 128.3, 127.5, 125.5, 120.7, 113.9, 51.9, 30.5, 33.3. HRMS(EI) m/z: calcd for $C_{20}H_{19}N_3O_2$: 333.1477. Found: 333.1476.

(*E*)-*N*-(4-(*tert*-butyl)phenyl)-2-styryl-4-aminoquinazoline (**3m**, yellowish solid, 62.0 %): ^{1}H NMR ($CDCl_3$): δ 8.01 (1H, d, *J* = 15.8 Hz, C*H*); 7.92 - 7.85 (2H, m, Ar); 7.85 - 7.80 (2H, m, Ar); 7.76 (1H, ddd, *J* = 1.2, 7.0, 8.3 Hz, Ar); 7.65 - 7.62 (2H, m, Ar); 7.54 (1H, br. s, N*H*); 7.51 - 7.46 (3H, m, Ar); 7.43 - 7.37 (2H, m, Ar); 7.36 - 7.30 (1H, m, Ar); 7.28 (1H, d, *J* = 15.8 Hz, C*H*); 1.39 (9H, s, 3×CH_3). ^{13}C NMR ($CDCl_3$): δ 160.6, 156.8, 150.5, 147.2, 138.1, 136.4, 135.9, 132.9, 128.8, 128.7, 128.5, 127.7, 126.0, 125.8, 120.9, 120.4, 113.9, 34.5, 31.4. HRMS(ESI) m/z: calcd for $C_{26}H_{26}N_3$ (M + H)$^+$: 380.21212. Found: 380.21194.

(*E*)-*N*-(4-butylphenyl)-2-styryl-4-aminoquinazoline (**3n**, yellowish solid, 72.3 %): ^{1}H NMR ($CDCl_3$): δ 8.00 (1H, d, *J* = 15.8 Hz, C*H*); 7.91 - 7.83 (2H, m, Ar); 7.81 - 7.73 (3H, m, Ar); 7.65 - 7.60 (2H, m, Ar); 7.52 - 7.45 (2H, m, Ar); 7.42 - 7.36 (2H, m, Ar); 7.35 - 7.30 (1H, m, Ar); 7.30 - 7.24 (3H, m, Ar, C*H*); 2.67 (2H, t, *J* = 7.7 Hz, CH_2); 1.70 - 1.62 (2H, m, CH_2); 1.46 - 1.36 (2H, m, CH_2); 0.97 (3H, t, *J* = 7.3 Hz, CH_3). ^{13}C NMR ($CDCl_3$): δ 160.7, 156.8, 150.6, 139.0, 138.0, 136.4, 136.1, 132.9, 128.8, 128.75, 128.7, 128.6, 127.6, 126.0, 121.3, 120.4, 113.9, 35.1, 33.7, 22.4, 14.0. HRMS(ESI) m/z: calcd for $C_{26}H_{26}N_3$ (M + H)$^+$: 380.21212. Found: 380.21219.

(*E*)-*N*-(4-propoxyphenyl)-2-styryl-4-aminoquinazoline (**3o**, yellow solid, 63.8 %): ^{1}H NMR ($CDCl_3$): δ 7.96 (1H, d, *J* = 15.8 Hz, C*H*); 7.91 - 7.82 (2H, m, Ar); 7.79 - 7.71 (3H, m, Ar); 7.65 - 7.59 (2H, m, Ar); 7.51 - 7.35 (4H, m, Ar, N*H*); 7.35 - 7.29 (1H, m, Ar); 7.27 - 7.22 (1H, m, C*H*); 7.03 - 6.97 (2H, m, Ar); 3.98 (2H, t, *J* = 6.6 Hz, CH_2); 1.86 (2H, h, *J* = 7.1 Hz, CH_2); 1.08 (3H, t, *J* = 7.4 Hz, CH_3). ^{13}C NMR ($CDCl_3$): δ 160.7, 157.0, 156.1, 150.6, 137.9, 136.4, 132.9, 131.3, 128.71, 128.68, 128.7, 128.63, 128.59, 127.6, 125.9, 123.3, 120.4, 114.7, 113.8, 69.9, 22.6, 10.6. HRMS(ESI) m/z: calcd for $C_{25}H_{24}N_3O$ (M + H)$^+$: 382.19139. Found: 382.19101.

(*E*)-*N*-mesityl-2-styryl-4-aminoquinazoline (**3p**, yellowish solid, 79.7 %): ^{1}H NMR ($CDCl_3$): δ 7.91 (1H, d, *J* = 8.3 Hz, Ar); 7.82 (1H, br. d, *J* = 5.6 Hz, Ar); 7.77 (1H, ddd, *J* = 1.3, 7.0, 8.4 Hz, Ar); 7.71 (1H, d, *J* = 15.8 Hz, C*H*); 7.55 - 7.52 (2H, m, Ar); 7.46 (1H, t, *J* = 7.6 Hz, Ar); 7.37 - 7.31 (2H, m, Ar); 7.30 - 7.26 (1H, m, Ar); 7.15 (1H, d, *J* = 15.8 Hz, C*H*); 7.01 (2H, s, Ar); 6.93 (1H, br. s, N*H*); 2.38 (3H, s, CH_3); 2.24 (6H, s, 2×CH_3). ^{13}C NMR ($CDCl_3$): δ 160.9, 158.1, 138.5, 136.5, 135.5, 132.8, 132.6, 129.0, 128.7, 128.6, 127.6, 125.7, 120.9, 113.6, 21.0, 18.7. HRMS(ESI) m/z: calcd for $C_{25}H_{24}N_3$ (M + H)$^+$: 366.19647. Found: 366.19658.

(*E*)-2-styryl-*N*-(2,4,6-trifluorophenyl)-4-aminoquinazoline (**3q**, yellowish solid, 75.1 %): ^{1}H NMR ($CDCl_3$): δ 7.95 (2H, ddd, *J* = 1.2, 4.0, 8.2 Hz, Ar); 7.80 (1H, ddd, *J* = 1.3, 7.0, 8.4 Hz, Ar); 7.75 (1H, d, *J* = 15.8 Hz, C*H*); 7.55 - 7.49 (3H, m, Ar); 7.40 - 7.33 (2H, m, Ar); 7.33 - 7.28 (1H, m, Ar); 7.19 (1H, d, *J* = 15.8 Hz, C*H*); 7.08 (1H, br. s, N*H*); 6.91 - 6.83 (2H, m, Ar). ^{13}C NMR ($CDCl_3$): δ 161.8 (d, *J* = 14.6 Hz), 160.4, 159.7 (dd, *J* = 15.1, 7.4 Hz), 159.3, 157.4, 157.2 (dd, *J* = 15.1, 7.3 Hz), 150.6 (d, *J* = 1.6 Hz), 138.4, 136.2, 133.3, 128.8, 128.7, 128.4, 127.9, 127.6, 126.2, 121.1, 113.7, 112.0 (td, *J* = 16.2, 5.0 Hz), 100.6 (td, *J* = 26.1, 3.3 Hz). HRMS(ESI) m/z: calcd for $C_{22}H_{15}N_3F_3$ (M + H)$^+$: 378.12126. Found: 378.12131.

(*E*)-*N*-(2-bromo-4-fluoro-6-methylphenyl)-2-styryl-4-aminoquinazoline (**3r**, yellow solid, 67.4 %): ^{1}H NMR ($CDCl_3$): δ 7.96 - 7.90 (2H, m, Ar); 7.81 (1H, ddd, *J* = 1.3, 7.0, 8.4 Hz, Ar); 7.65 (1H, d, *J* = 15.8 Hz, C*H*); 7.57 - 7.48 (3H, m, Ar); 7.39 - 7.27 (4H, m, Ar); 7.15 (1H, d, *J* = 15.8 Hz, C*H*); 7.11 - 7.06 (2H, m, Ar, N*H*); 2.35 (3H, s, CH_3). ^{13}C NMR ($CDCl_3$): δ 161.8, 160.6, 159.4, 157.5, 150.9, 140.6 (d, *J* = 8.6 Hz), 137.9, 136.3, 133.1, 131.8 (d, *J* = 3.3 Hz), 128.7 (d, *J* = 2.9 Hz), 128.6, 128.5, 127.6, 126.1, 122.4 (d, *J* = 10.7 Hz), 120.8, 117.3 (d, *J* = 25.3 Hz), 116.6 (d, *J* = 21.7 Hz), 113.6, 20.0 (d, *J* = 1.3 Hz). HRMS(ESI) m/z: calcd for $C_{23}H_{18}N_3BrF$ (M + H)$^+$: 434.06626. Found: 434.06622.

(*E*)-*N*-(3,5-dimethoxyphenyl)-2-styryl-4-aminoquinazoline (**3s**, yellow solid, 68.2 %): ^{1}H NMR ($CDCl_3$): δ 8.05 (1H, d, *J* = 15.8 Hz, C*H*); 7.88 (2H, ddd, *J* = 1.0, 8.2, 14.9 Hz, Ar); 7.76 (1H, ddd, *J* = 1.3, 7.0, 8.4

Hz, Ar); 7.62 - 7.57 (2H, m, Ar); 7.54 (1H, br. s, N*H*); 7.49 (1H, ddd, *J* = 1.2, 7.0, 8.2 Hz, Ar); 7.42 - 7.36 (2H, m, Ar); 7.35 - 7.31 (1H, m, Ar); 7.28 (1H, d, *J* = 15.9 Hz, C*H*); 7.19 (2H, d, *J* = 2.2 Hz, Ar); 6.32 (1H, t, *J* = 2.2 Hz, Ar); 3.87 (6H, s, 2×CH_3). ^{13}C NMR ($CDCl_3$): *δ* 161.0, 160.5, 156.7, 150.5, 140.3, 137.9, 136.3, 133.0, 128.84, 128.78, 128.63, 128.57, 127.5, 126.1, 120.3, 113.9, 99.3, 96.6, 55.5. HRMS(ESI) m/z: calcd for $C_{24}H_{22}N_3O_2$ (M + H)$^+$: 384.17065. Found: 384.17073.

(*E*)-*N*-(3,5-bis(trifluoromethyl)phenyl)-2-styryl-4-aminoquinazoline (**3t**, yellowish solid, 37.7 %): ^{1}H NMR ($CDCl_3$): *δ* 8.55 (2H, s, Ar); 7.99 (1H, d, *J* = 15.9 Hz, C*H*); 7.92 (2H, dddd, *J* = 0.6, 1.3, 8.2, 12.9 Hz, Ar); 7.82 (1H, ddd, *J* = 1.3, 7.0, 8.3 Hz, Ar); 7.75 (1H, br. s, N*H*); 7.68 (1H, heptuplet, *J* = 0.8 Hz, Ar); 7.65 - 7.61 (2H, m, Ar); 7.56 (1H, ddd, *J* = 1.2, 7.0, 8.2 Hz, Ar); 7.44 - 7.39 (2H, m, Ar); 6.38 - 6.32 (1H, m, Ar); 7.29 (1H, d, *J* = 15.8 Hz, C*H*). ^{13}C NMR ($CDCl_3$): *δ* 160.3, 156.2, 151.0, 140.1, 138.5, 136.0, 133.6, 132.2 (q, *J* = 33.3 Hz), 129.1 (d, *J* = 1.8 Hz), 128.8, 127.9, 127.6, 126.6, 124.7, 122.0, 120.5 (d, *J* = 3.0 Hz), 120.0, 119.3, 116.8 (p, *J* = 4.1 Hz), 113.6. HRMS(ESI) m/z: calcd for $C_{24}H_{16}N_3F_6$ (M + H)$^+$: 460.12429. Found: 460.12423.

(*E*)-2-styryl-*N*-(4-(trifluoromethyl)phenyl)-4-aminoquinazoline (**3u**, yellowish solid, 54.1 %): ^{1}H NMR ($CDCl_3$): *δ* 8.04 (2H, d, *J* = 8.4 Hz, Ar); 7.98 (1H, d, *J* = 15.9 Hz, C*H*); 7.89 (2H, ddd, *J* = 1.2, 8.4, 17.8 Hz, Ar); 7.79 (1H, ddd, *J* = 1.3, 7.0, 8.4 Hz, Ar); 7.70 (2H, d, *J* = 8.5 Hz, Ar); 7.66 - 7.60 (3H, m, Ar, N*H*); 7.51 (1H, ddd, *J* = 1.3, 6.9, 8.2 Hz, Ar); 7.43 - 7.38 (2H, m, Ar); 7.37 - 7.32 (1H, m, Ar); 7.28 (1H, d, *J* = 15.8 Hz, C*H*). ^{13}C NMR ($CDCl_3$): *δ* 160.4, 156.5, 150.9, 141.7, 138.3, 136.1, 133.3, 129.0, 128.9, 128.8, 128.3, 127.7, 126.4, 126.2 (q, *J* = 3.8 Hz), 125.7, 125.5, 125.4, 122.9, 120.5, 113.8. HRMS(ESI) m/z: calcd for $C_{23}H_{17}N_3F_3$ (M + H)$^+$: 392.13691. Found: 392.13704.

(*E*)-4-((2-styrylquinazolin-4-yl)oxy)aniline (**3v**, yellowish solid, 70.7 %): ^{1}H NMR ($CDCl_3$): *δ* 8.32 (1H, ddd, *J* = 0.7, 1.5, 8.2 Hz, Ar); 7.96 - 7.92 (1H, m, Ar); 7.85 (1H, ddd, *J* = 1.5, 6.9, 8.4 Hz, Ar); 7.76 (1H, d, *J* = 15.8 Hz, C*H*); 7.59 - 7.53 (3H, m, Ar); 7.38 - 7.27 (3H, m, Ar); 7.19 (1H, d, *J* = 15.8 Hz, C*H*); 7.17 - 7.12 (2H, m, Ar); 6.83 - 6.77 (2H, m, Ar); 3.73 (2H, s, NH_2). ^{13}C NMR ($CDCl_3$): *δ* 166.5, 160.5, 152.3, 144.7, 143.9, 138.5, 138.5, 136.2, 133.9, 128.7, 127.9, 127.7, 127.5, 126.6, 123.8, 123.7, 122.6, 122.5, 115.7, 115.6, 115.3. HRMS(EI) m/z: calcd for $C_{22}H_{17}N_3O$: 339.1372. Found: 339.1374.

*Synthesis of 7-bromo-2,4-dichloroquinazoline (**4**)*

7-Bromo-2,4-dichloroquinazoline **4** was synthesized according to a reported procedure.[5] To 2-amino-4-bromobenzoic acid (**1b**, 8.00 g, 37.0 mmol) was added urea (22.2 g, 370 mmol) and together were grinded in mortar with pestle. When grinded, the mixture was placed into an oven heated to 200°C where it was left for 3 h. Then, it was let to cool down to 80°C, suspended in water and the mixture was filtered through Büchner funnel and the solids were dried on rotary evaporator. The obtained 7-bromoquinazoline-2,4(1*H*,3*H*)-dione was used directly in the next step without purification. To 7-bromoquinazoline-2,4(1*H*,3*H*)-dione (6.00 g, 24.9 mmol) was added $POCl_3$ (30 mL, 328 mmol) and *N*,*N*-dimethylaniline (DMA, 6.30 mL, 49.8 mmol) and the resulting mixture was stirred at 120°C for 4 h. Then, $POCl_3$ was evaporated, the residue was dissolved in dichloromethane and washed with water and brine. Dichloromethane was evaporated and the residue was purified by column chromatography in hexane/ethyl acetate using gradient from 10/1 (v/v) to 5/1 (v/v), which afforded pure product, 7-bromo-2,4-dichloroquinazoline **4**, as a yellowish solid in 73.7 % yield. ^{1}H NMR ($CDCl_3$): *δ* 8.20 (1H, dd, *J* = 0.6, 1.8 Hz, Ar); 8.12 (1H, dd, *J* = 0.5, 8.8 Hz, Ar); 7.83 (1H, dd, *J* = 1.9, 8.9 Hz, Ar). ^{13}C NMR ($CDCl_3$): *δ* 164.0, 156.2, 152.8, 133.0, 131.6, 130.5, 127.2, 121.1. HRMS(EI) m/z: calcd for $C_8H_3N_2Cl_2Br$: 275.8857. Found: 275.8854.

*General procedure for quinazoline-4-amines (**5a**-**b**)*

The synthesis followed a reported procedure.[5] 7-Bromo-2,4-dichloroquinazoline **4** (1×n) was dissolved in THF and corresponding benzylamine (1.1×n) was added followed by the addition of sodium acetate (NaOAc, 1.1×n). The mixture was stirred under reflux at 65°C overnight. The mixture was then cooled down to r.t., washed with water and water phase was extracted with ethyl acetate. Combined organic layers were dried over $MgSO_4$, filtered and evaporated. The obtained residue was purified by column chromatography to give 4-aminoquinazoline **5a-b**, respectively. Es an eluent a mixture of hexane/ethyl acetate 4/1 (v/v) was used.

7-Bromo-2-chloro-*N*-(4-methoxybenzyl)-4-aminoquinazoline (**5a**, white solid, 87.6%): ^1H NMR ($CDCl_3$): *δ* 7.93 (1H, dd, *J* = 0.6, 1.7 Hz, Ar); 7.55 - 7.47 (2H, m, Ar); 7.33 (2H, ddd, *J* = 2.1, 3.1, 8.8 Hz, Ar); 6.90 (2H, ddd, *J* = 2.1, 3.1, 8.6 Hz, Ar); 6.01 (1H, br. s, N*H*); 4.76 (2H, d, *J* = 5.1 Hz, C*H₂*); 3.81 (3H, s, OC*H₃*). ^{13}C NMR ($CDCl_3$): *δ* 160.3, 159.5, 158.7, 151.8, 130.4, 129.8, 129.6, 128.9, 128.1, 122.1, 114.4, 111.8, 55.3, 45.4. HRMS(ESI) m/z: calcd for $C_{16}H_{14}N_3OBrCl$ $(M + H)^+$: 378.00033. Found: 378.00009.

7-Bromo-2-chloro-*N*-(3,4-difluorobenzyl)-4-aminoquinazoline (**5b**, white solid, 77.5 %): ^1H NMR ($CDCl_3$): *δ* 7.96 (1H, dd, *J* = 0.6, 1.8 Hz, Ar); 7.57 (1H, dd, *J* = 1.8, 8.8 Hz, Ar); 7.53 (1H, dd, *J* = 0.6, 8.8 Hz, Ar); 7.25 - 7.19 (1H, m, Ar); 7.18 - 7.11 (2H, m, Ar); 6.11 (1H, br. s, N*H*); 4.82 (2H, d, *J* = 5.6 Hz, C*H₂*). ^{13}C NMR ($CDCl_3$): *δ* 160.4, 158.5, 151.9, 151.7 (d, *J* = 12.6 Hz), 151.4 (d, *J* = 12.4 Hz), 149.2 (d, *J* = 12.5 Hz), 148.9 (d, *J* = 12.6 Hz), 134.1 (dd, *J* = 3.9, 5.3 Hz), 130.5, 129.9, 128.4, 124.3 (dd, *J* = 3.6, 6.4 Hz), 121.9, 117.7 (d, *J* = 17.2 Hz), 117.3 (d, *J* = 17.4 Hz), 111.7, 44.7, 44.6. HRMS(ESI) m/z: calcd for $C_{15}H_{10}N_3BrClF_2$ $(M + H)^+$: 383.97092. Found: 383.97055.

*General procedure for Suzuki coupling to obtain compounds (**6a-e**)*

Compound **5a** or **5b** (1×n), respectively, was dissolved in a mixture of toluene, dioxane and water (10/5/8, v/v/v) and K_2CO_3 (3×n), and corresponding boronic acid (1.4×n) and 5% of $PdCl_2(PPh_3)_2$ were added. The reaction mixture was stirred at 90°C overnight. Then, it was let to cool down to r.t., extracted with ethyl acetate and dichloromethane and the combined organic layers were washed with water and brine and dried over $MgSO_4$. After filtration, they were evaporated and the residue was subjected to column chromatography to give the products **6a-e**, respectively. Es an eluent a mixture of ethyl acetate/methanol 10/1 (v/v) was used.

2-Chloro-*N*-(4-methoxybenzyl)-7-(4-methoxyphenyl)-4-aminoquinazoline(**6a**, white solid, 64.8%): ^1H NMR ($CDCl_3$): *δ* 7.94 (1H, dd, *J* = 0.8, 1.6 Hz, Ar); 7.68 - 7.60 (4H, m, Ar); 7.35 (2H, ddd, *J* = 2.1, 3.1, 8.6 Hz, Ar); 7.01 (2H, ddd, *J* = 2.1, 3.1, 8.8 Hz, Ar); 6.92 (2H, ddd, *J* = 2.1, 3.1, 8.6 Hz, Ar); 6.01 (1H, br. s, N*H*); 4.80 (2H, d, *J* = 5.1 Hz, C*H₂*); 3.87 (3H, s, OC*H₃*); 3.82 (3H, s, OC*H₃*). ^{13}C NMR ($CDCl_3$): *δ* 160.4, 160.2, 159.5, 158.0, 151.4, 145.9, 131.5, 129.8, 129.3, 128.5, 125.2, 124.6, 121.2, 114.6, 114.3, 111.5, 55.4, 55.3, 45.3. HRMS(ESI) m/z: calcd for $C_{23}H_{21}N_3O_2Cl$ $(M + H)^+$: 406.13168. Found: 406.13127.

2-Chloro-7-(4-fluorophenyl)-*N*-(4-methoxybenzyl)-4-aminoquinazoline (**6b**, white solid, 58.0%): ^1H NMR ($CDCl_3$): *δ* 7.92 (1H, d, *J* = 1.8 Hz, Ar); 7.69 (1H, d, *J* = 8.5 Hz, Ar); 7.65 - 7.59 (3H, m, Ar); 7.35 (2H, ddd, *J* = 2.1, 3.0, 8.6 Hz, Ar); 7.21 - 7.12 (2H, m, Ar); 6.91 (2H, ddd, *J* = 2.1, 3.0, 8.7 Hz, Ar); 6.05 (1H, t, *J* = 5.3 Hz, N*H*); 4.80 (2H, d, *J* = 5.1 Hz, C*H₂*); 3.82 (3H, s, OC*H₃*). ^{13}C NMR ($CDCl_3$): *δ* 164.4, 161.9, 160.4, 159.5, 158.2, 151.3 145.2, 135.3 (d, *J* = 3.2 Hz), 129.8, 129.2, 129.0 (d, *J* = 8.3 Hz), 125.3, 121.4, 116.1 (d, *J* = 21.6 Hz), 114.3, 111.9, 55.3, 45.3. HRMS(ESI) m/z: calcd for $C_{22}H_{18}N_3OClF$ $(M + H)^+$: 394.11169. Found: 394.11150.

2-Chloro-*N*-(4-methoxybenzyl)-7-(thiophen-2-yl)-4-aminoquinazoline (**6c**, white solid, 86.0%): ^{1}H NMR ($CDCl_3$): *δ* 7.99 (1H, d, *J* = 1.7 Hz, Ar); 7.66 (1H, dd, *J* = 1.8, 8.6 Hz, Ar); 7.61 (1H, d, *J* = 8.6 Hz, Ar); 7.48 (1H, dd, *J* = 1.1, 3.7 Hz, Ar); 7.39 (1H, dd, *J* = 1.1, 5.1 Hz, Ar); 7.34 (2H, ddd, *J* = 2.1, 3.0, 8.6 Hz, Ar); 7.13 (1H, dd, *J* = 3.7, 5.1 Hz, Ar); 6.91 (2H, ddd, *J* = 2.1, 3.0, 8.7 Hz, Ar); 6.00 (1H, t, *J* = 5.3 Hz, N*H*); 4.78 (2H, d, *J* = 5.1 Hz, C*H₂*); 3.82 (3H, s, OC*H₃*). ^{13}C NMR ($CDCl_3$): *δ* 160.3, 159.5, 158.3, 151.4 142.3, 139.3, 129.8, 129.2, 128.5, 127.0, 125.1, 124.0, 123.5, 121.4, 114.3, 111.9, 55.3, 45.3. HRMS(ESI) m/z: calcd for $C_{20}H_{17}N_3OClS$ $(M + H)^+$: 382.07754. Found: 382.07743.

2-Chloro-*N*-(3,4-difluorobenzyl)-7-(4-methoxyphenyl)-4-aminoquinazoline (**6d**, white solid, 73.2 %): ^{1}H NMR ($CDCl_3$): *δ* 7.94 (1H, d, *J* = 1.7 Hz, Ar); 7.73 (1H, d, *J* = 8.6 Hz, Ar); 7.68 (1H, dd, *J* = 1.8, 8.6 Hz, Ar); 7.61 (2H, ddd, *J* = 2.1, 3.1, 8.7 Hz, Ar); 7.26 - 7.19 (1H, m, Ar); 7.17 - 7.11 (2H, m, Ar); 7.01 (2H, ddd, *J* = 2.1, 3.1, 8.8 Hz, Ar); 6.20 (1H, t, *J* = 5.7 Hz, N*H*); 4.84 (2H, d, *J* = 5.6 Hz, C*H₂*); 3.87 (3H, s, OC*H₃*). ^{13}C NMR ($CDCl_3$): *δ* 160.5, 160.2, 157.9, 151.6 (d, *J* = 12.7 Hz), 151.5, 151.2 (d, *J* = 12.5 Hz), 149.2 (d, *J* = 12.8 Hz), 148.8 (d, *J* = 12.5 Hz), 146.1, 134.5 (dd, *J* = 4.0, 5.1 Hz), 131.4, 128.5, 125.4, 124.6, 124.2 (dd, *J* = 3.6, 6.5 Hz), 121.1, 117.6 (d, *J* = 17.3 Hz), 117.2 (d, *J* = 17.4 Hz), 114.6, 111.4, 55.4, 44.5. HRMS(ESI) m/z: calcd for $C_{22}H_{17}N_3OClF_2$ $(M + H)^+$: 412.10227. Found: 412.102217.

2-Chloro-*N*-(3,4-difluorobenzyl)-7-(thiophen-2-yl)-4-aminoquinazoline (**6e**, white solid, 67.9 %): ^{1}H NMR ($CDCl_3$): *δ* 8.01 (1H, d, *J* = 1.8 Hz, Ar); 7.71 (1H, dd, *J* = 1.8, 8.6 Hz, Ar); 7.66 (1H, d, *J* = 8.6 Hz, Ar); 7.49 (1H, dd, *J* = 1.2, 3.7 Hz, Ar); 7.41 (1H, dd, *J* = 1.1, 5.1 Hz, Ar); 7.26 - 7.20 (1H, m, Ar); 7.19 - 7.09 (3H, m, Ar); 6.12 (1H, t, *J* = 5.9 Hz, N*H*); 4.84 (2H, d, *J* = 5.6 Hz, C*H₂*). ^{13}C NMR ($CDCl_3$): *δ* 160.4, 158.2, 151.7 (d, *J* = 12.8 Hz), 151.6, 151.3 (d, *J* = 12.4 Hz), 149.2 (d, *J* = 12.7 Hz), 148.8 (d, *J* = 12.5 Hz), 142.1, 139.6, 134.4 (dd, *J* = 3.9, 5.3 Hz), 128.5, 127.1, 125.2, 124.3, 124.2 (dd, *J* = 3.6, 6.2 Hz), 121.3, 117.7 (d, *J* = 17.1 Hz), 117.3 (d, *J* = 17.4 Hz), 111.7, 44.6. HRMS(ESI) m/z: calcd for $C_{19}H_{13}N_3ClF_2S$ $(M + H)^+$: 388.04813. Found: 388.04802.

*General procedure for the second series of quinazoline derivatives (**7a-o**)*

The synthesis followed a reported procedure.[5] The compounds **6a-e** (1×n), respectively, were dissolved in dimethylformamide and appropriate secondary amine (3×n), KI (1.1×n), K_2CO_3 (3×n) and diisopropylethylamine (DIPEA, 3×n) were added. The mixture was then heated to 110°C and stirred at this temperature overnight. Then, it was let to cool down to r.t., extracted with tetrahydrofuran and ethyl acetate and the combined organic layers were washed with water and brine and dried over $MgSO_4$. After filtration, they were evaporated and the residue was subjected to column chromatography to give the final quinazolines of the second series, **7a-o**. Es an eluent a mixture of hexane/ethyl acetate between 4/1 and 1/1 (v/v) was used.

N-(4-Methoxybenzyl)-7-(4-methoxyphenyl)-2-morpholino-4-aminoquinazoline (**7a**, white solid, 89.8 %): ^{1}H NMR ($CDCl_3$): *δ* 7.67 (1H, d, *J* = 1.8 Hz, Ar); 7.63 (2H, ddd, *J* = 2.1, 3.1, 8.9 Hz, Ar); 7.49 (1H, d, *J* = 8.4 Hz, Ar); 7.34 - 7.28 (3H, m, Ar); 6.99 (2H, ddd, *J* = 2.1, 3.1, 8.8 Hz, Ar); 6.90 (2H, ddd, *J* = 2.1, 3.1, 8.7 Hz, Ar); 5.69 (1H, t, *J* = 5.3 Hz, N*H*); 4.74 (2H, d, *J* = 5.2 Hz, C*H₂*); 3.94 - 3.88 (4H, m, 2×C*H₂*); 3.86 (3H, s, OC*H₃*); 3.82 - 3.76 (7H, m, OC*H₃*, 2×C*H₂*). ^{13}C NMR ($CDCl_3$): *δ* 159.6, 159.5, 159.4, 159.1, 152.6, 144.8, 132.8, 130.7, 129.3, 128.3, 123.1, 121.1, 120.3, 114.2, 114.1, 109.1, 67.1, 55.3, 55.3, 44.7, 44.6. HRMS(ESI) m/z: calcd for $C_{27}H_{29}N_4O_3$ $(M + H)^+$: 457.22342. Found: 457.22294.

N-(4-Methoxybenzyl)-7-(4-methoxyphenyl)-2-(pyrrolidine-1-yl)-4-aminoquinazoline (**7b**, white solid, 80.2 %): ^{1}H NMR ($CDCl_3$): *δ* 7.69 (1H, d, *J* = 1.8 Hz, Ar); 7.64 (2H, ddd, *J* = 2.1, 3.1, 8.8 Hz, Ar); 7.48 (1H, d, *J* = 8.4 Hz, Ar); 7.35 (2H, ddd, *J* = 2.1, 3.1, 8.6 Hz, Ar); 7.22 (1H, dd, *J* = 1.8, 8.4 Hz, Ar); 6.97 (2H, ddd, *J* = 2.1, 3.1, 8.8 Hz, Ar); 6.89 (2H, ddd, *J* = 2.1, 3.1, 8.6 Hz, Ar); 5.69 (1H, t, *J* = 5.4 Hz, N*H*); 4.76

(2H, d, *J* = 5.3 Hz, C*H₂*); 3.85 (3H, s, OC*H₃*); 3.81 (3H, s, OC*H₃*); 3.73 - 3.66 (4H, m, 2×C*H₂*); 2.00 - 1.95 (4H, m, 2×C*H₂*). ^{13}C NMR ($CDCl_3$): *δ* 159.5, 159.2, 159.0, 158.4, 153.1, 144.5, 133.0, 131.2, 129.3, 128.3, 122.7, 121.2, 119.2, 114.1, 114.0, 108.8, 55.3, 55.3, 46.6, 44.5, 25.6. HRMS(ESI) m/z: calcd for $C_{27}H_{29}N_4O_2$ $(M + H)^+$: 441.22850. Found: 441.22785.

N-(4-Methoxybenzyl)-7-(4-methoxyphenyl)-2-(piperazin-1-yl)-4-aminoquinazoline (**7c**, white solid, 79.6 %): ^{1}H NMR ($CDCl_3$): *δ* 7.67 (1H, d, *J* = 1.7 Hz, Ar); 7.63 (2H, ddd, *J* = 2.1, 3.1, 8.8 Hz, Ar); 7.49 (1H, d, *J* = 8.4 Hz, Ar); 7.33 (2H, ddd, *J* = 2.1, 3.1, 8.6 Hz, Ar); 7.29 - 7.26 (1H, m, Ar); 6.98 (2H, ddd, *J* = 2.1, 3.1, 8.8 Hz, Ar); 6.89 (2H, ddd, *J* = 2.1, 3.1, 8.6 Hz, Ar); 5.71 (1H, br. s, N*H*); 4.74 (2H, d, *J* = 5.2 Hz, C*H₂*); 3.93 (4H, dd, *J* = 5.0, 5.2 Hz, 2×C*H₂*); 3.85 (3H, s, OC*H₃*); 3.81 (3H, s, OC*H₃*); 2.97 (4H, dd, *J* = 5.0, 5.2 Hz, 2×C*H₂*); 2.50 (1H, br.s, N*H*). ^{13}C NMR ($CDCl_3$): *δ* 159.6, 159.5, 159.2, 159.1, 152.6, 144.8, 132.8, 130.7, 129.3, 128.3, 122.9, 121.1, 120.1, 114.2, 114.1, 109.0, 55.3, 55.3, 46.0, 44.9, 44.7. HRMS(ESI) m/z: calcd for $C_{27}H_{30}N_5O_2$ $(M + H)^+$: 456.23940. Found: 456.23894.

N^2,N^2-Diethyl-N^4-(4-methoxybenzyl)-7-(4-methoxyphenyl)-2,4-diaminoquinazoline (**7d**, white solid, 40.7 %): ^{1}H NMR ($CDCl_3$): *δ* 7.69 - 7.58 (3H, m, Ar); 7.47 (1H, d, *J* = 8.4 Hz, Ar); 7.33 (2H, ddd, *J* = 2.1, 3.1, 8.6 Hz, Ar); 7.21 (1H, dd, *J* = 1.8, 8.4 Hz, Ar); 7.08 (2H, ddd, *J* = 2.1, 3.1, 8.8 Hz, Ar); 6.89 (2H, ddd, *J* = 2.1, 3.1, 8.6 Hz, Ar); 5.62 (1H, br. s, N*H*); 4.74 (2H, d, *J* = 5.3 Hz, C*H₂*); 3.86 (3H, s, OC*H₃*); 3.81 (3H, s, OC*H₃*); 3.72 (4H, t, *J* = 7.0 Hz, 2×C*H₂*); 1.21 (6H, t, *J* = 7.0 Hz, 2×C*H₃*). ^{13}C NMR ($CDCl_3$): *δ* 159.5, 159.3, 159.0, 158.8, 153.3, 144.4, 133.1, 131.2, 129.1, 128.3, 123.0, 121.0, 119.2, 114.1, 114.0, 108.6, 55.3, 55.3, 44.6, 44.4, 13.6. HRMS(ESI) m/z: calcd for $C_{27}H_{31}N_4O_2$ $(M + H)^+$: 443.24415. Found: 443.24396.

7-(4-Fluorophenyl)-*N*-(4-methoxybenzyl)-2-morpholino-4-aminoquinazoline (**7e**, white solid, 94.9 %): ^{1}H NMR ($CDCl_3$): *δ* 7.70 - 7.61 (3H, m, Ar); 7.51 (1H, d, *J* = 8.4 Hz, Ar); 7.33 (2H, ddd, *J* = 2.1, 3.1, 8.6 Hz, Ar); 7.29 - 7.24 (1H, m, Ar); 7.19 - 7.09 (2H, m, Ar); 6.91 (2H, ddd, *J* = 2.1, 3.1, 8.6 Hz, Ar); 5.70 (1H, t, *J* = 5.3 Hz, N*H*); 4.74 (2H, d, *J* = 5.2 Hz, C*H₂*); 3.92 (4H, dd, *J* = 3.9, 5.7 Hz, 2×C*H₂*); 3.82 (3H, s, OC*H₃*); 3.79 (4H, dd, *J* = 4.0, 5.6 Hz, 2×C*H₂*). ^{13}C NMR ($CDCl_3$): *δ* 164.0, 161.6, 159.5, 159.4, 159.2, 152.6, 144.2, 136.5 (d, *J* = 3.3 Hz), 130.6, 129.3, 128.9 (d, *J* = 8.1 Hz), 123.7, 121.3, 120.3, 115.7 (d, *J* = 21.5 Hz), 114.2, 109.4, 67.1, 55.3, 44.8, 44.5. HRMS(ESI) m/z: calcd for $C_{26}H_{26}N_4O_2F$ $(M + H)^+$: 445.20343. Found: 445.20273.

7-(4-Fluorophenyl)-*N*-(4-methoxybenzyl)-2-(pyrrolidine-1-yl)-4-aminoquinazoline (**7f**, white solid, 96.6 %): ^{1}H NMR ($CDCl_3$): *δ* 7.69 - 7.61 (3H, m, Ar); 7.50 (1H, d, *J* = 8.4 Hz, Ar); 7.30 (2H, ddd, *J* = 2.1, 3.1, 8.6 Hz, Ar); 7.19 (1H, dd, *J* = 1.8, 8.4 Hz, Ar); 7.15 - 7.09 (2H, m, Ar); 6.89 (2H, ddd, *J* = 2.1, 3.1, 8.6 Hz, Ar); 5.71 (1H, br.s, N*H*); 4.76 (2H, d, *J* = 5.3 Hz, C*H₂*); 3.81 (3H, s, OC*H₃*); 3.74 - 3.65 (4H, m, 2×C*H₂*); 2.01 - 1.94 (4H, m, 2×C*H₂*). ^{13}C NMR ($CDCl_3$): *δ* 163.9, 161.5, 159.2, 159.0, 158.4, 153.1, 143.9, 136.7 (d, *J* = 3.1 Hz), 131.1, 129.3, 128.8 (d, *J* = 8.2 Hz), 123.3, 121.4, 119.3, 115.6 (d, *J* = 21.4 Hz), 114.0, 109.1, 55.3, 46.6, 44.5, 25.6. HRMS(ESI) m/z: calcd for $C_{26}H_{26}N_4OF$ $(M + H)^+$: 429.20852. Found: 429.20787.

7-(4-Fluorophenyl)-*N*-(4-methoxybenzyl)-2-(piperazin-1-yl)-4-aminoquinazoline (**7g**, white solid, 90.8 %): ^{1}H NMR ($CDCl_3$): *δ* 7.69 - 7.58 (3H, m, Ar); 7.50 (1H, d, *J* = 8.4 Hz, Ar); 7.34 (2H, ddd, *J* = 2.1, 3.1, 8.6 Hz, Ar); 7.24 (1H, dd, *J* = 1.8, 8.4 Hz, Ar); 7.17 - 7.08 (2H, m, Ar); 6.90 (2H, ddd, *J* = 2.1, 3.1, 8.6 Hz, Ar); 5.71 (1H, t, *J* = 5.3 Hz, N*H*); 4.74 (2H, d, *J* = 5.2 Hz, C*H₂*); 3.98 - 3.89 (4H, m, 2×C*H₂*); 3.82 (3H, s, OC*H₃*); 3.01 - 2.92 (4H, m, 2×C*H₂*); 2.11 (1H, br. s, N*H*). ^{13}C NMR ($CDCl_3$): *δ* 164.0, 161.5, 159.4, 159.3, 159.1, 152.7, 144.1, 136.5 (d, *J* = 3.3 Hz), 130.7, 129.3, 128.8 (d, *J* = 8.1 Hz), 123.6, 121.3, 120.1, 115.7 (d, *J* = 21.4 Hz), 114.1, 109.3, 55.3, 46.1, 45.0, 44.8. HRMS(ESI) m/z: calcd for $C_{26}H_{27}N_5OF$ $(M + H)^+$: 444.21942. Found: 444.21864.

N^2,N^2-Diethyl-7-(4-fluorophenyl)-N^4-(4-methoxybenzyl)-2,4-diaminoquinazoline (**7h**, white solid, 34.7 %): ^{1}H NMR ($CDCl_3$): δ 7.67 - 7.61 (3H, m, Ar); 7.48 (1H, d, *J* = 8.4 Hz, Ar); 7.34 (2H, ddd, *J* = 2.1, 3.1, 8.6 Hz, Ar); 7.18 (1H, dd, *J* = 1.8, 8.4 Hz, Ar); 7.15 - 7.10 (2H, m, Ar); 6.89 (2H, ddd, *J* = 2.1, 3.1, 8.6 Hz, Ar); 5.64 (1H, br.s, N*H*); 4.75 (2H, d, *J* = 5.3 Hz, C*H₂*); 3.81 (3H, s, OC*H₃*); 3.72 (4H, q, *J* = 7.0 Hz, 2×C*H₂*); 1.21 (6H, t, *J* = 7.0 Hz, 2×C*H₃*). ^{13}C NMR ($CDCl_3$): δ 163.9, 161.5, 159.3, 159.0, 158.9, 153.3, 143.8, 136.8 (d, *J* = 3.0 Hz), 131.1, 129.1, 128.9 (d, *J* = 8.1 Hz), 123.6, 121.2, 119.2, 115.6 (d, *J* = 21.3 Hz), 114.1, 109.0, 55.3, 44.6, 41.5, 13.6. HRMS(ESI) m/z: calcd for $C_{26}H_{28}N_4OF$ $(M + H)^+$: 431.22417. Found: 431.22349.

N-(4-Methoxybenzyl)-2-morpholino-7-(thiophen-2-yl)-4-aminoquinazoline (**7i**, yellowish solid, 82.6 %): ^{1}H NMR ($CDCl_3$): δ 7.72 (1H, d, *J* = 1.8 Hz, Ar); 7.47 - 7.42 (2H, m, Ar); 7.36 - 7.30 (4H, m, Ar); 7.10 (1H, dd, *J* = 3.6, 5.1 Hz, Ar); 6.89 (2H, ddd, *J* = 2.1, 3.1, 8.6 Hz, Ar); 5.67 (1H, t, *J* = 5.4 Hz, N*H*); 4.73 (2H, d, *J* = 5.2 Hz, C*H₂*); 3.91 (4H, dd, *J* = 4.0, 5.6 Hz, 2×C*H₂*); 3.82 (3H, s, OC*H₃*); 3.79 (4H, dd, *J* = 4.0, 5.6 Hz, 2×C*H₂*). ^{13}C NMR ($CDCl_3$): δ 159.4, 159.1, 152.8, 143.6, 138.3, 130.6, 129.3, 128.1, 125.8, 124.2, 122.0, 121.4, 119.3, 114.1, 109.5, 67.1, 55.3, 44.8, 44.5. HRMS(ESI) m/z: calcd for $C_{24}H_{25}N_4O_2S$ $(M + H)^+$: 433.16927. Found: 433.16909.

N-(4-Methoxybenzyl)-2-(pyrrolidin-1-yl)-7-(thiophen-2-yl)-4-aminoquinazoline (**7j**, yellow solid, 87.6 %): ^{1}H NMR ($CDCl_3$): δ 7.74 (1H, d, *J* = 1.8 Hz, Ar); 7.44 (1H, dd, *J* = 1.1, 3.6 Hz, Ar); 7.34 (2H, ddd, *J* = 2.1, 3.1, 8.6 Hz, Ar); 7.31 (1H, dd, *J* = 1.1, 5.1 Hz, Ar); 7.28 - 7.24 (2H, m, Ar); 7.09 (1H, dd, *J* = 3.6, 5.1 Hz, Ar); 6.89 (2H, ddd, *J* = 2.1, 3.1, 8.6 Hz, Ar); 5.66 (1H, br.s, N*H*); 4.75 (2H, d, *J* = 5.4 Hz, C*H₂*); 3.81 (3H, s, OC*H₃*); 3.74 - 3.58 (4H, m, 2×C*H₂*); 2.00 - 1.95 (4H, m, 2×C*H₂*). ^{13}C NMR ($CDCl_3$): δ 159.1, 159.0, 158.4, 153.1, 143.9, 138.0, 131.1, 129.3, 128.0, 125.6, 124.0, 121.7, 121.4, 118.3, 114.0, 109.2, 55.3, 46.6, 44.5, 25.6. HRMS(ESI) m/z: calcd for $C_{24}H_{25}N_4OS$ $(M + H)^+$: 417.17436. Found: 417.17423.

N-(3,4-Difluorobenzyl)-7-(4-methoxyphenyl)-2-morpholino-4-aminoquinazoline (**7k**, white solid, 90.1 %): ^{1}H NMR ($CDCl_3$): δ 7.68 (1H, d, *J* = 1.8 Hz, Ar); 7.63 (2H, ddd, *J* = 2.1, 3.1, 8.8 Hz, Ar); 7.53 (1H, d, *J* = 8.4 Hz, Ar); 7.33 (1H, dd, *J* = 1.8, 8.4 Hz, Ar); 7.21 (1H, ddd, *J* = 2.1, 7.5, 11.2 Hz, Ar); 7.19 - 7.08 (2H, m, Ar); 6.99 (2H, ddd, *J* = 2.1, 3.1, 8.8 Hz, Ar); 5.82 (1H, t, *J* = 5.7 Hz, N*H*); 4.77 (2H, d, *J* = 5.6 Hz, C*H₂*); 3.89 - 3.84 (7H, m, OC*H₃*, 2×C*H₂*); 3.79 - 3.74 (4H, m, 2×C*H₂*). ^{13}C NMR ($CDCl_3$): δ 159.7, 159.5, 159.1, 152.7, 151.6 (d, *J* = 12.7 Hz), 150.9 (d, *J* = 12.4 Hz), 149.1 (d, *J* = 12.8 Hz), 148.4 (d, *J* = 12.5 Hz), 145.1, 135.9 (dd, *J* = 3.9, 5.2 Hz), 132.6, 128.3, 123.4 (dd, *J* = 3.4, 6.2 Hz), 123.1, 121.0, 120.5, 117.4 (d, *J* = 17.1 Hz), 116.6 (d, *J* = 17.4 Hz), 114.3, 108.9, 67.0, 55.4, 44.5, 44.1. HRMS(ESI) m/z: calcd for $C_{26}H_{25}N_4O_2F_2$ $(M + H)^+$: 463.19401. Found: 463.19327.

N-(3,4-Difluorobenzyl)-7-(4-methoxyphenyl)-2-(pyrrolidine-1-yl)-4-aminoquinazoline (**7l**, white solid, 91.8 %): ^{1}H NMR ($CDCl_3$): δ 7.67 (1H, d, *J* = 1.8 Hz, Ar); 7.62 (2H, ddd, *J* = 2.1, 3.1, 8.8 Hz, Ar); 7.55 (1H, d, *J* = 8.4 Hz, Ar); 7.27 - 7.18 (2H, m, Ar); 7.12 - 7.06 (2H, m, Ar); 6.97 (2H, ddd, *J* = 2.1, 3.1, 8.8 Hz, Ar); 5.95 (1H, t, *J* = 5.8 Hz, N*H*); 4.76 (2H, d, *J* = 5.6 Hz, C*H₂*); 3.85 (3H, s, OC*H₃*); 3.64 (4H, t, *J* = 6.5 Hz, 2×C*H₂*); 1.98 - 1.93 (4H, m, 2×C*H₂*). ^{13}C NMR ($CDCl_3$): δ 159.5, 159.2, 158.1, 153.2, 151.5 (d, *J* = 12.5 Hz), 150.7 (d, *J* = 12.6 Hz), 149.0 (d, *J* = 12.6 Hz), 148.2 (d, *J* = 12.5 Hz), 144.7, 136.4 (dd, *J* = 3.9, 5.0 Hz), 132.8, 128.3, 123.5 (dd, *J* = 3.5, 6.3 Hz), 122.7, 121.2, 119.4, 117.1 (d, *J* = 17.1 Hz), 116.6 (d, *J* = 17.4 Hz), 114.2, 108.6, 55.3, 46.6, 43.9, 43.9, 25.5. HRMS(ESI) m/z: calcd for $C_{26}H_{25}N_4OF_2$ $(M + H)^+$: 447.19909. Found: 447.19858.

N-(3,4-Difluorobenzyl)-7-(4-methoxyphenyl)-2-(piperazine-1-yl)-4-aminoquinazoline (**7m**, white solid, 84.9 %): ^{1}H NMR ($CDCl_3$): δ 7.67 (1H, d, *J* = 1.7 Hz, Ar); 7.63 (2H, ddd, *J* = 2.1, 3.1, 8.8 Hz, Ar); 7.53 (1H, d, *J* = 8.4 Hz, Ar); 7.30 (1H, dd, *J* = 1.8, 8.4 Hz, Ar); 7.25 - 7.18 (1H, m, Ar); 7.16 - 7.08 (2H, m, Ar); 6.99 (2H, ddd, *J* = 2.1, 3.1, 8.8 Hz, Ar); 5.83 (1H, t, *J* = 5.7 Hz, N*H*); 4.77 (2H, d, *J* = 5.5 Hz, C*H₂*); 3.89 - 3.84 (7H, m, OC*H₃*, 2×C*H₂*); 2.95 - 2.90 (4H, m, 2×C*H₂*); 1.86 (1H, br. s, N*H*). ^{13}C NMR ($CDCl_3$): δ 159.6,

159.4, 159.1, 152.8, 151.6 (d, J = 12.8 Hz), 150.8 (d, J = 12.6 Hz), 149.1 (d, J = 13.0 Hz), 148.4 (d, J = 12.4 Hz), 145.0, 136.1 (dd, J = 3.9, 5.0 Hz), 132.7, 128.3, 123.5 (dd, J = 3.5, 6.4 Hz), 123.0, 121.0, 120.2, 117.3 (d, J = 17.2 Hz), 116.6 (d, J = 17.5 Hz), 114.2, 108.7, 55.3, 46.1, 45.1, 44.1. HRMS(ESI) m/z: calcd for $C_{26}H_{26}N_5OF_2$ $(M + H)^+$: 462.20999. Found: 462.20961.

N^4-(3,4-Difluorobenzyl)-N^2,N^2-diethyl-7-(4-methoxyphenyl)-2,4-diaminoquinazoline (**7n**, yellow solid, 54.4 %): ^{1}H NMR ($CDCl_3$): δ 7.67 - 7.58 (3H, m, Ar); 7.52 (1H, d, J = 8.4 Hz, Ar); 7.27 - 7.23 (1H, m, Ar); 7.23 - 7.17 (1H, m, Ar); 7.14 - 7.08 (2H, m, Ar); 6.98 (2H, ddd, J = 2.1, 3.1, 8.8 Hz, Ar); 5.79 (1H, br. s, N*H*); 4.76 (2H, d, J = 5.6 Hz, C*H₂*); 3.86 (3H, s, OC*H₃*); 3.66 (4H, q, J = 7.0 Hz, 2×C*H₂*); 1.16 (6H, t, J = 7.0 Hz, 2×CH_3). ^{13}C NMR ($CDCl_3$): δ 159.5, 159.2, 158.6, 153.3, 151.6 (d, J = 13.0 Hz), 150.7 (d, J = 12.8 Hz), 149.1 (d, J = 12.9 Hz), 148.2 (d, J = 12.8 Hz), 144.7, 136.5 (dd, J = 3.9, 4.8 Hz), 133.0, 128.3, 123.2 (dd, J = 3.5, 6.3 Hz), 123.0, 121.0, 119.4, 117.2 (d, J = 17.2 Hz), 116.3 (d, J = 17.5 Hz), 114.2, 108.4, 55.3, 44.1, 44.0, 41.5, 13.5. HRMS(ESI) m/z: calcd for $C_{26}H_{27}N_4OF_2$ $(M + H)^+$: 449.21474. Found: 445.21443.

N-(3,4-Difluorobenzyl)-2-morpholino-7-(thiophen-2-yl)-4-aminoquinazoline (**7o**, yellowish solid, 90.5 %): ^{1}H NMR ($CDCl_3$): δ 7.73 (1H, d, J = 1.9 Hz, Ar); 7.54 - 7.42 (2H, m, Ar); 7.40 - 7.31 (2H, m, Ar); 7.20 (1H, ddd, J = 1.9, 7.5, 10.9 Hz, Ar); 7.16 - 7.06 (3H, m, Ar); 5.81 (1H, t, J = 5.6 Hz, N*H*); 4.76 (2H, d, J = 5.5 Hz, C*H₂*); 3.87 (4H, dd, J = 3.7, 5.8 Hz, 2×C*H₂*); 3.76 (4H, dd, J = 3.9, 5.6 Hz, 2×C*H₂*). ^{13}C NMR ($CDCl_3$): δ 159.4, 159.2, 152.8, 151.6 (d, J = 13.0 Hz), 150.7 (d, J = 12.6 Hz), 149.1 (d, J = 12.5 Hz), 148.4 (d, J = 12.7 Hz), 143.5, 138.5, 135.8 (dd, J = 3.9, 5.0 Hz), 128.2, 125.9, 124.3, 123.5 (dd, J = 3.7, 6.2 Hz), 122.1, 121.3, 119.5, 117.4 (d, J = 17.2 Hz), 116.6 (d, J = 17.6 Hz), 109.3, 67.0, 44.5, 44.2. HRMS(ESI) m/z: calcd for $C_{23}H_{21}N_4OF_2S$ $(M + H)^+$: 439.13986. Found: 439.13920.

*General procedure for 4-aminoquinazolines (**8a**-**b**)*

The synthesis followed a reported procedure.[4] (*E*)-7-Bromo-4-chloro-2-styrylquinazoline **2b** (1×n) was dissolved in toluene and corresponding amine (2×n), triethylamine (2×n) and dimethyl aminopyridine (DMAP, 0.2×n) were added. The reaction mixture was stirred at 120°C for 20 h. Then, it was evaporated, the residue dissolved in chloroform and washed with water. The chloroform was then evaporated and the residue was purified by column chromatography to give (*E*)-7-bromo-2-styryl-*N*-(*p*-tolyl)-4-aminoquinazoline **8a** and (*E*)-7-bromo-*N*-(3,4-difluorobenzyl)-2-styryl-4-aminoquinazoline **8b**, respectively. Es an eluent a mixture of hexane/ethyl acetate 4/1 (v/v) was used.

(*E*)-7-Bromo-2-styryl-*N*-(*p*-tolyl)-4-aminoquinazoline (**8a**, yellowish solid, 53.4 %): ^{1}H NMR (DMSO-d6): δ 9.83 (1H, s, Ar); 8.48 (1H, d, J = 8.9 Hz, Ar); 7.94 (1H, d, J = 2.0 Hz, Ar); 7.88 - 7.81 (3H, m, Ar, C*H*); 7.73 (1H, dd, J = 2.1, 8.8 Hz, Ar); 7.71 - 7.67 (2H, m, Ar); 7.46 - 7.40 (2H, m, Ar); 7.40 - 7.34 (1H, m, Ar); 7.28 (2H, d, J = 8.2 Hz, Ar); 7.17 (1H, d, J = 15.9 Hz, C*H*); 2.34 (3H, s, C*H₃*). ^{13}C NMR (DMSO-d6): δ 160.9, 157.2, 151.6, 137.5, 136.5, 135.7, 132.9, 129.5, 129.1, 128.9, 128.6, 127.6, 126.6, 125.3, 122.2, 113.0, 20.6. HRMS(ESI) m/z: calcd for $C_{23}H_{19}N_3Br$ $(M + H)^+$: 416.07569. Found: 416.07529.

(*E*)-7-Bromo-*N*-(3,4-difluorobenzyl)-2-styryl-4-aminoquinazoline (**8b**, yellowish solid, 93.2 %): ^{1}H NMR ($CDCl_3$): δ 8.03 (1H, dd, J = 0.6, 1.8 Hz, Ar); 7.97 (1H, d, J = 15.8 Hz, C*H*); 7.65 - 7.59 (2H, m, Ar); 7.54 (1H, dd, J = 0.6, 8.8 Hz, Ar); 7.50 (1H, dd, J = 1.8, 8.7 Hz, Ar); 7.42 - 7.37 (2H, m, Ar); 7.36 - 7.31 (1H, m, Ar); 7.31 - 7.26 (1H, m, Ar); 7.22 - 7.14 (3H, m, Ar); 5.93 (1H, t, J = 5.7 Hz, N*H*); 4.93 (2H, d, J = 5.5 Hz, C*H₂*). ^{13}C NMR ($CDCl_3$): δ 161.7, 158.6, 151.8 (d, J = 9.7 Hz), 151.6, 151.1 (d, J = 12.6 Hz), 138.2, 136.2, 135.5 (d, J = 6.6 Hz), 135.4 (d, J = 5.0 Hz), 131.0, 129.0, 128.93, 128.86, 128.8, 128.4, 128.2, 127.6, 127.2, 123.9 (dd, J = 3.6, 6.2 Hz), 121.8, 117.6 (d, J = 17.3 Hz), 117.0 (d, J = 17.4 Hz), 112.3, 105.0, 44.3. HRMS(ESI) m/z: calcd for $C_{23}H_{17}N_3BrF_2$ $(M + H)^+$: 452.05684. Found: 452.05619.

*General procedure for Suzuki coupling to obtain final quinazoline derivatives of the third series (**9a-b**)*

Compound **8a** or **8b** (1×n), respectively, was dissolved in a mixture of toluene, dioxane and water (10/5/8, v/v/v) and K_2CO_3 (3×n), and thiophene-2-boronic acid pinacol ester (1.4×n) and 5% of $PdCl_2(PPh_3)_2$ were added. The reaction mixture was stirred at 90°C overnight. Then, it was let to cool down to r.t., extracted with ethyl acetate and dichloromethane and the combined organic layers were washed with water and brine and dried over $MgSO_4$. After filtration, they were evaporated and the residue was subjected to column chromatography to give the final quinazolines of the third series, **9a-b**. Es an eluent a mixture of hexane/ethyl acetate from 4/1 to 3/1 (v/v) was used.

(*E*)-2-Styryl-*N*-(*p*-tolyl)-7-(thiophen-2-yl)-4-aminoquinazoline (**9a**, yellowish solid, 77.0 %): ^{1}H NMR ($CDCl_3$): δ 8.09 (1H, d, *J* = 1.8 Hz, Ar); 7.98 (1H, d, *J* = 15.8 Hz, C*H*); 7.80 (1H, d, *J* = 8.6 Hz, Ar); 7.78 - 7.73 (2H, m, Ar); 7.70 (1H, dd, *J* = 1.9, 8.5 Hz, Ar); 7.65 - 7.60 (2H, m, Ar); 7.50 (1H, dd, *J* = 1.2, 3.6 Hz, Ar); 7.43 - 7.37 (4H, m, Ar, N*H*); 7.36 - 7.31 (1H, m, Ar); 7.28 - 7.22 (3H, m, Ar, C*H*); 7.13 (1H, dd, *J* = 3.6, 5.1 Hz, Ar); 2.41 (3H, s, C*H$_3$*). ^{13}C NMR ($CDCl_3$): δ 161.2, 156.6, 142.7, 138.7, 138.2, 136.4, 135.9, 133.9, 129.4, 128.8, 128.7, 128.4, 127.7, 126.6, 124.8, 124.2, 123.8, 121.4, 121.2, 112.7, 21.0. HRMS(ESI) m/z: calcd for $C_{27}H_{22}N_3S$ (M + H)$^+$: 420.15289. Found: 420.15221.

(*E*)-*N*-(3,4-Difluorobenzyl)-2-styryl-7-(thiophen-2-yl)-4-aminoquinazoline (**9b**, yellowish solid, 85.3 %): ^{1}H NMR ($CDCl_3$): δ 8.08 (1H, dd, *J* = 0.7, 1.8 Hz, Ar); 7.97 (1H, d, *J* = 15.8 Hz, C*H*); 7.72 - 7.59 (4H, m, Ar); 7.50 (1H, dd, *J* = 1.2, 3.6 Hz, Ar); 7.42 - 7.36 (3H, m, Ar); 7.35 - 7.27 (2H, m, Ar); 7.23 (1H, d, *J* = 15.8 Hz, C*H*); 7.20 - 7.10 (3H, m, Ar); 6.02 (1H, t, *J* = 5.6 Hz, N*H*); 4.93 (2H, d, *J* = 5.6 Hz, C*H$_2$*). ^{13}C NMR ($CDCl_3$): δ 161.3, 158.6, 151.6 (d, *J* = 12.7 Hz), 151.0 (m, 2C), 149.2 (d, *J* = 12.7 Hz), 148.5 (d, *J* = 12.6 Hz), 142.8, 138.6, 137.6, 136.4, 135.8 (dd, J = 4.0, 5.1 Hz), 128.8, 128.7, 128.4, 127.6, 126.5, 124.7, 124.2, 123.8 (dd, *J* = 3.6, 6.4 Hz), 123.6, 121.2, 117.5 (d, *J* = 17.3 Hz), 116.9 (d, *J* = 17.4 Hz), 112.5, 105.0, 44.3. HRMS(ESI) m/z: calcd for $C_{27}H_{20}N_3F_2S$ (M + H)$^+$: 456.13405. Found: 456.13374.

COX-1 and COX-2 assay

The inhibitory activity of compounds was tested using *in vitro* enzymatic assay. Ovine COX-1 (1 unit/reaction) or human recombinant COX-2 (0.5 unit/reaction; both enzymes Sigma-Aldrich, USA) was added to 180 µL of 100 mM Tris buffer (pH 8.0), containing 5 µM porcine hematin, 18 mM L-epinephrine, and 50 µM Na_2EDTA. Ten µL of tested compound dissolved in DMSO or pure DMSO (as a blank) were added to the reaction. (*S*)-(+)-Ibuprofen and selective COX-1 inhibitor SC-560 (both Sigma-Aldrich, USA) was used as a reference inhibitor. After 5 min incubation at room temperature, reaction was initiated by 5 µL of arachidonic acid (10 µM) and incubated 20 min in 37°C. Then the reaction was stopped by 20 µL of formic acid (10% v/v). The concentration of prostaglandin E_2 (PGE_2) was measured with Prostaglandin E_2 ELISA kit (Enzo Life Sciences, USA). Reaction mixture was diluted 1:15 in assay buffer (provided in the kit) and incubated according to the manufacturer instructions. The concentration of PGE_2 produced during the reaction was estimated according to absorbance measured at 405 nm by a Tecan Infinite M200 microplate reader (Tecan Group, Switzerland). The inhibitory activity was calculated as percentual inhibition of PGE_2 production compared to blank. At first, the inhibitory activity of compounds was tested at 20 µM to see if there is any activity. IC_{50} values were determined only for compounds with at least 90 % inhibition of COX-1 or 50 % inhibition of COX-2. The level of percentual inhibition was chosen based on the percentual inhibition caused by

the reference compound ibuprofen. All the experiments were carried out in duplicates and repeated at least two times. To test whether increasing substrate concentration influences the activity of compounds, AA was used in the reaction in final 250, 1250 and 6250 nM concentrations. Compound **9b** was selected as a representative compound for the tests and (*S*)-(+)-ibuprofen was used as a reference compound. The inhibitory activity was calculated as percentual inhibition of PGE2 production compared to blank with respective concentration of AA.

References:

1. Sidhu R. S.; Lee J. Y.; Yuan C.; Smith W. L. Comparison of Cyclooxygenase-1 Crystal Structures: Cross-Talk between Monomers Comprising Cyclooxygenase-1 Homodimers. *Biochemistry*. **2010**, *49*, 33, 7069-7079.
2. Kurumbail, R. G.; Stevens, A. M.; Gierse, J. K.; McDonald, J. J.; Stegeman, R. A.; Pak, J. Y.; Gildehaus, D.; Miyashiro, J. M.; Penning, T. D.; Seibert, K.; Isakson, P. C.; Stallings, W. C. Structural Basis for Selective Inhibition of Cyclooxygenase-2 by Anti-Inflammatory Agents. *Nature*. **1996**, *384*, 644-648.
3. Jones, G.; Willet, P.; Glen, R. C.; Leach, A. R.; Taylor, R. Development and Validation of a Genetic Algorithm for Flexible Docking. *J. Mol. Biol*. **1997**, *267*, 727-748.
4. Okano, M.; Mito, J.; Maruyama, Y.; Masuda, H.; Niwa, T.; Nakagawa, S.; Nakamura, Y.; Matsuura, A. Discovery and Structure-Activity Relationships of 4-Aminoquinazoline Derivatives, a Novel Class of Opioid Receptor Like-1 (ORL1) Antagonists. *Bioorg. Med. Chem.* **2009**, *17*, 119-132.
5. Jiang, Y.; Chen, A. C.; Kuang, G. T.; Wang, S. K.; Ou, T. M.; Tan, J. H.; Li, D.; Huang, Z. S. Design, Synthesis and Biological Evaluation of 4-Anilinoquinazoline Derivatives as New c-Myc G-Quadruplex Ligands. *Eur. J. Med. Chem.* **2016**, *122*, 264-279.
