# Supplementary material for: Synthesis, Inhibitory Activity, and In Silico Modeling of Selective COX-1 Inhibitors with a Quinazoline Core
Source: ACS Med Chem Lett. 2021 Mar 12;12(4):610–6. doi: 10.1021/acsmedchemlett.1c00004 (PMC8040043; doi:10.1021/acsmedchemlett.1c00004)
Supplement: Supplementary file 2 — ml1c00004_si_002.pdf [file ml1c00004_si_002.pdf]

Synthesis, Inhibitory Activity, and *In Silico* Modeling of Selective COX-1 Inhibitors with Quinazoline Core

Marcela Dvorakova[a,*], Lenka Langhansova[a], Veronika Temml[b], Antonio Pavicic[a], Tomas Vanek[a], Premysl Landa[a]

[a] Laboratory of Plant Biotechnologies, Czech Academy of Sciences, Institute of Experimental Botany, Rozvojova 263, 165 02 Prague 6 - Lysolaje, Czech Republic

[b] Department of Pharmaceutical and Medicinal Chemistry, Paracelsus Medical University of Salzburg, Strubergasse 21, 5020 Salzburg, Austria

Supporting Information

Table of Contents

^{1}H-NMR and ^{13}C-NMR spectra of compounds **3a-v**......S2-S23

^{1}H-NMR and ^{13}C-NMR spectra of compounds **5a-b**......S24-S25

^{1}H-NMR and ^{13}C-NMR spectra of compounds **6a-e**......S26-S30

^{1}H-NMR and ^{13}C-NMR spectra of compounds **7a-o**......S31-S45

^{1}H-NMR and ^{13}C-NMR spectra of compounds **8a-b**......S46-S47

^{1}H-NMR and ^{13}C-NMR spectra of compounds **9a-b**......S48-S49

Table 1S. Percentual inhibition of COX-1 and COX-2 isoenzymes by tested compounds at 20 µM....S50

Table 2S. COX-1 inhibition by compound **9b** and Ibuprofen at different substrate concentrations...S50

Graph 1S. COX-1 inhibitory activity in % vs. log(concentration of the most active compounds)........S51

UPLC/UV-VIS chromatogram_Blanks......S51-S52

UPLC/UV-VIS chromatograms of the active compounds......S52-S57

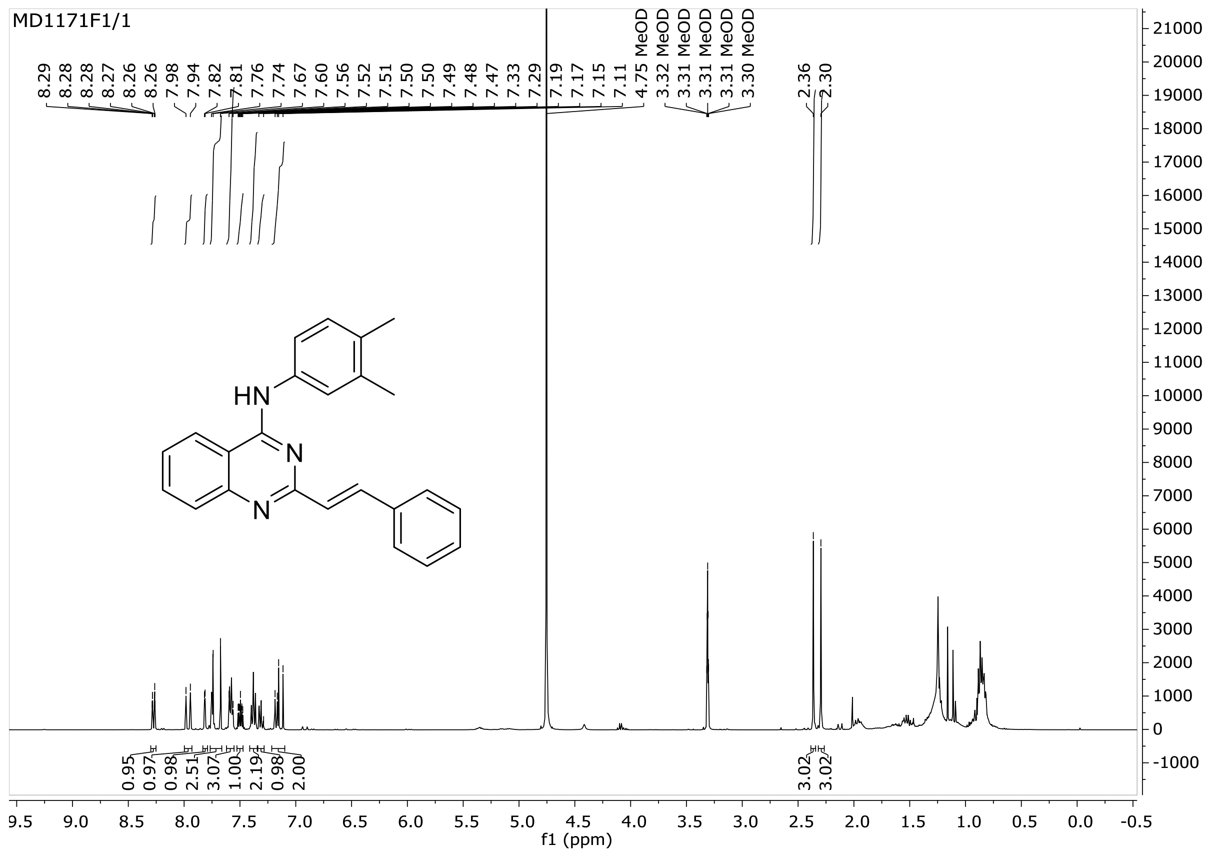

^{1}H-NMR spectrum of compound **3a**

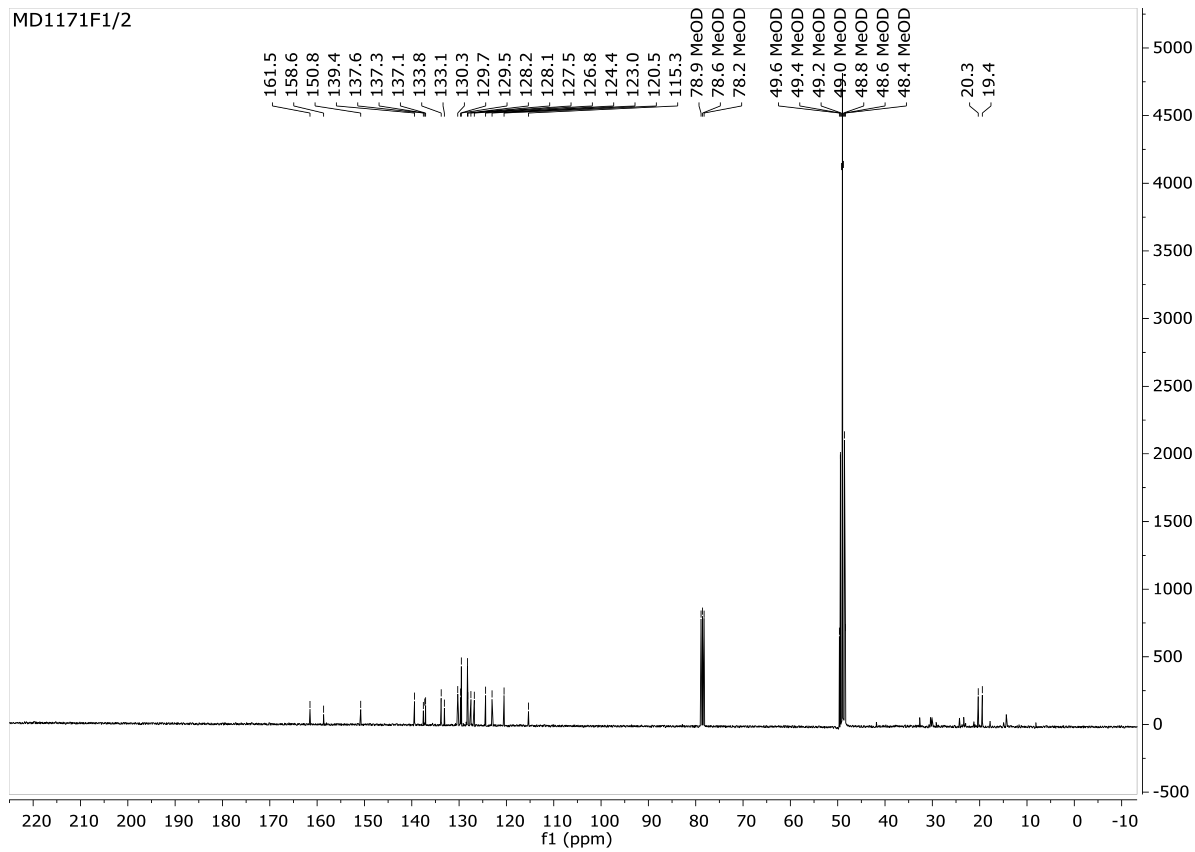

^{13}C-NMR spectrum of compound **3a**

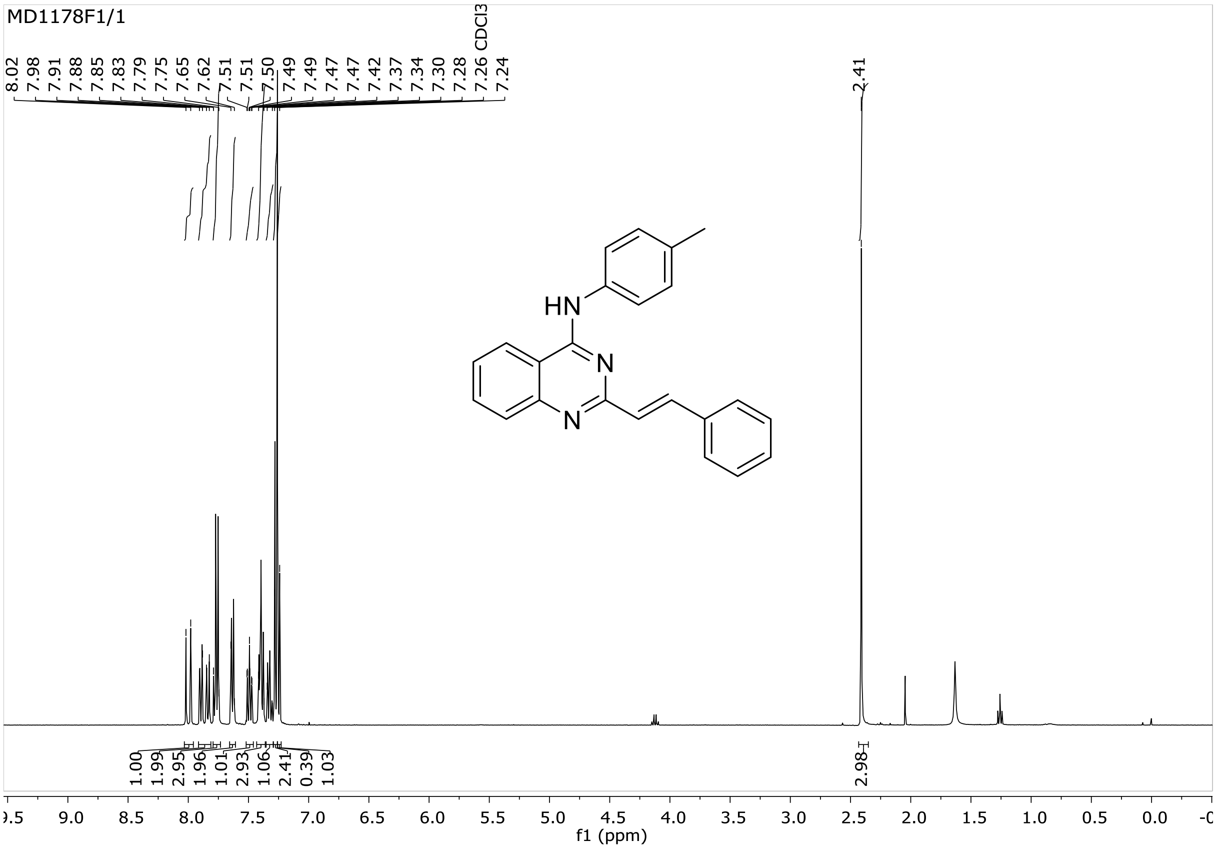

^{1}H-NMR spectrum of compound **3b**

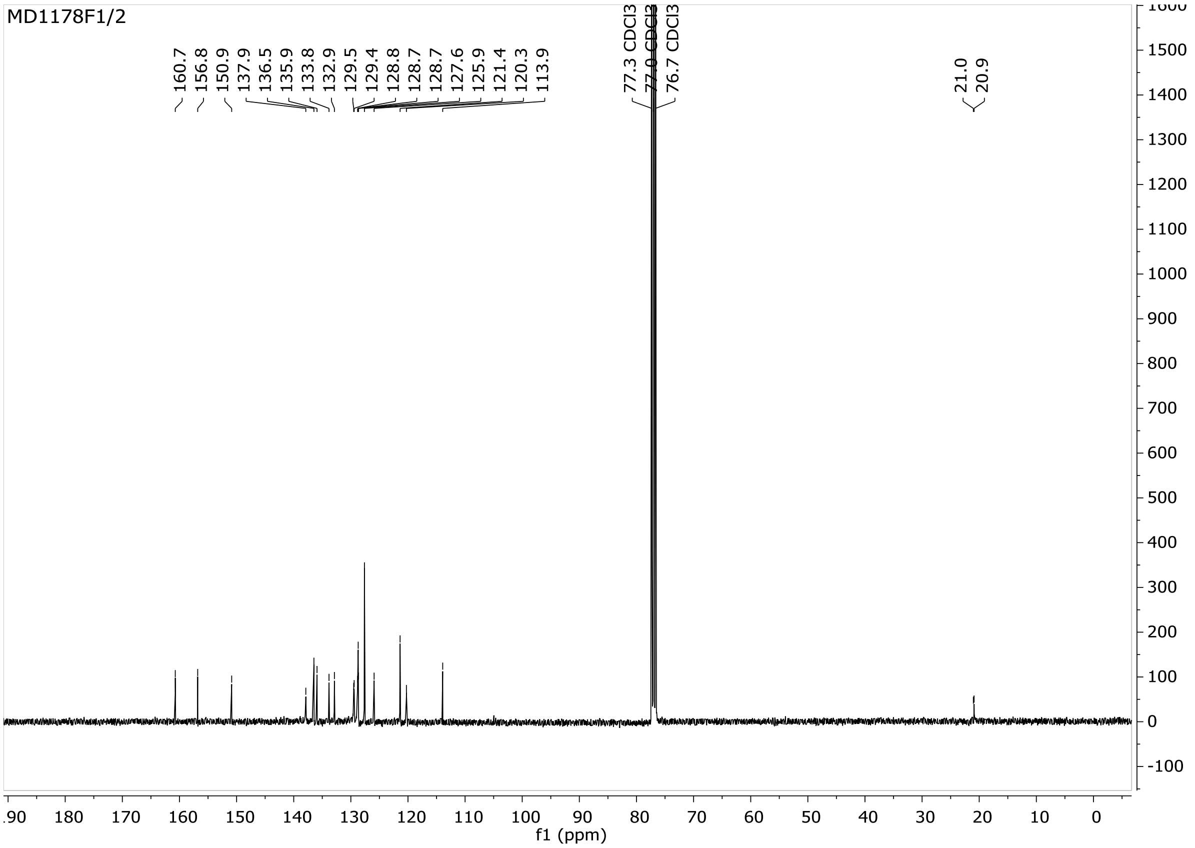

^{13}C-NMR spectrum of compound **3b**

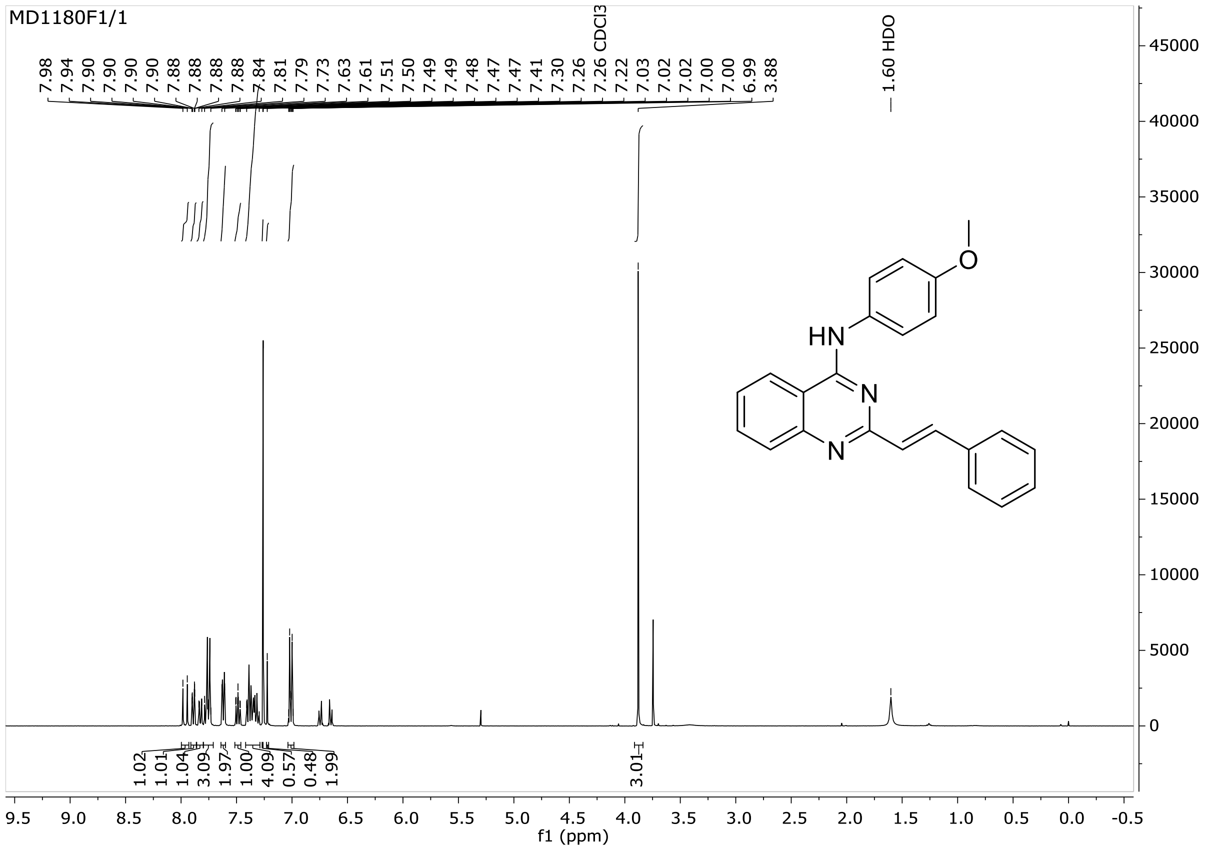

^{1}H-NMR spectrum of compound **3c**

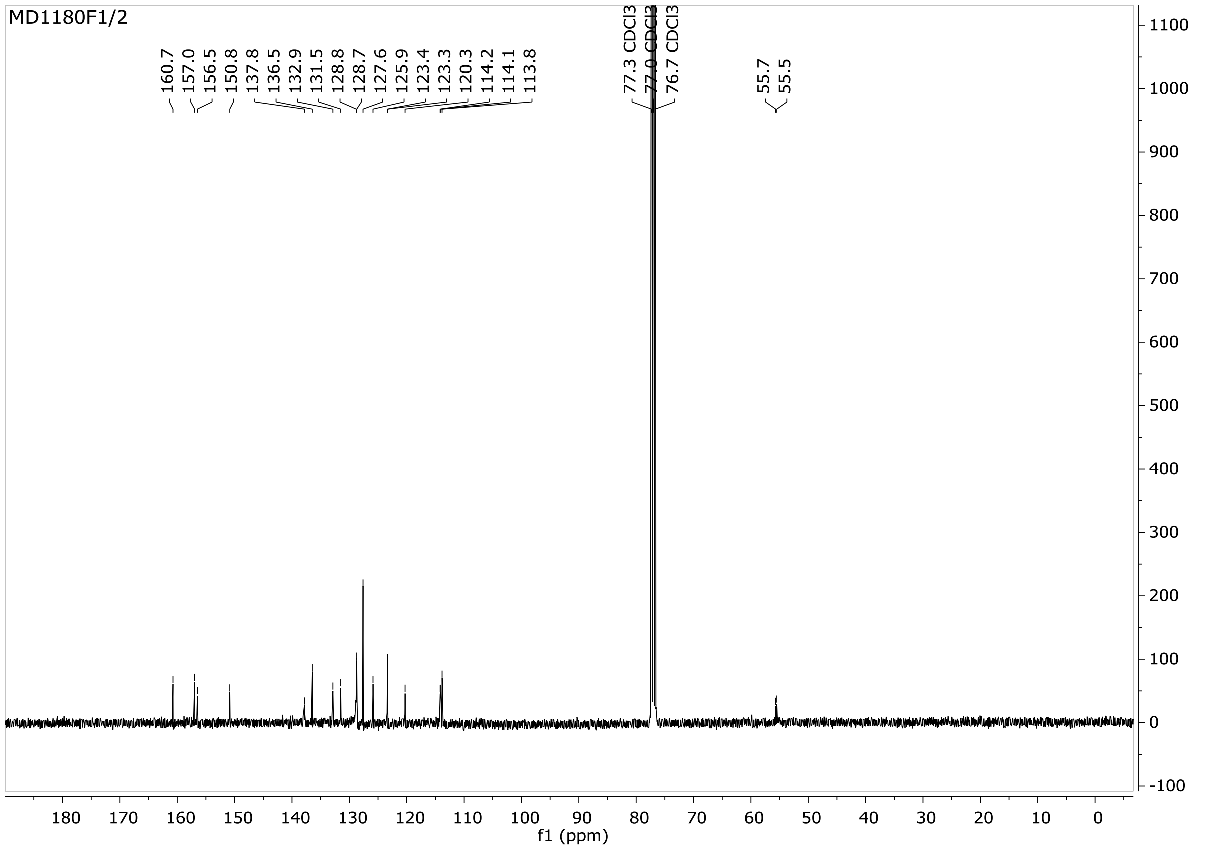

^{13}C-NMR spectrum of compound **3c**

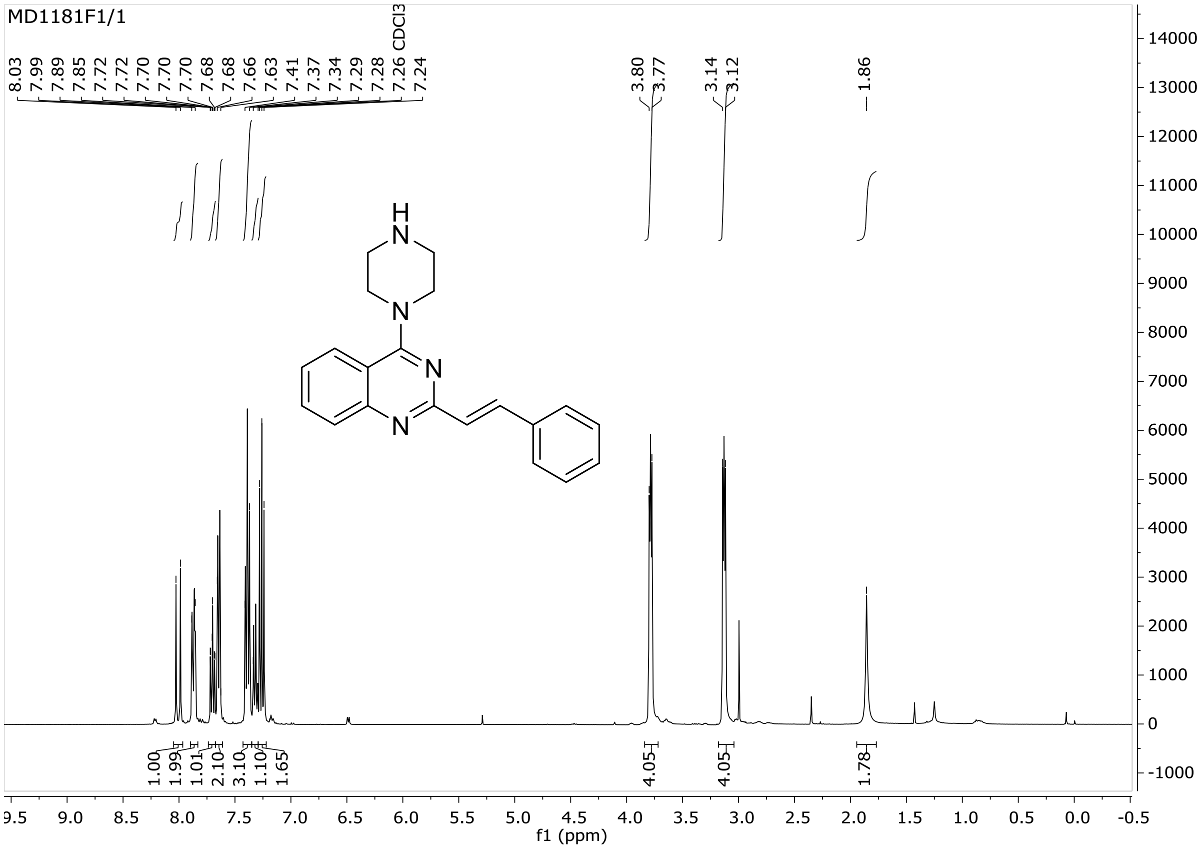

^{1}H-NMR spectrum of compound **3d**

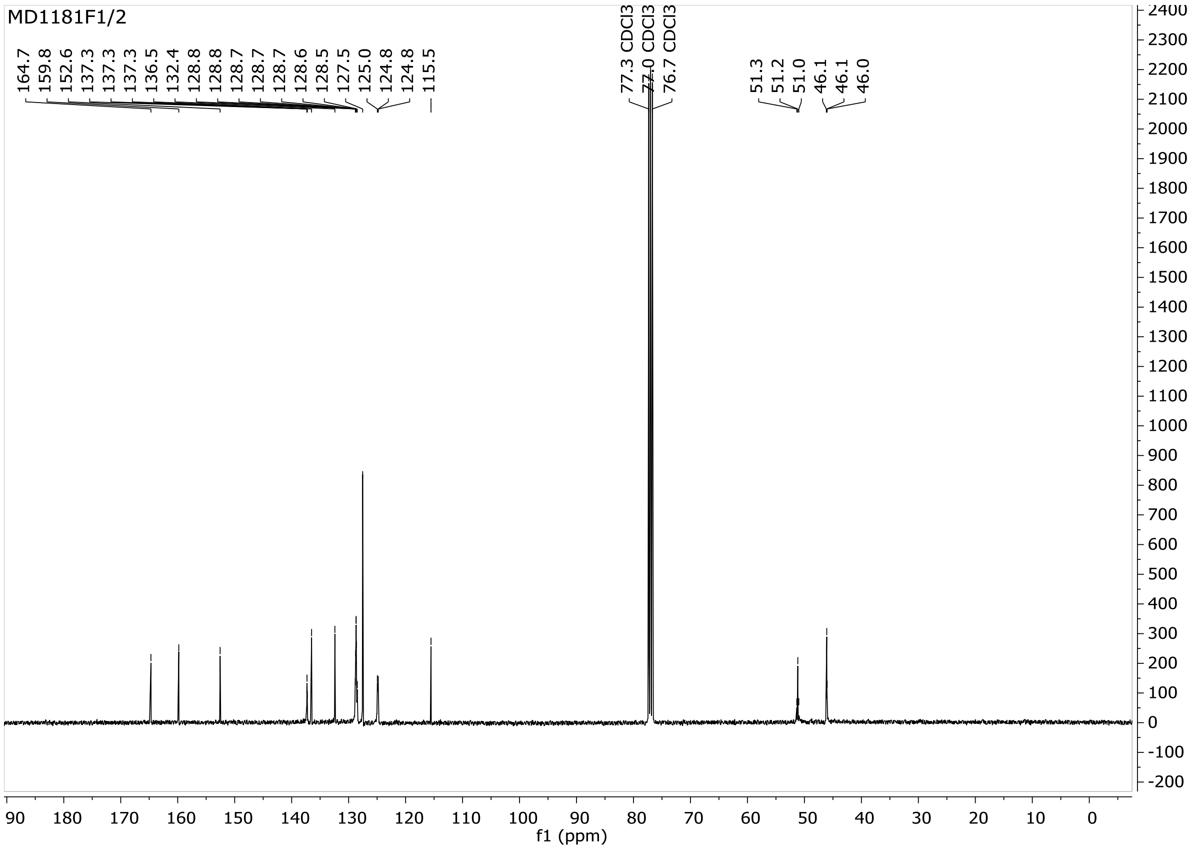

^{13}C-NMR spectrum of compound **3d**

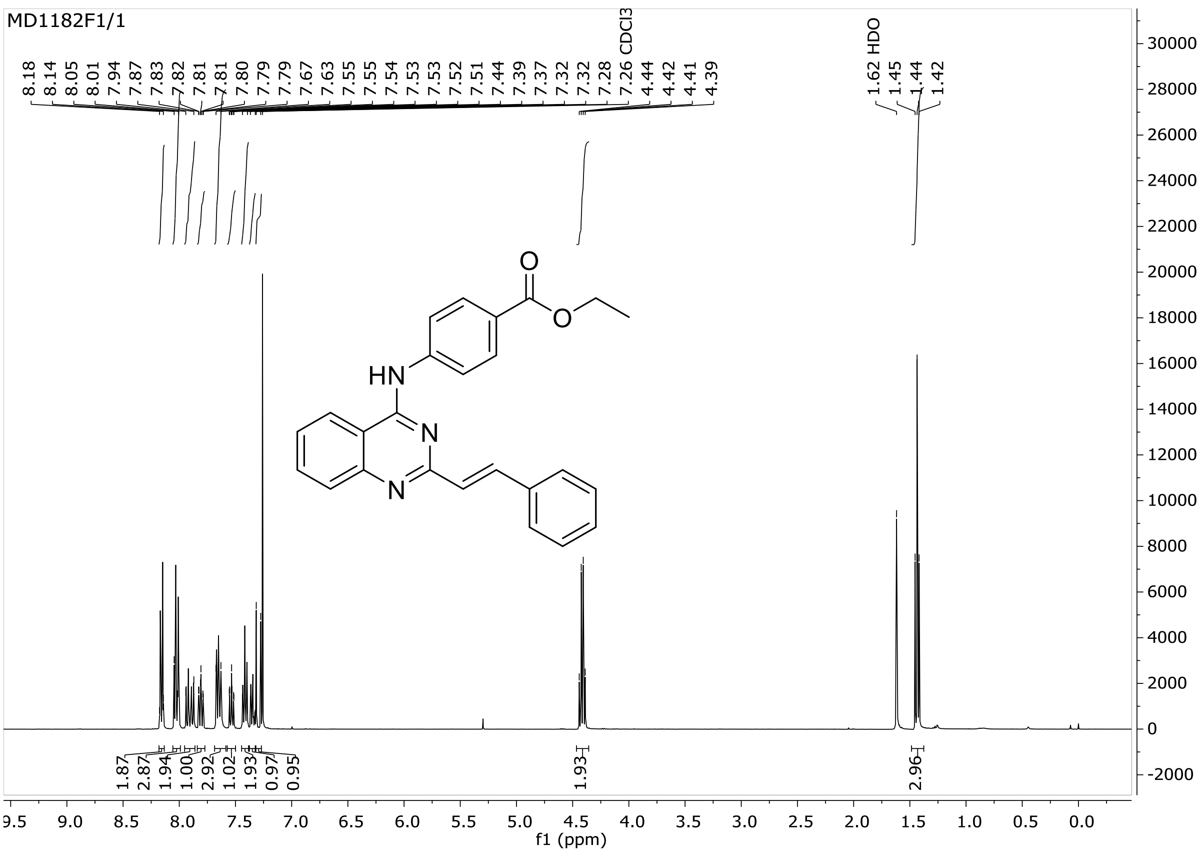

^{1}H-NMR spectrum of compound **3e**

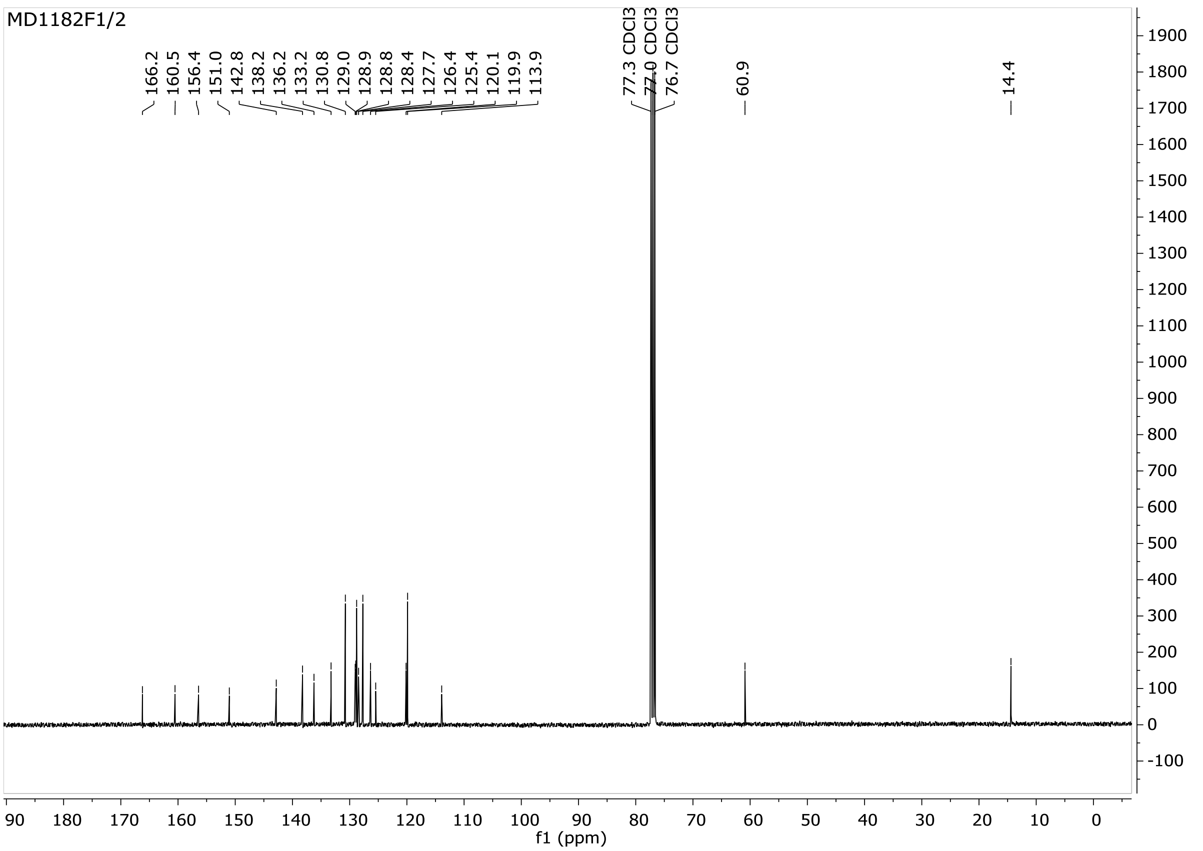

^{13}C-NMR spectrum of compound **3e**

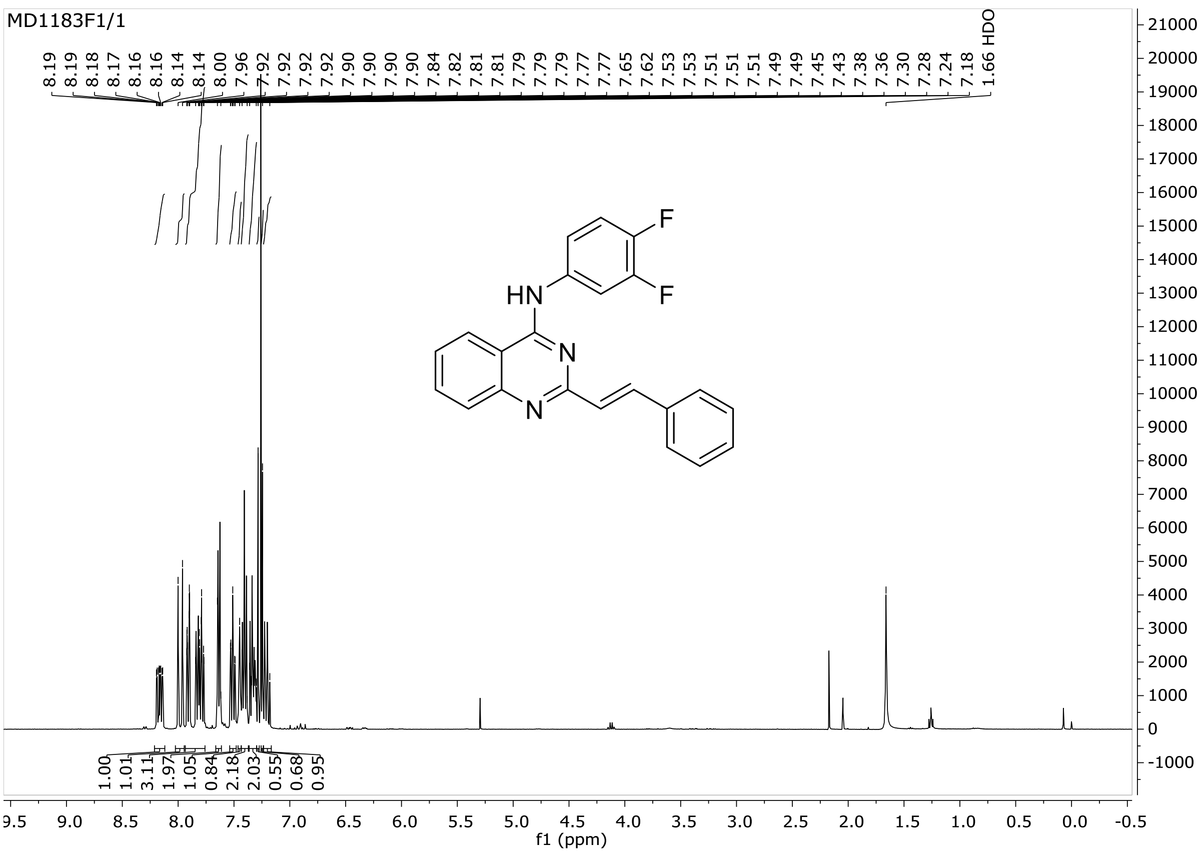

^{1}H-NMR spectrum of compound **3f**

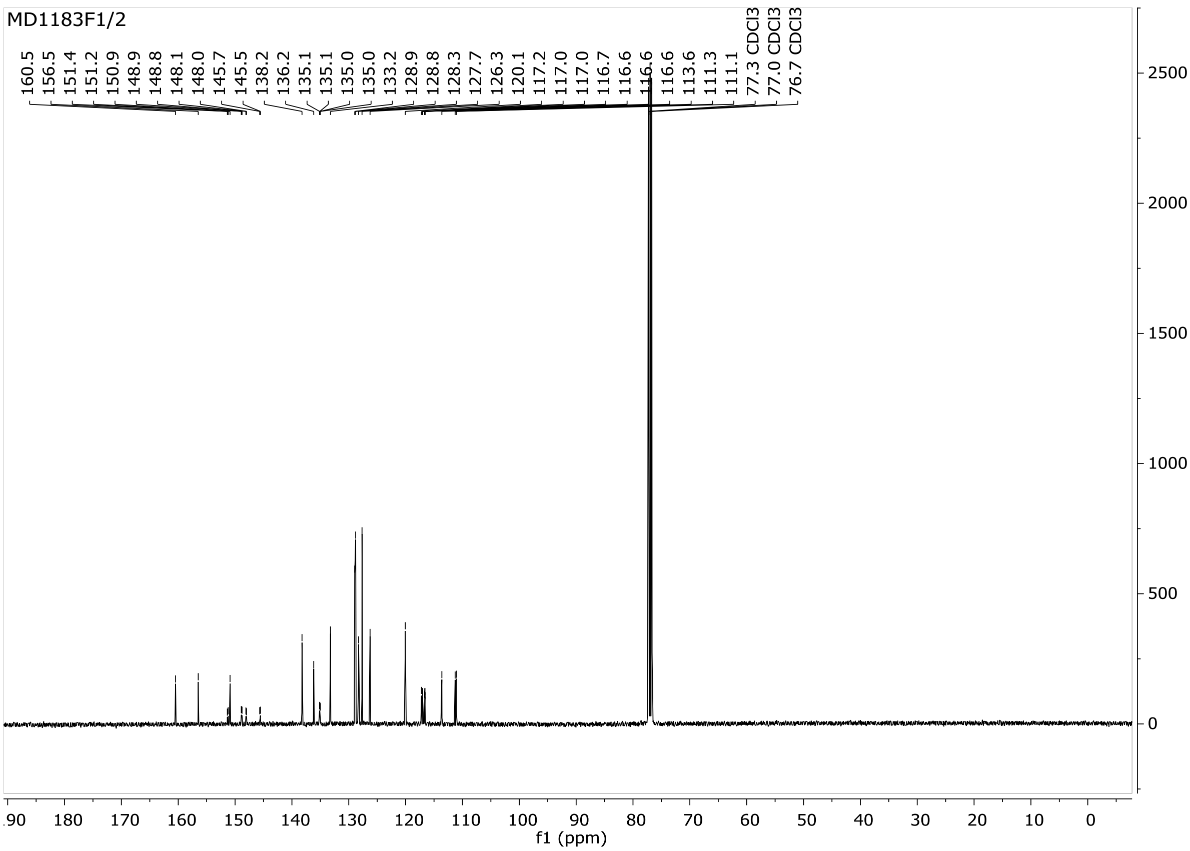

^{13}C-NMR spectrum of compound **3f**

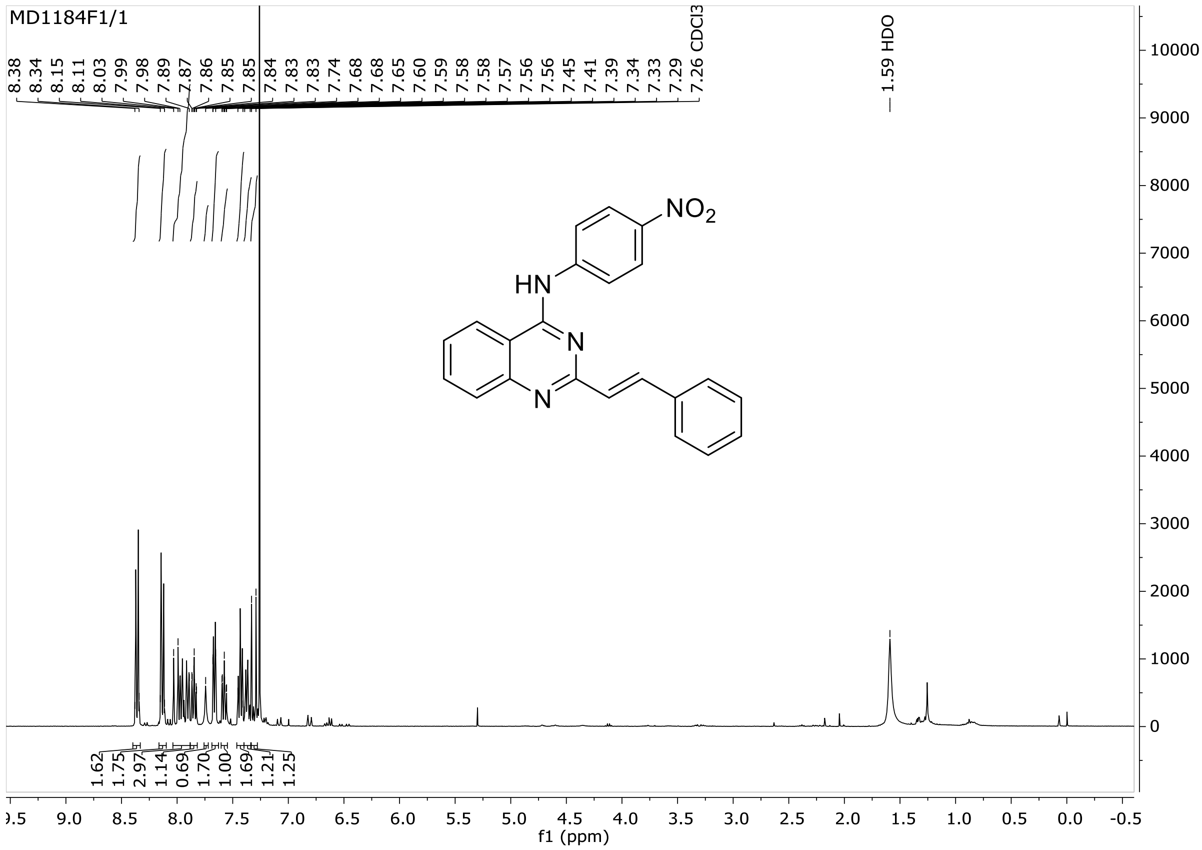

^{1}H-NMR spectrum of compound **3g**

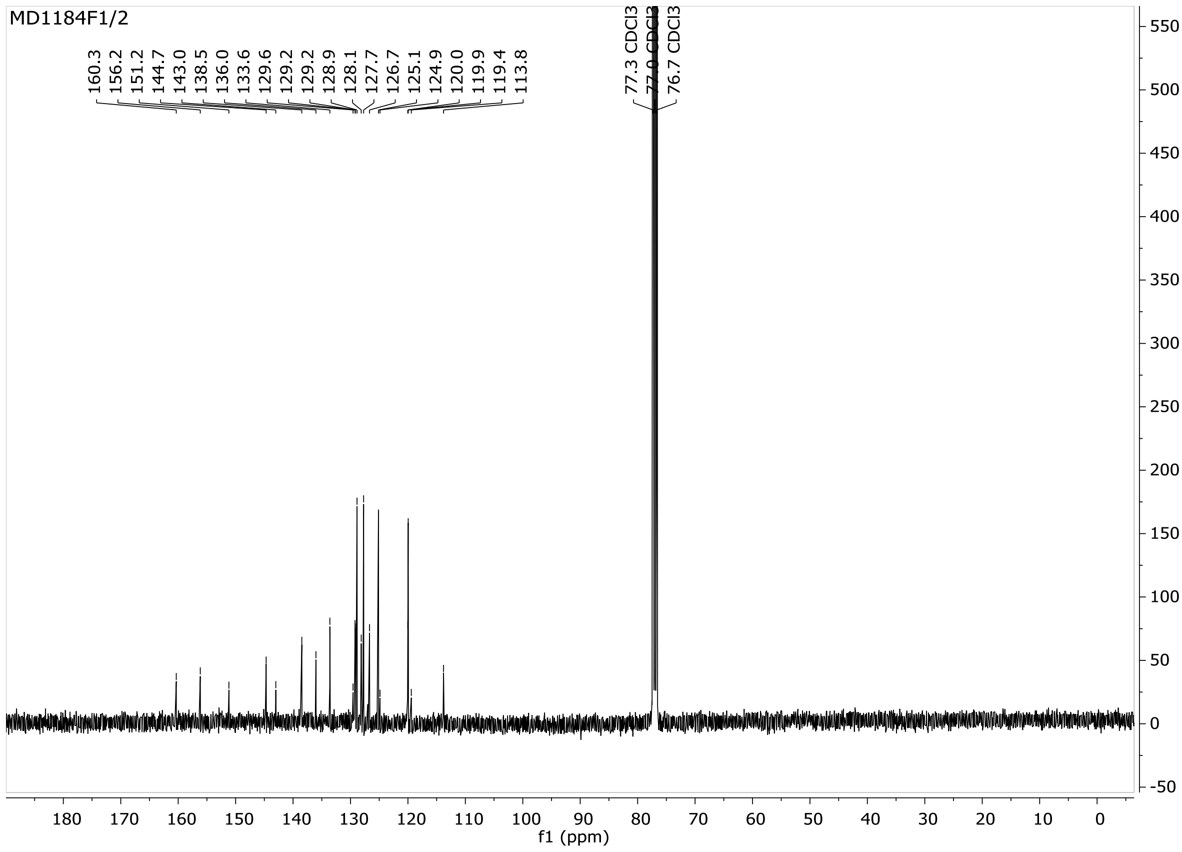

^{1}H-NMR spectrum of compound **3g**

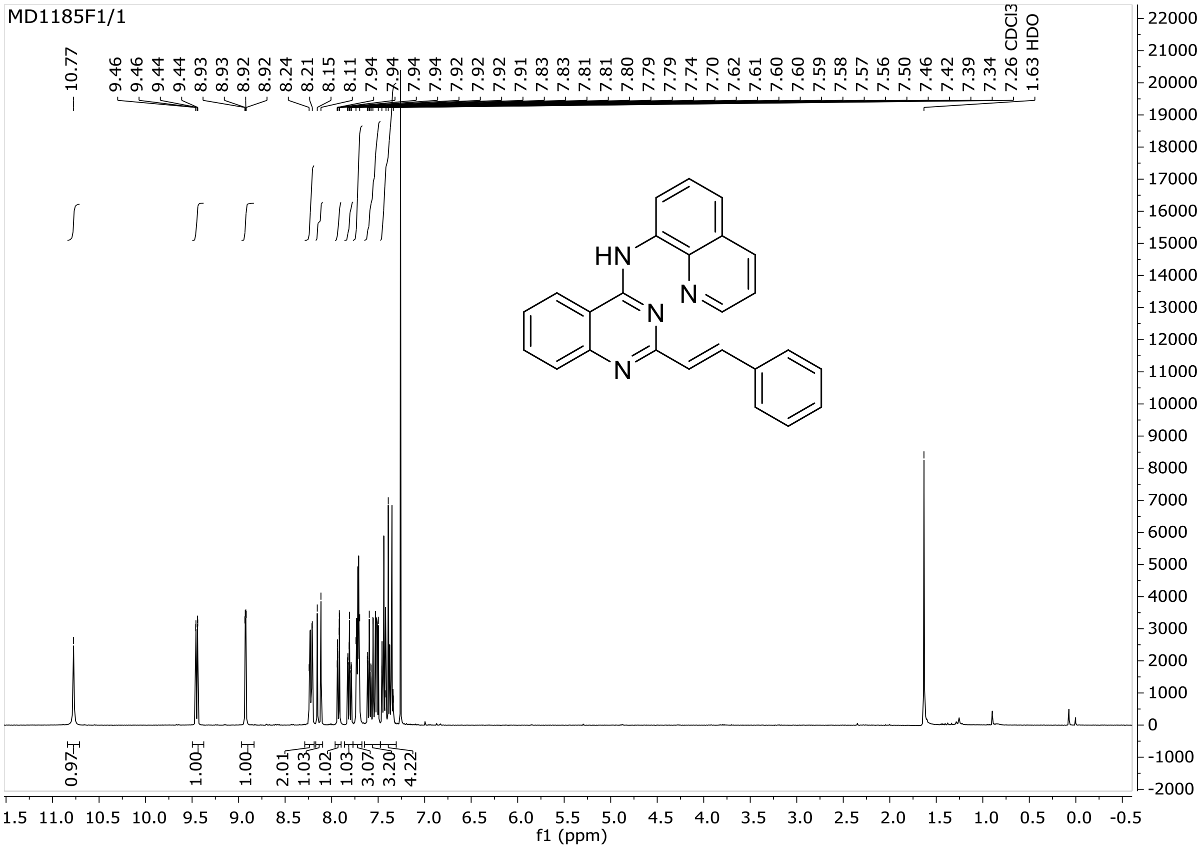

^{1}H-NMR spectrum of compound **3h**

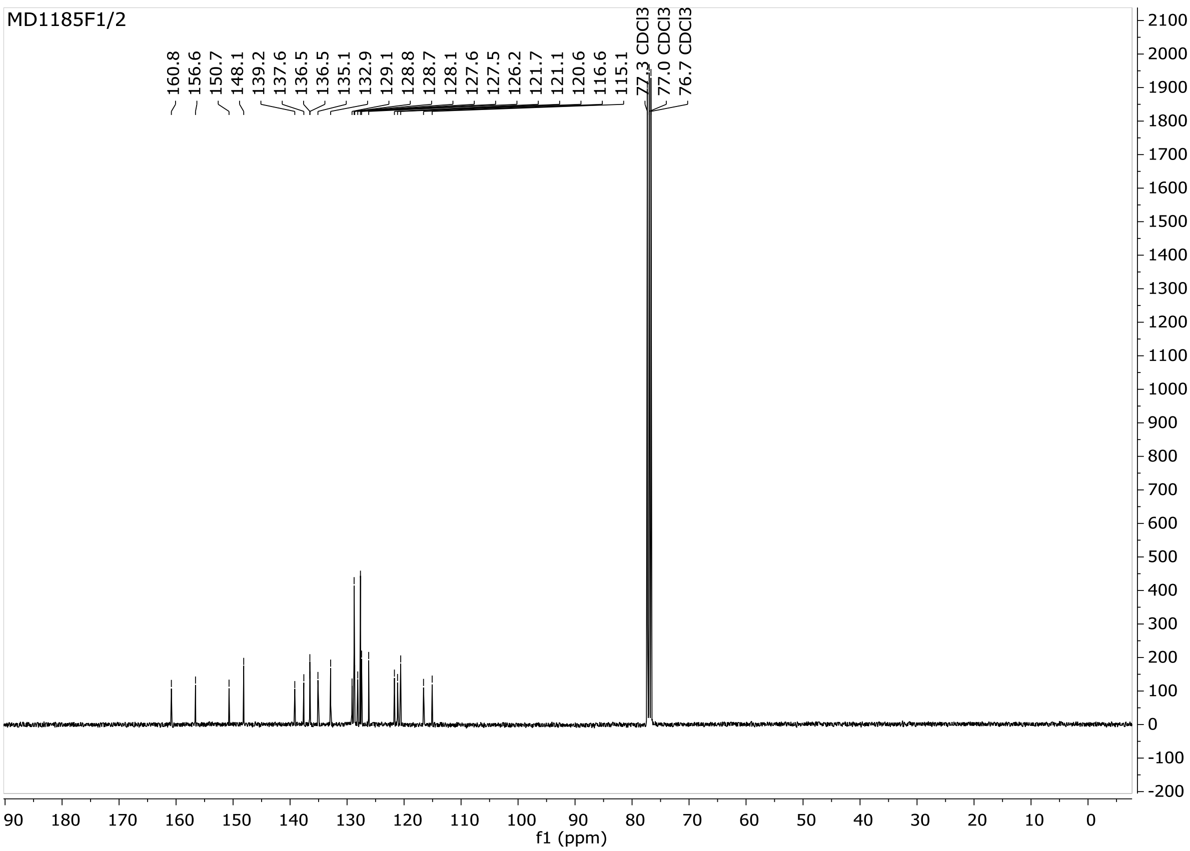

^{13}C-NMR spectrum of compound **3h**

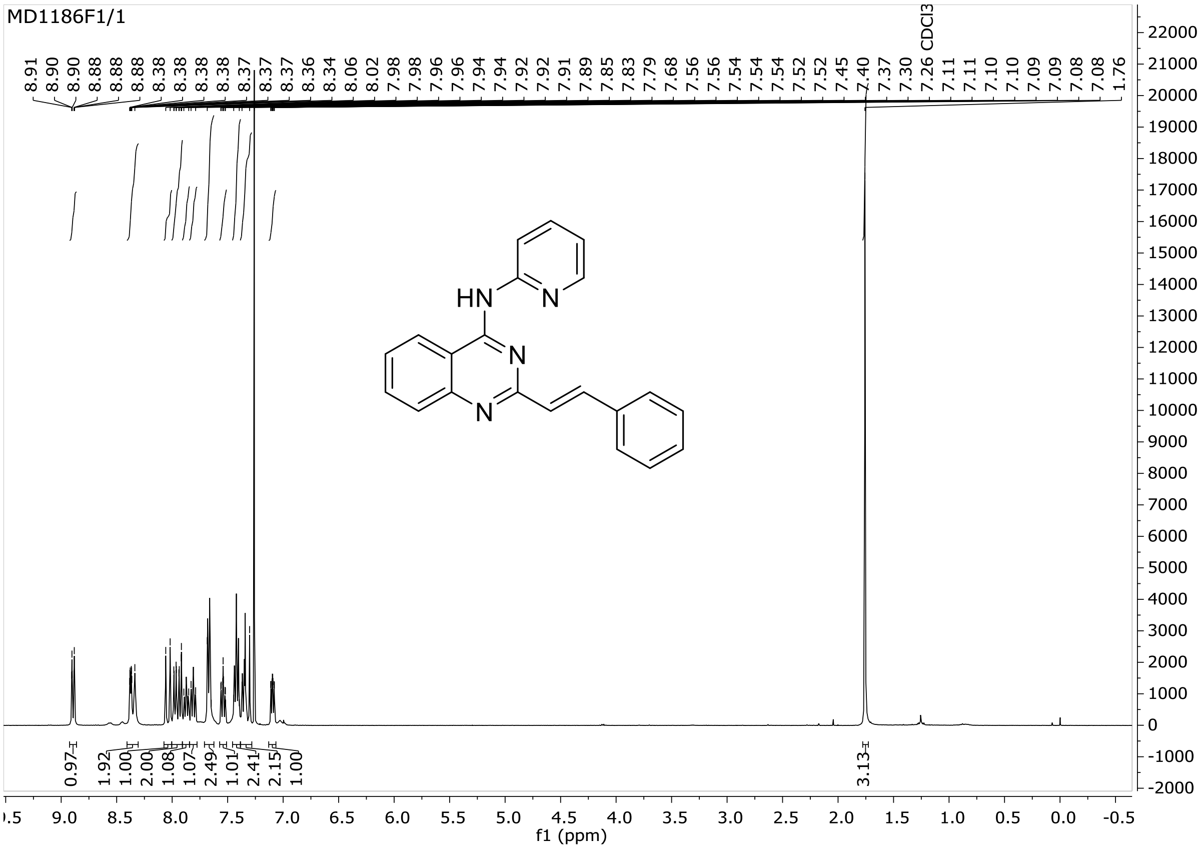

^{1}H-NMR spectrum of compound **3i**

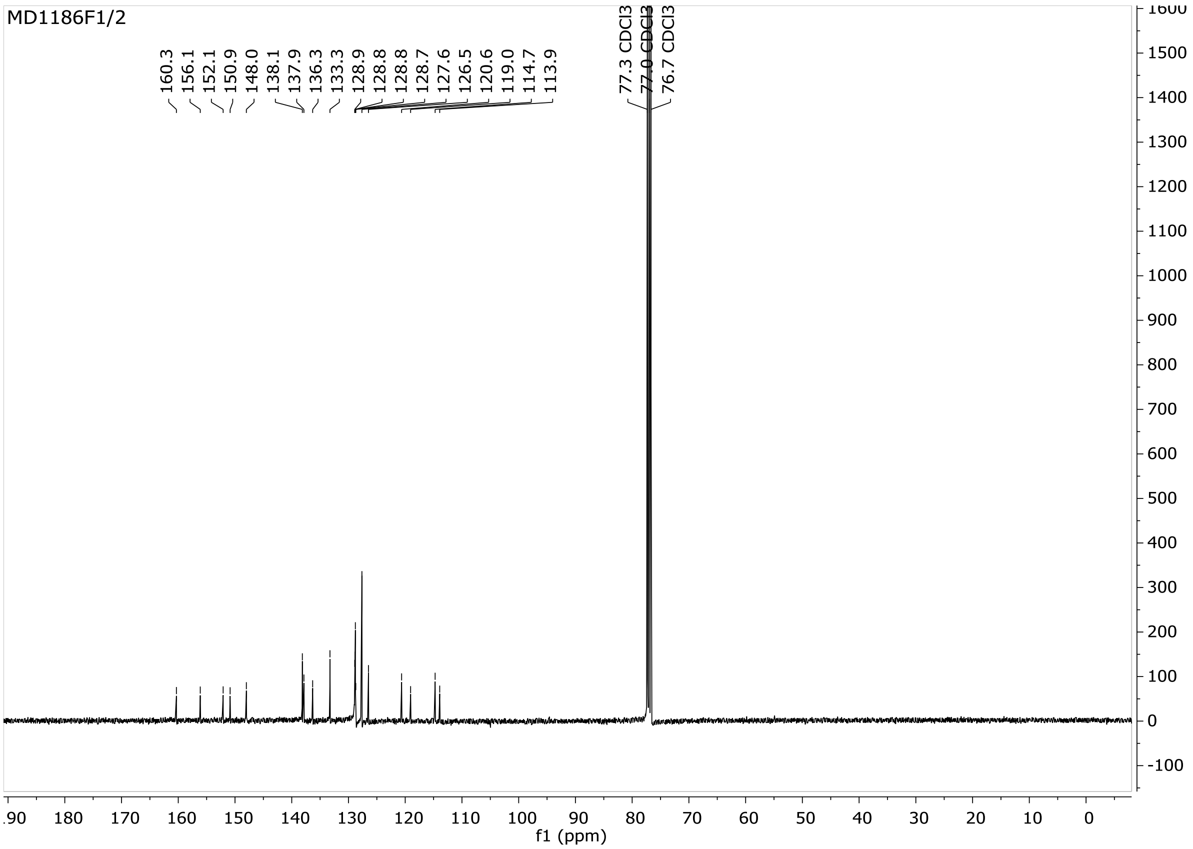

^{13}C-NMR spectrum of compound **3i**

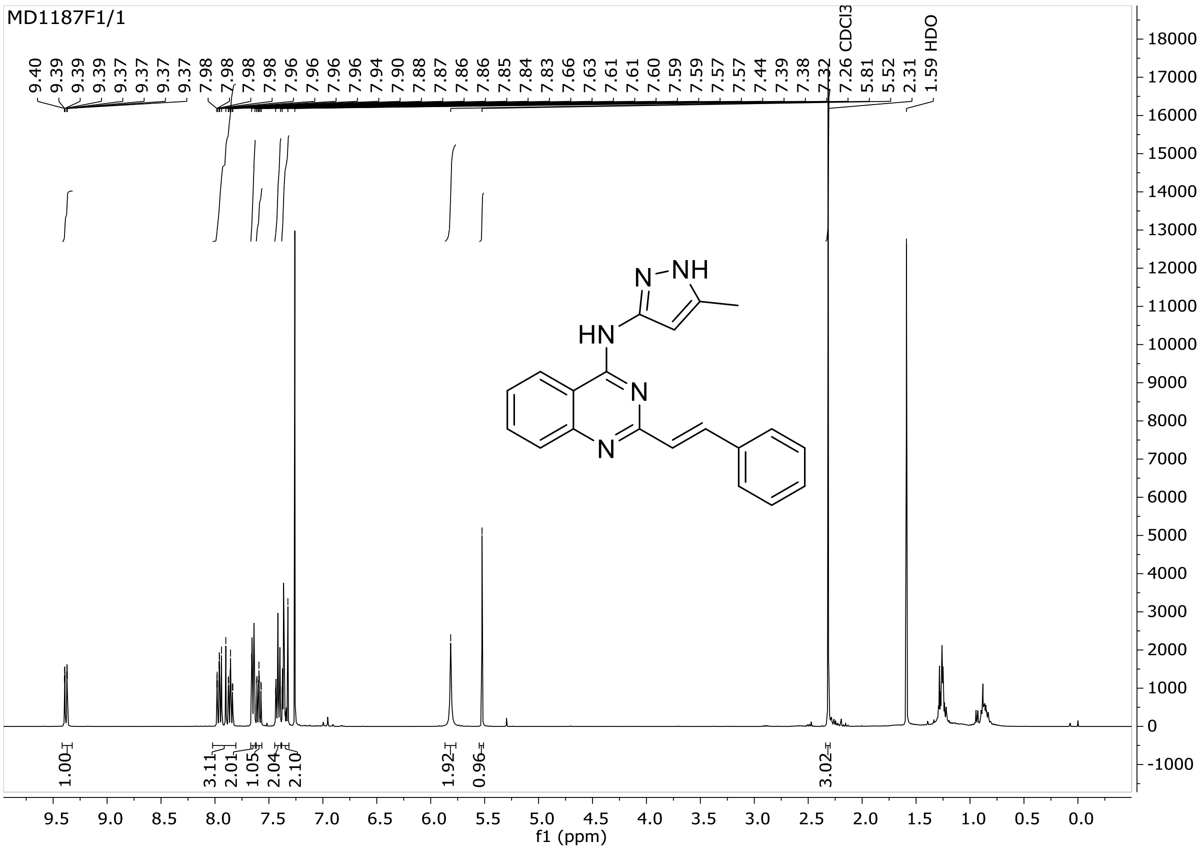

^{1}H-NMR spectrum of compound **3j**

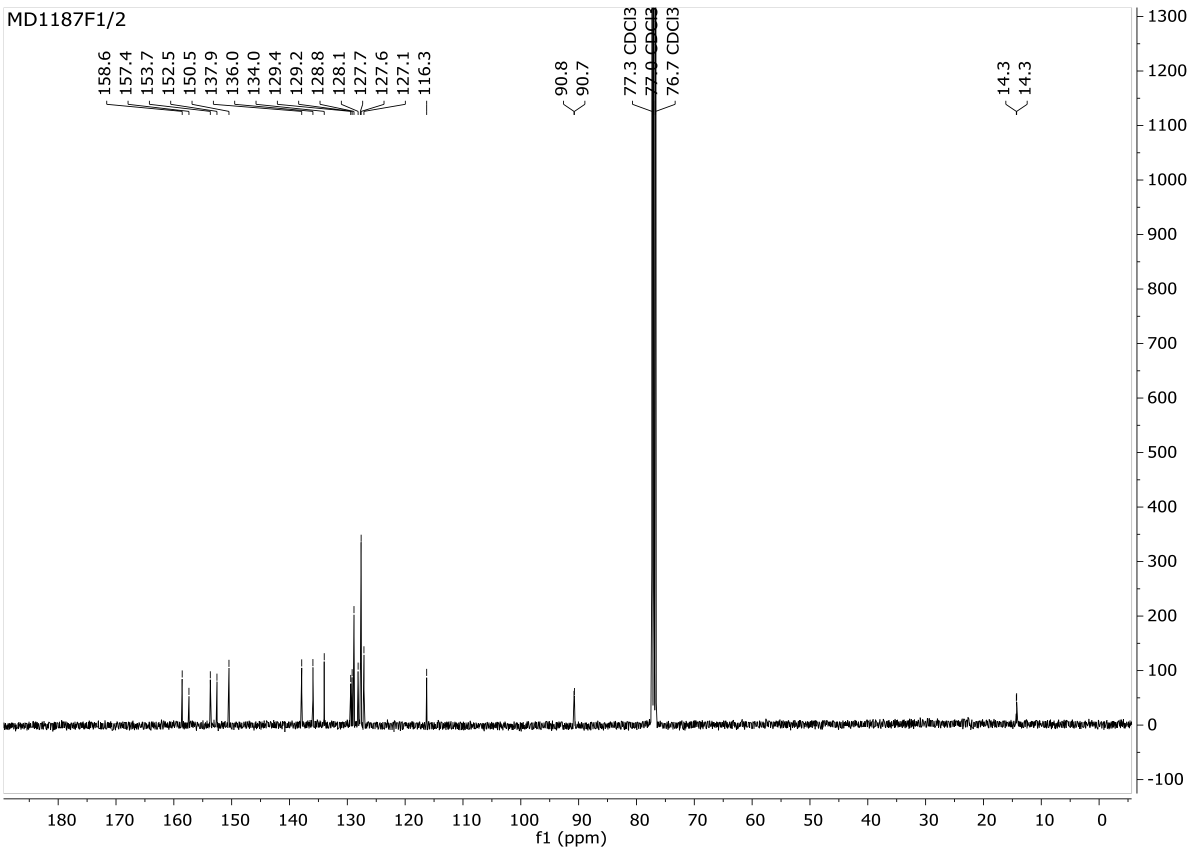

^{13}C-NMR spectrum of compound **3j**

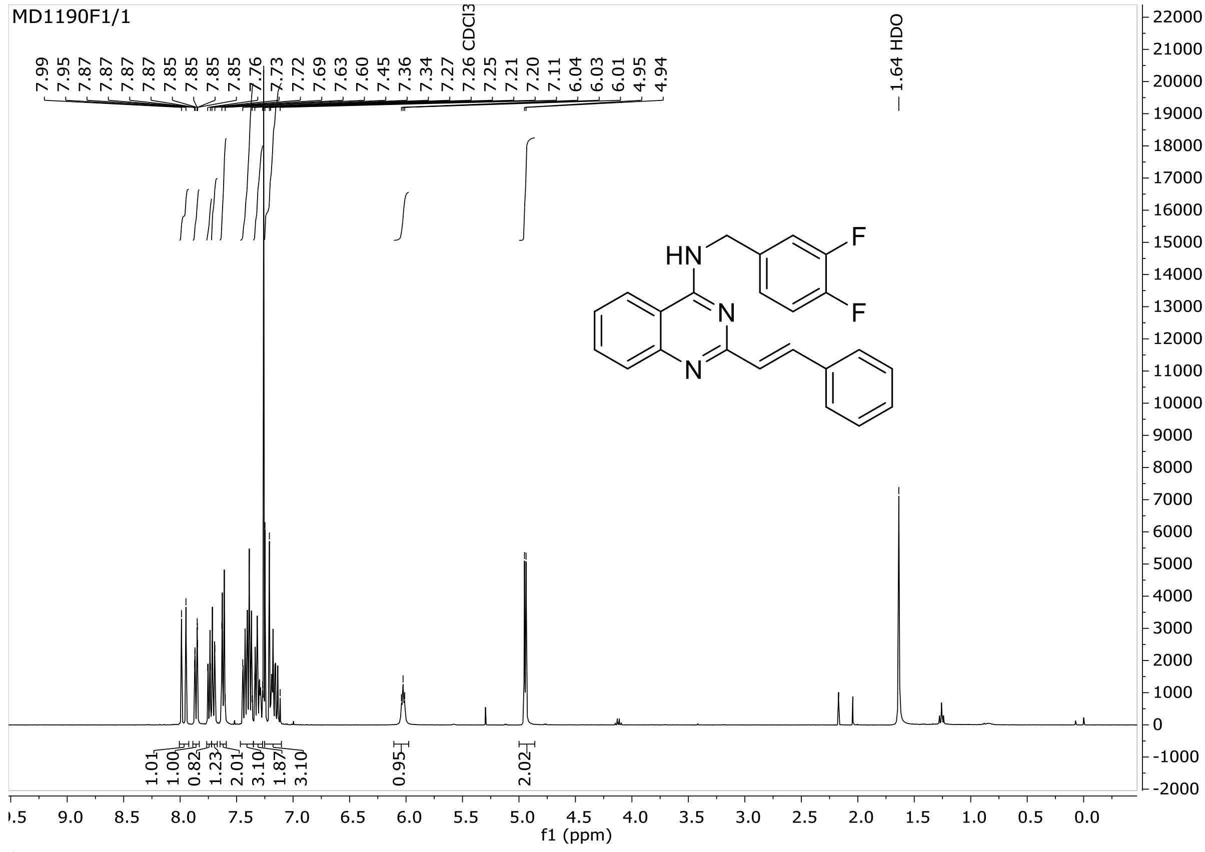

^{1}H-NMR spectrum of compound **3k**

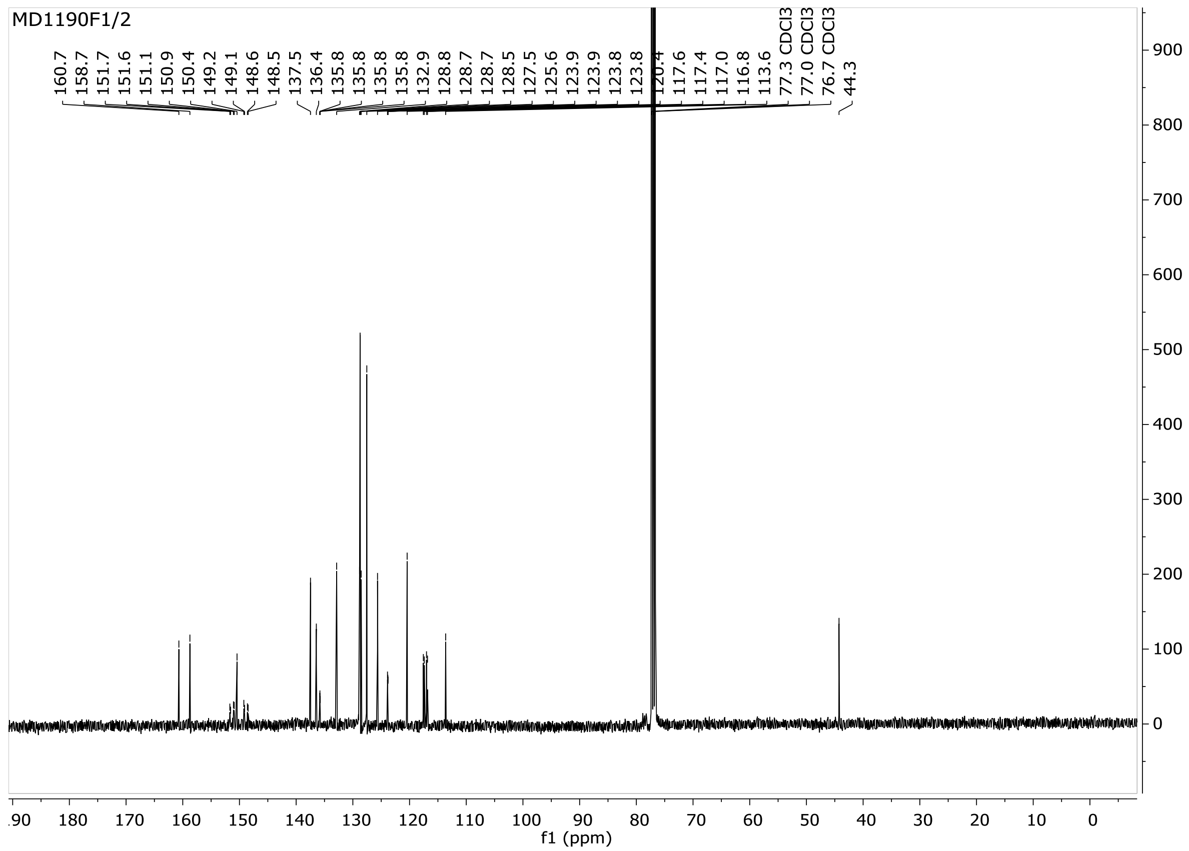

^{13}C-NMR spectrum of compound **3k**

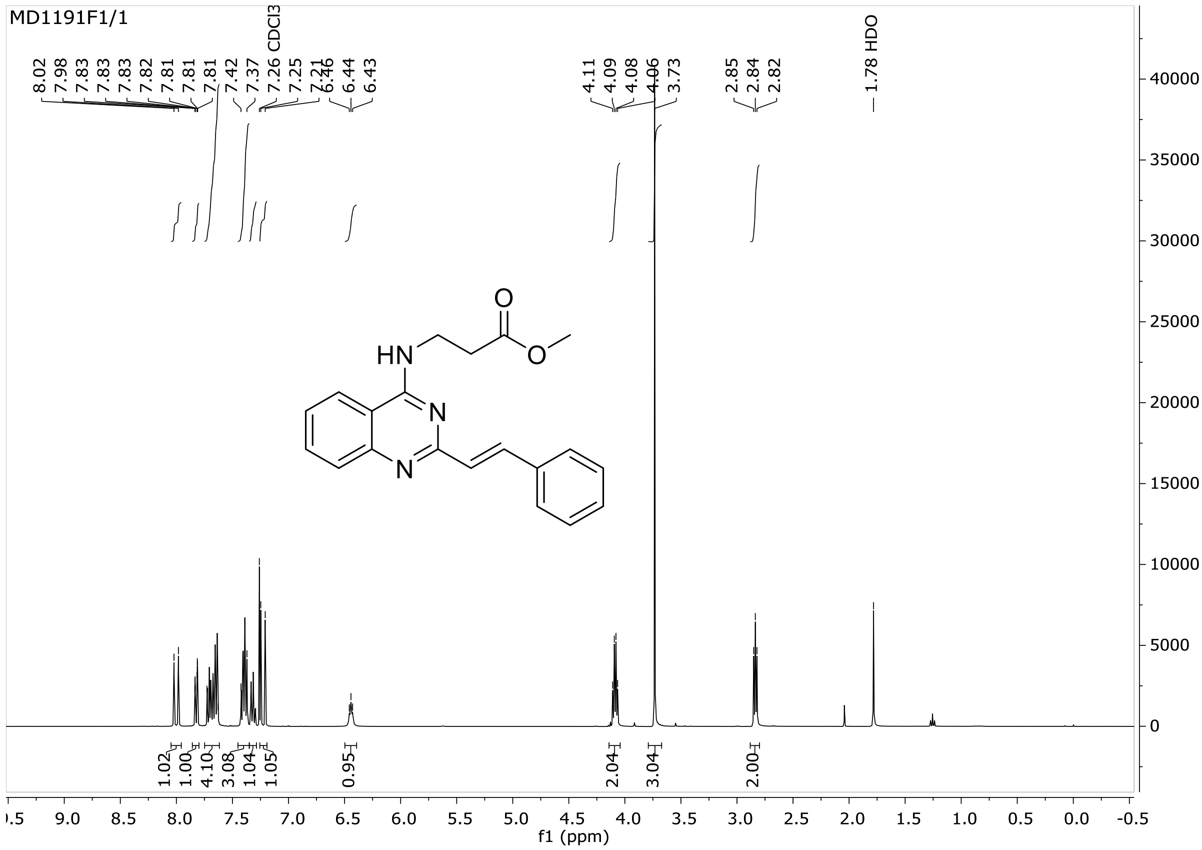

^{1}H-NMR spectrum of compound **3l**

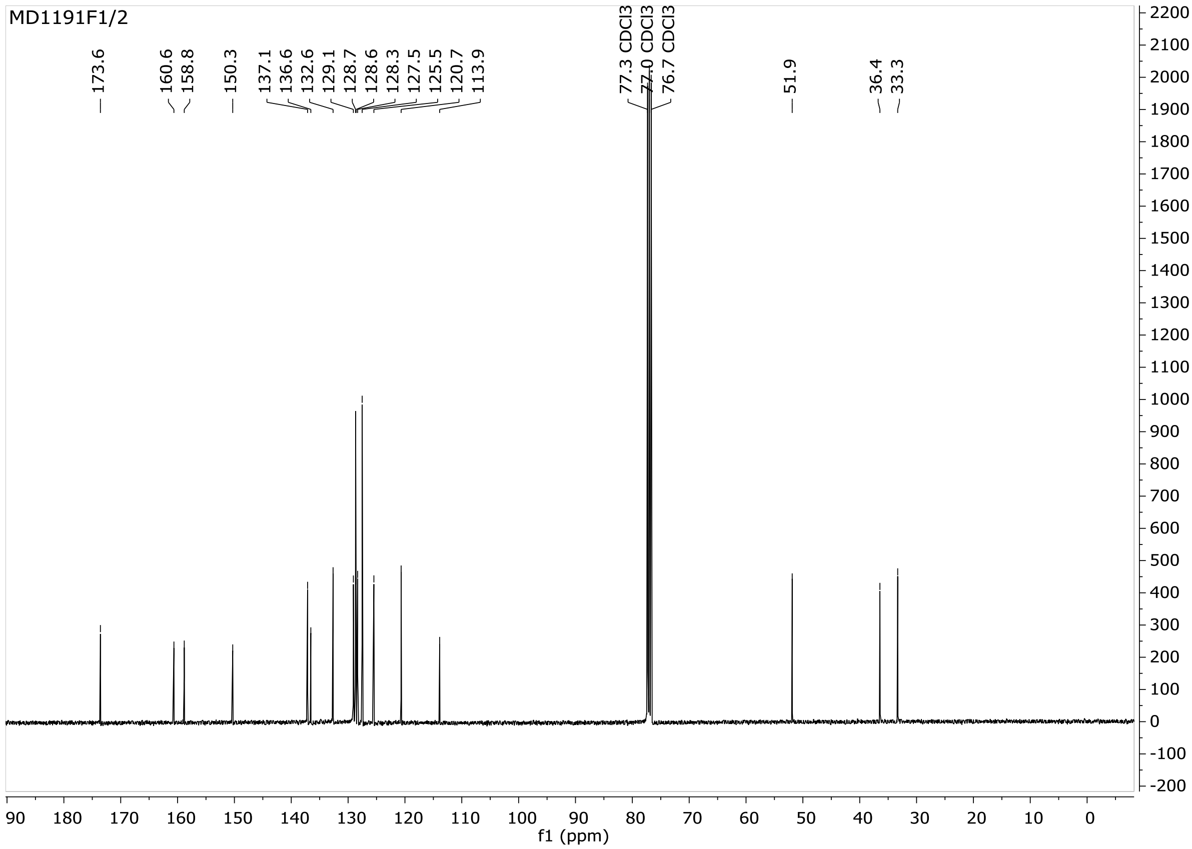

^{13}C-NMR spectrum of compound **3l**

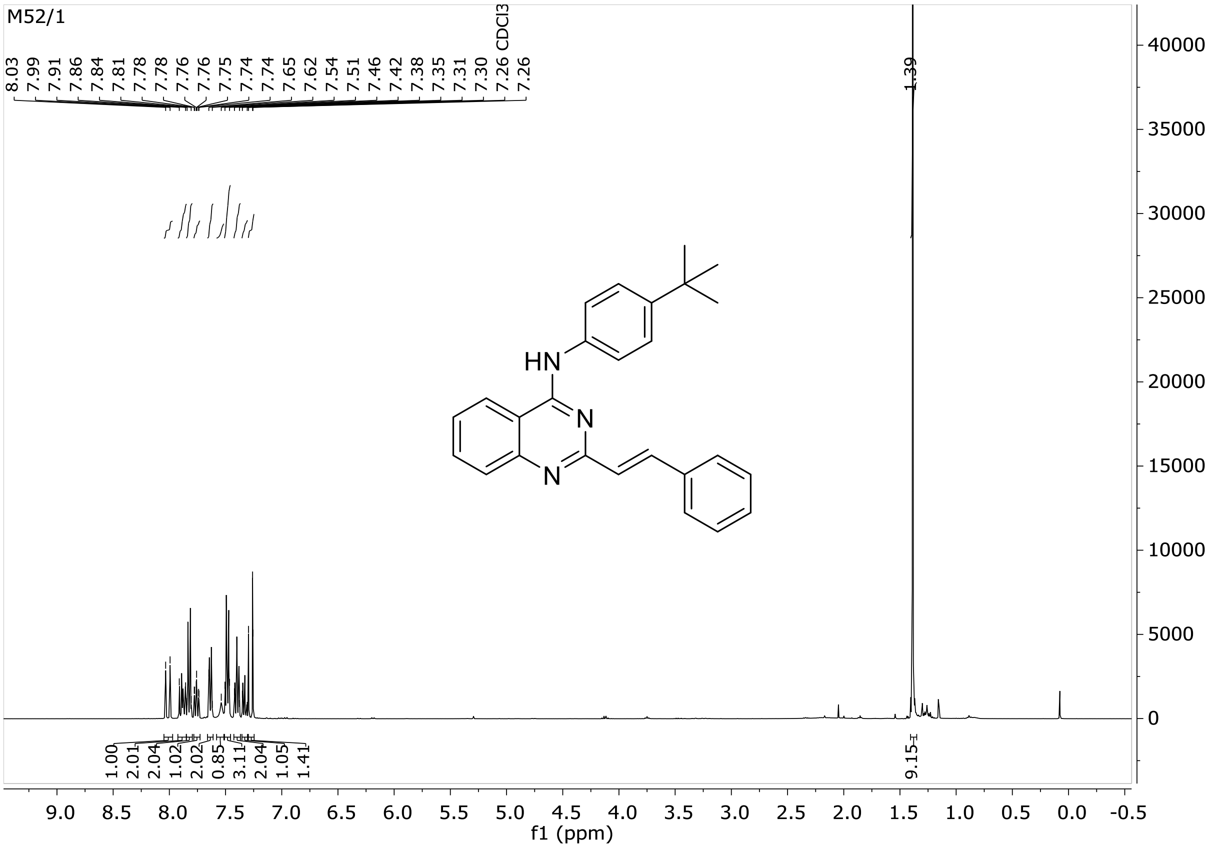

^{1}H-NMR spectrum of compound **3m**

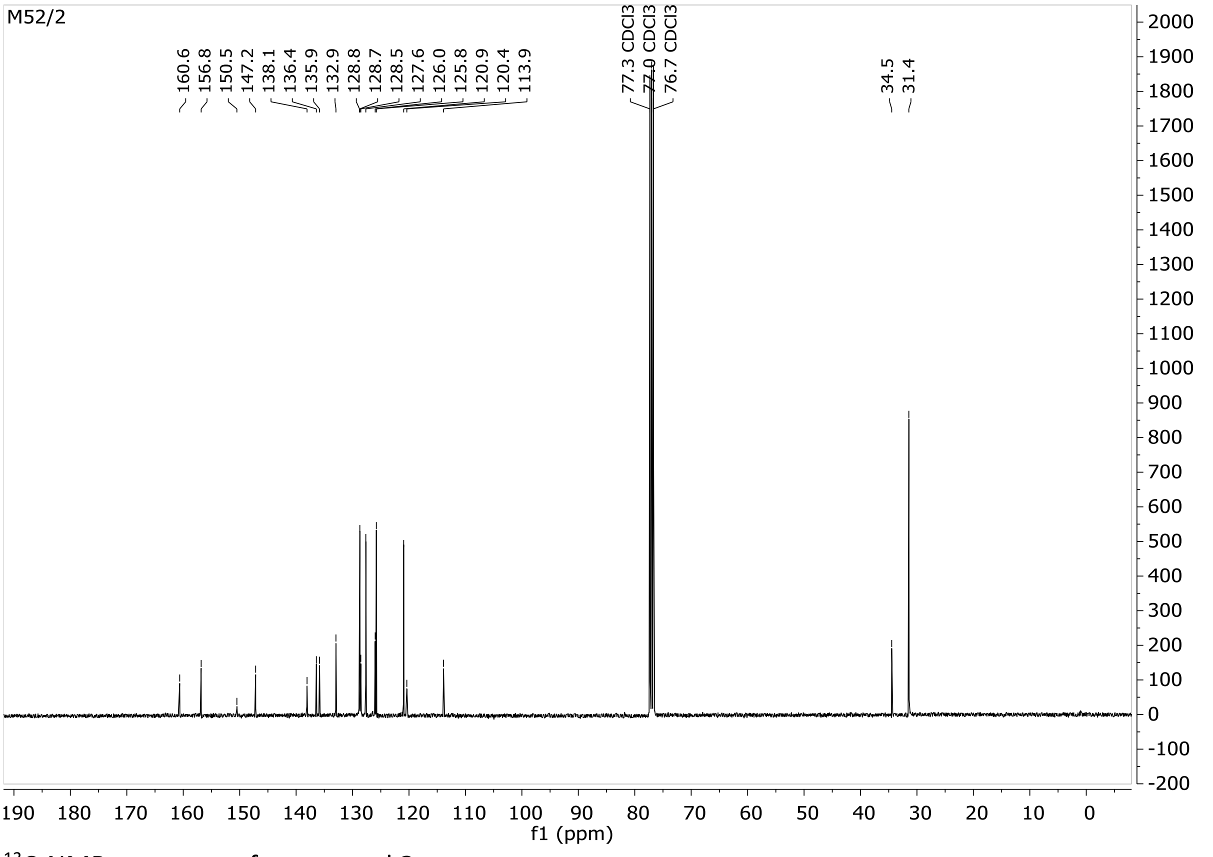

^{13}C-NMR spectrum of compound **3m**

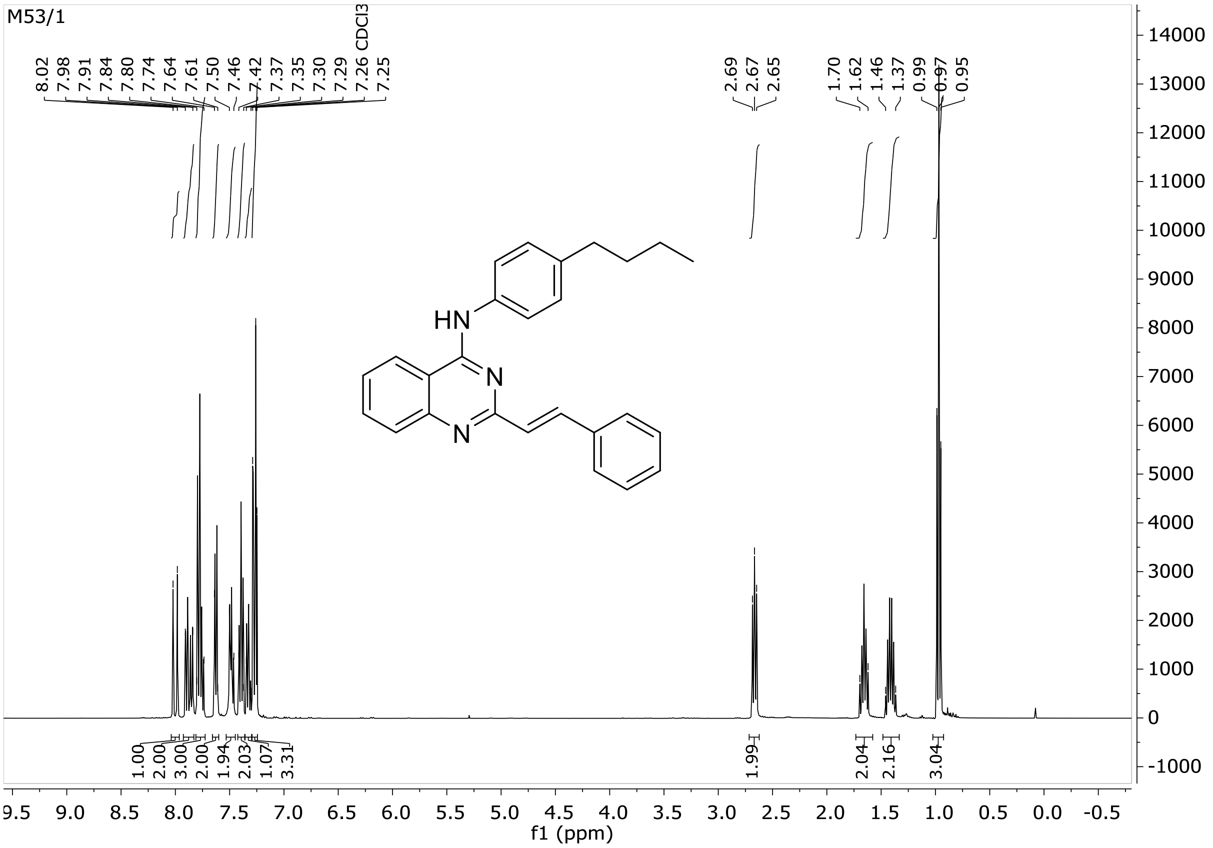

^{1}H-NMR spectrum of compound **3n**

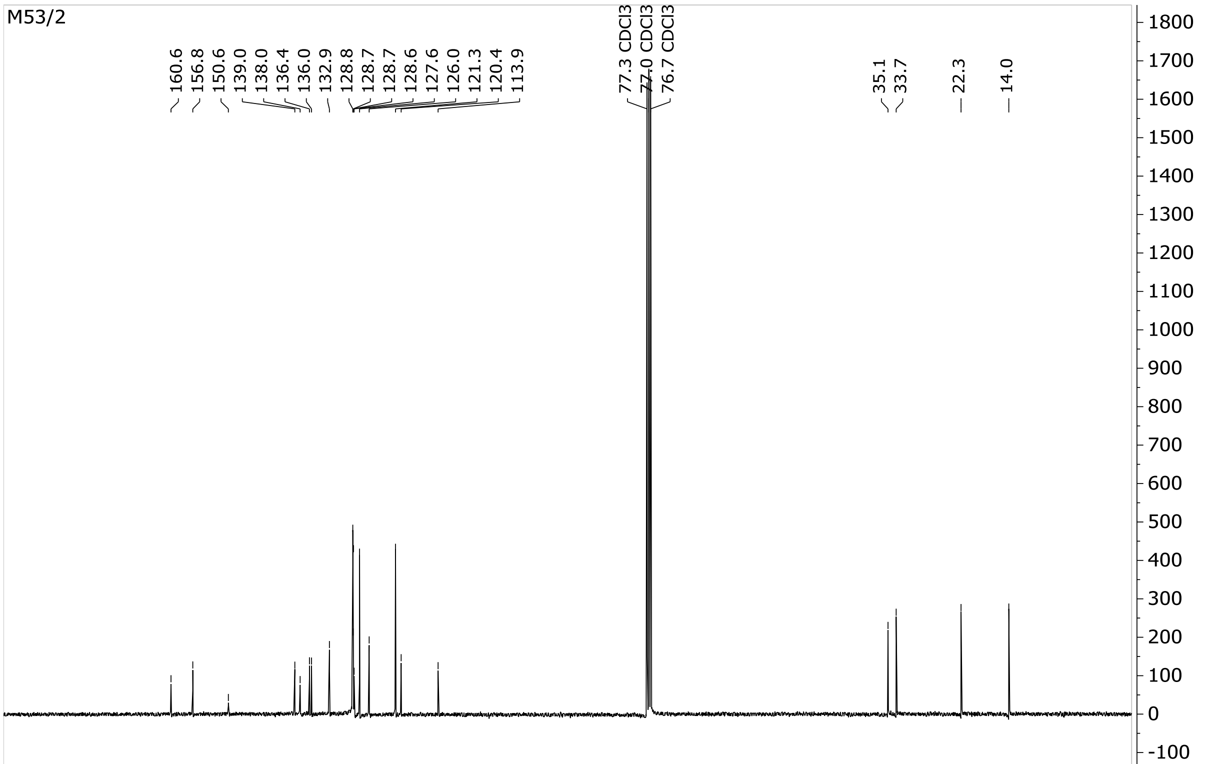

^{13}C-NMR spectrum of compound **3n**

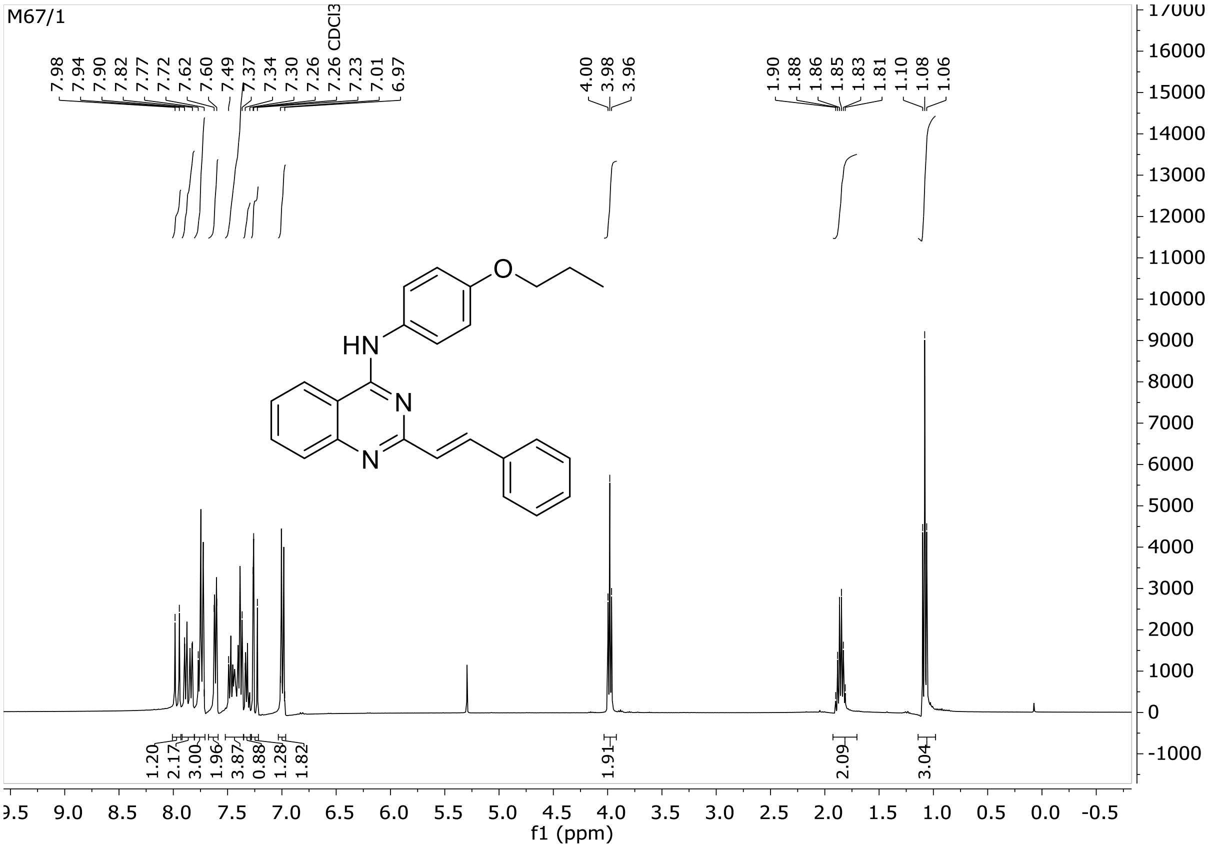

^{1}H-NMR spectrum of compound **3o**

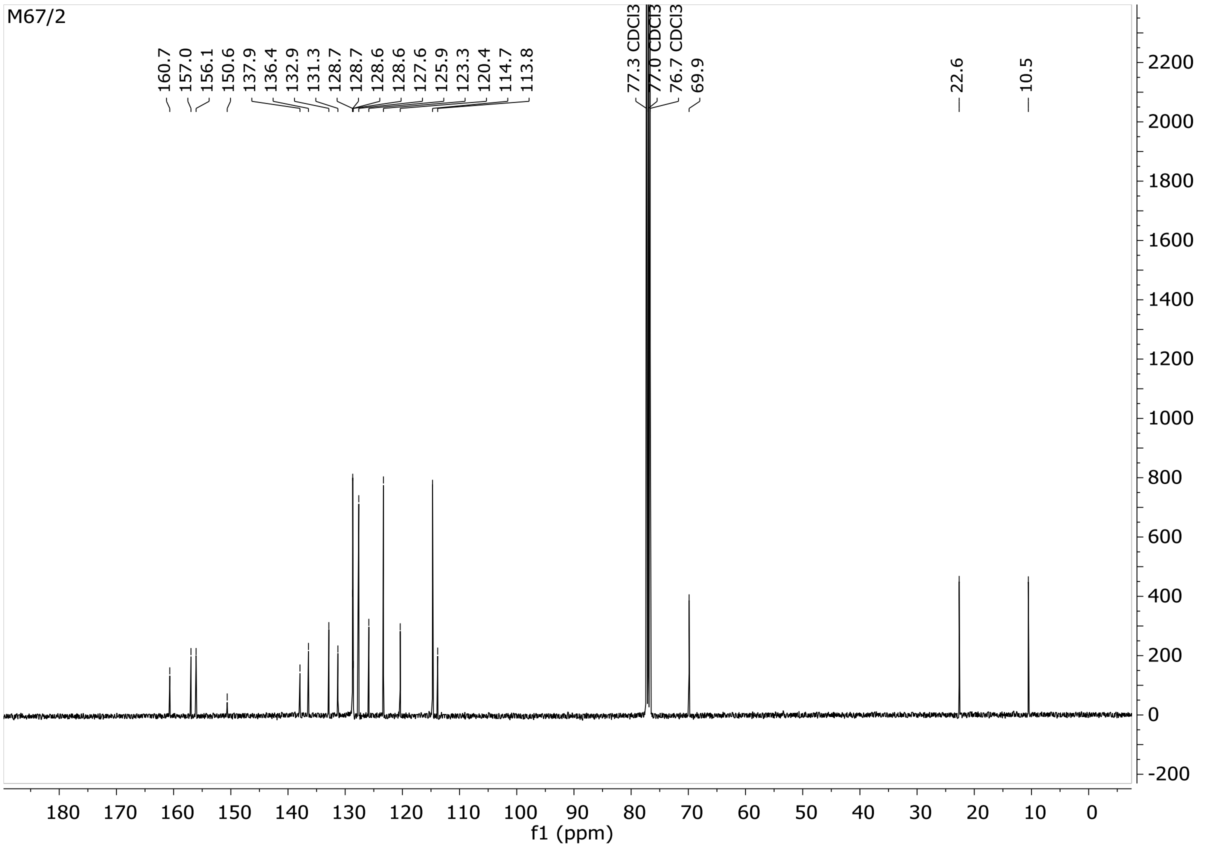

^{13}C-NMR spectrum of compound **3o**

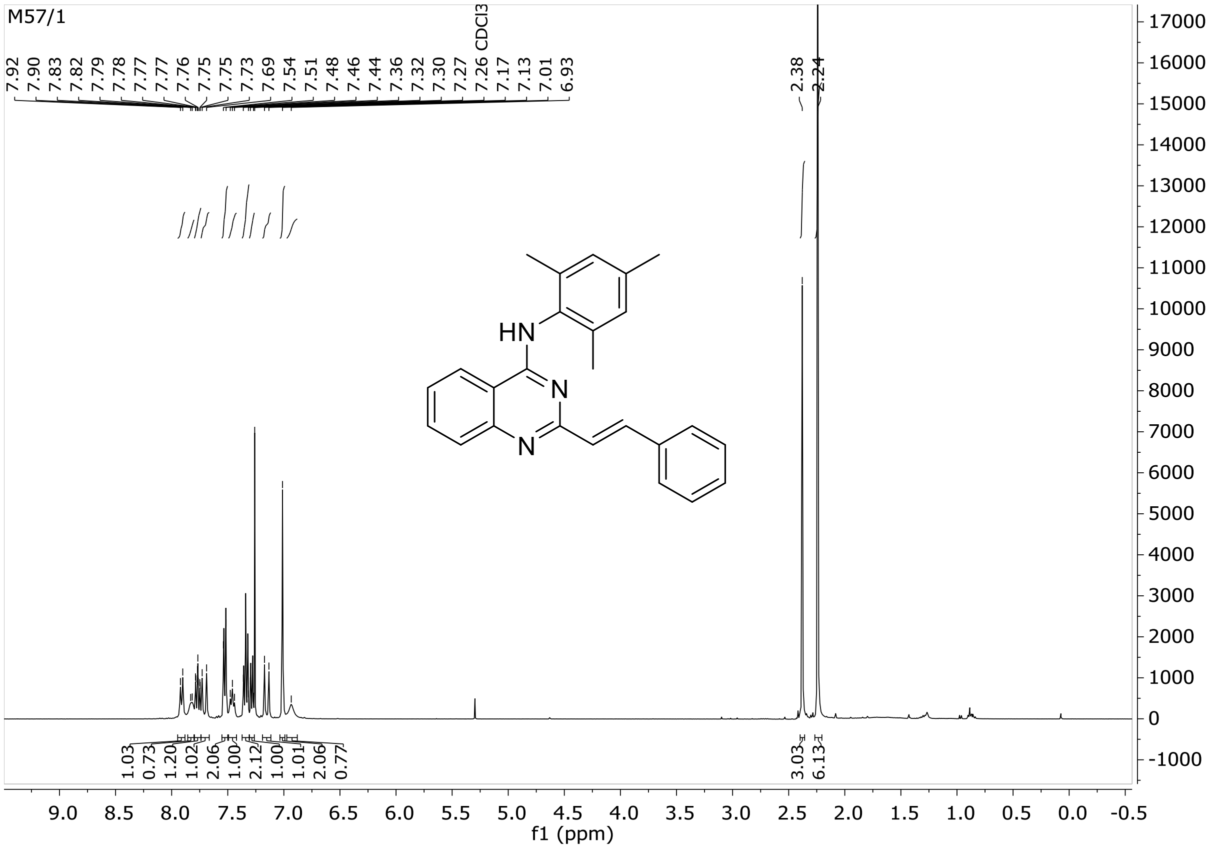

^{1}H-NMR spectrum of compound **3p**

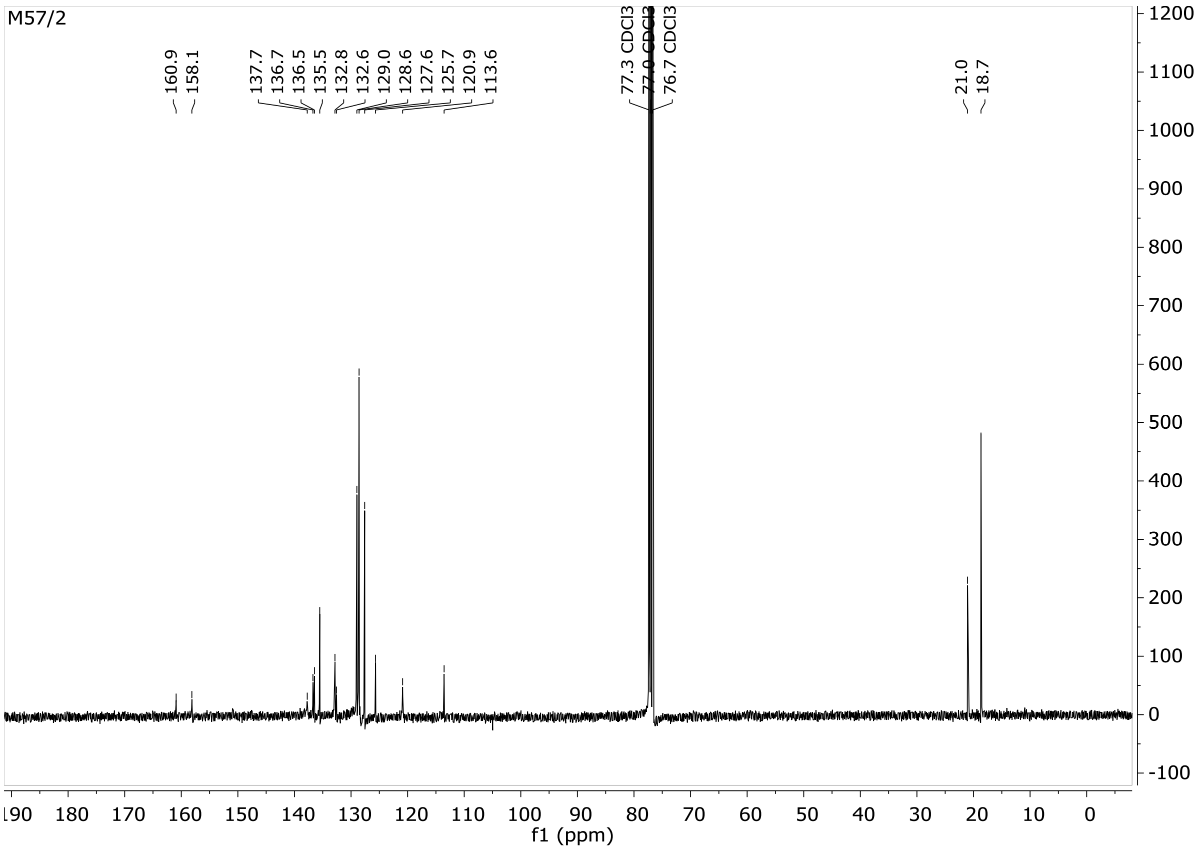

^{13}C-NMR spectrum of compound **3p**

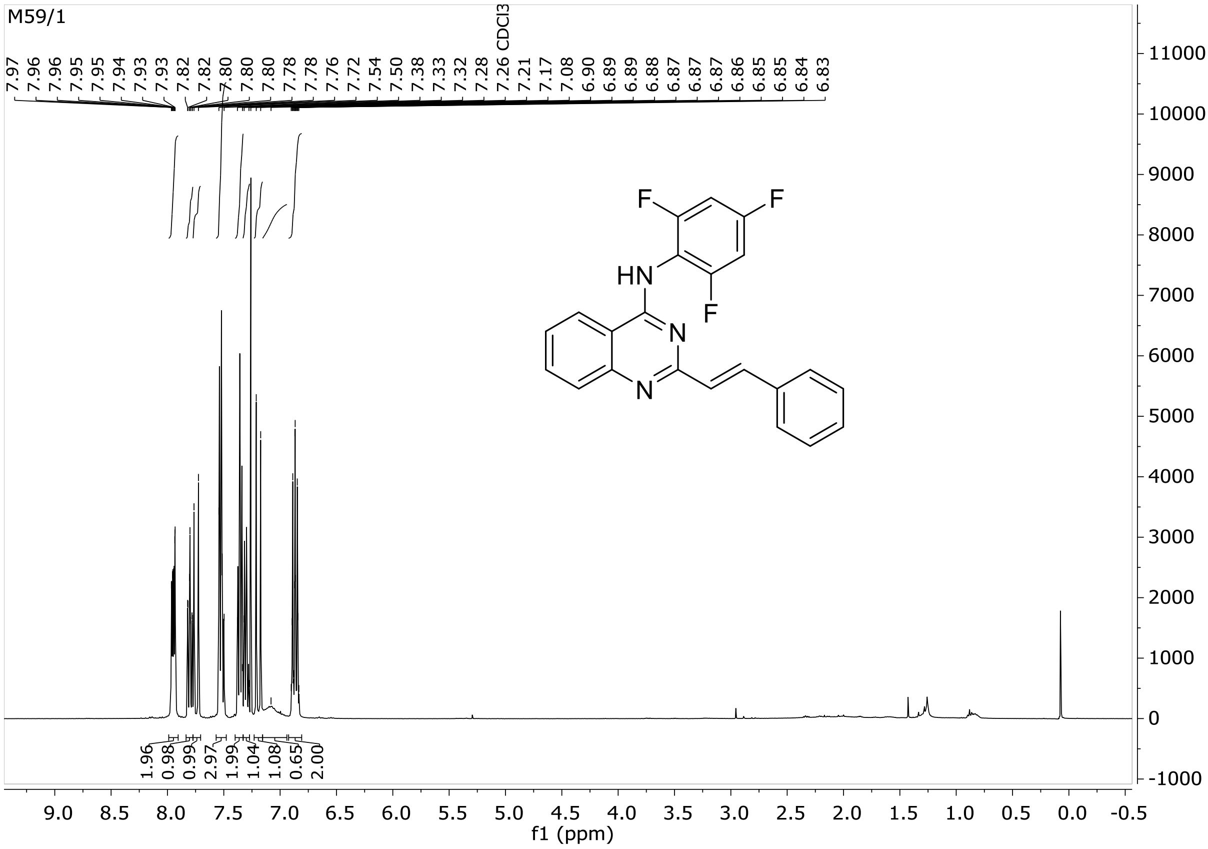

^{1}H-NMR spectrum of compound **3q**

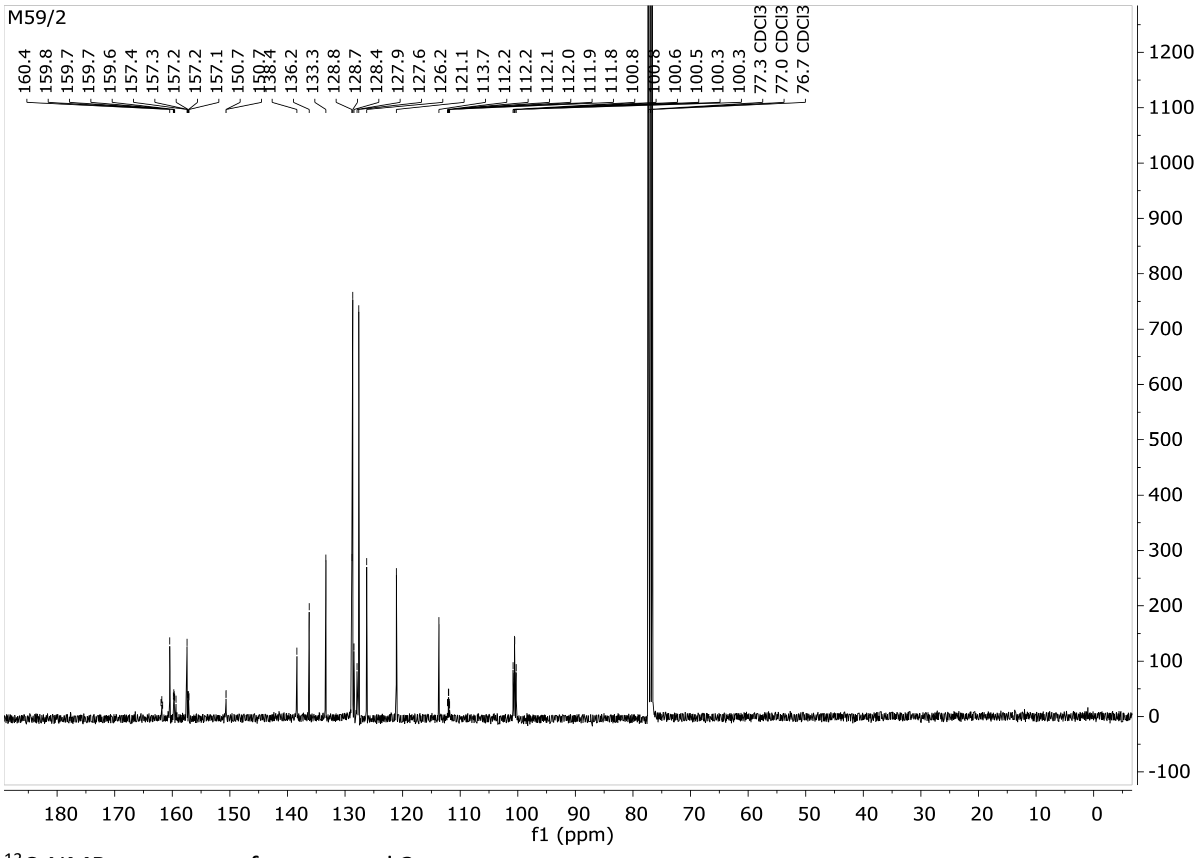

^{13}C-NMR spectrum of compound **3q**

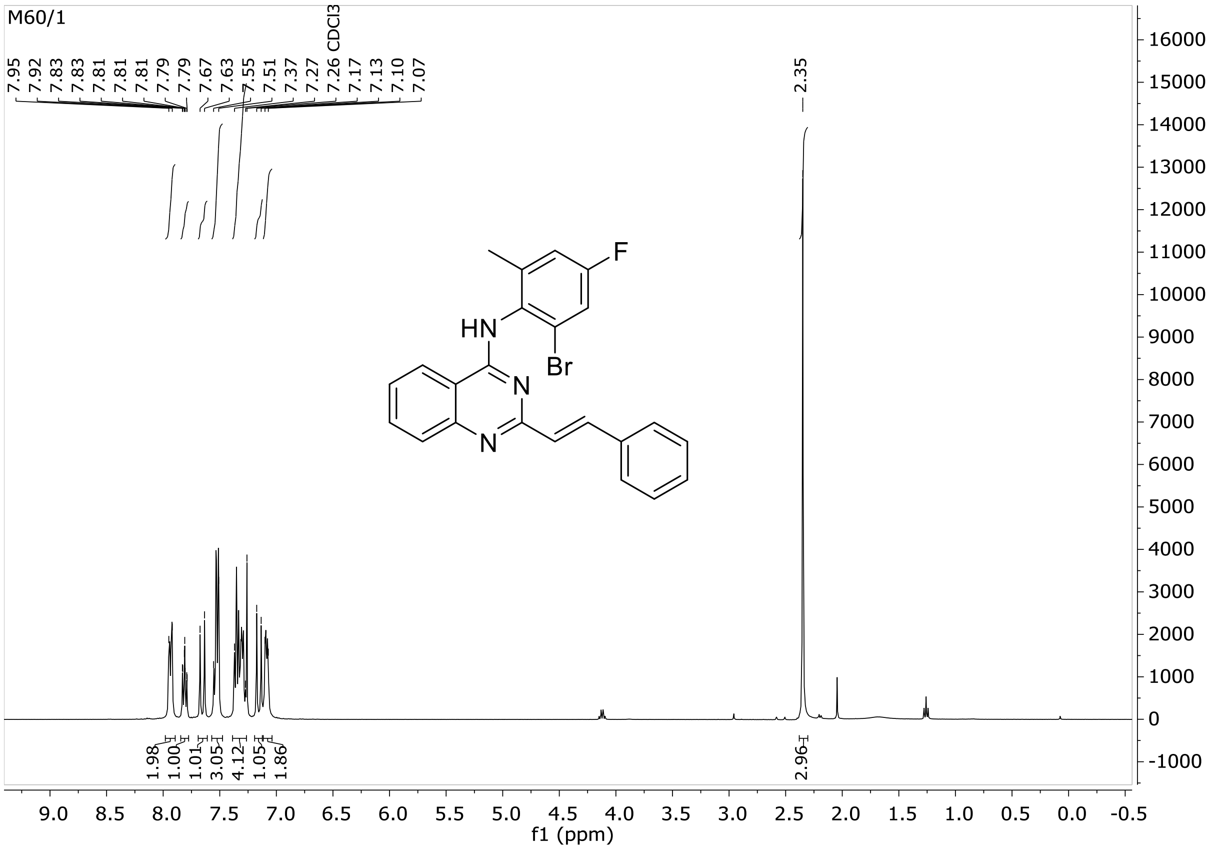

^{1}H-NMR spectrum of compound **3r**

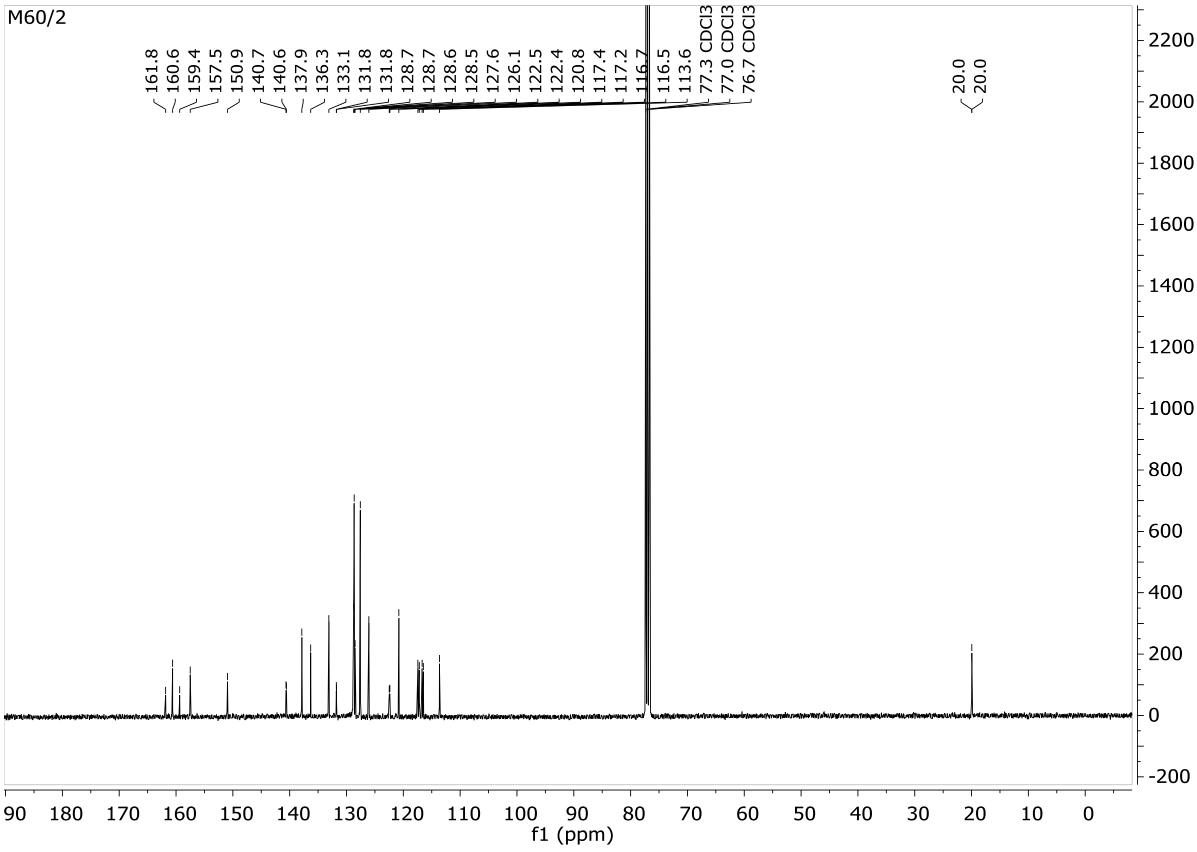

^{13}C-NMR spectrum of compound **3r**

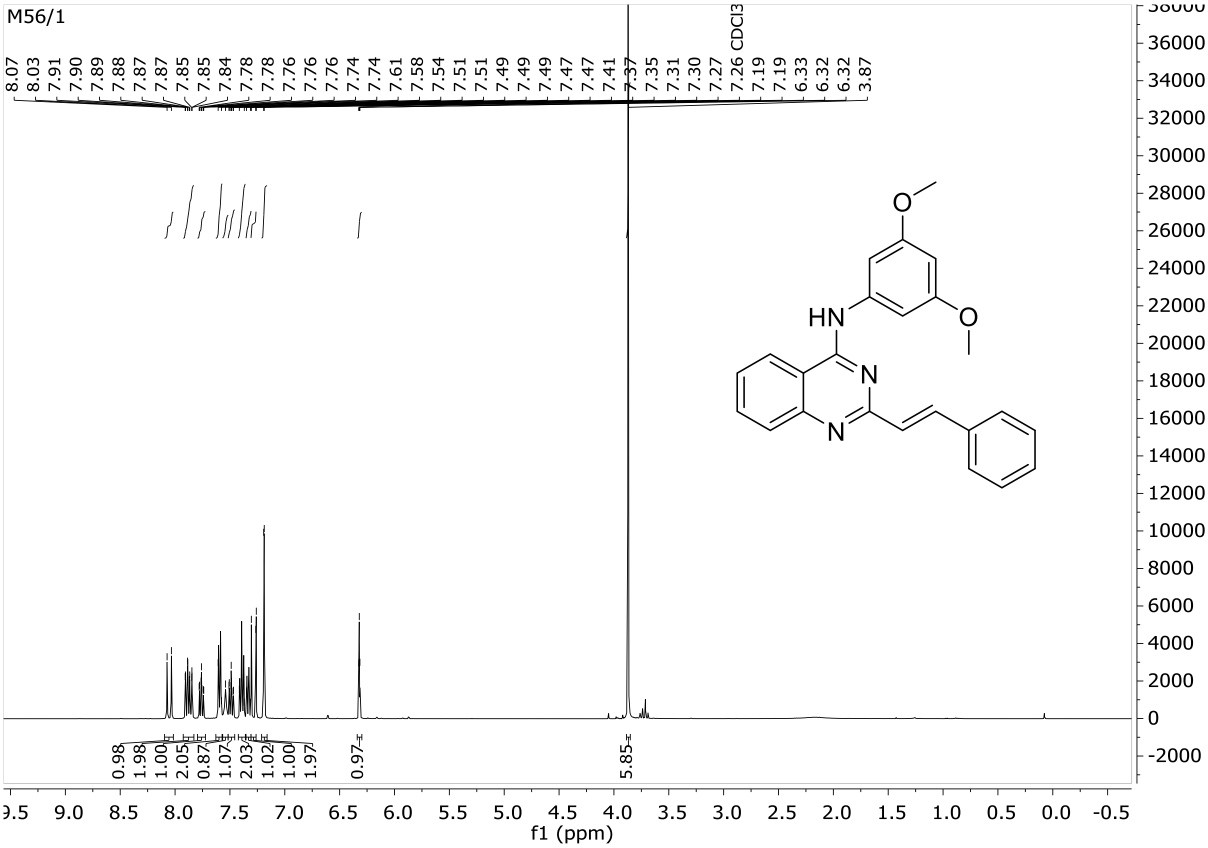

^{1}H-NMR spectrum of compound **3s**

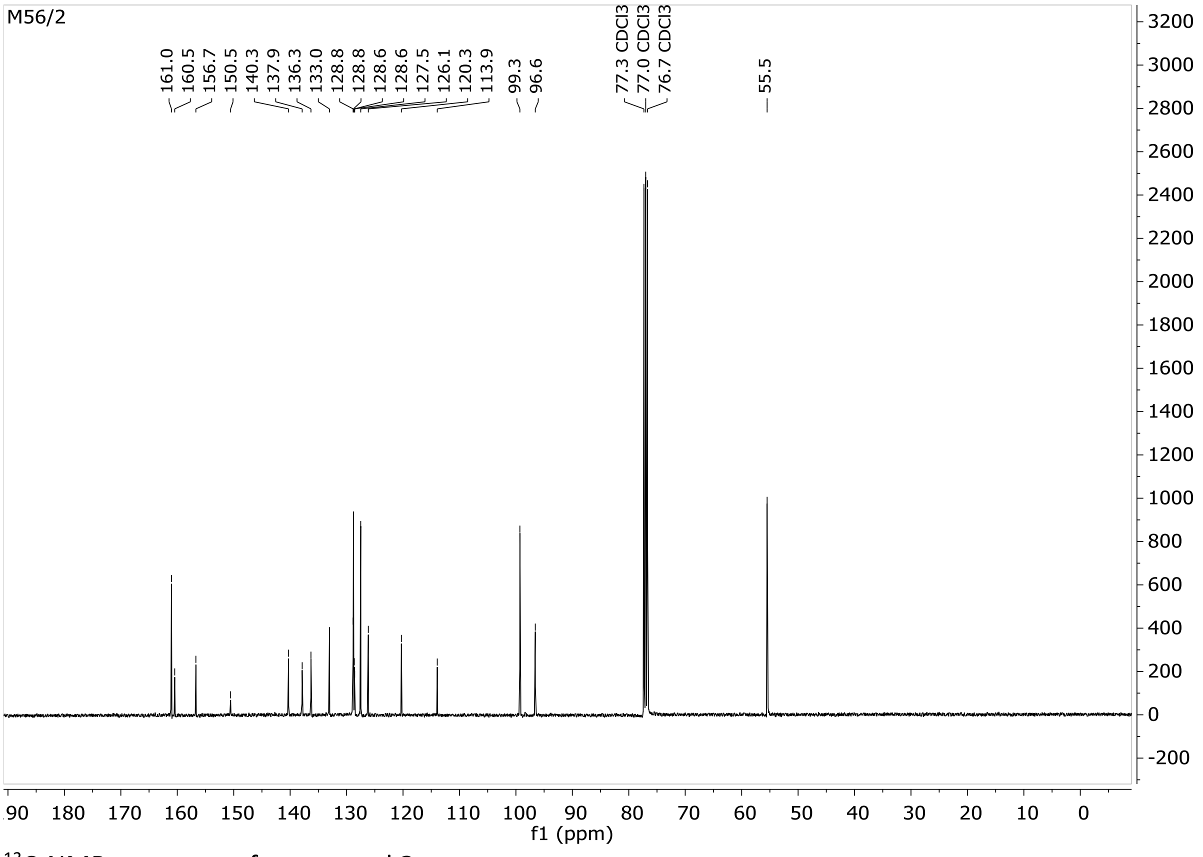

^{13}C-NMR spectrum of compound **3s**

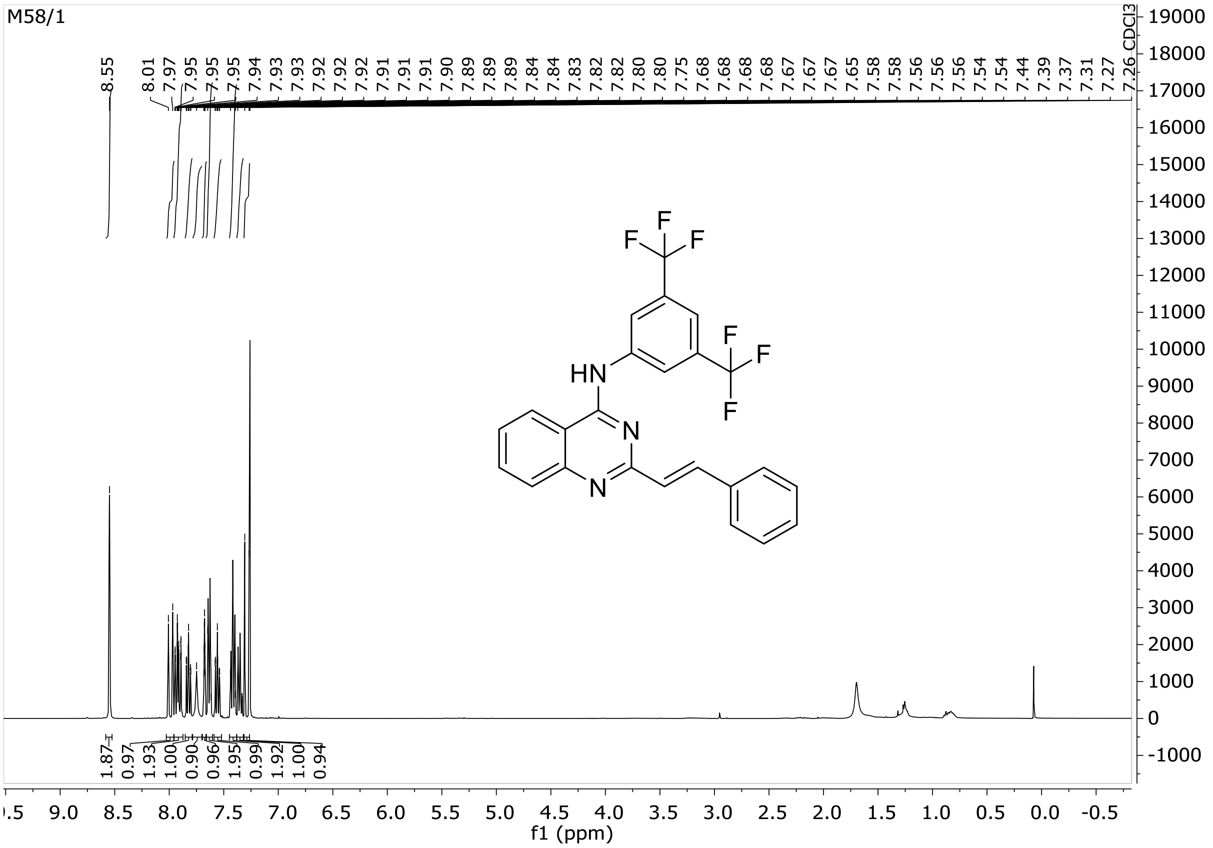

^{1}H-NMR spectrum of compound **3t**

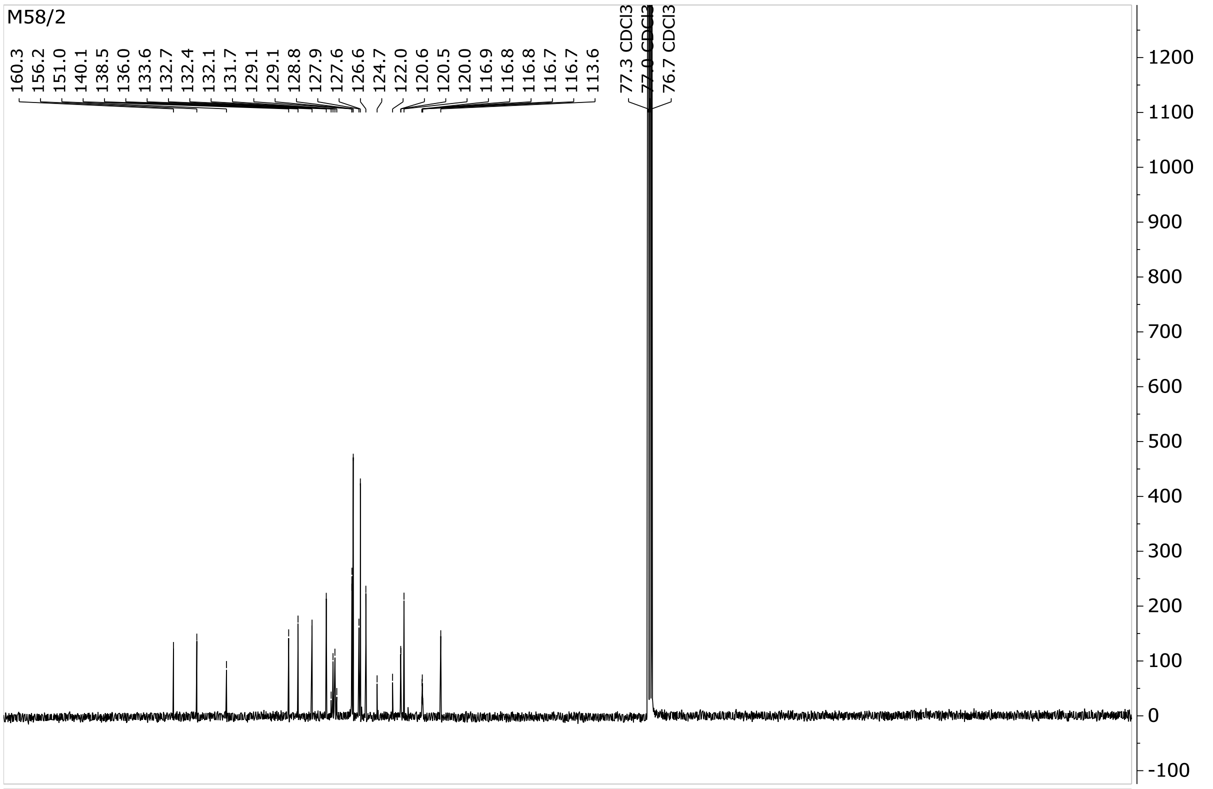

^{13}C-NMR spectrum of compound **3t**

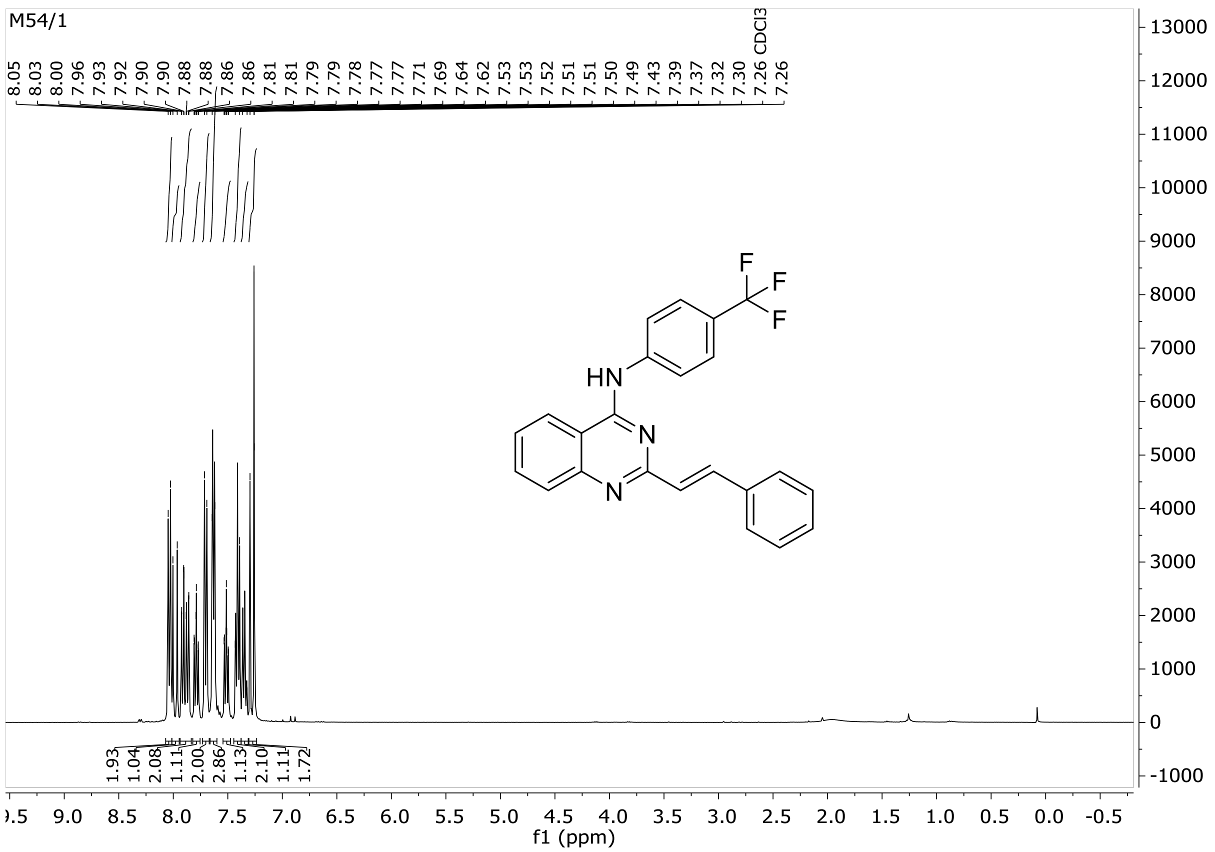

^{1}H-NMR spectrum of compound **3u**

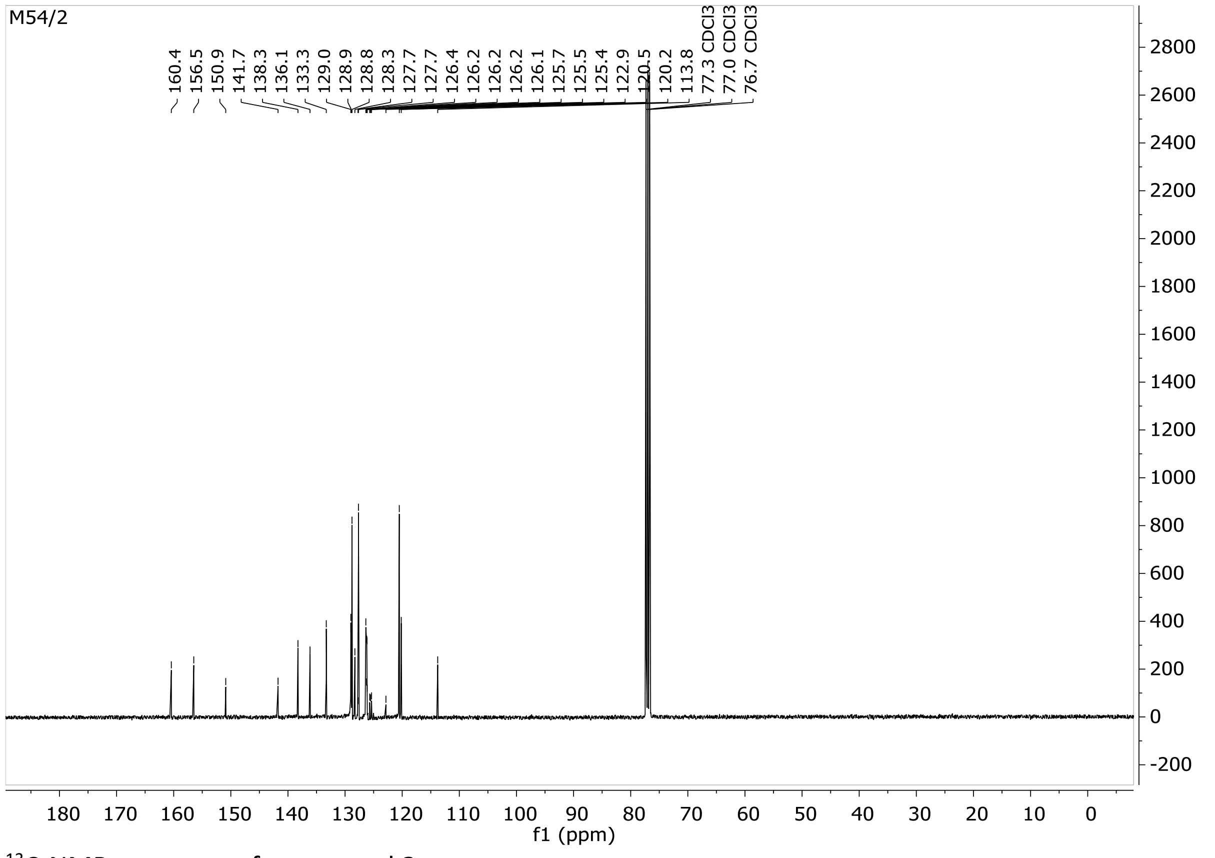

^{13}C-NMR spectrum of compound **3u**

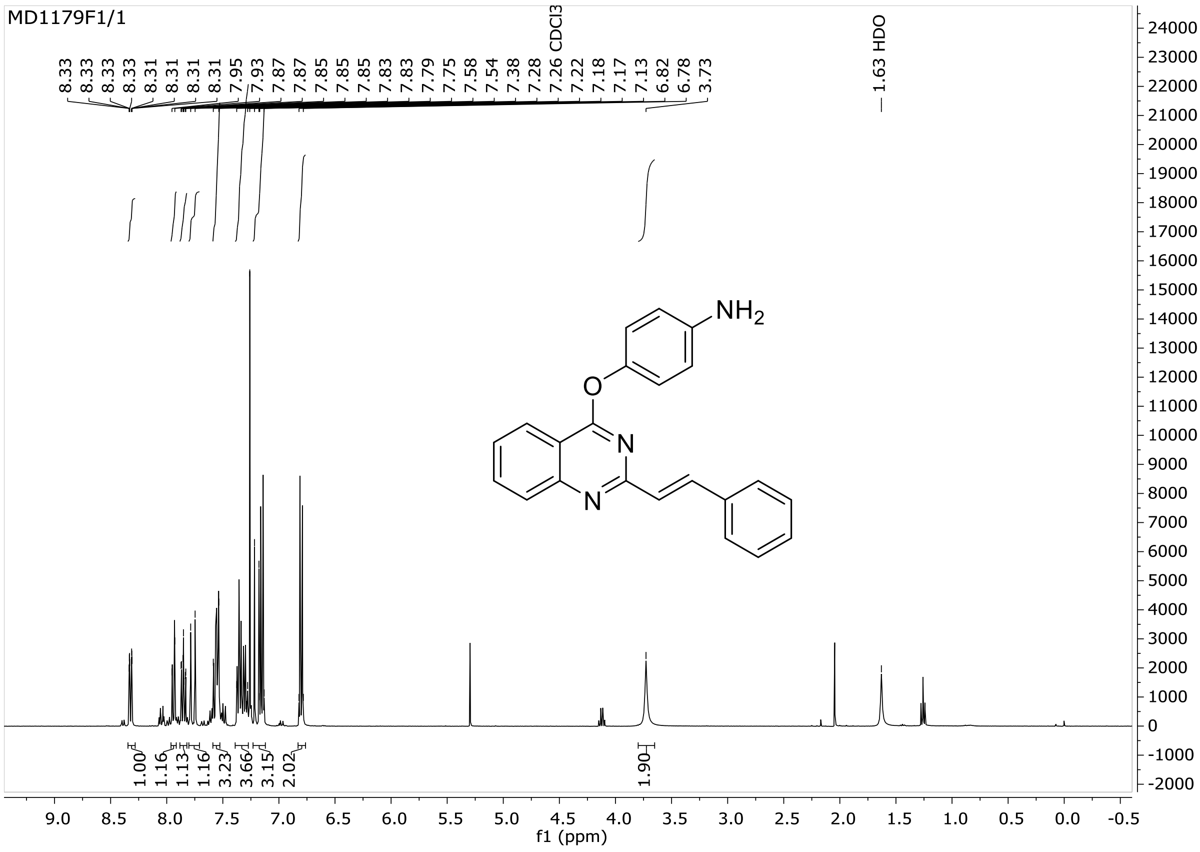

^{1}H-NMR spectrum of compound **3v**

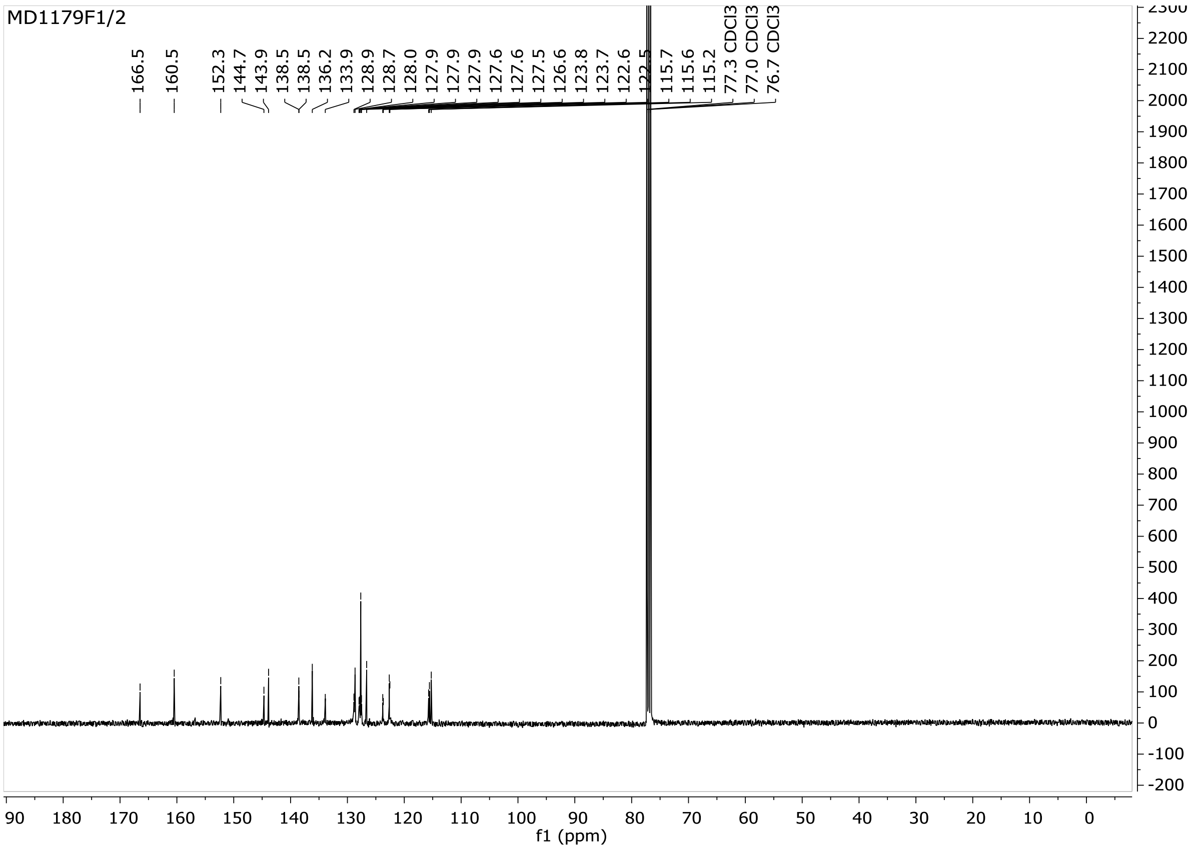

^{13}C-NMR spectrum of compound **3v**

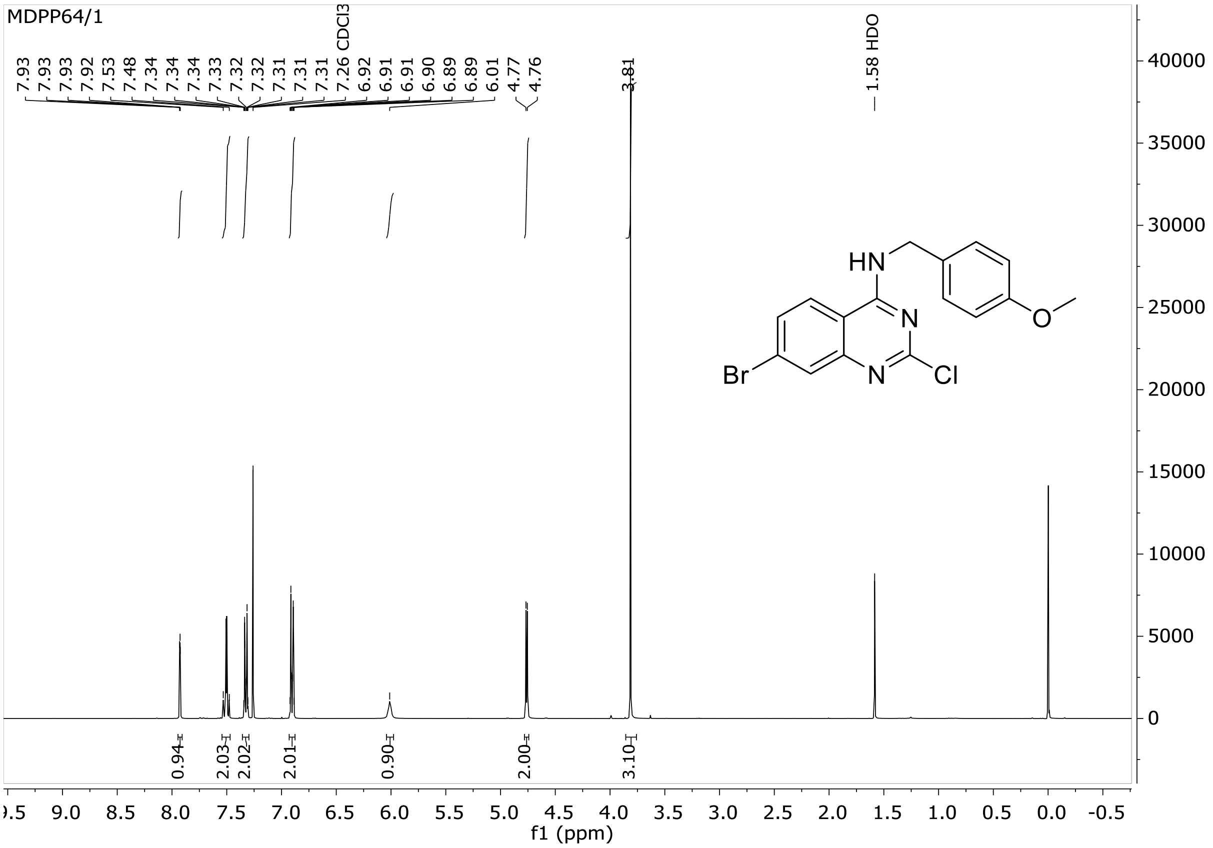

^{1}H-NMR spectrum of compound **5a**

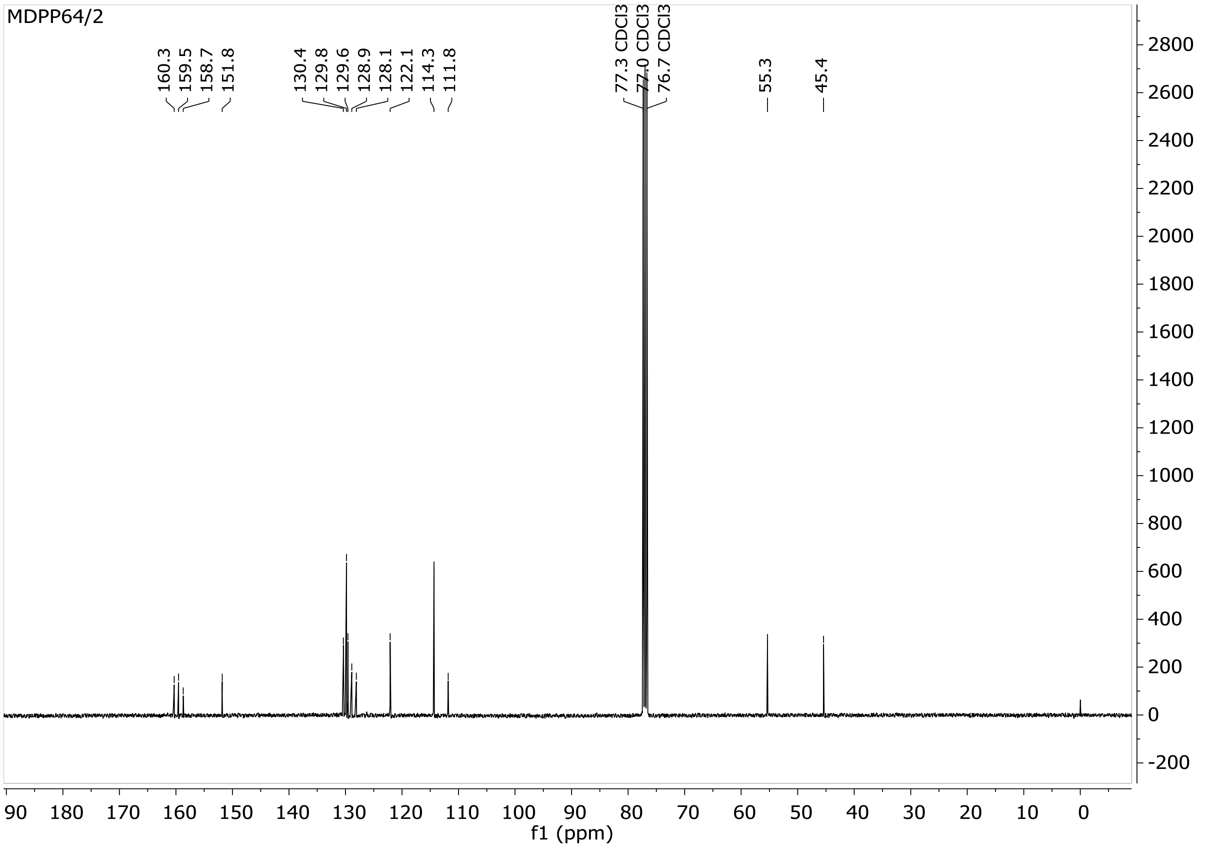

^{13}C-NMR spectrum of compound **5a**

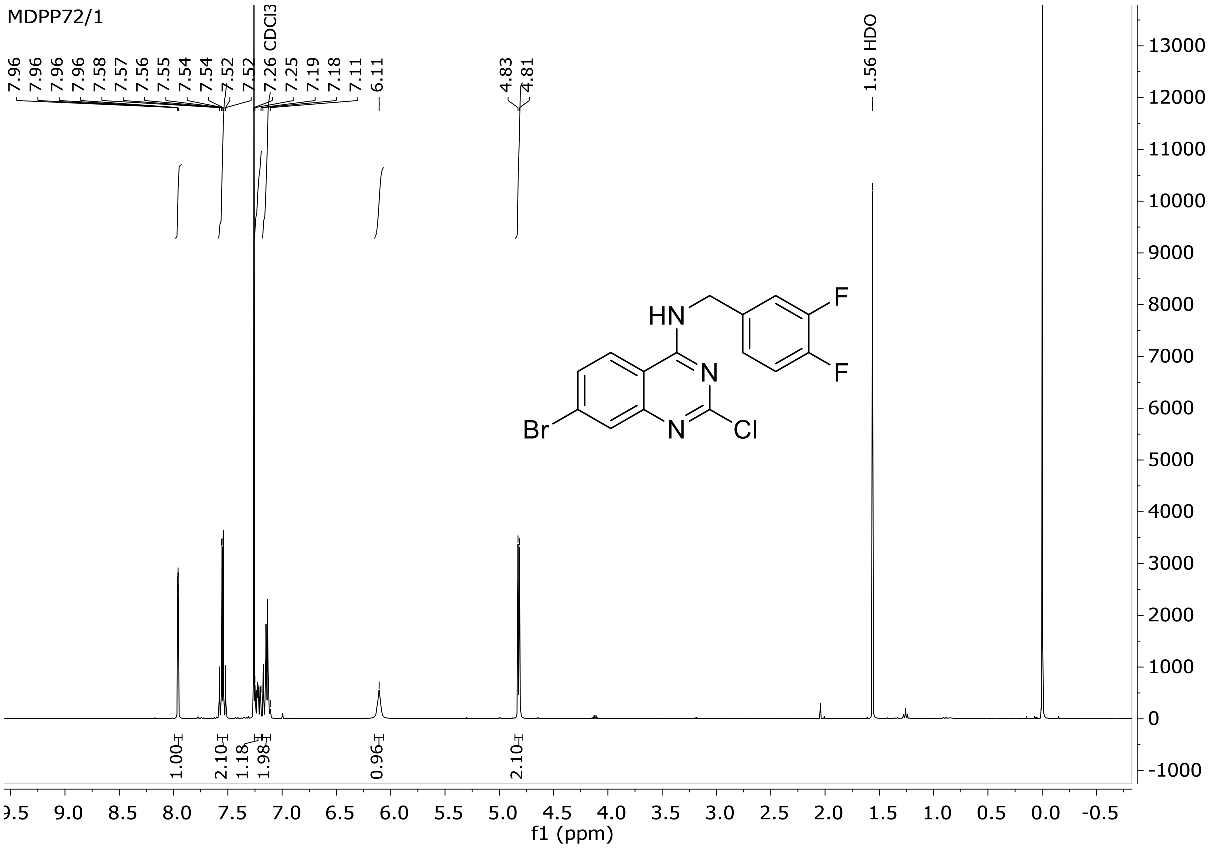

^{1}H-NMR spectrum of compound **5b**

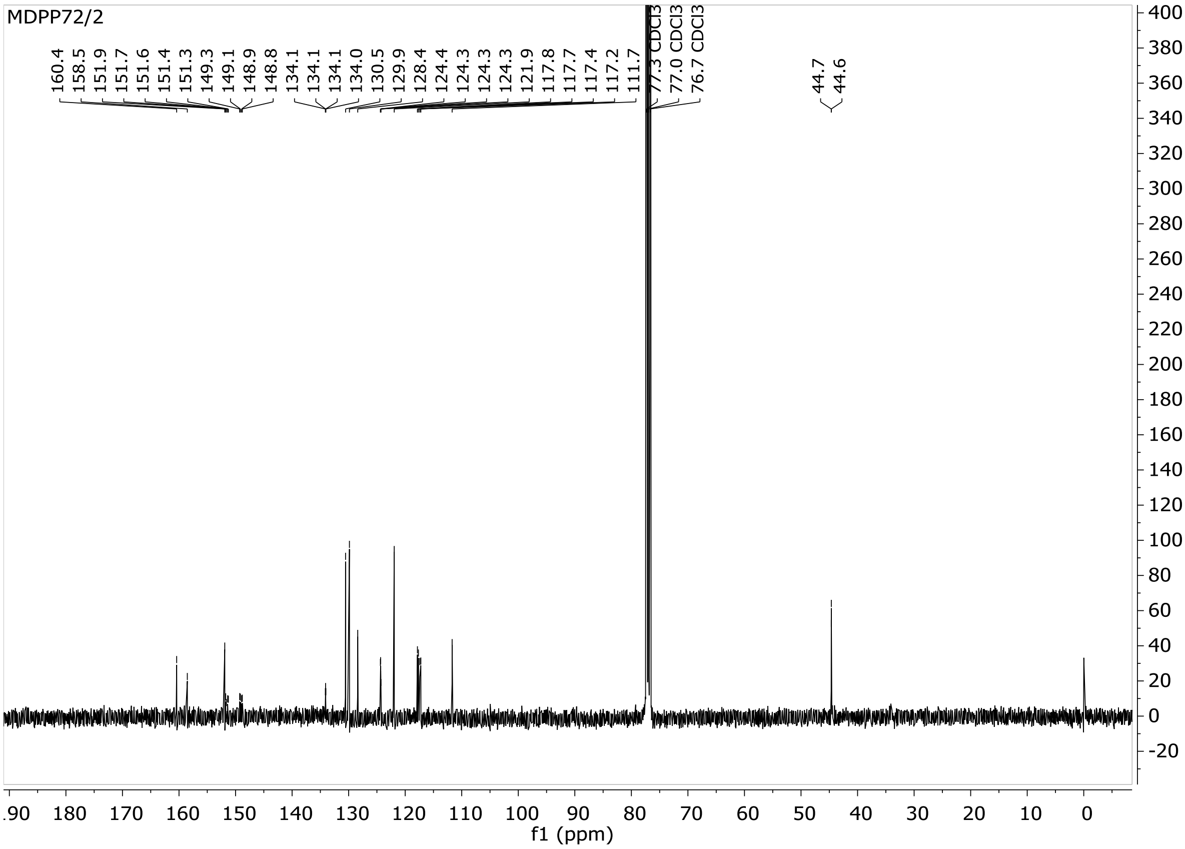

^{13}C-NMR spectrum of compound **5b**

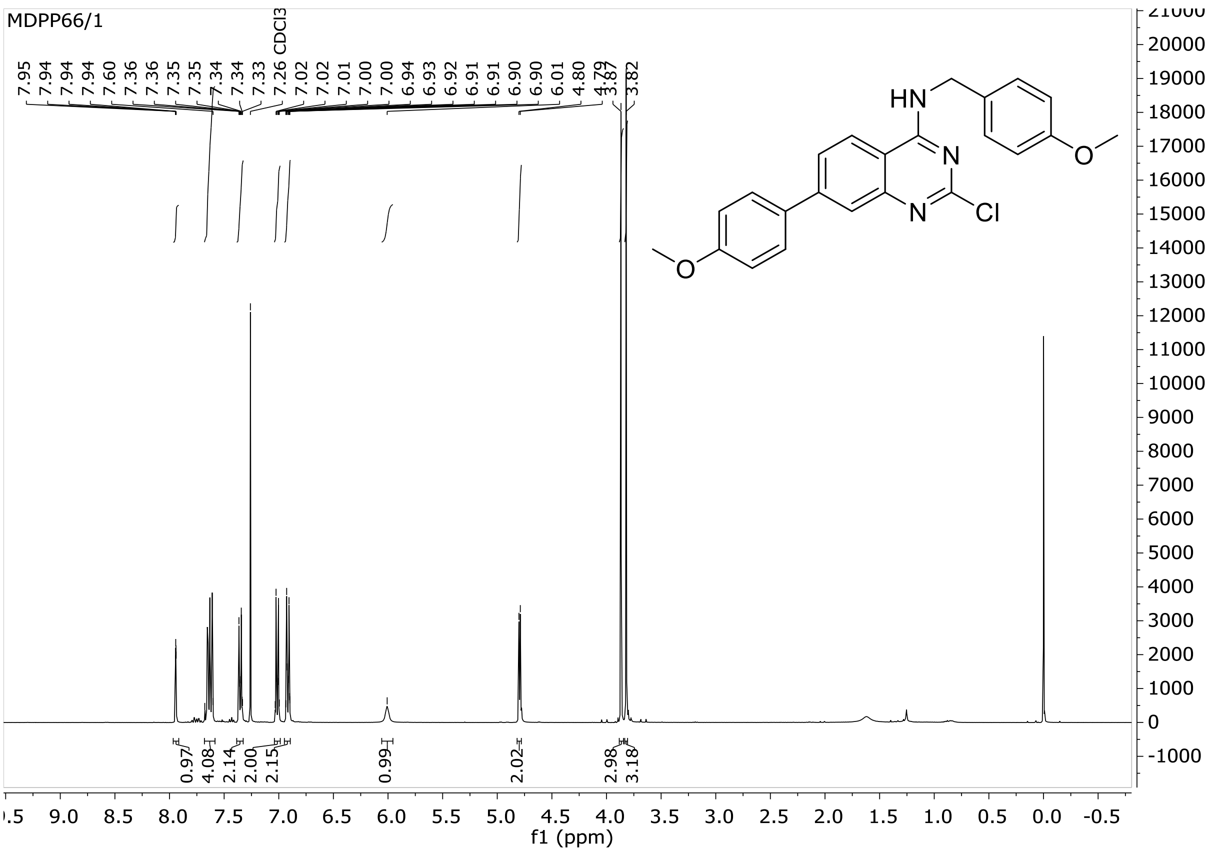

^{1}H-NMR spectrum of compound **6a**

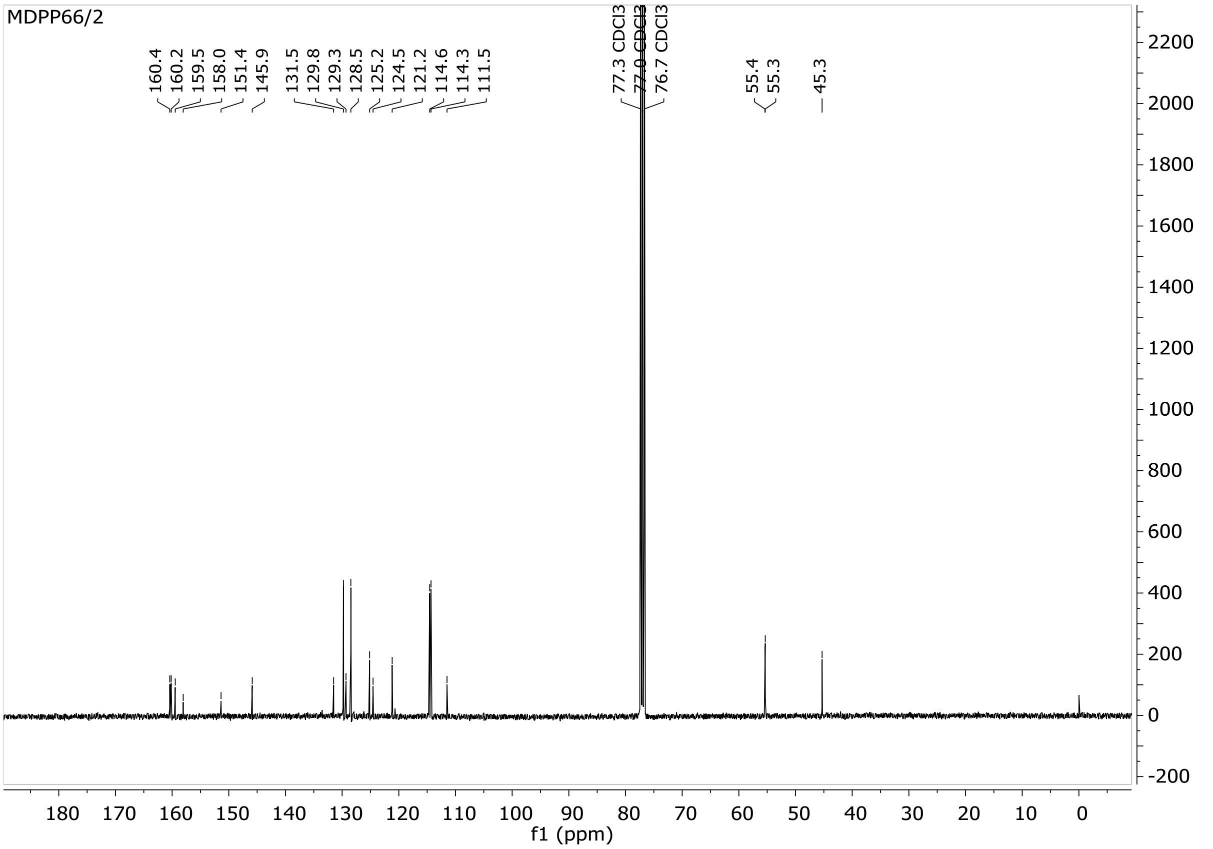

^{13}C-NMR spectrum of compound **6a**

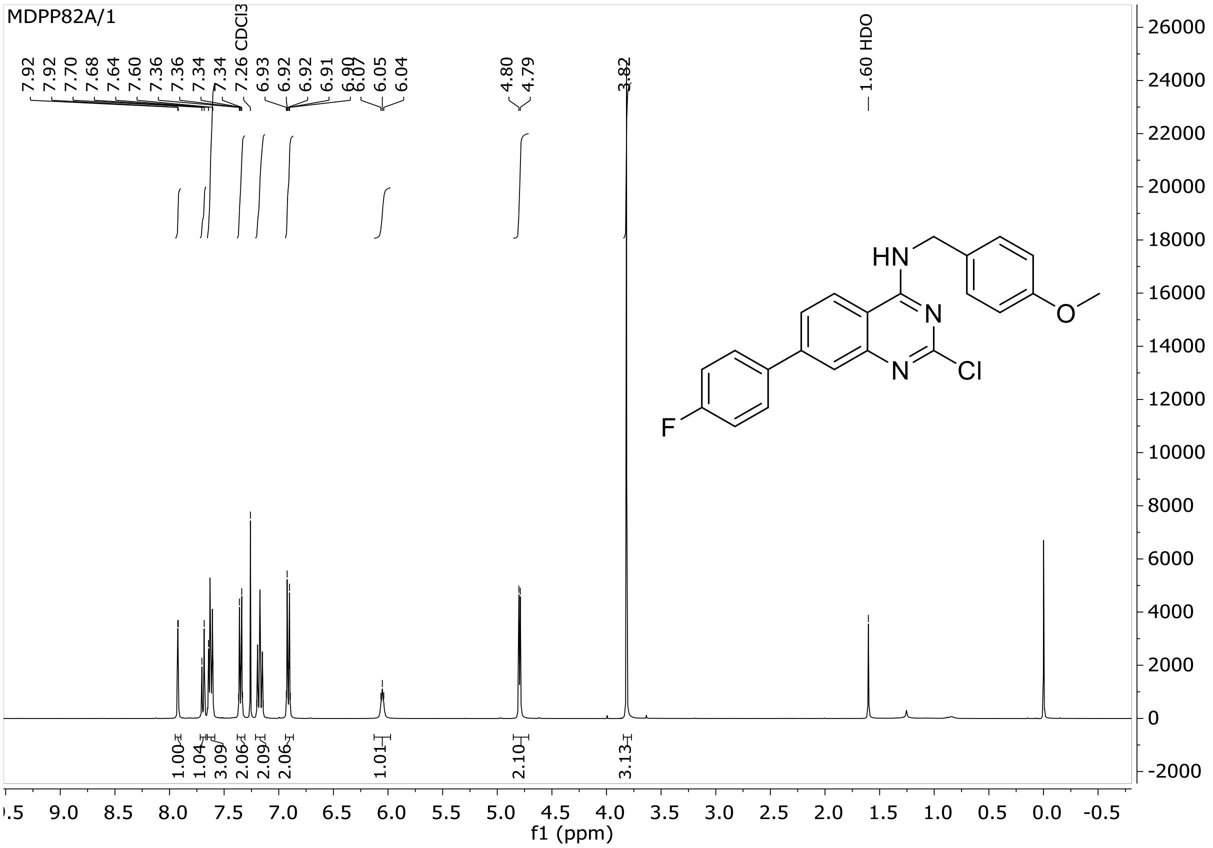

^{1}H-NMR spectrum of compound **6b**

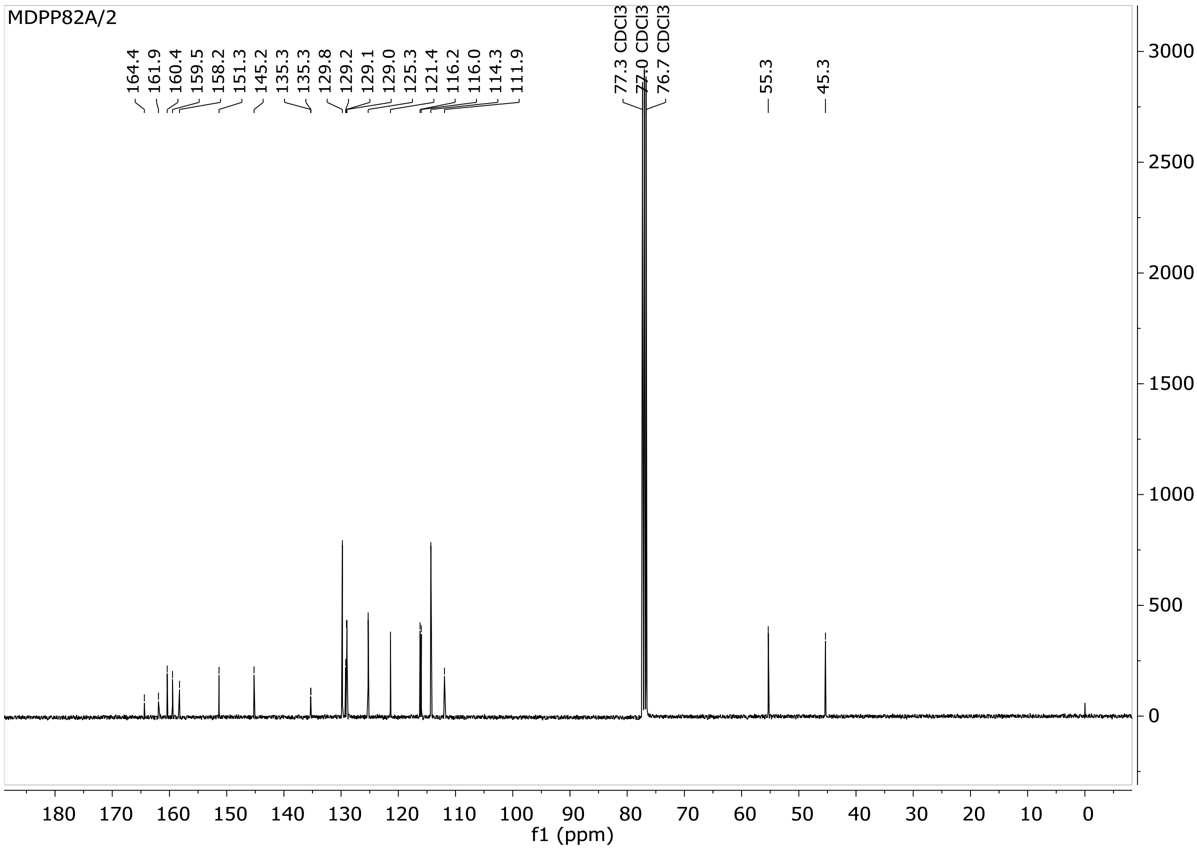

^{13}C-NMR spectrum of compound **6b**

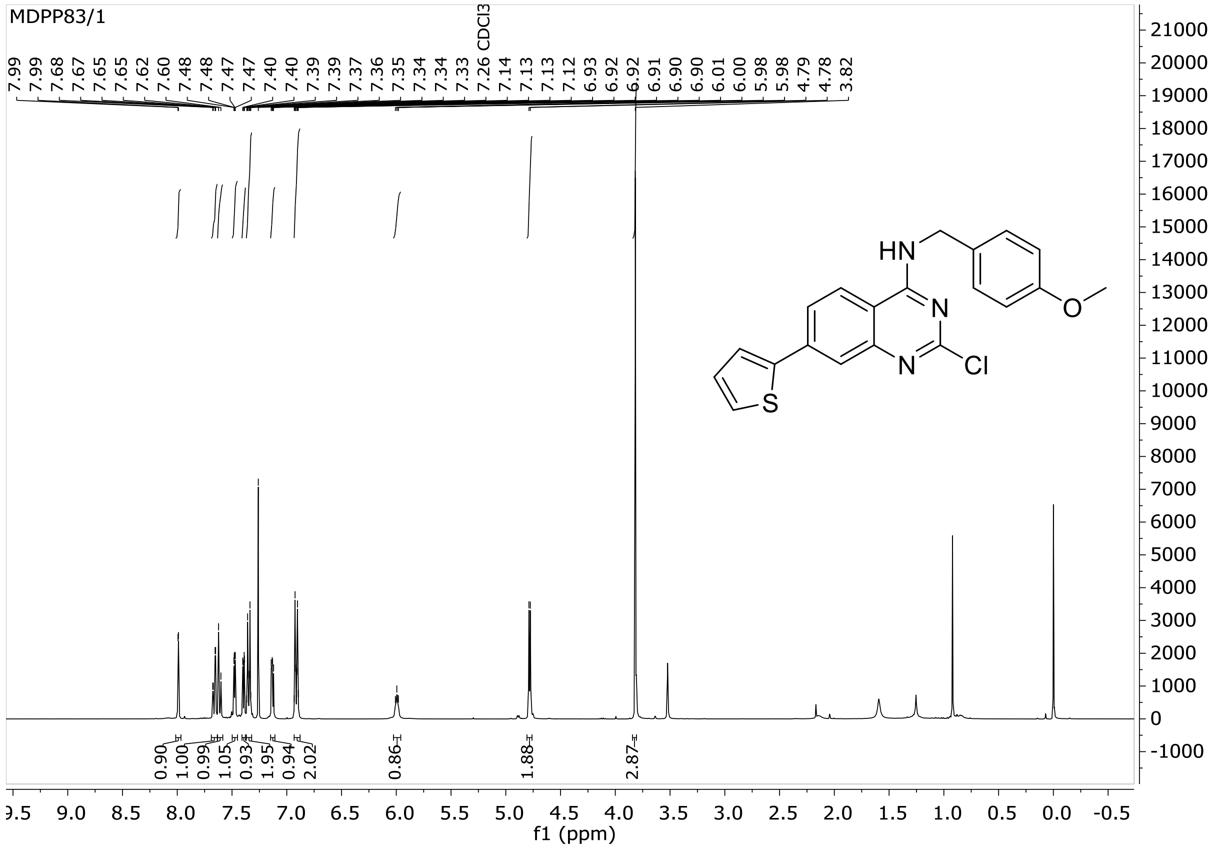

^{1}H-NMR spectrum of compound **6c**

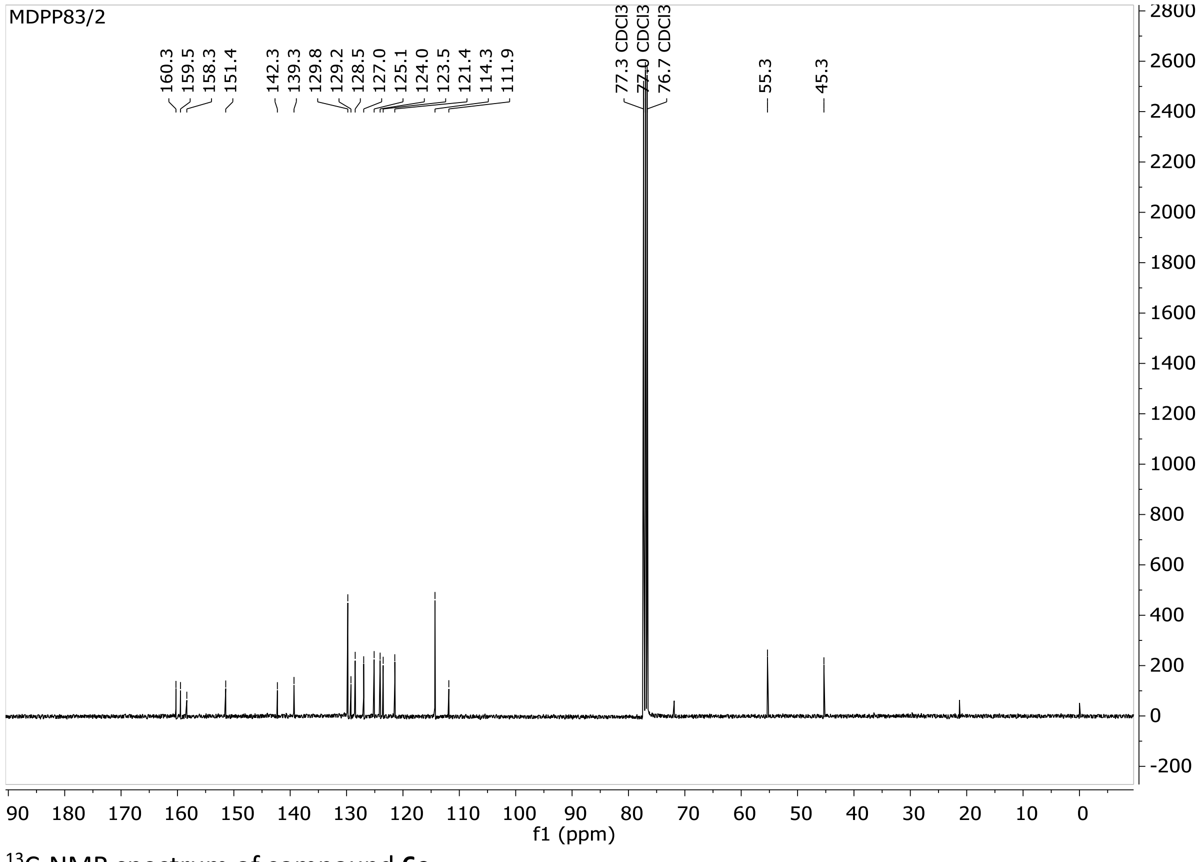

^{13}C-NMR spectrum of compound **6c**

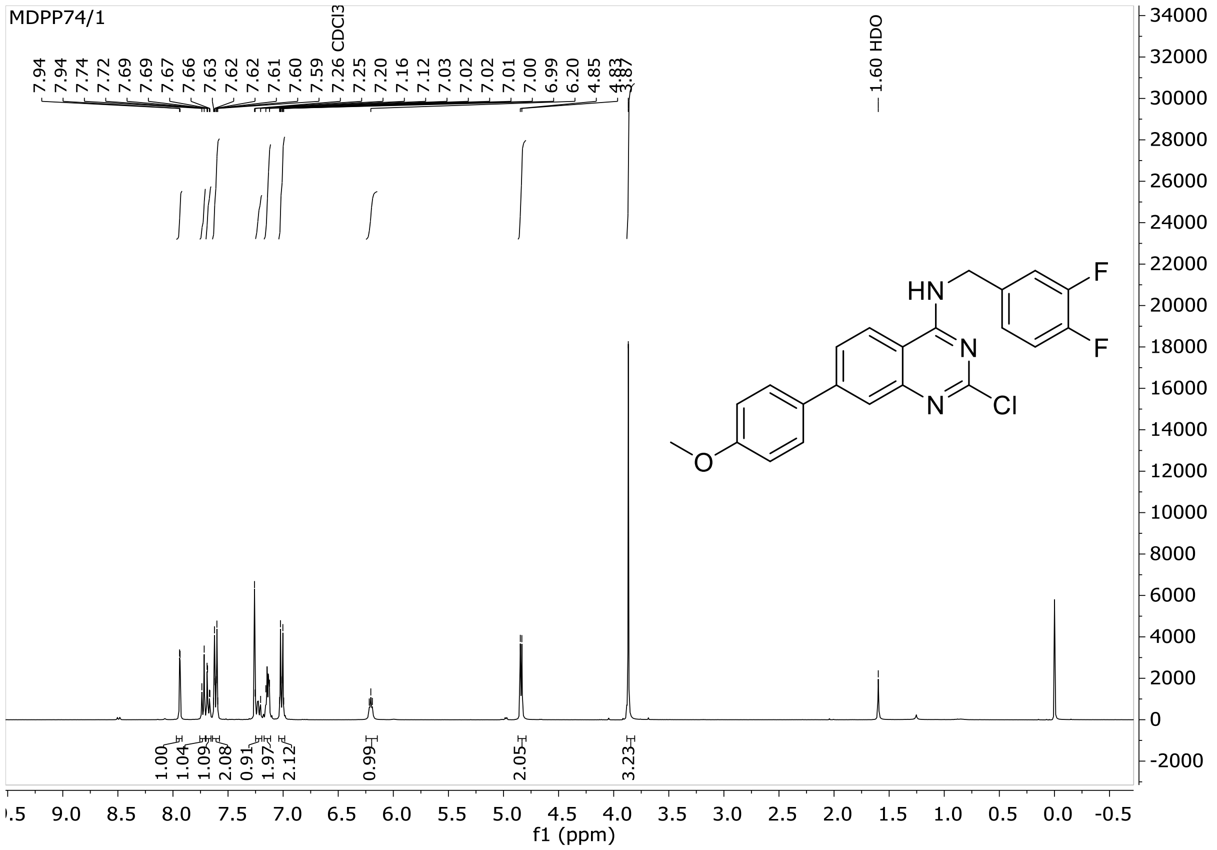

^{1}H-NMR spectrum of compound **6d**

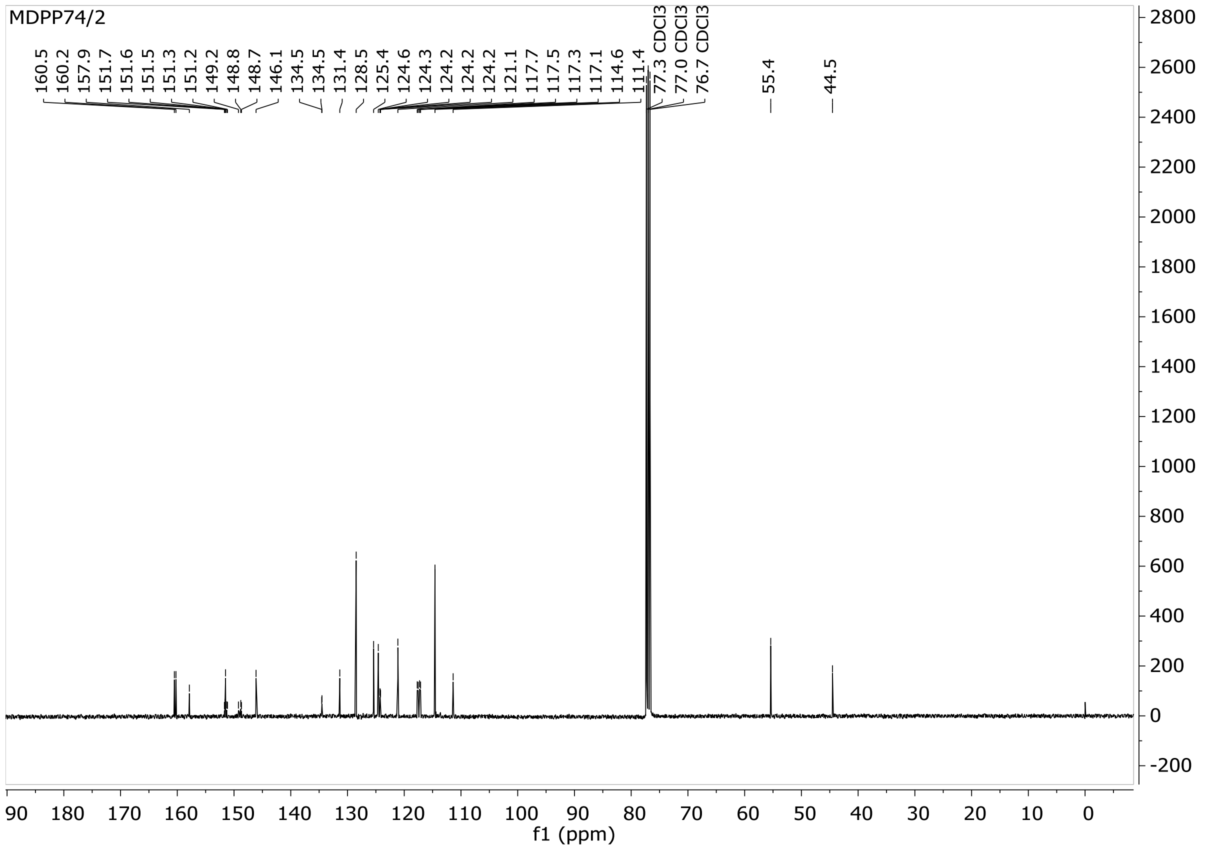

^{13}C-NMR spectrum of compound **6d**

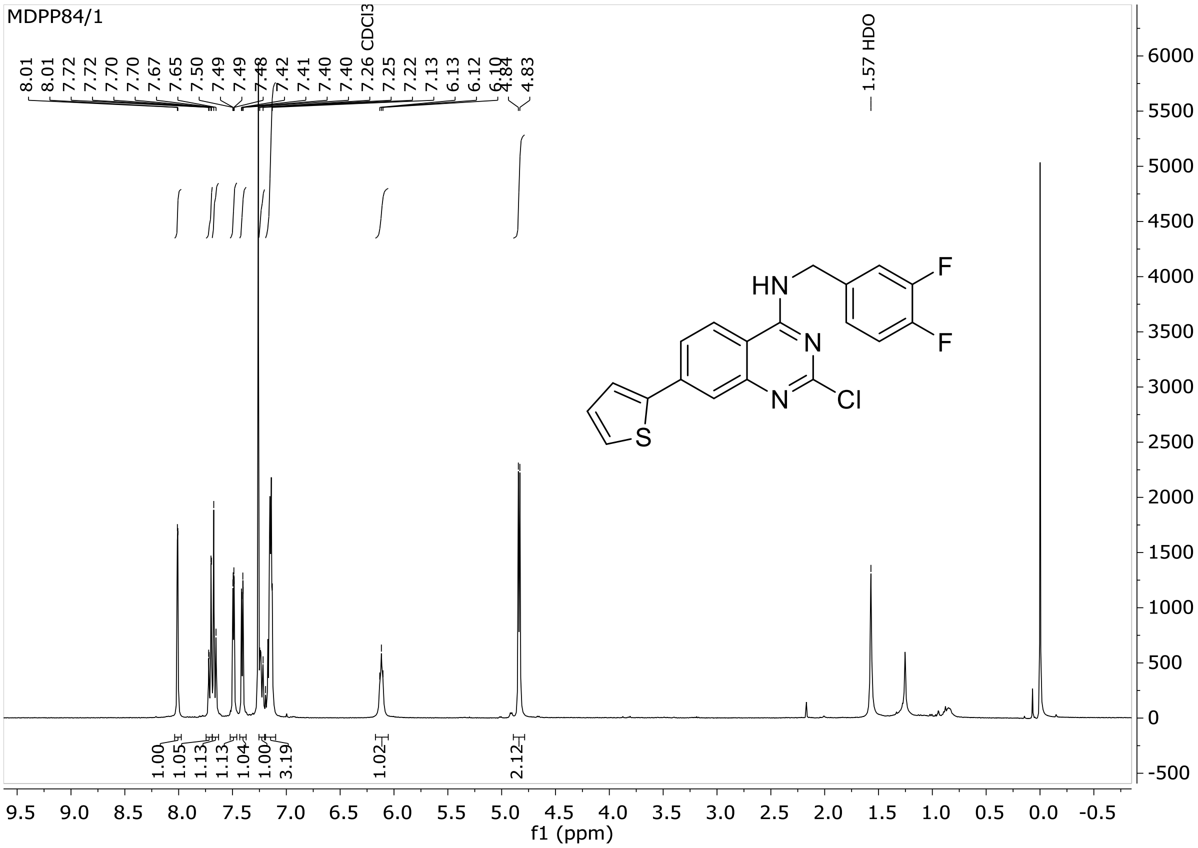

^{1}H-NMR spectrum of compound **6e**

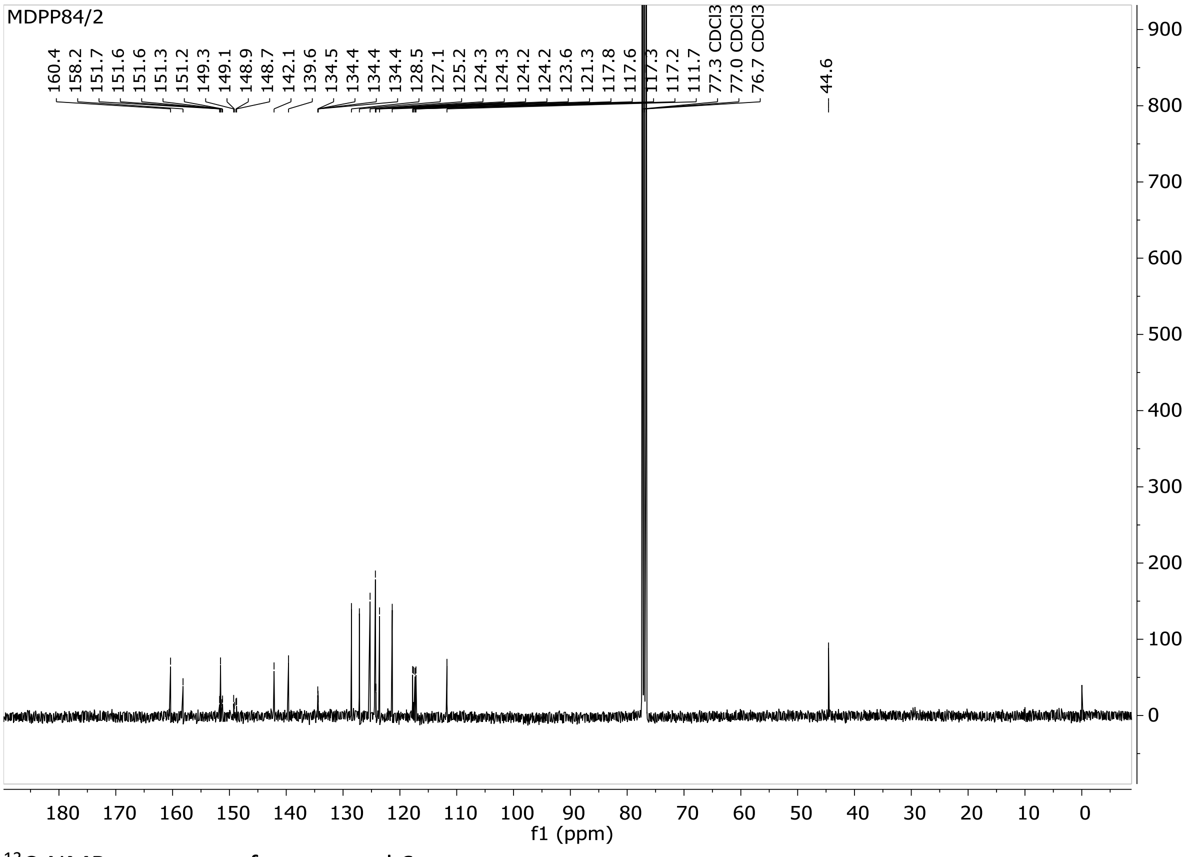

^{13}C-NMR spectrum of compound **6e**

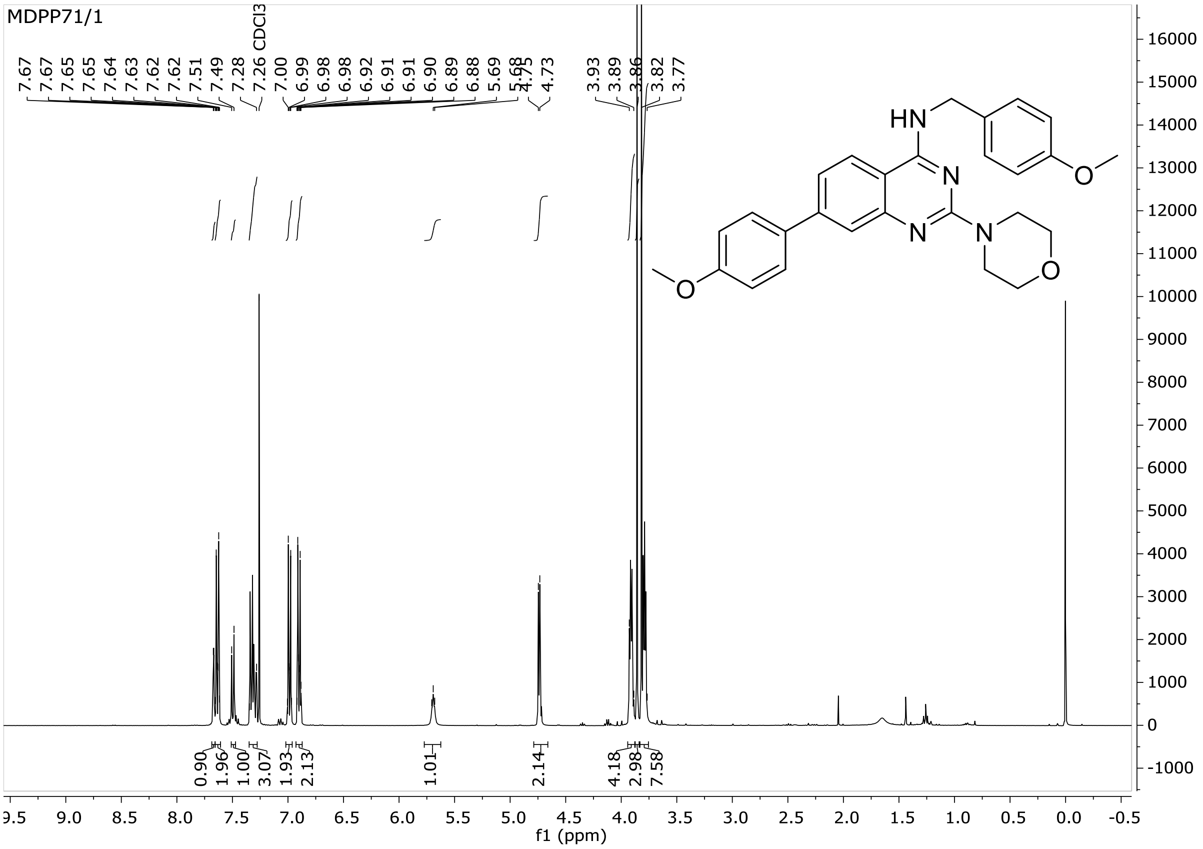

^{1}H-NMR spectrum of compound **7a**

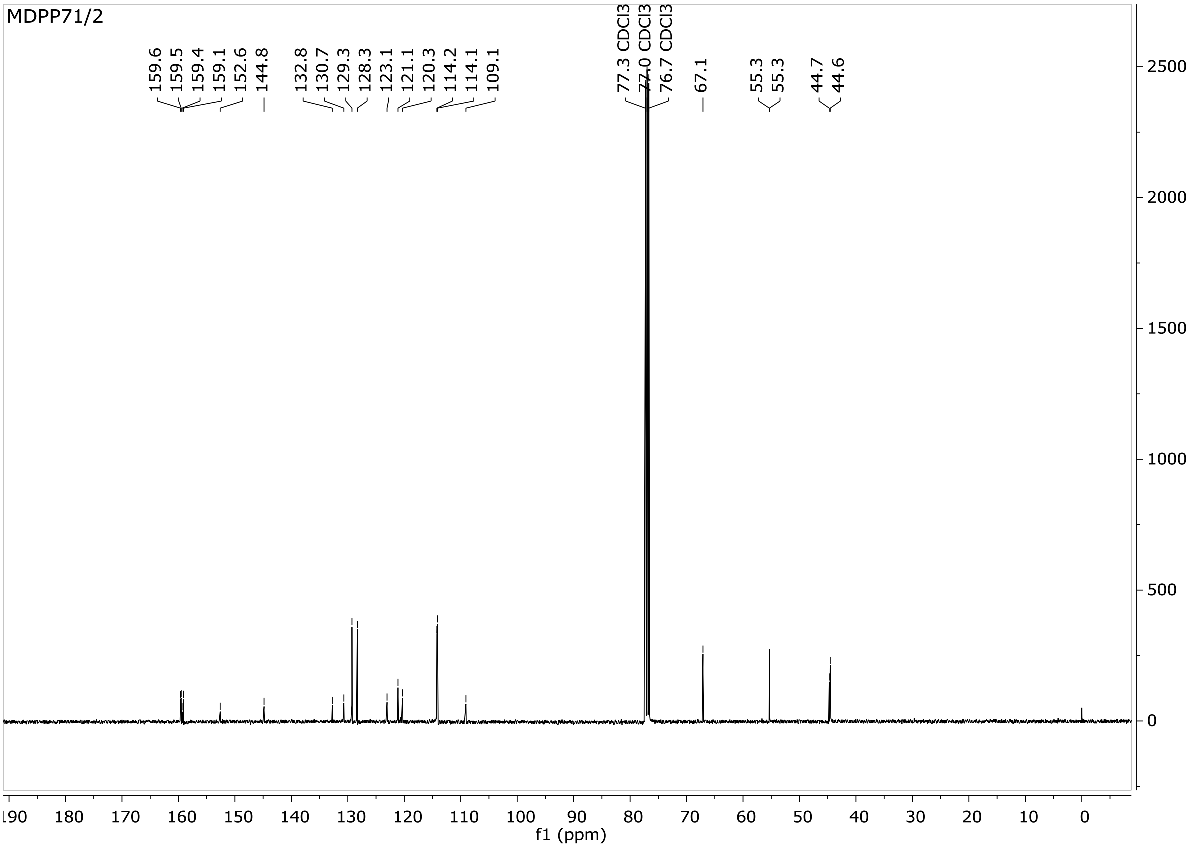

^{13}C-NMR spectrum of compound **7a**

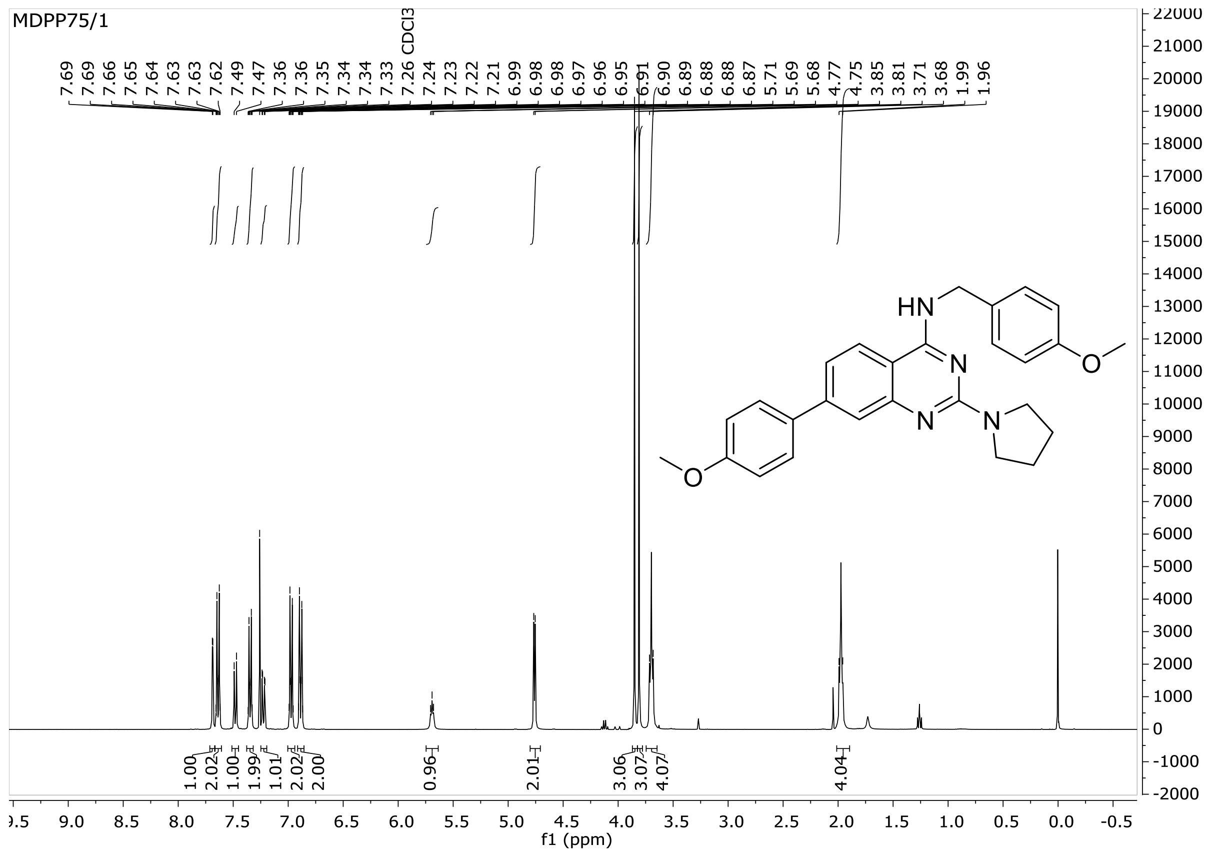

^{1}H-NMR spectrum of compound **7b**

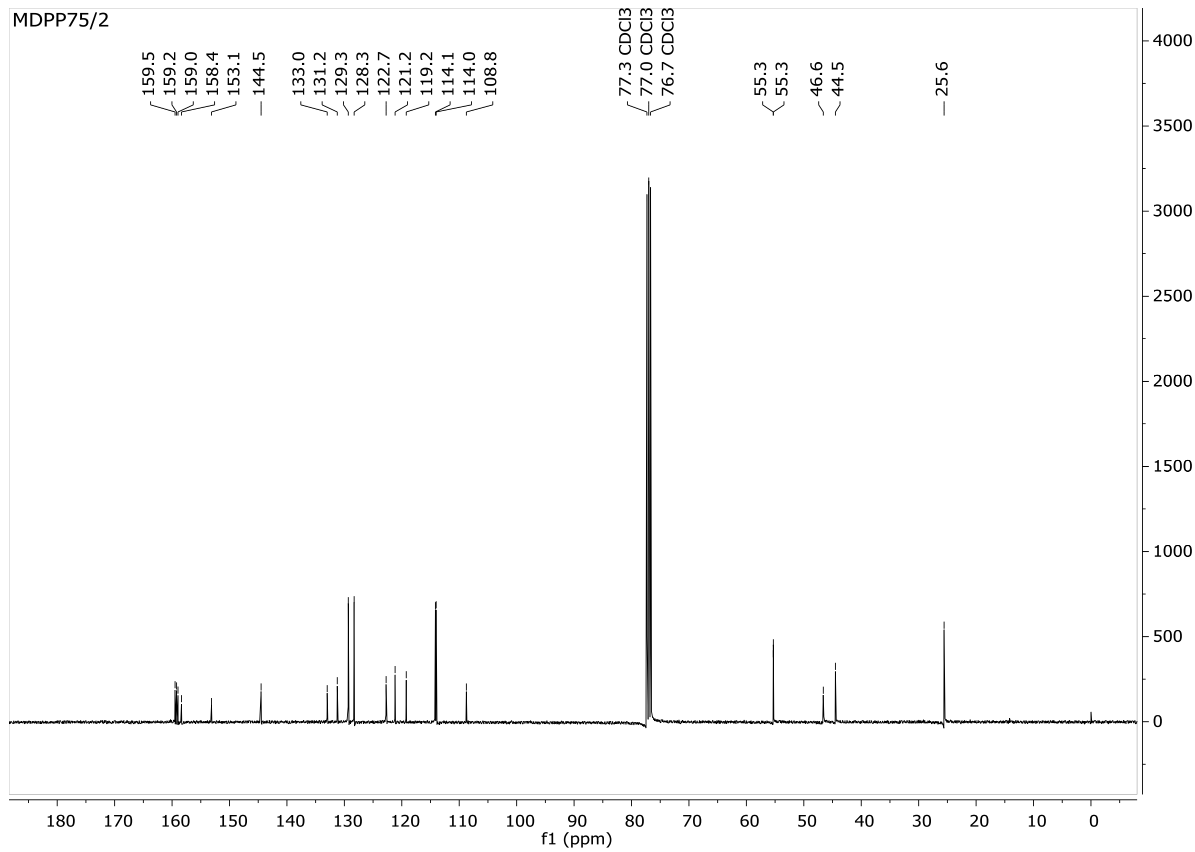

^{13}C-NMR spectrum of compound **7b**

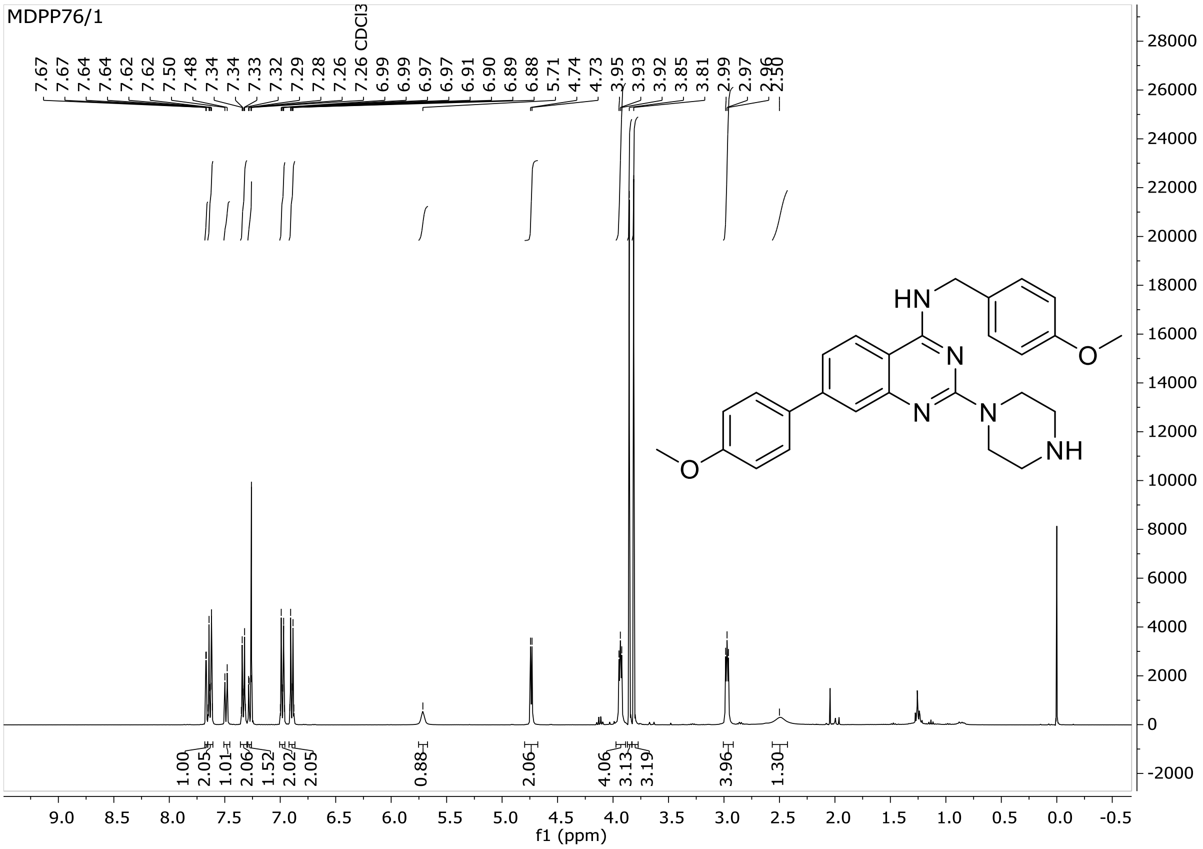

^{1}H-NMR spectrum of compound **7c**

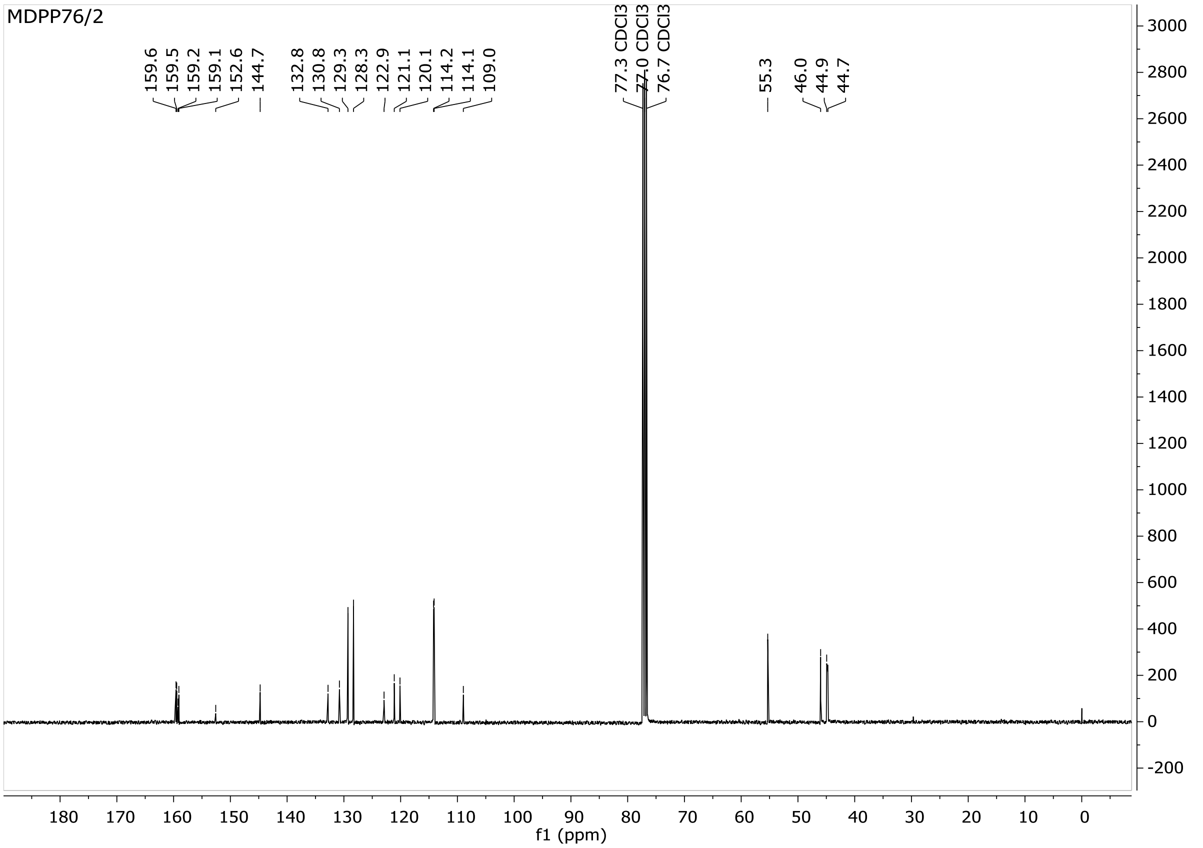

^{13}C-NMR spectrum of compound **7c**

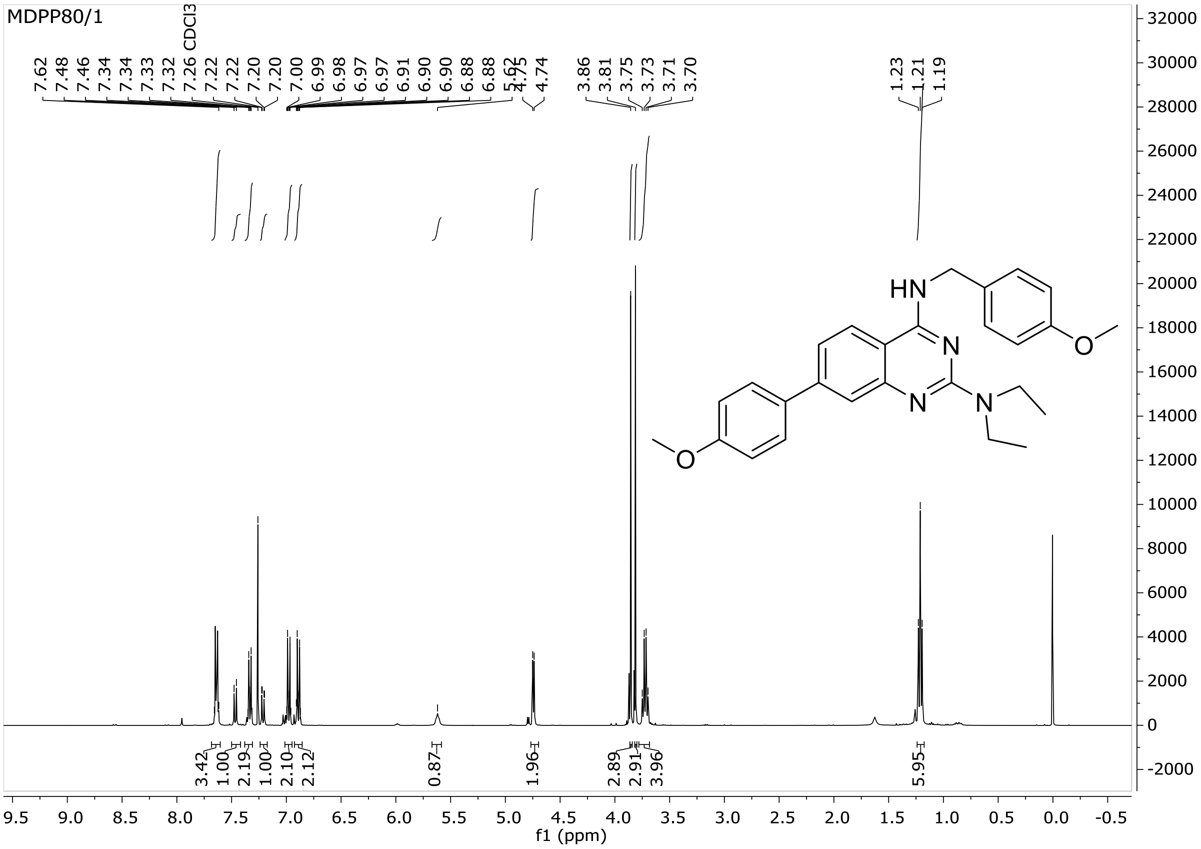

^{1}H-NMR spectrum of compound **7d**

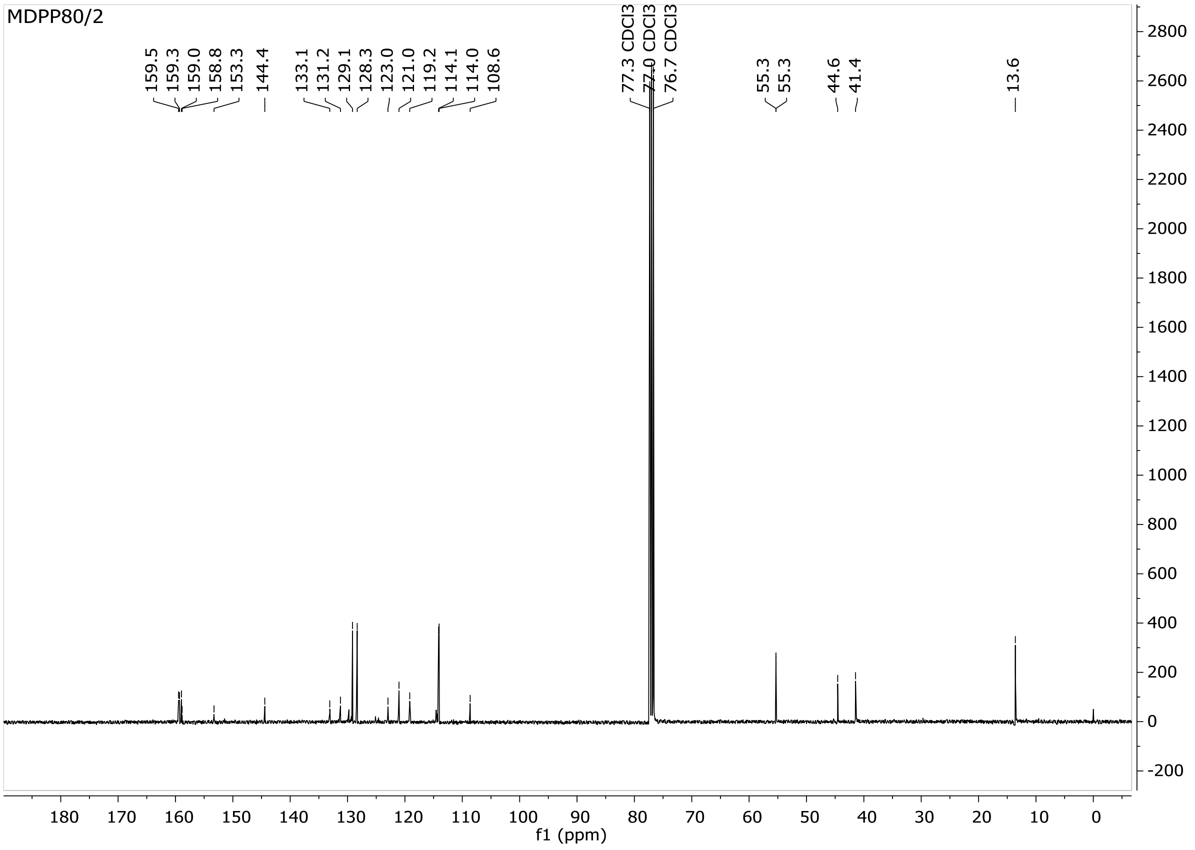

^{13}C-NMR spectrum of compound **7d**

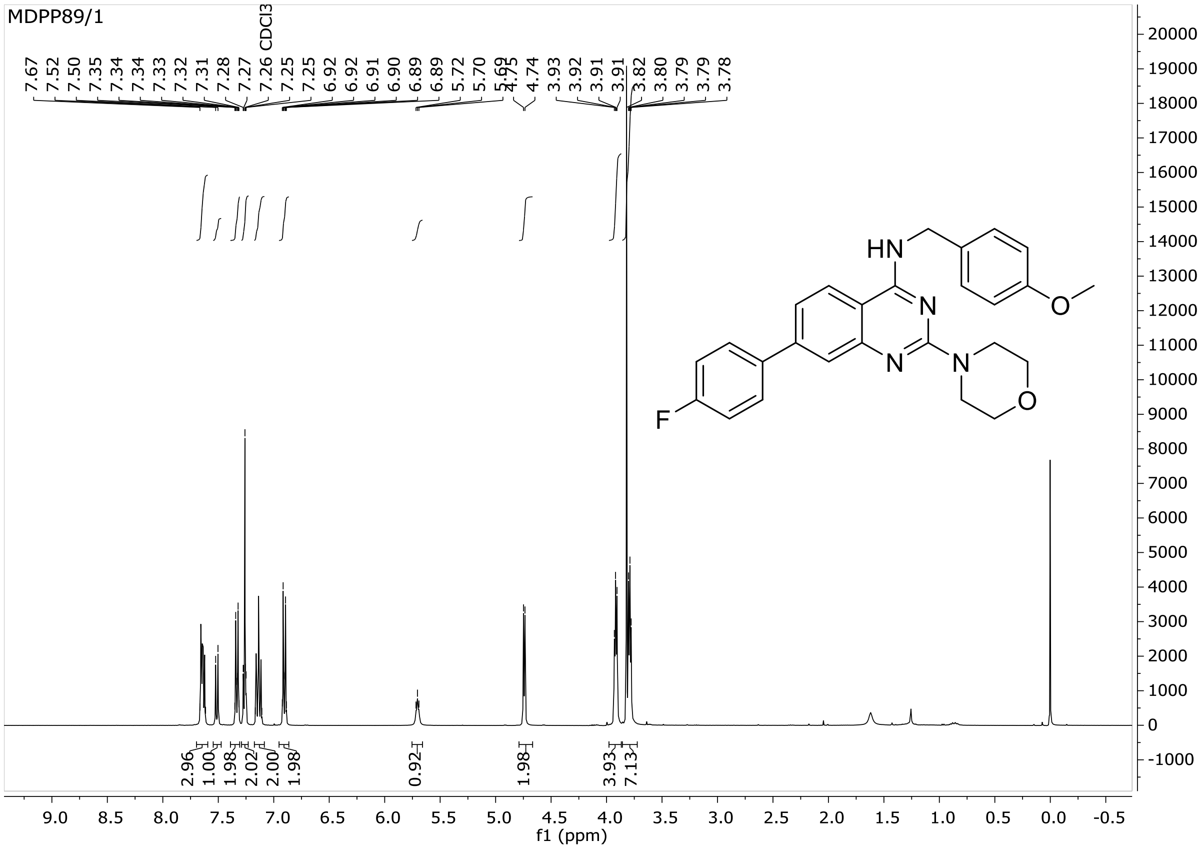

^{1}H-NMR spectrum of compound **7e**

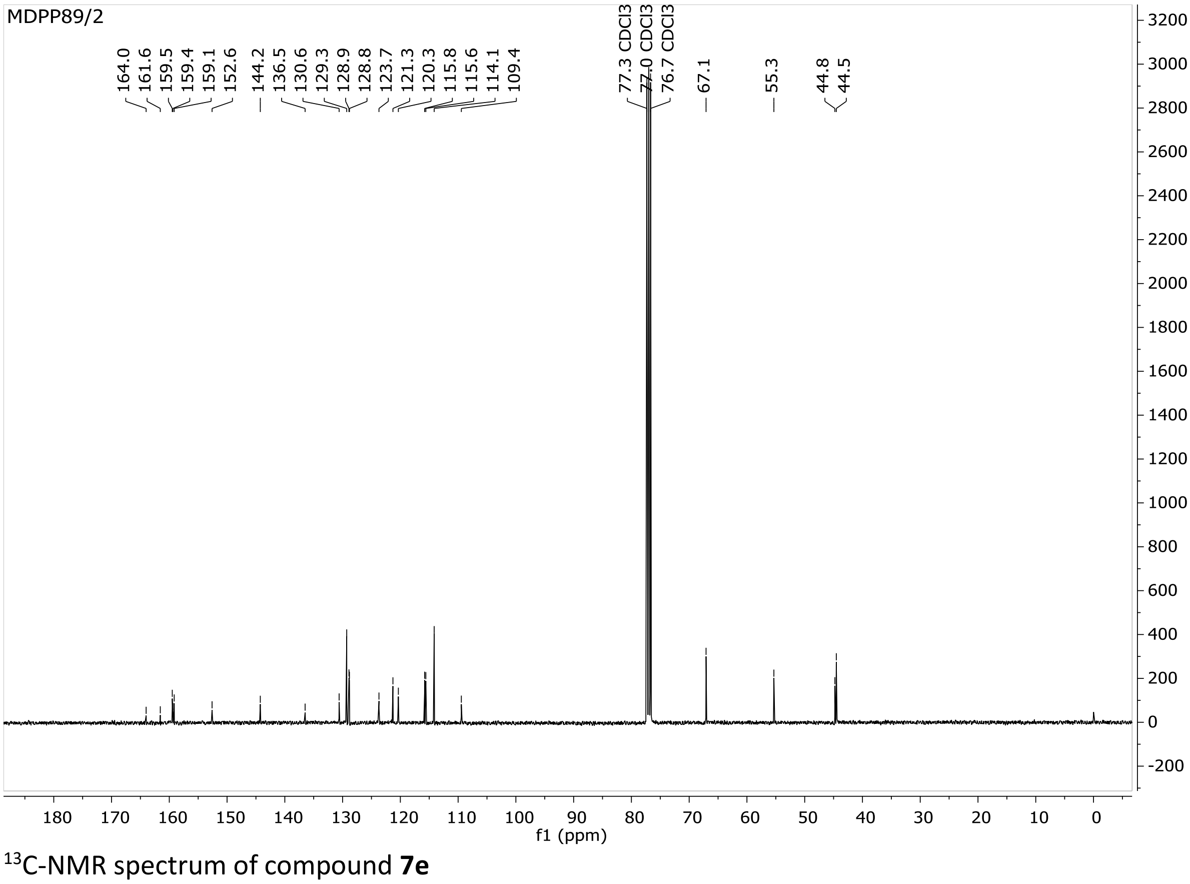

^{13}C-NMR spectrum of compound **7e**

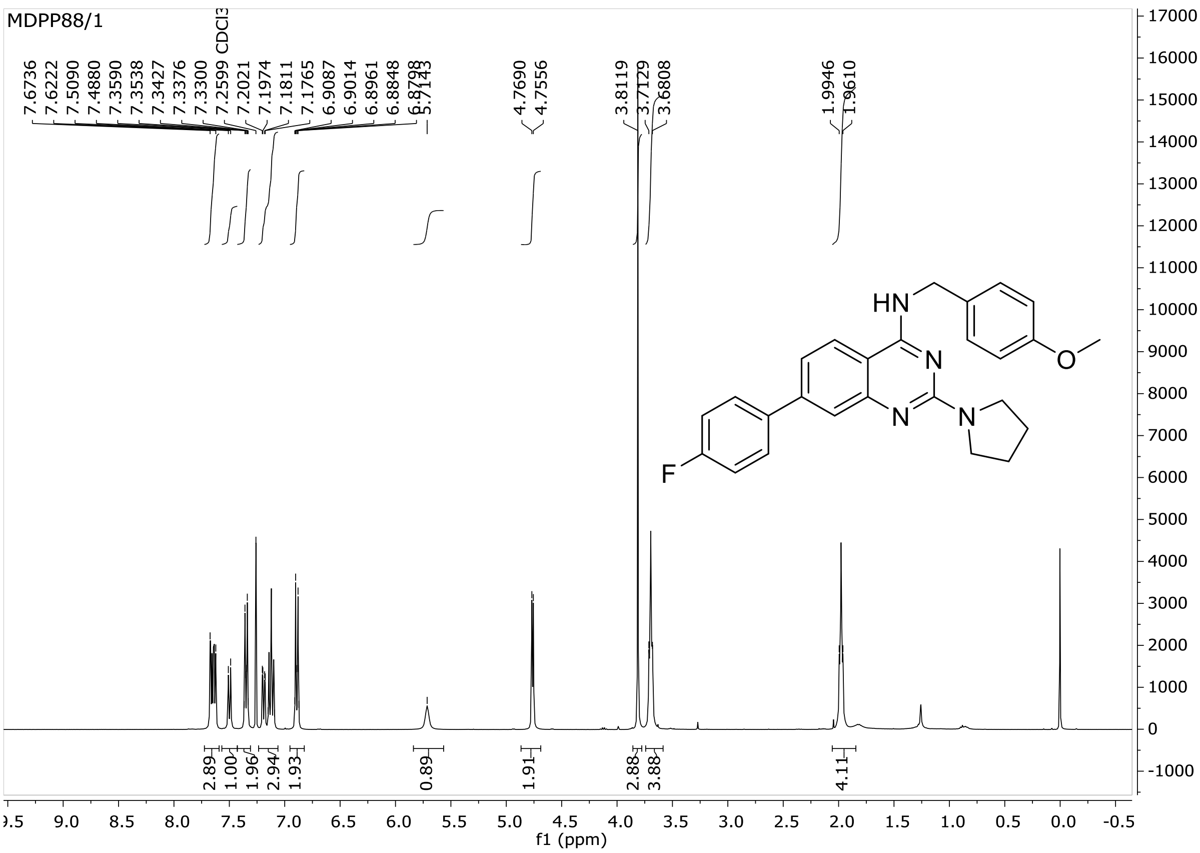

^{1}H-NMR spectrum of compound **7f**

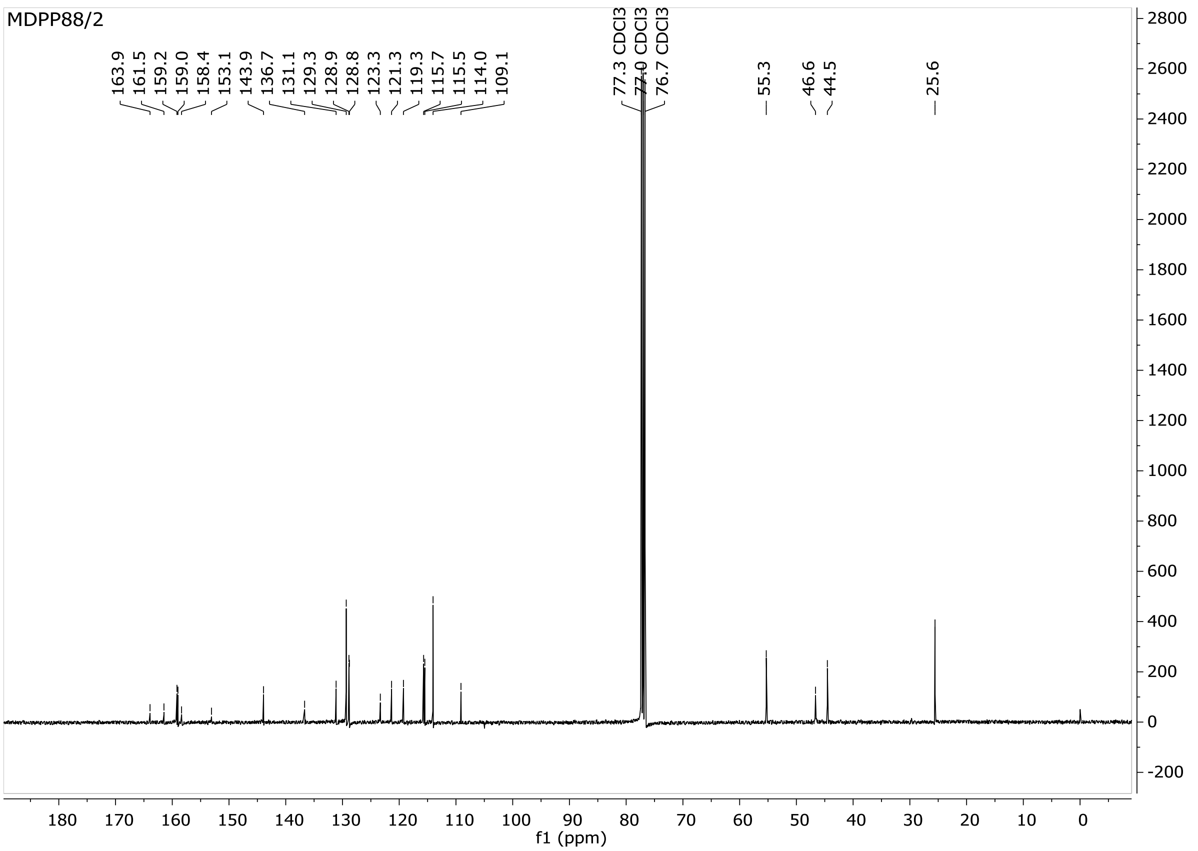

^{13}C-NMR spectrum of compound **7f**

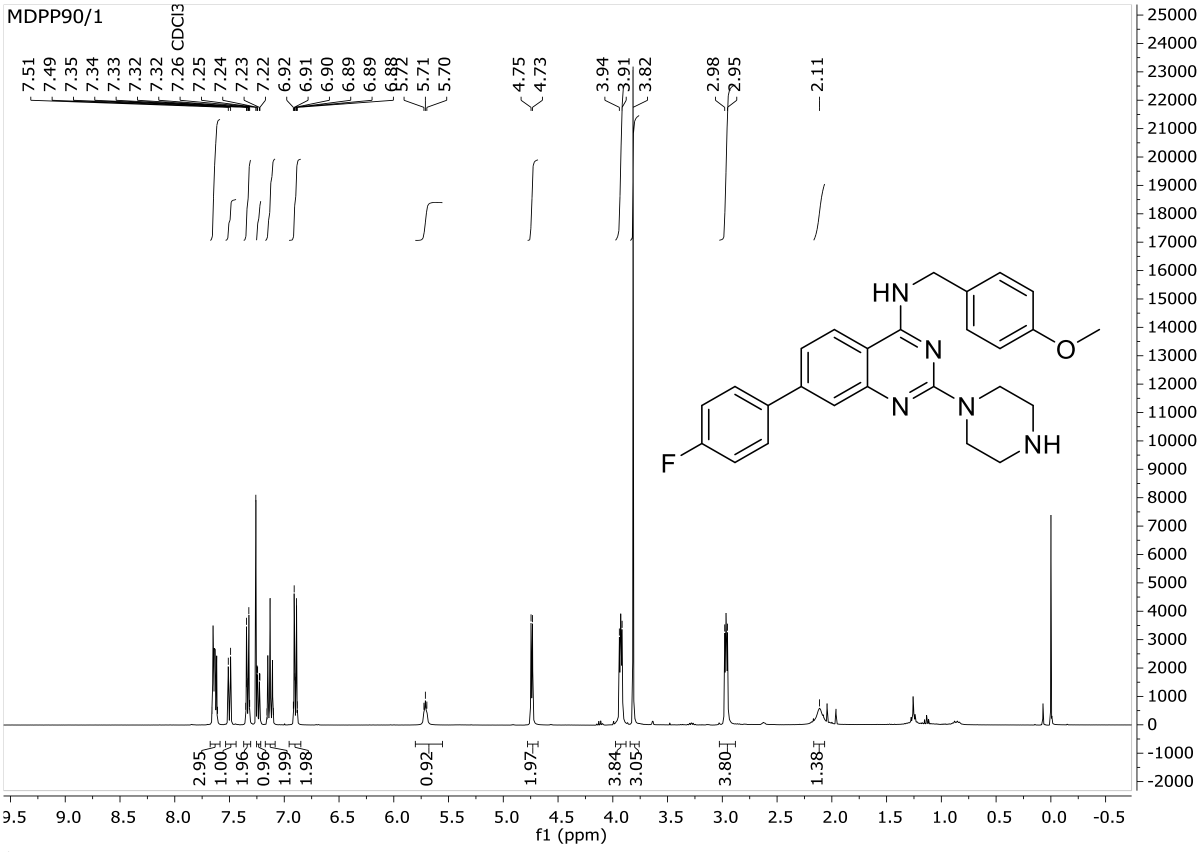

^{1}H-NMR spectrum of compound **7g**

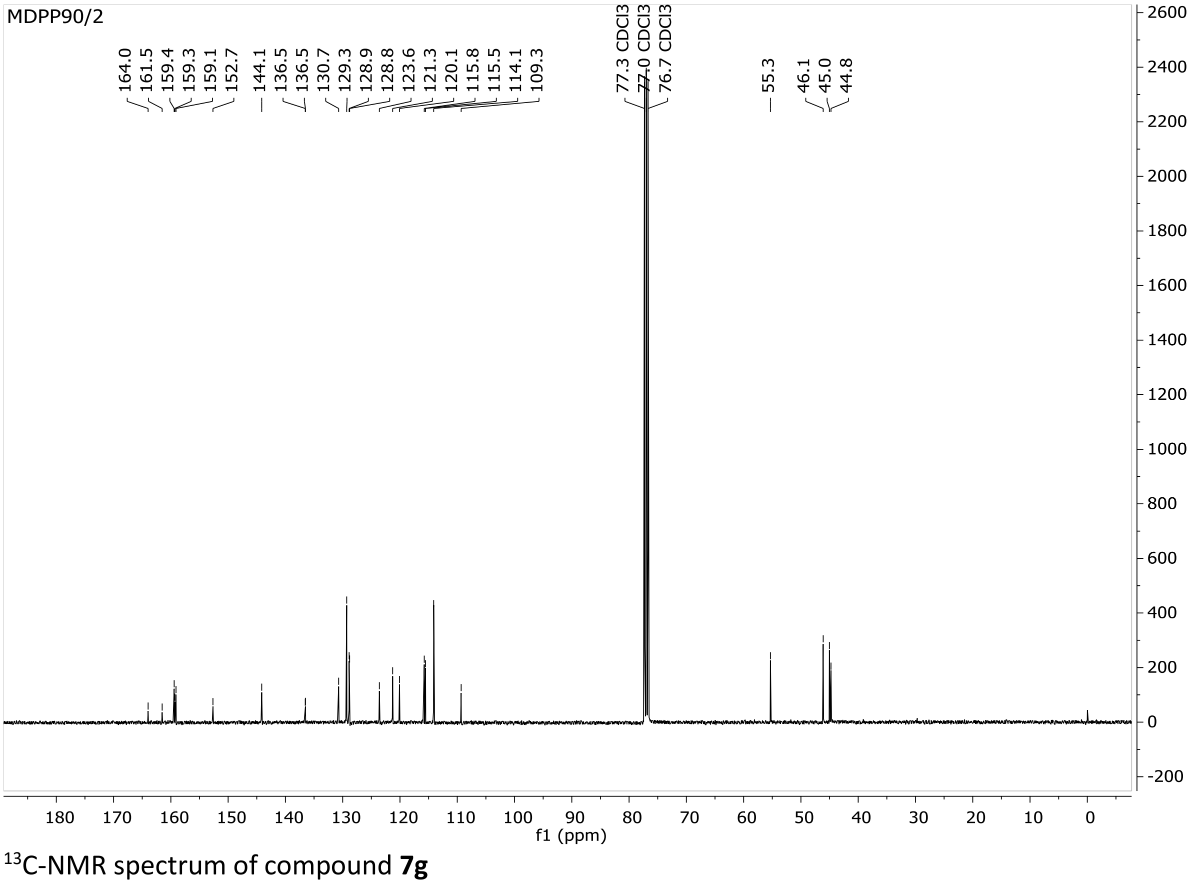

^{13}C-NMR spectrum of compound **7g**

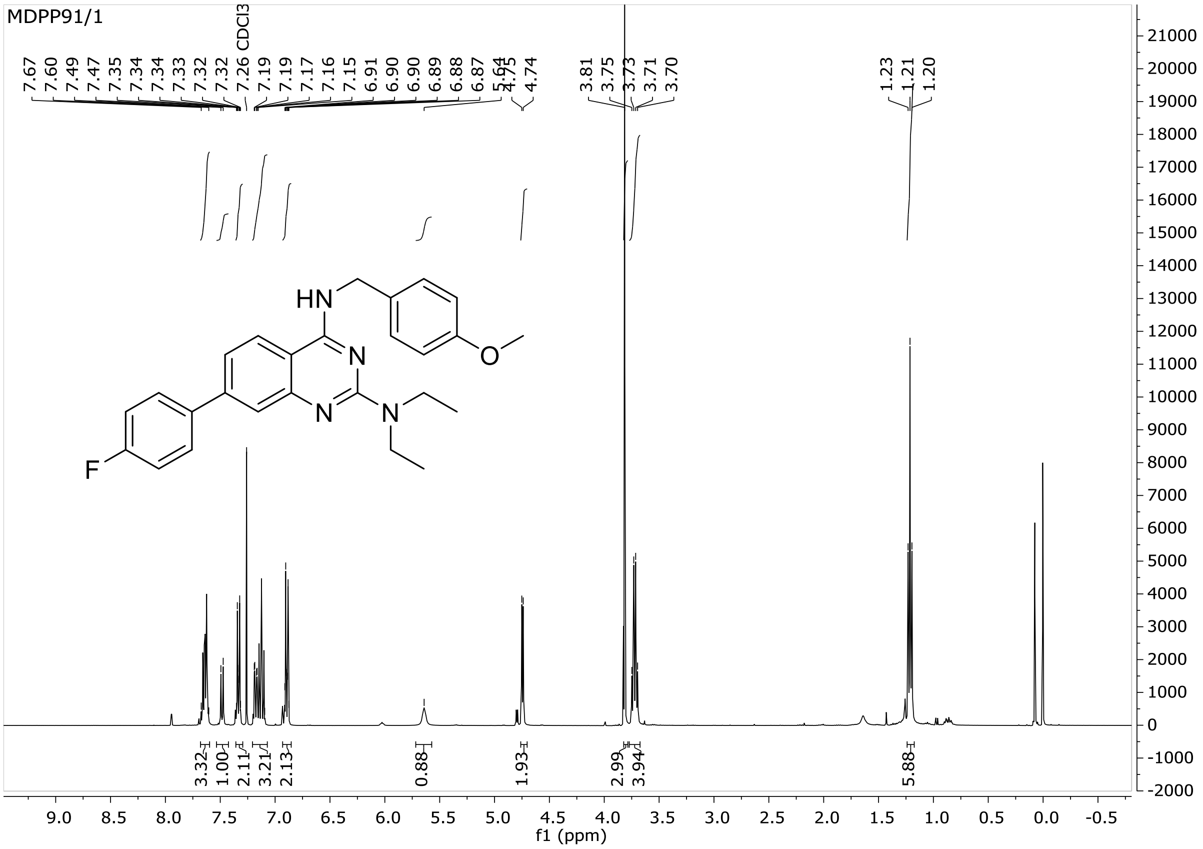

^{1}H-NMR spectrum of compound **7h**

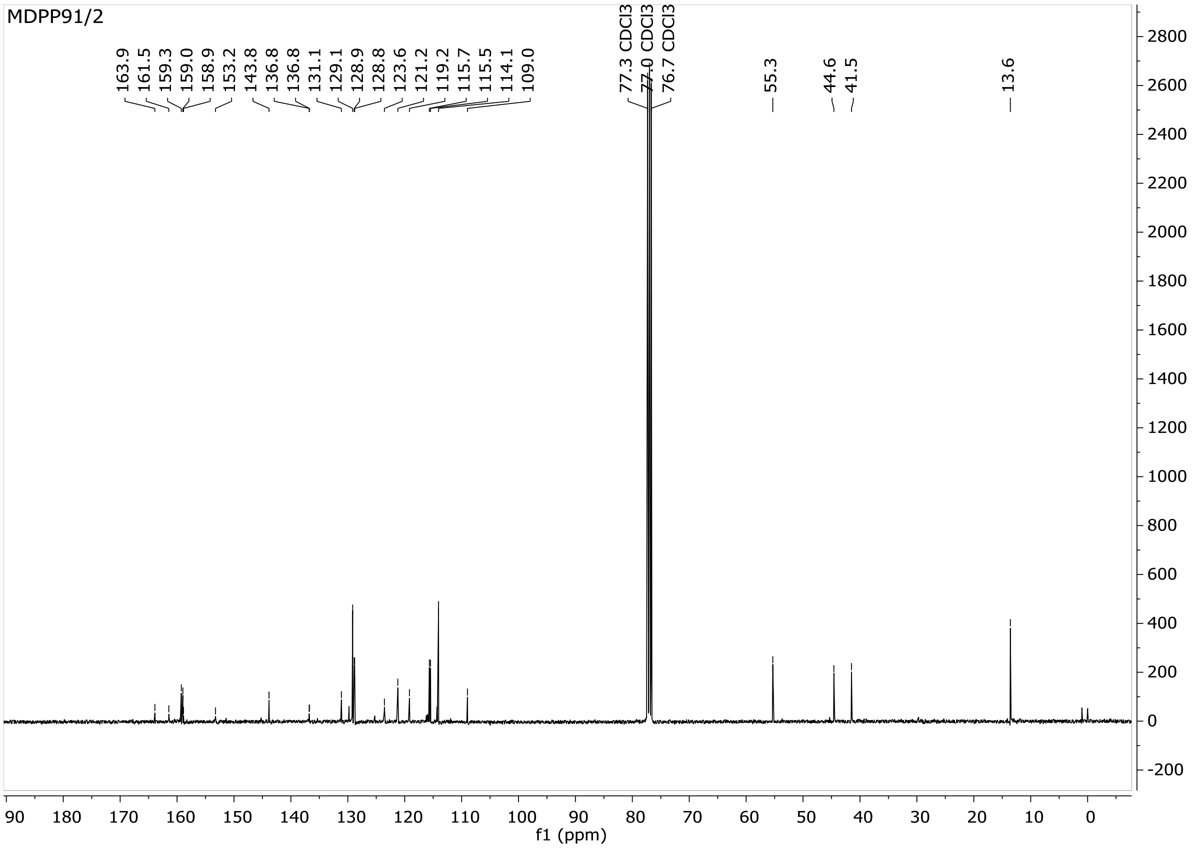

^{13}C-NMR spectrum of compound **7h**

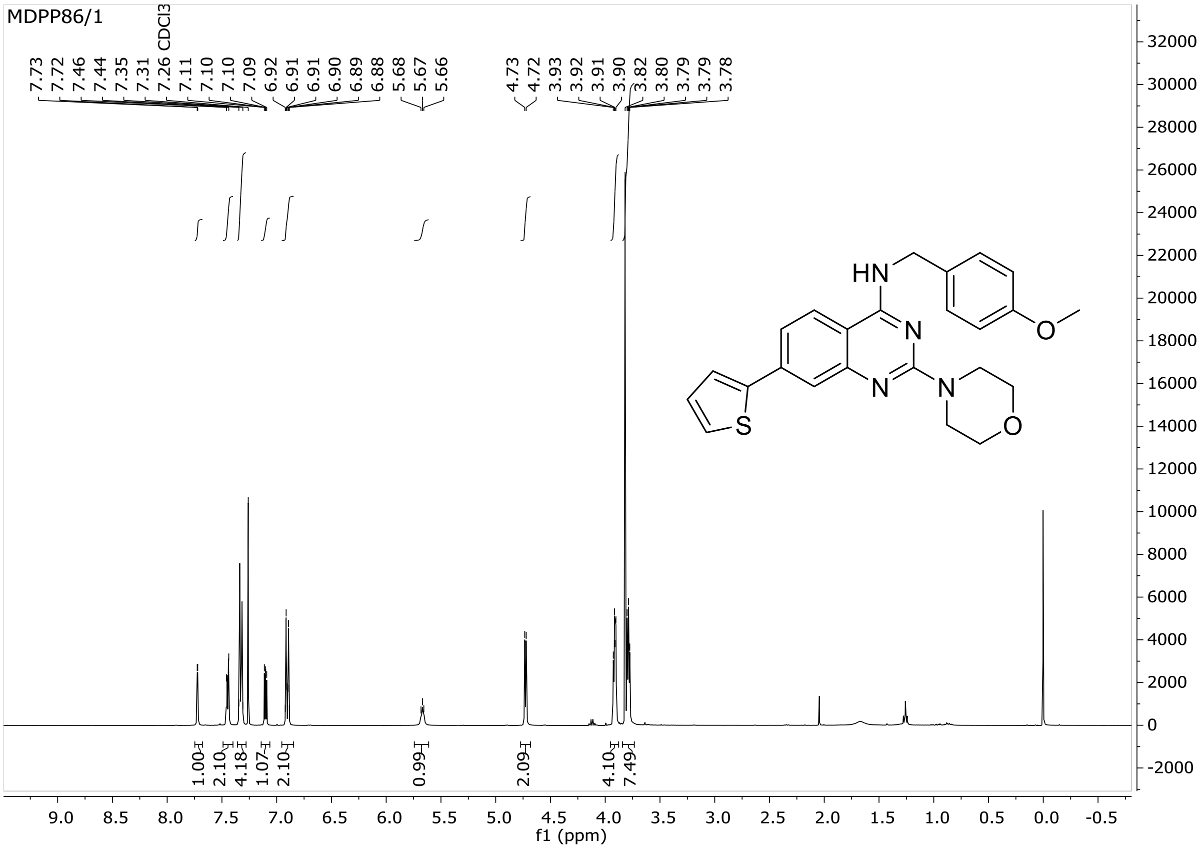

^{1}H-NMR spectrum of compound **7i**

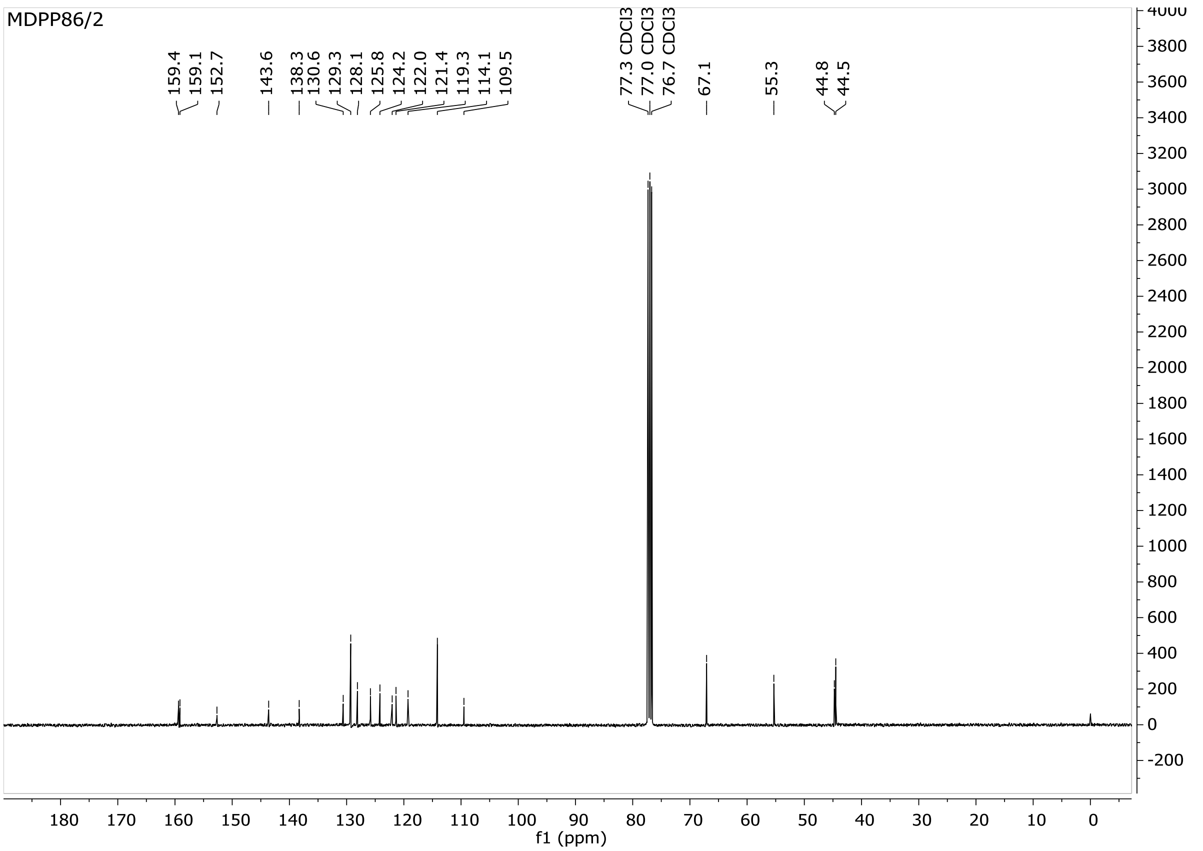

^{13}C-NMR spectrum of compound **7i**

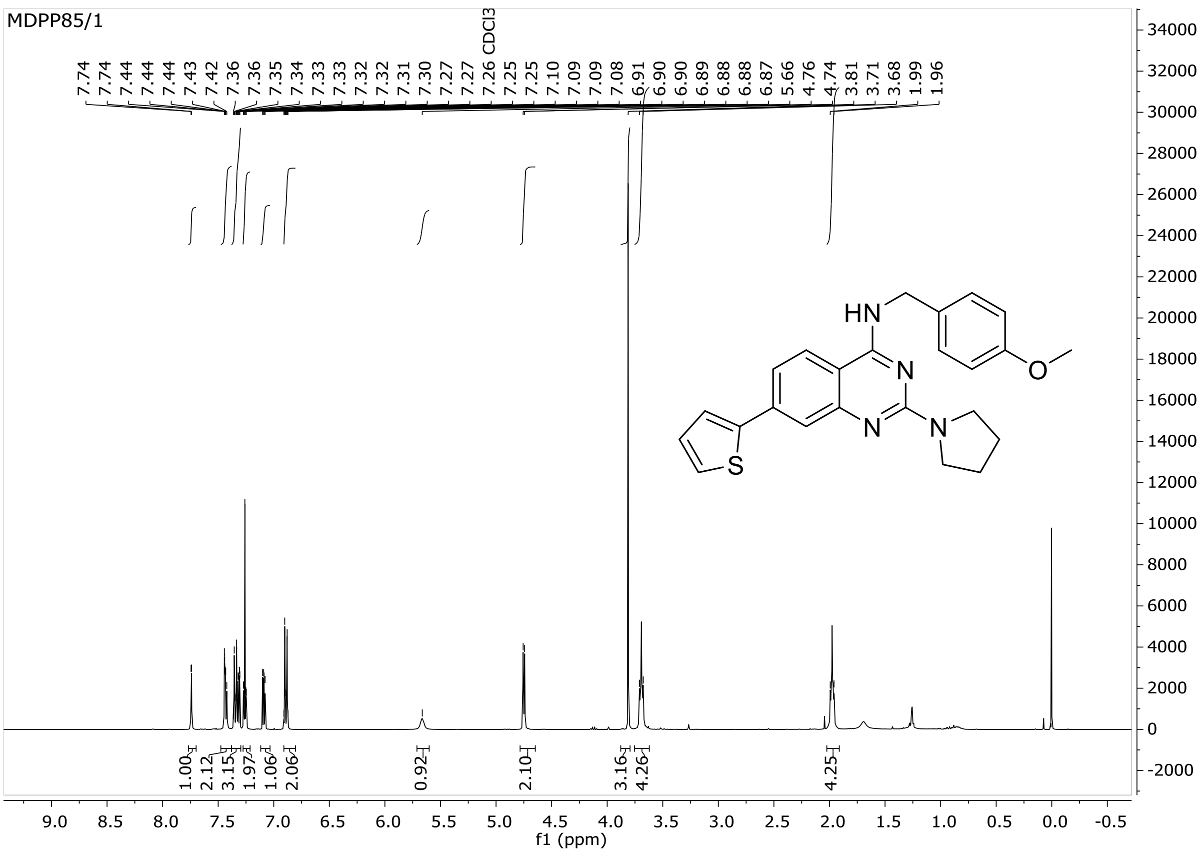

^{1}H-NMR spectrum of compound **7j**

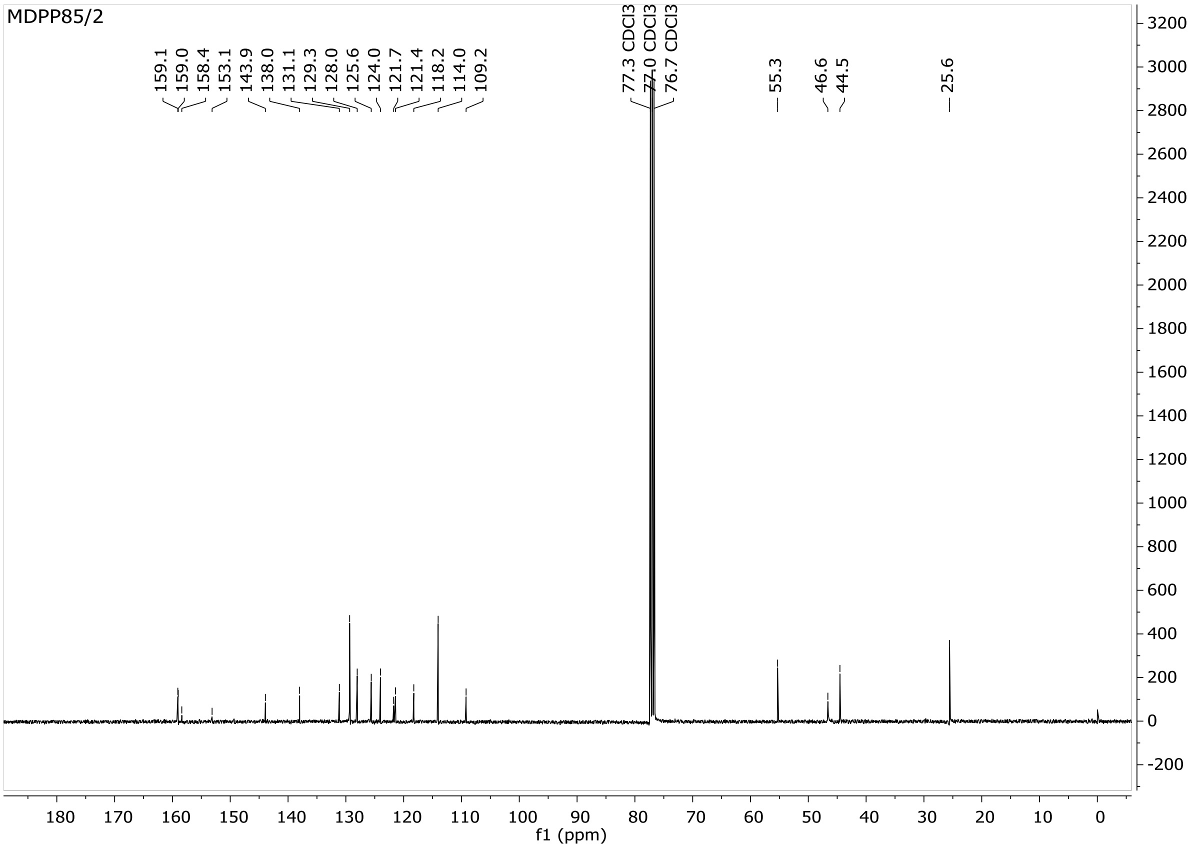

^{13}C-NMR spectrum of compound **7j**

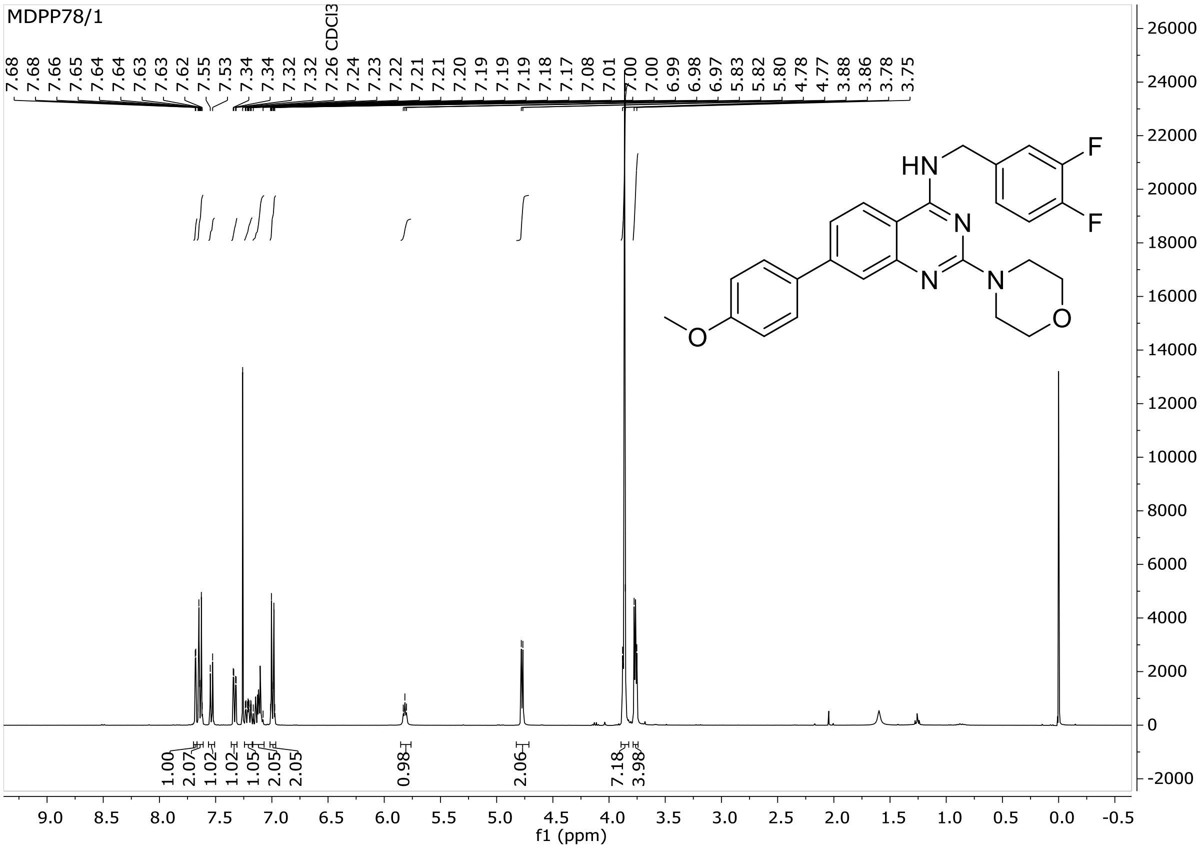

^{1}H-NMR spectrum of compound **7k**

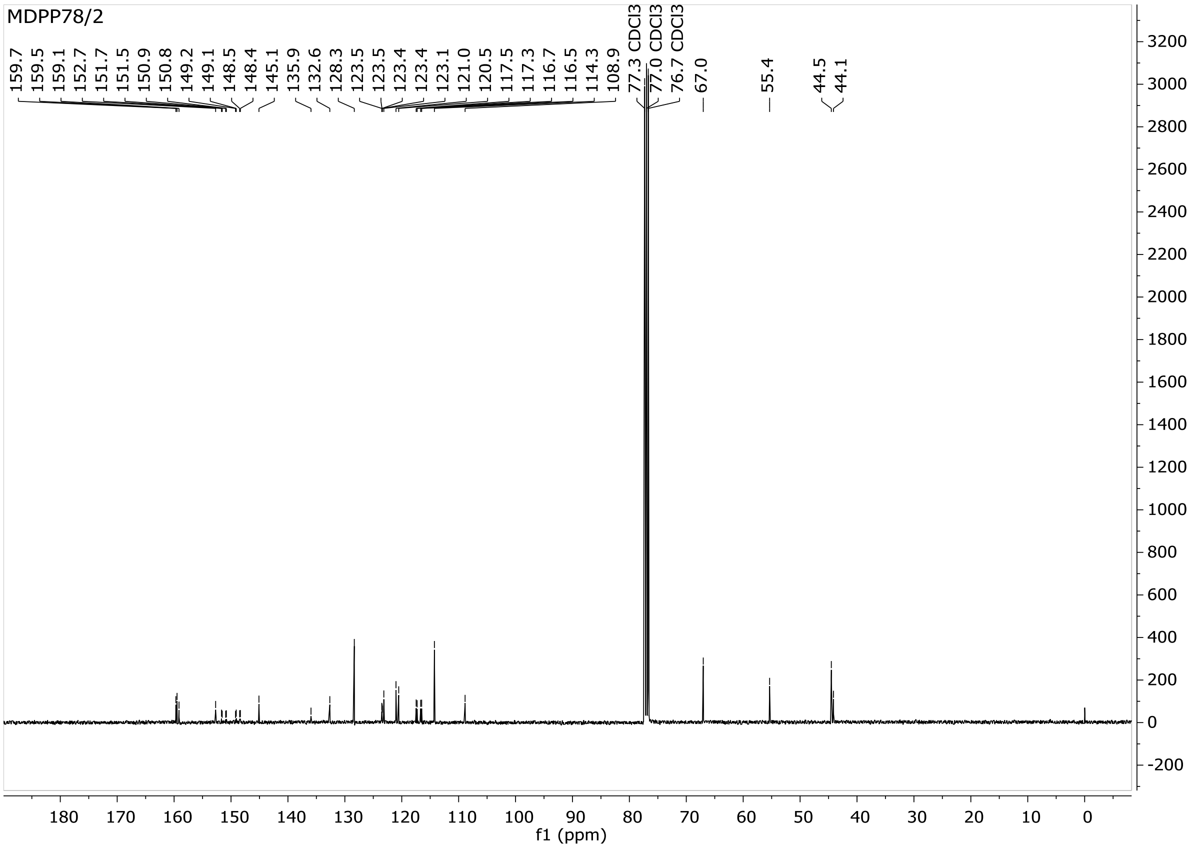

^{13}C-NMR spectrum of compound **7k**

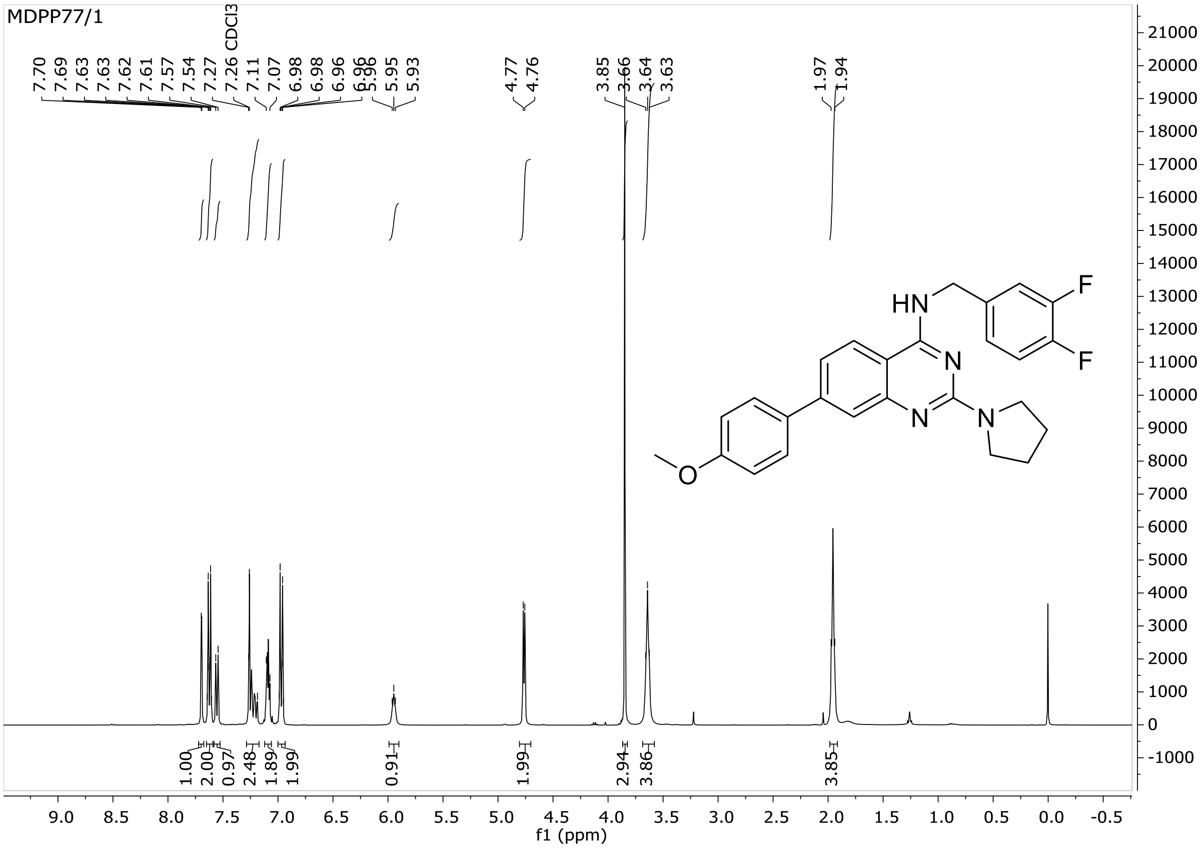

^{1}H-NMR spectrum of compound **7l**

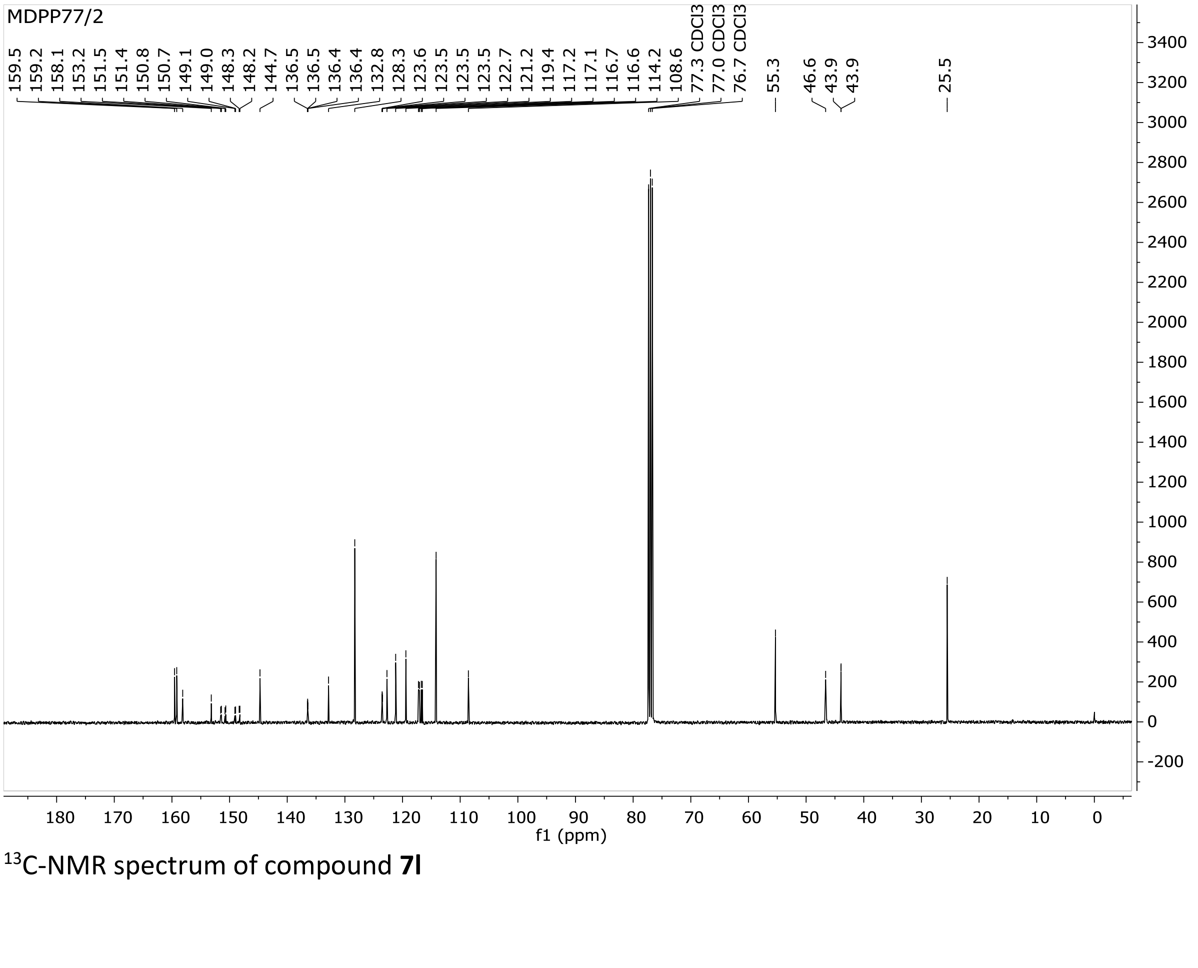

^{13}C-NMR spectrum of compound **7l**

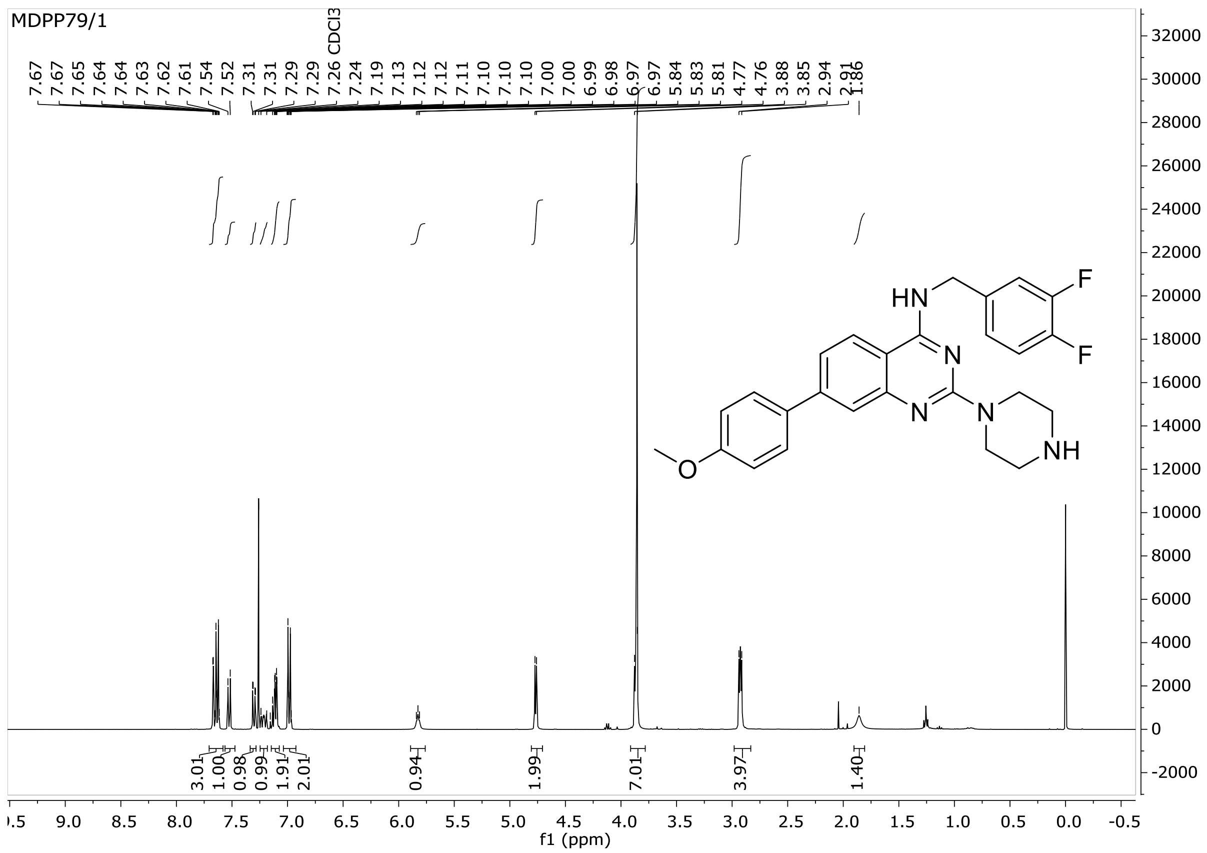

^{1}H-NMR spectrum of compound **7m**

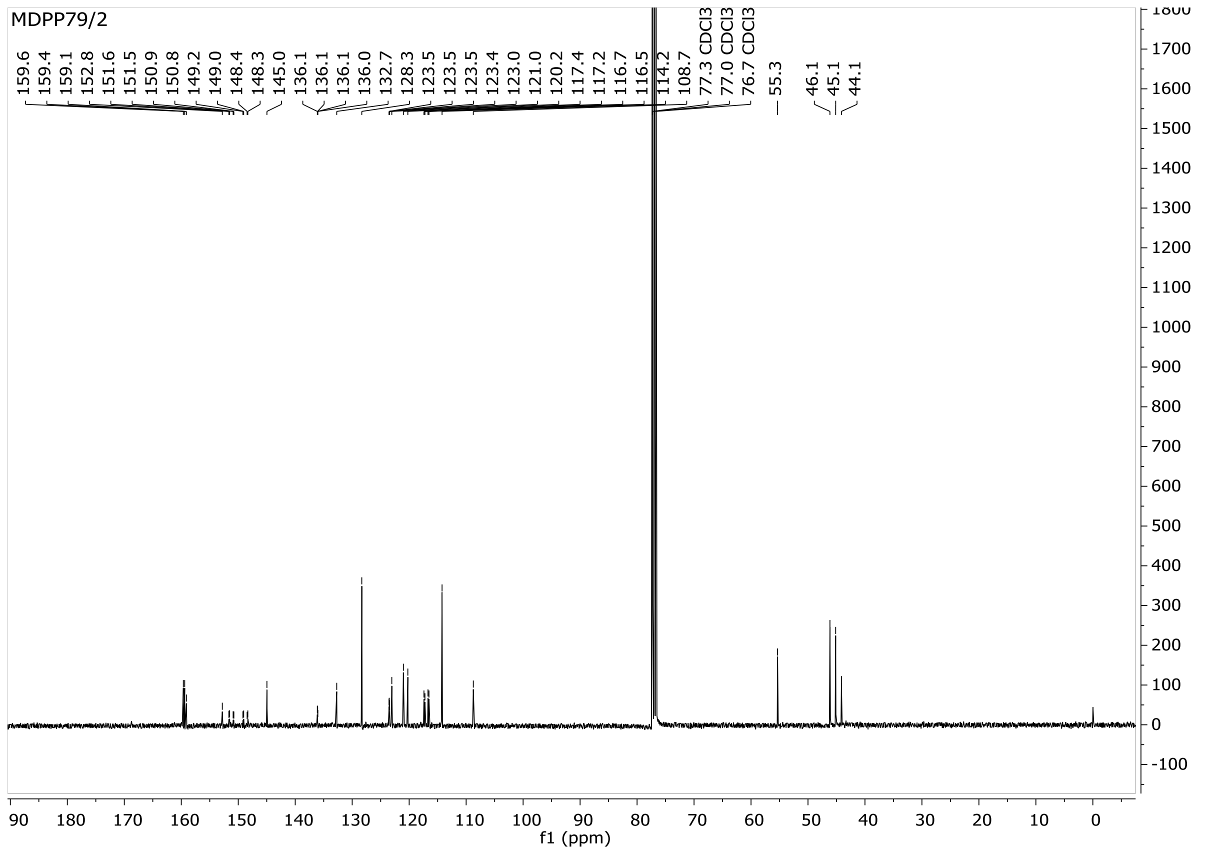

^{13}C-NMR spectrum of compound **7m**

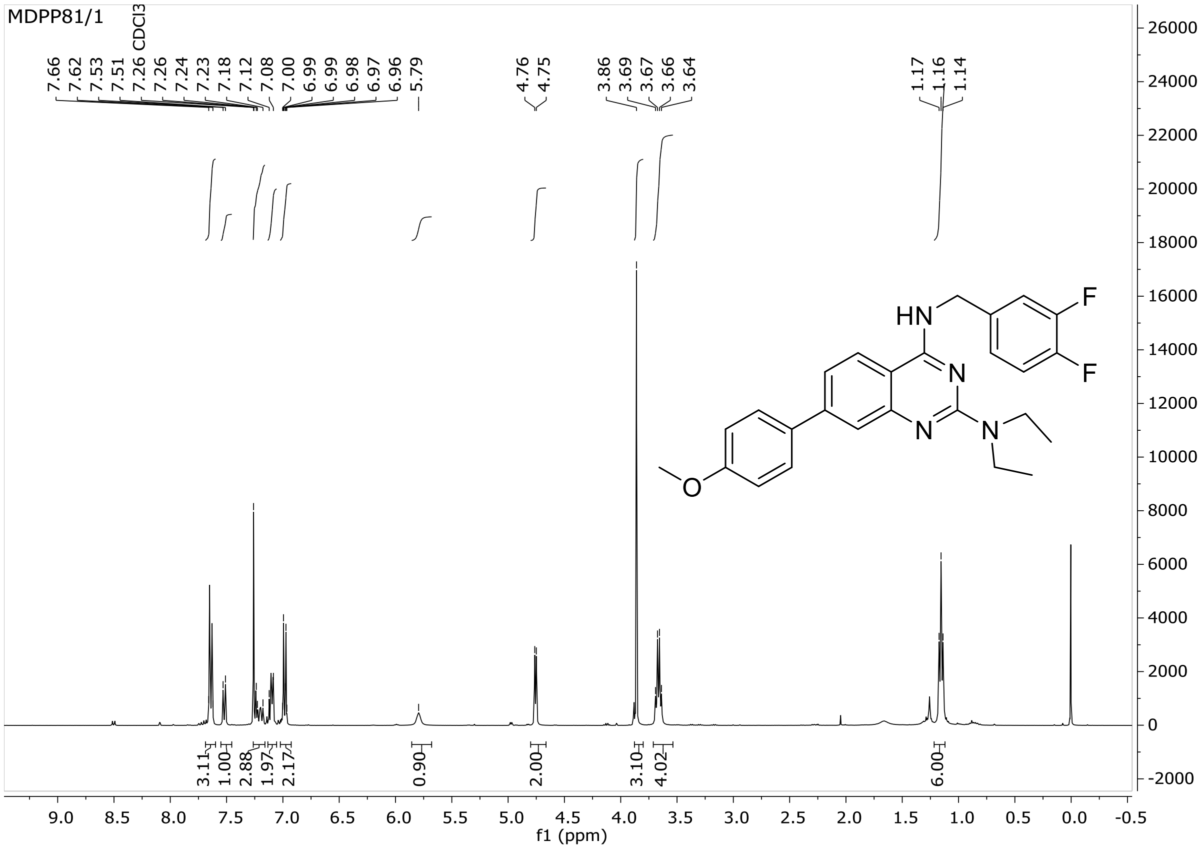

^{1}H-NMR spectrum of compound **7n**

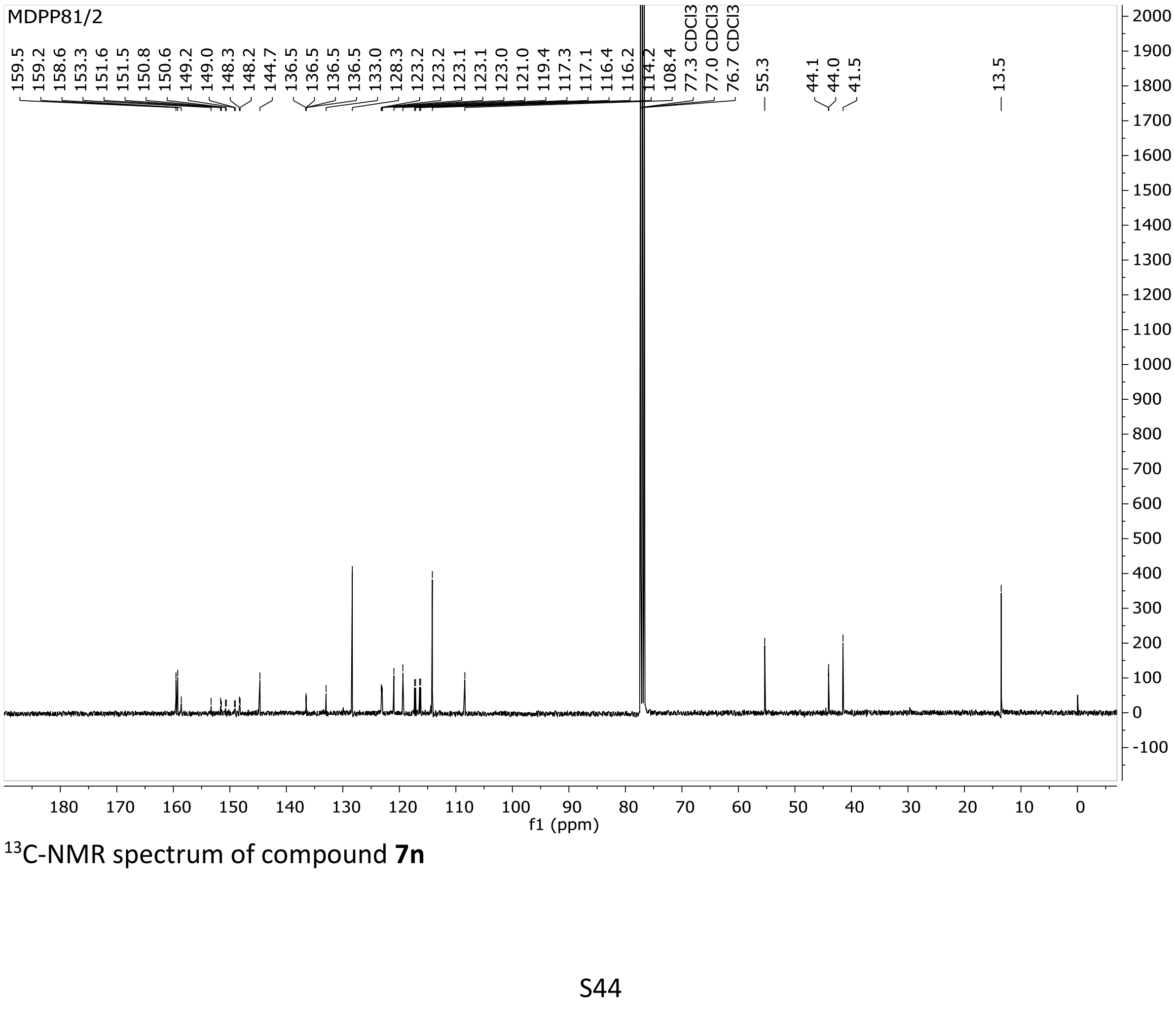

^{13}C-NMR spectrum of compound **7n**

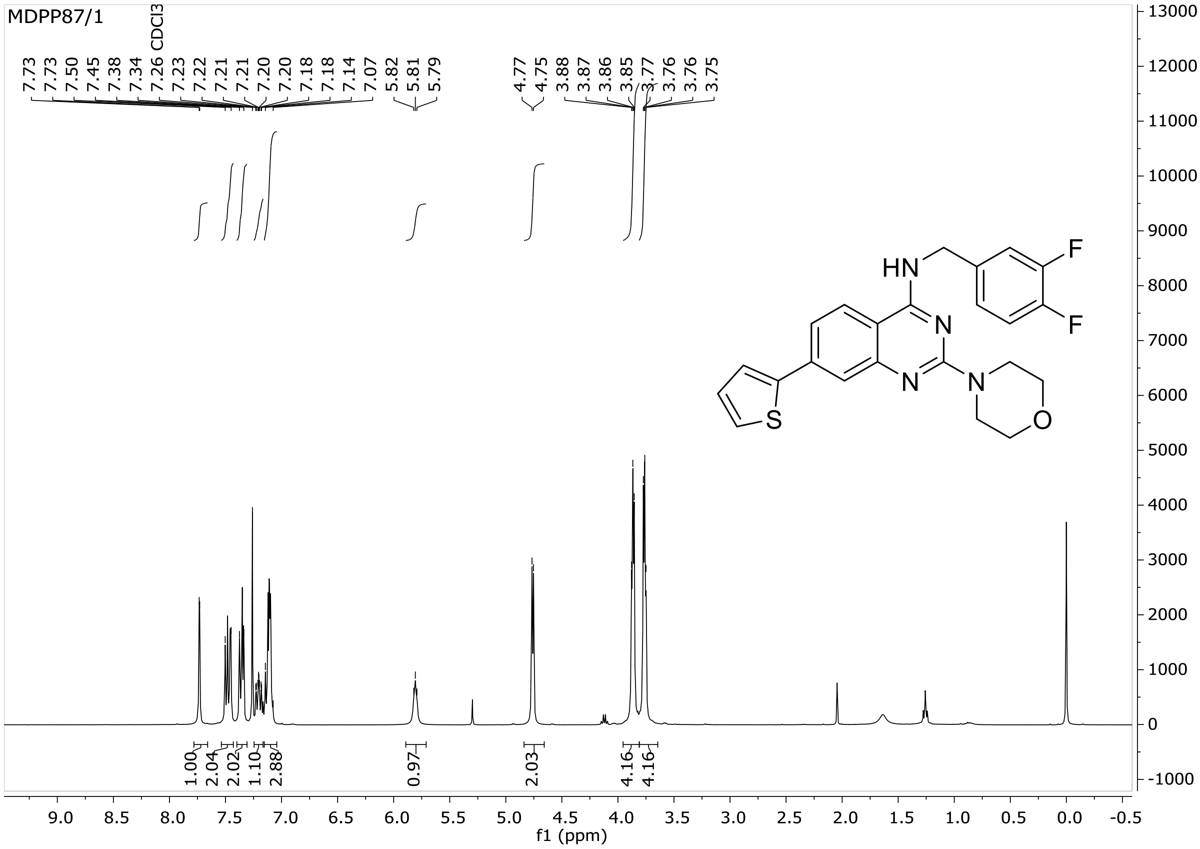

^{1}H-NMR spectrum of compound **7o**

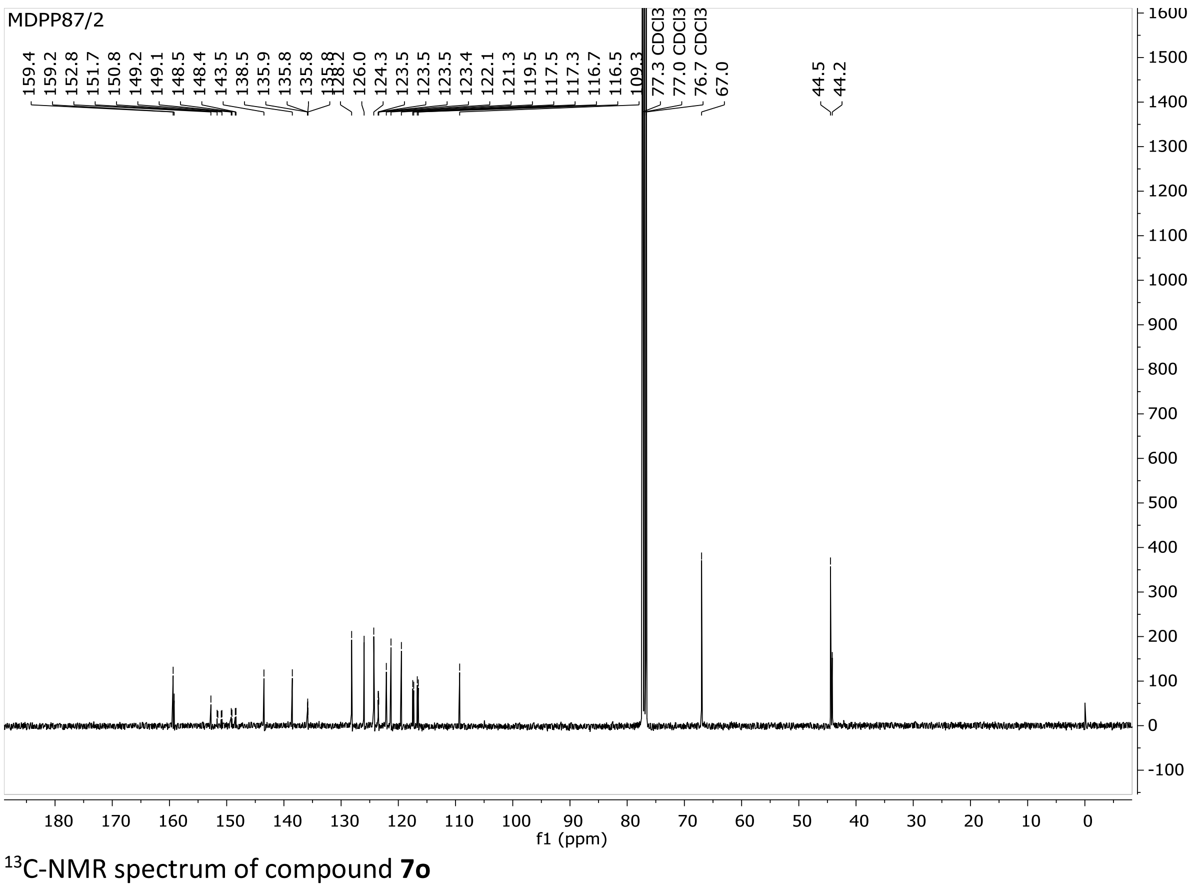

^{13}C-NMR spectrum of compound **7o**

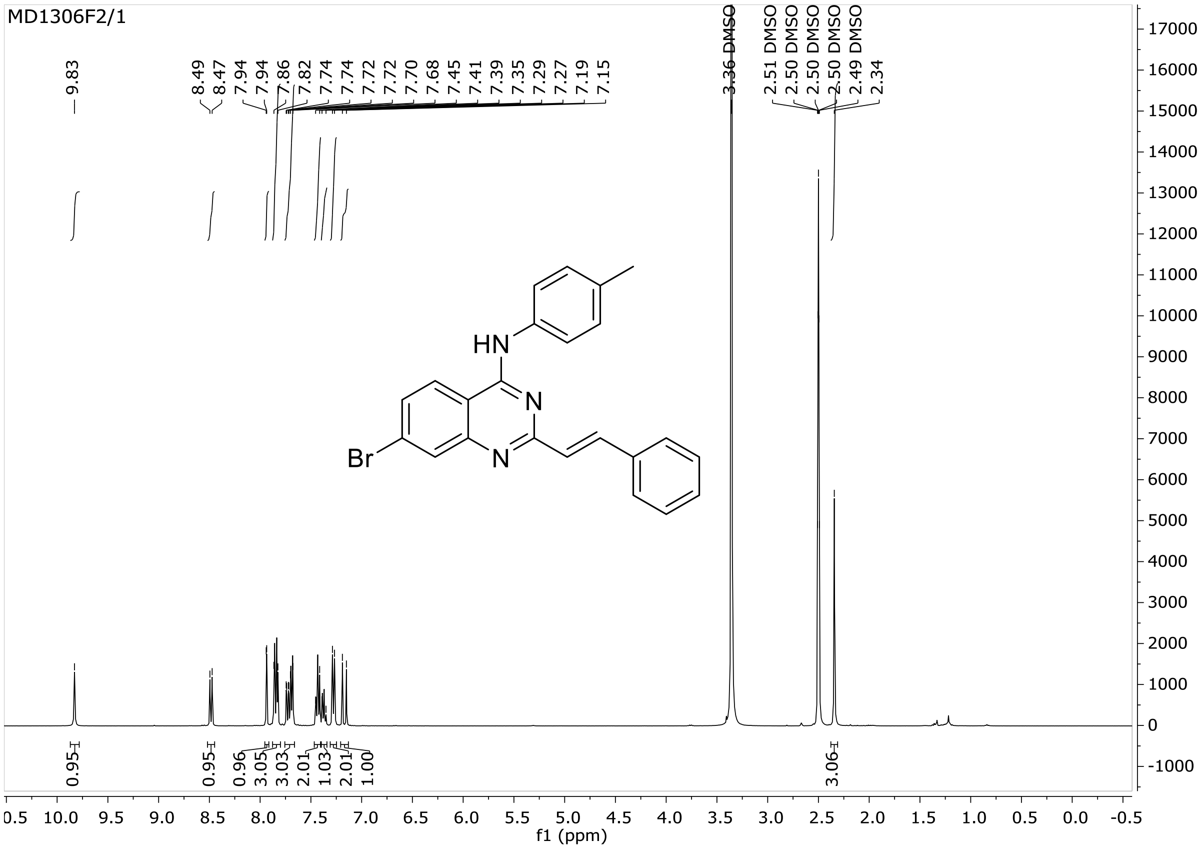

^{1}H-NMR spectrum of compound **8a**

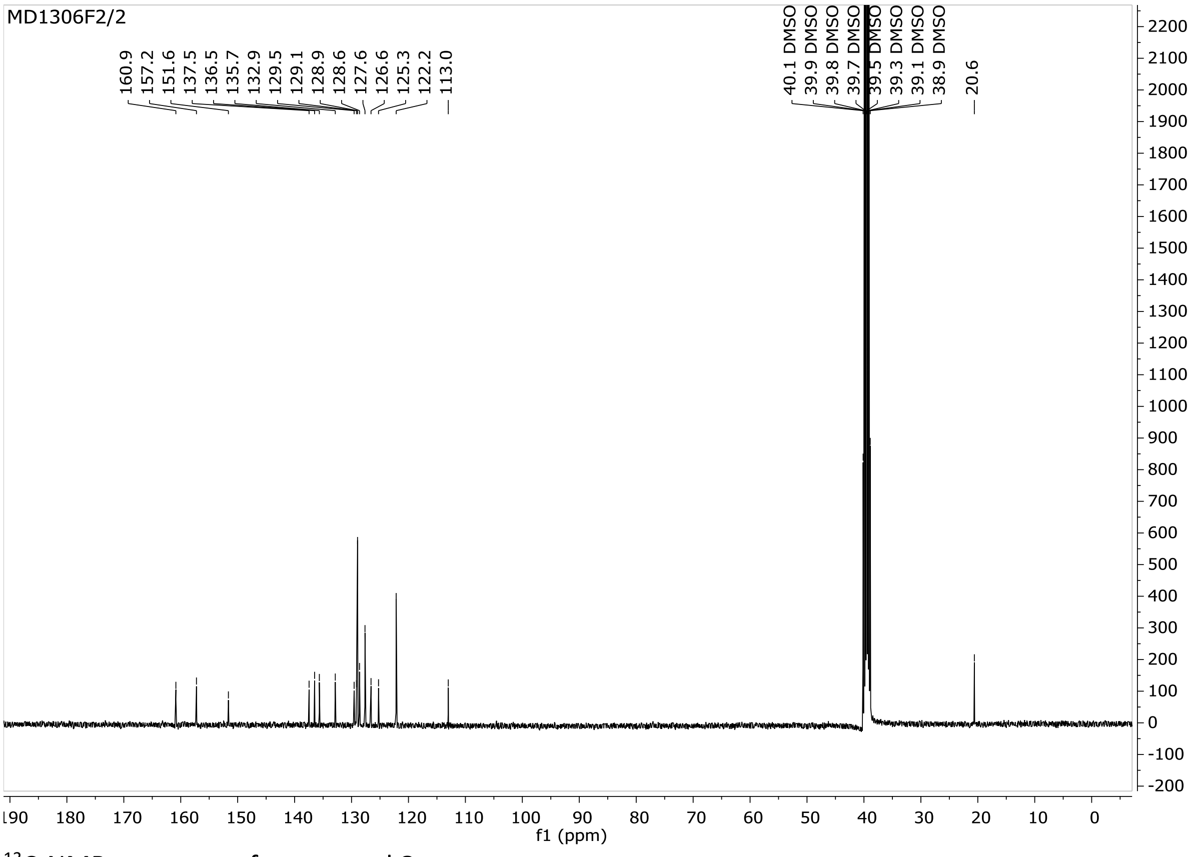

^{13}C-NMR spectrum of compound **8a**

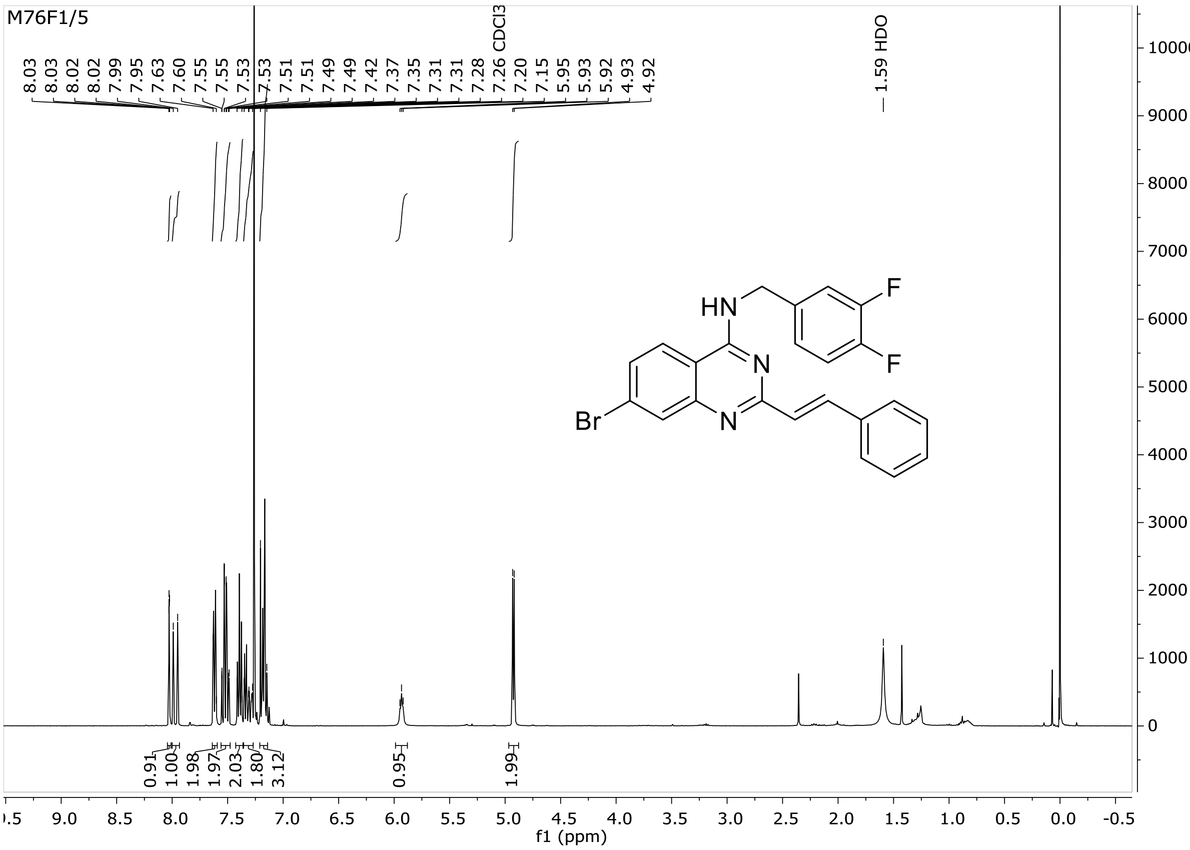

^{1}H-NMR spectrum of compound **8b**

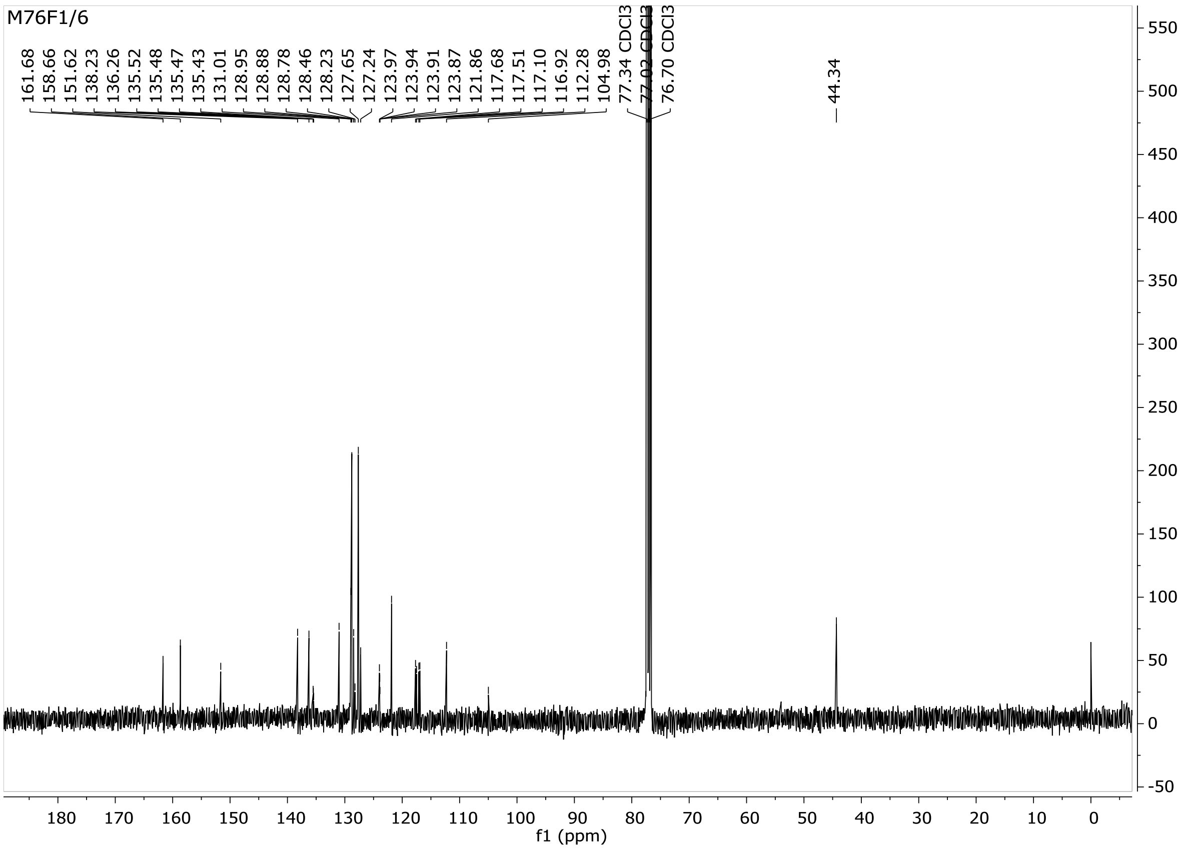

^{13}C-NMR spectrum of compound **8b**

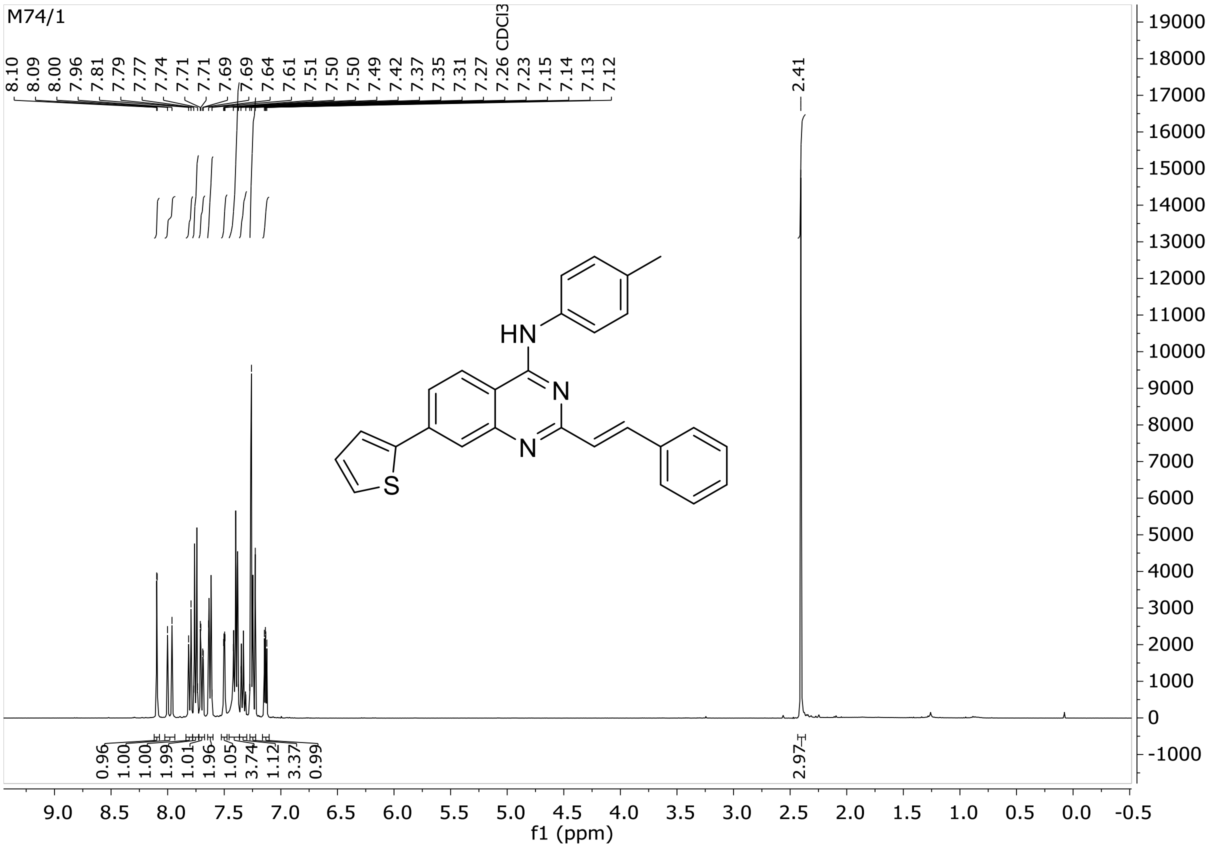

^{1}H-NMR spectrum of compound **9a**

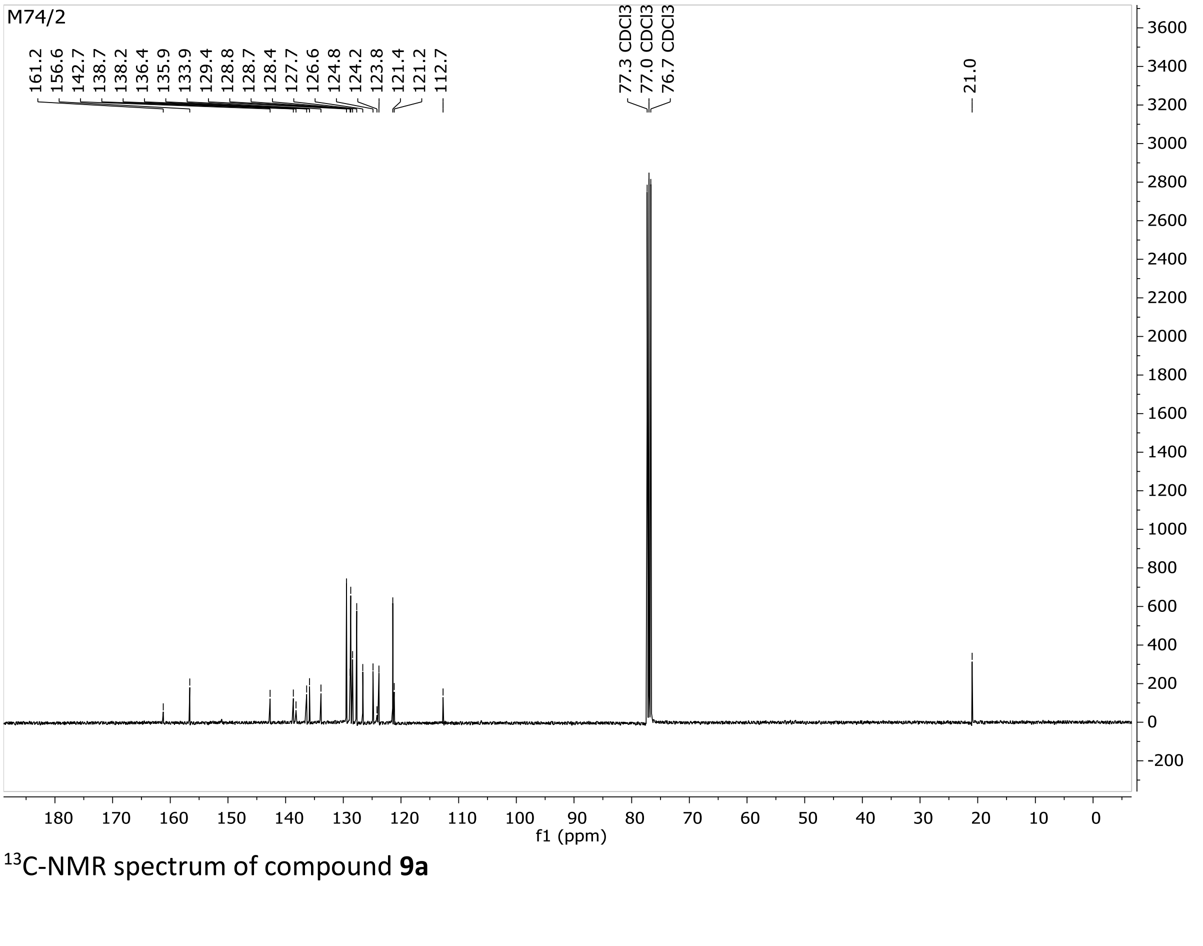

^{13}C-NMR spectrum of compound **9a**

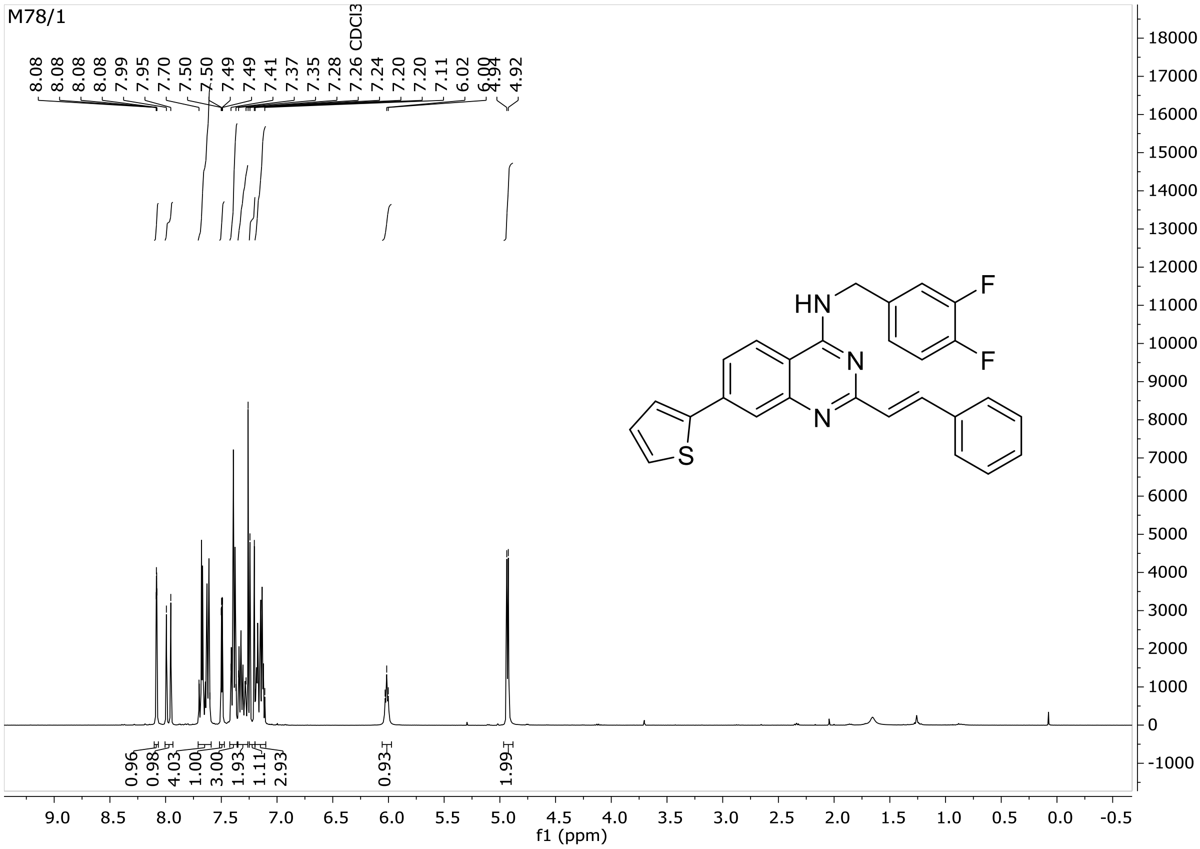

^{1}H-NMR spectrum of compound **9b**

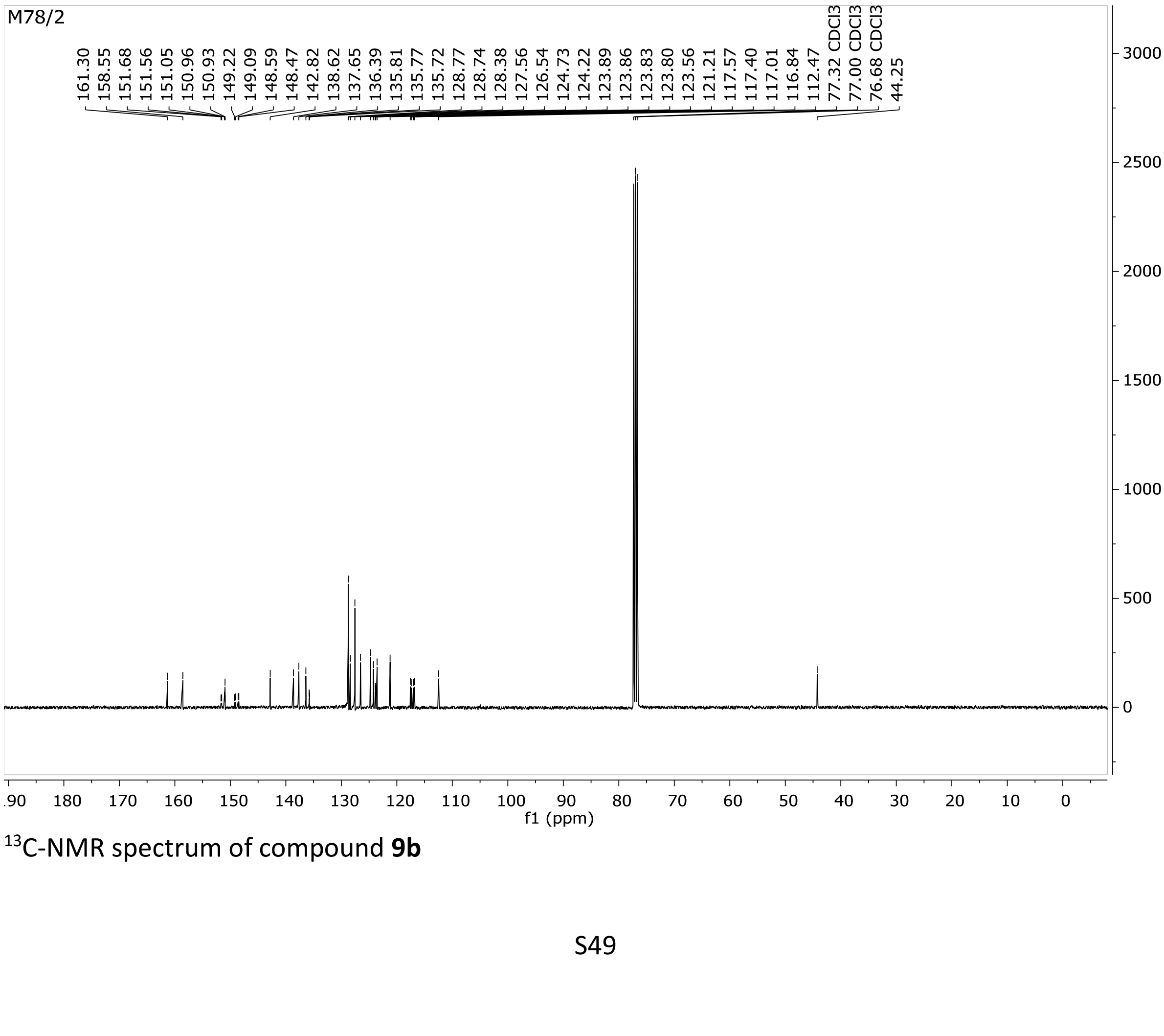

^{13}C-NMR spectrum of compound **9b**

Table 1S. Percentual inhibition of COX-1 and COX-2 isoenzymes, respectively, by tested compounds at 20 μM.

Compound	Inhibition %		Compound	Inhibition %		Compound	Inhibition %	
	COX-1	COX-2		COX-1	COX-2		COX-1	COX-2
**3a**	50.49	-38.70	**5a**	48.80	22.69	**8a**	82.21	27.49
**3b**	95.16	3.54	**5b**	30.38	30.65	**8b**	84.65	21.54
**3c**	95.29	2.64						
**3d**	31.24	-4.94	**6a**	34.09	7.04	**9a**	97.22	16.59
**3e**	6.76	-16.33	**6b**	26.57	27.64	**9b**	96.97	38.96
**3f**	27.63	-14.53	**6c**	92.40	32.29			
**3g**	13.74	-24.45	**6d**	31.62	26.49			
**3h**	31.52	3.02	**6e**	96.48	39.92			
**3i**	17.70	-11.07						
**3j**	41.61	13.80	**7a**	-3.35	0.80			
**3k**	96.76	71.78	**7b**	6.91	19.38			
**3l**	46.16	14.95	**7c**	-16.49	24.18			
**3m**	14.00	9.92	**7d**	-10.92	-1.39			
**3n**	12.77	5.59	**7e**	-5.92	-7.39			
**3o**	7.33	-4.79	**7f**	32.14	3.22			
**3p**	37.96	-22.92	**7g**	-13.89	-10.07			
**3q**	24.91	12.61	**7h**	-1.12	4.43			
**3r**	30.65	-1.77	**7i**	14.85	-5.36			
**3s**	23.09	-14.13	**7j**	-38.13	-2.17			
**3t**	20.84	10.81	**7k**	6.66	7.98			
**3u**	16.79	16.40	**7l**	-23.64	8.82			
**3v**	94.26	3.50	**7m**	-6.25	11.65			
			**7n**	17.97	14.63			
			**7o**	66.00	-3.62			

Table 2S. COX-1 inhibition by compound **9b** and Ibuprofen at different substrate (arachidonic acid, AA) concentrations.

Compound (conc. AA)	IC_{50} (μM)
**9b** (250 nM AA)	0.0211 ± 0.0051
**9b** (1250 nM AA)	0.0728 ± 0.0255
**9b** (6250 nM AA)	0.2536 ± 0.0648
IBU (250 nM AA)	4.00 ± 1.52
IBU (1250 nM AA)	39.9 ± 11.2
IBU (6250 nM AA)	70.1 ± 0.32

Graph 1S. Percentual inhibition of COX-1 by active compounds at different concentrations - depicted as % inhibition vs. log(concentration) in µM

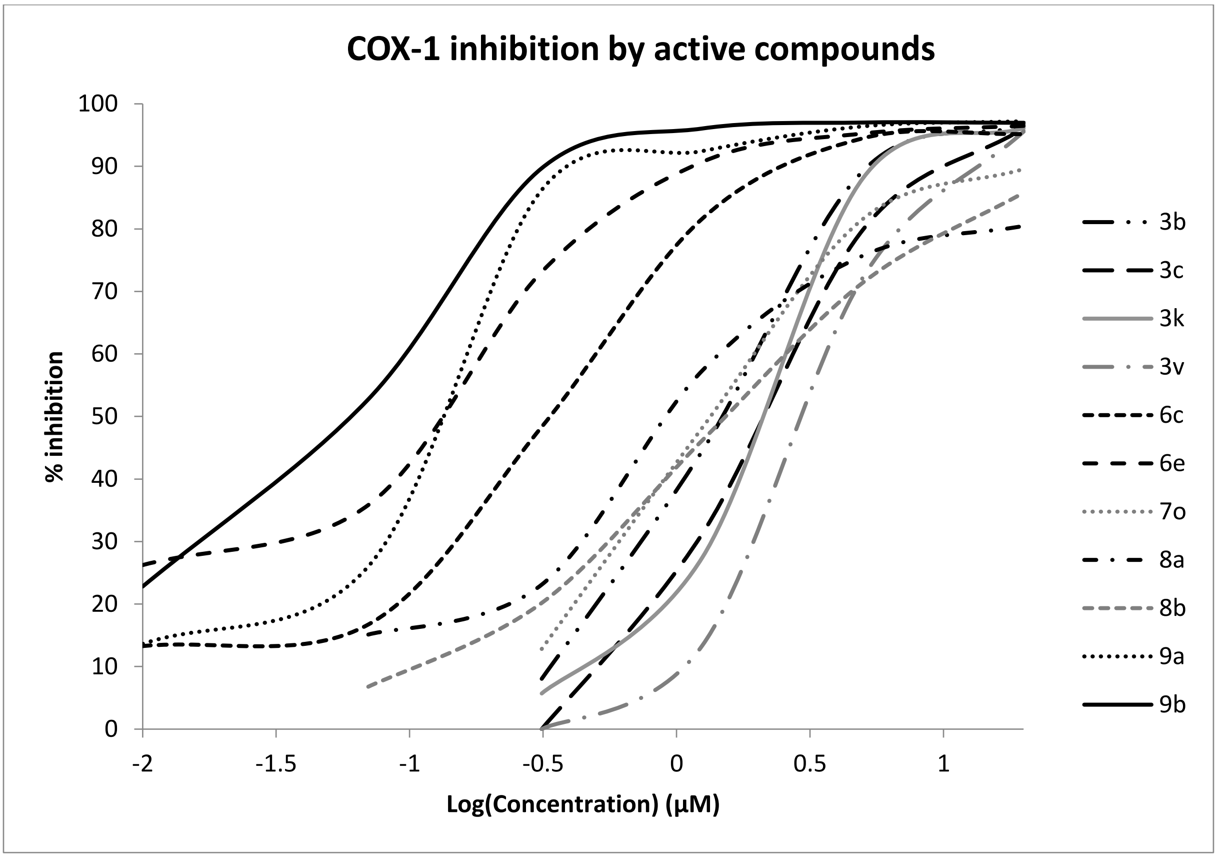

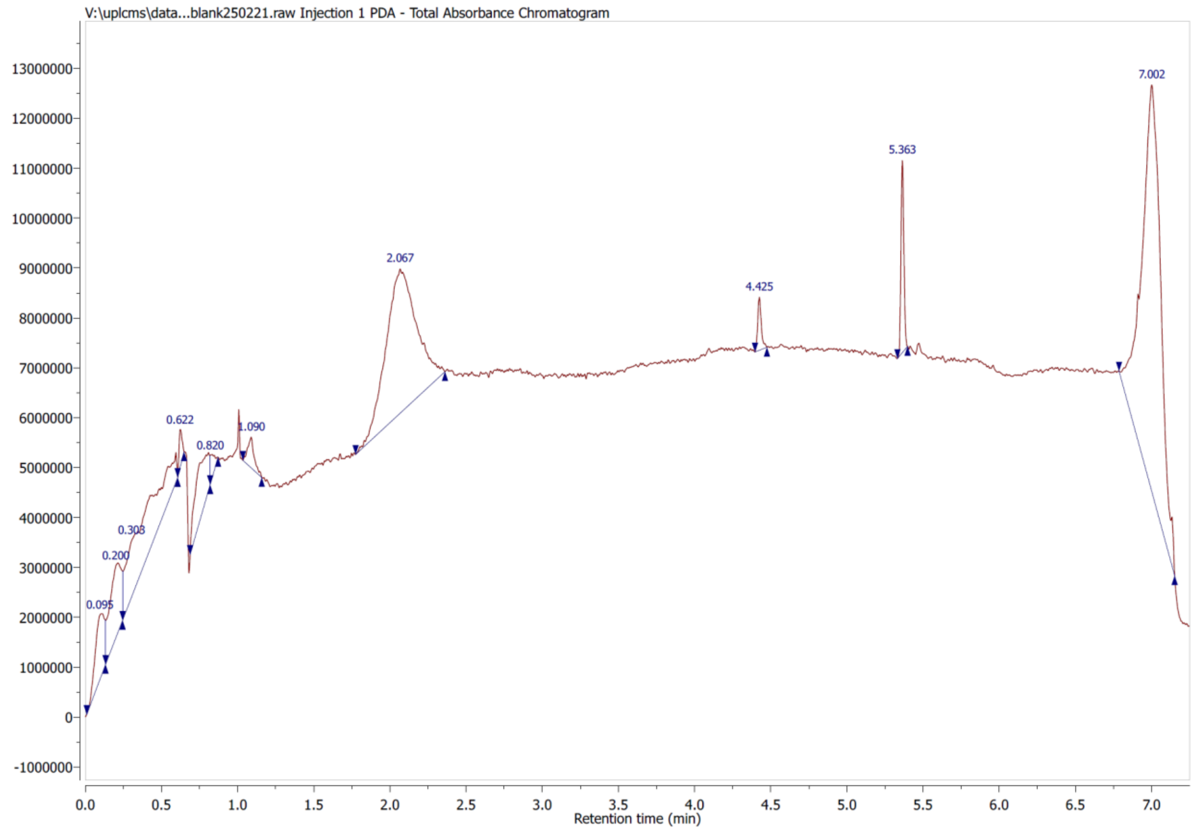

UPLC/UV-VIS chromatogram_Blank_1

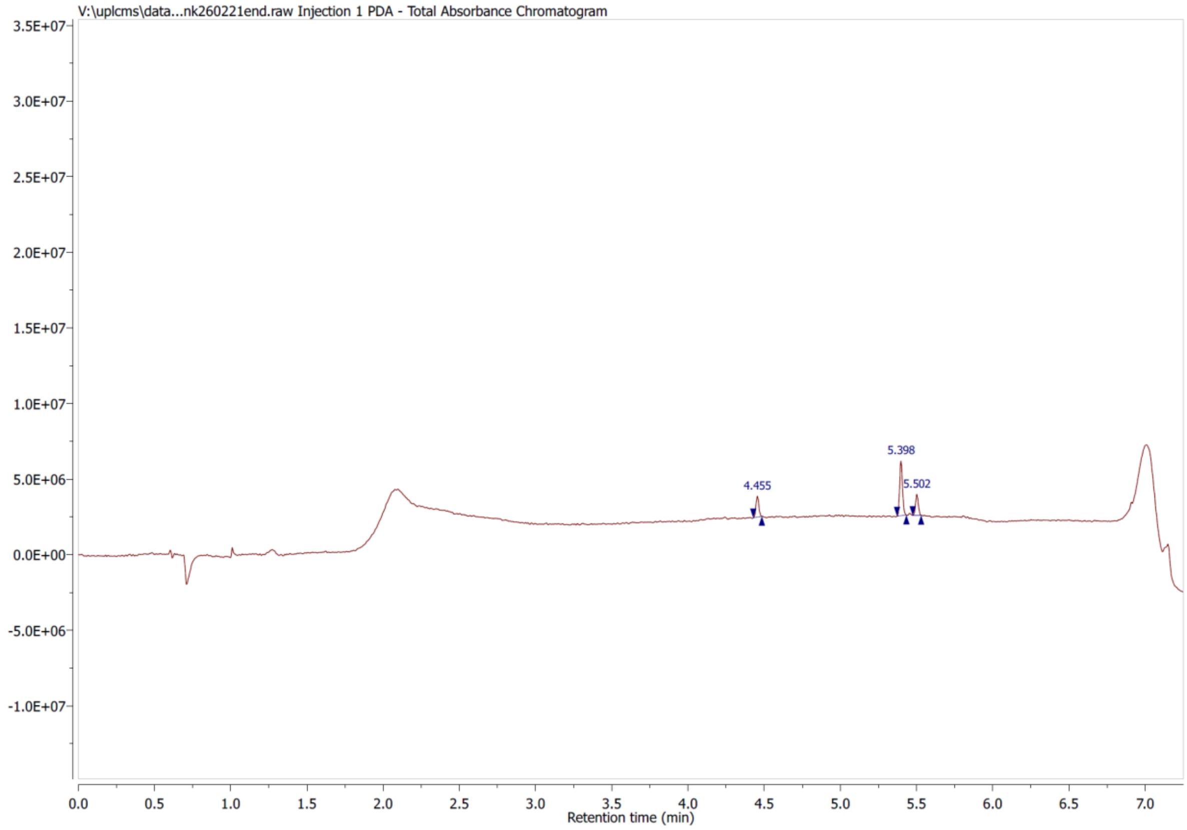

UPLC/UV-VIS chromatogram_Blank_2

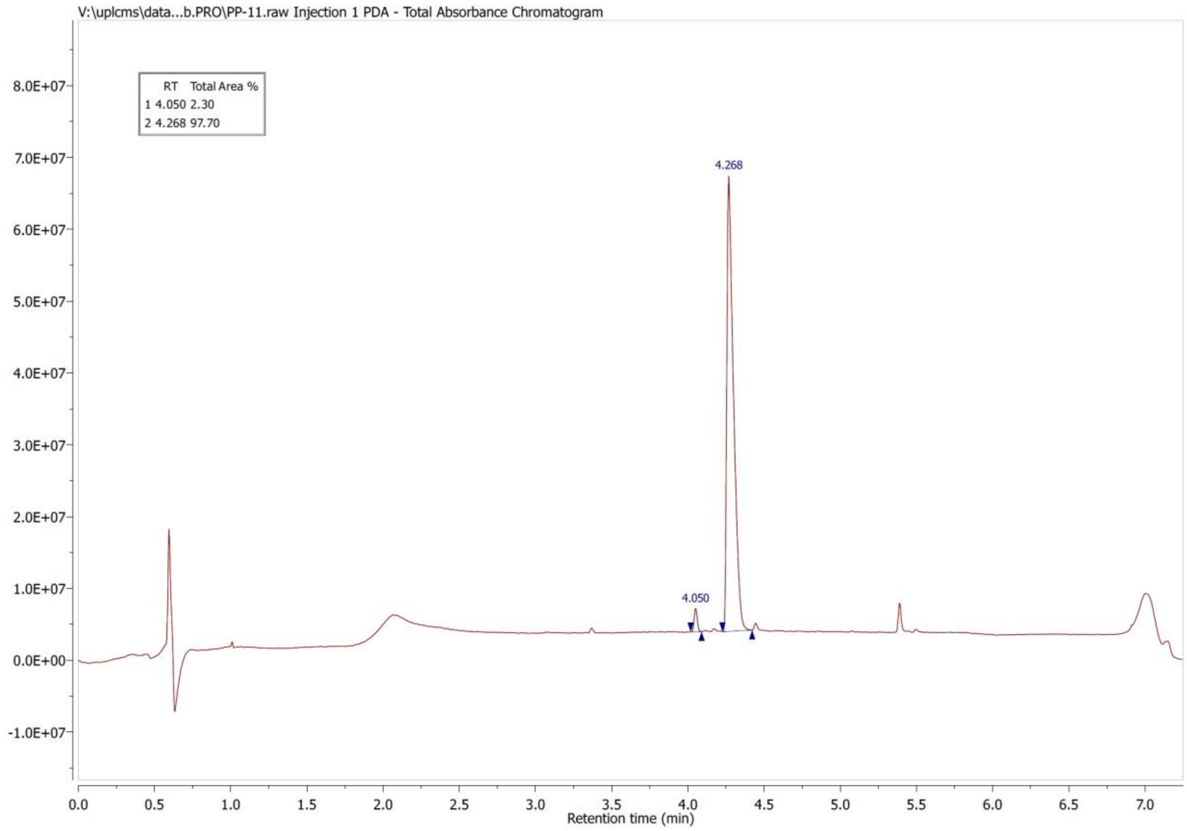

UPLC/UV-VIS chromatogram_Compound **3b**

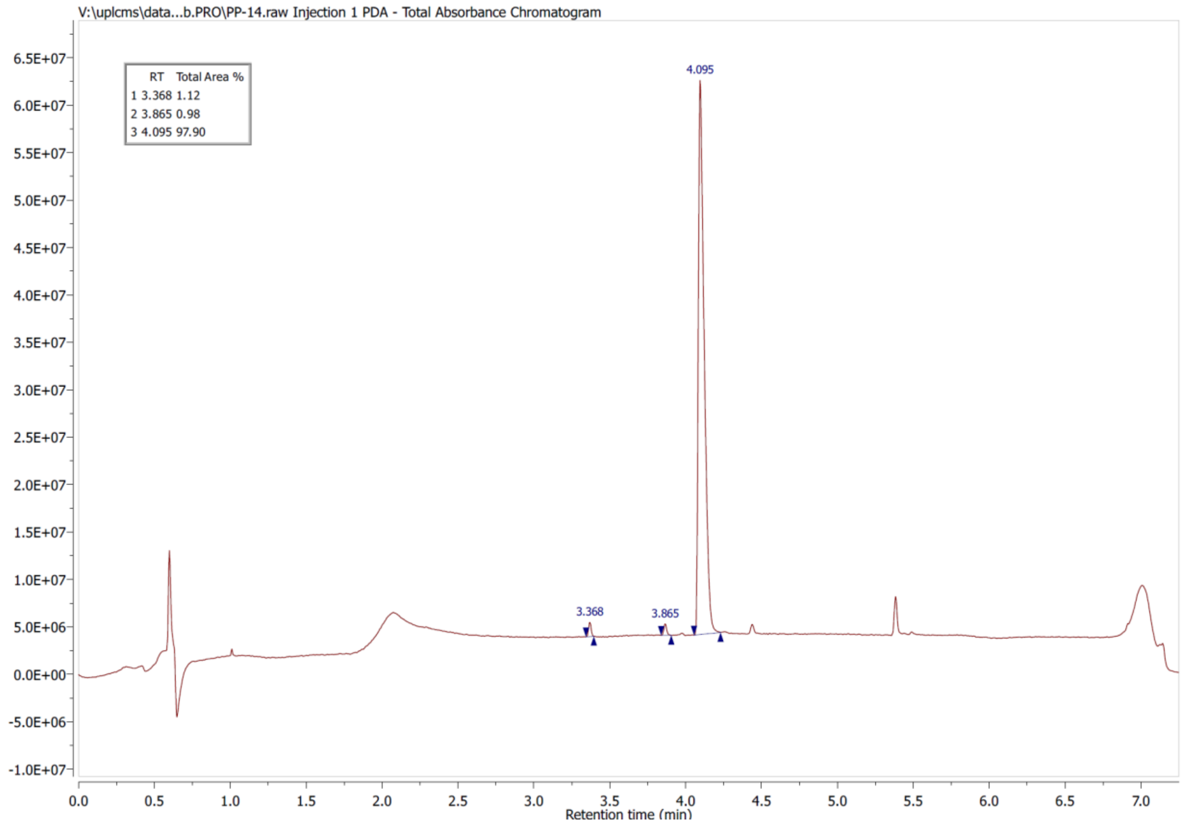

UPLC/UV-VIS chromatogram_Compound **3c**

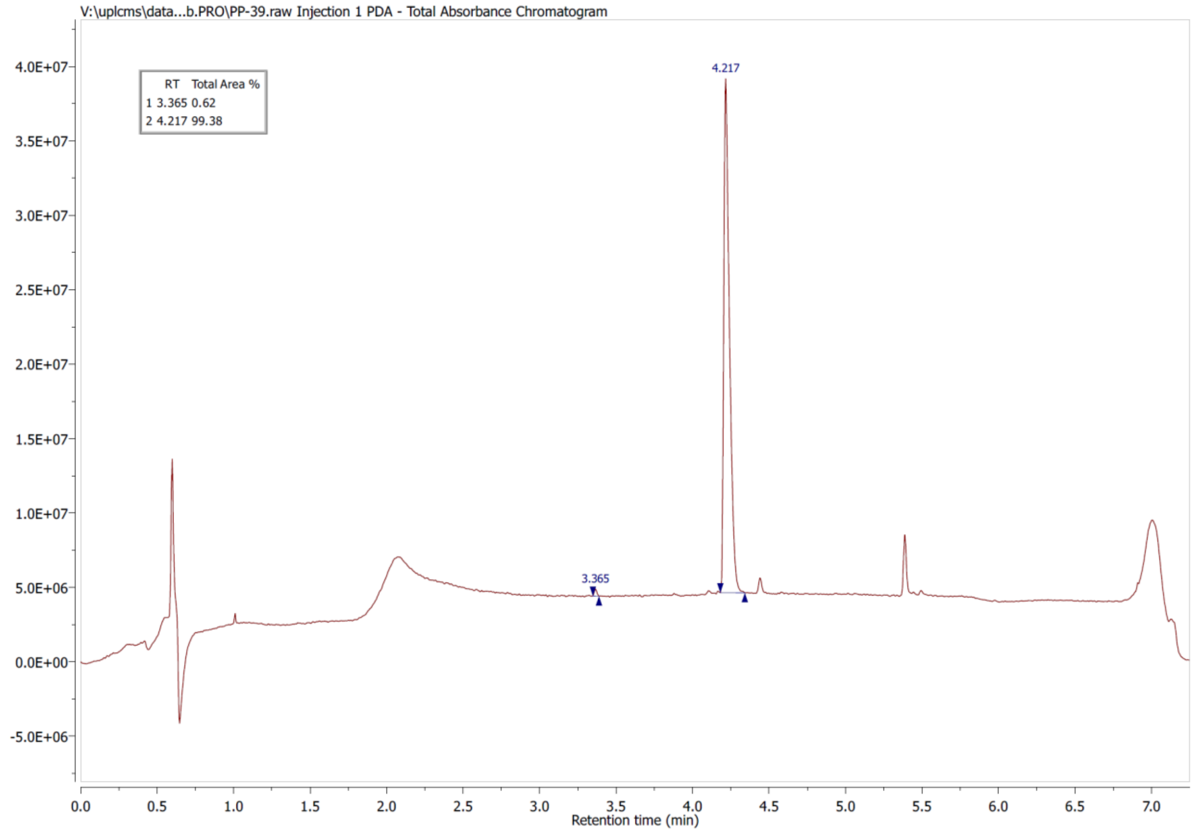

UPLC/UV-VIS chromatogram_Compound **3k**

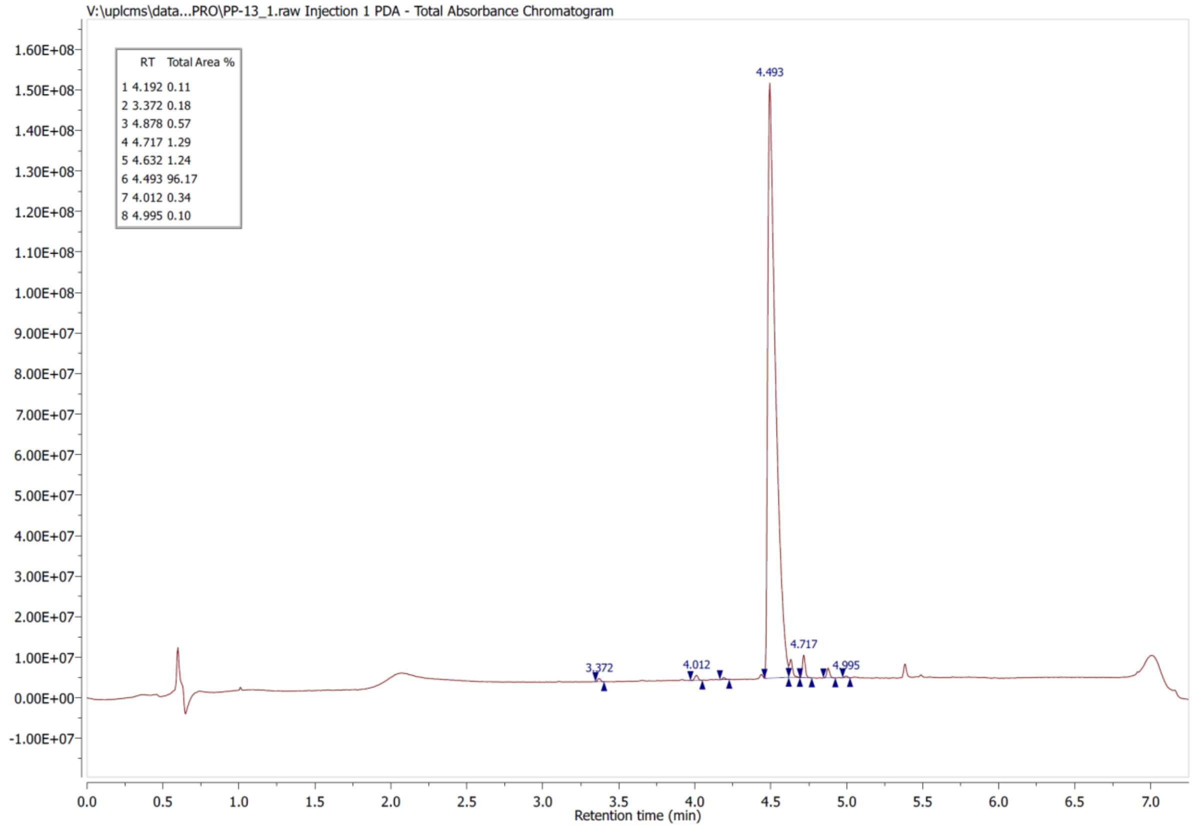

UPLC/UV-VIS chromatogram_Compound **3v**

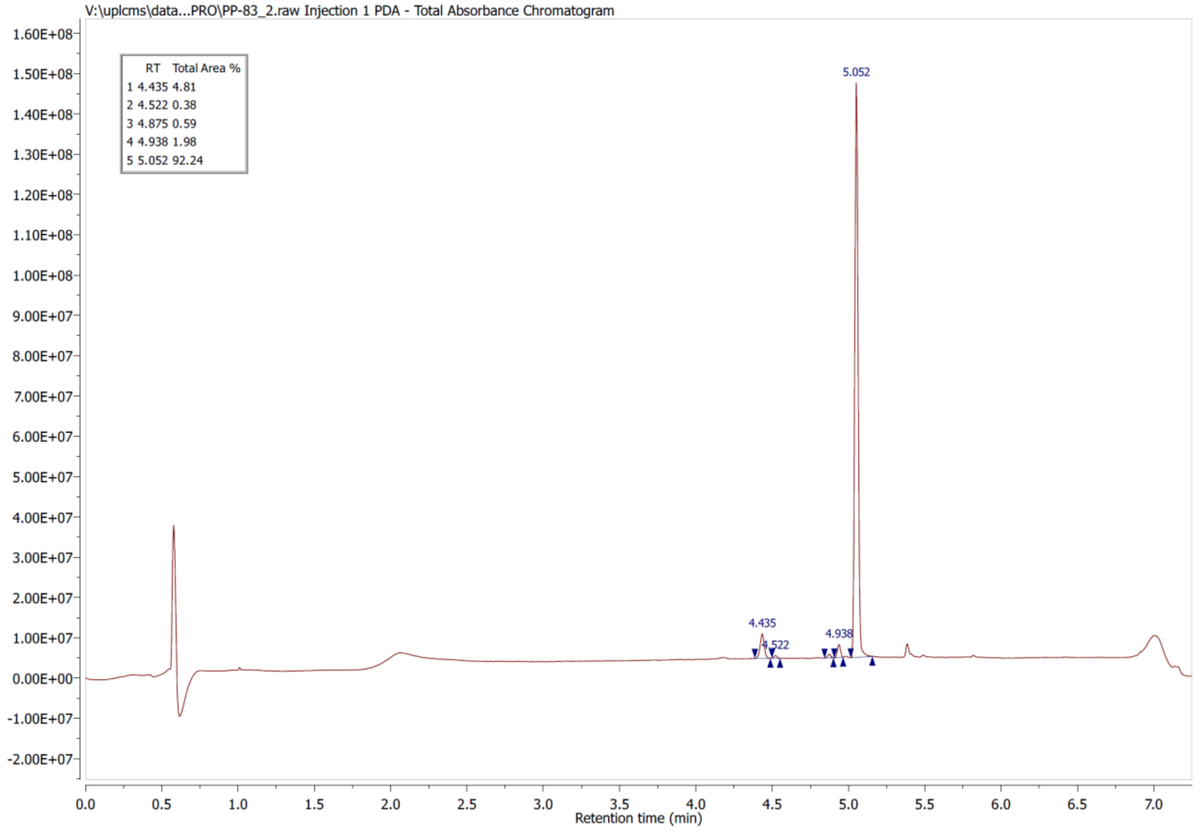

UPLC/UV-VIS chromatogram_Compound **6c**

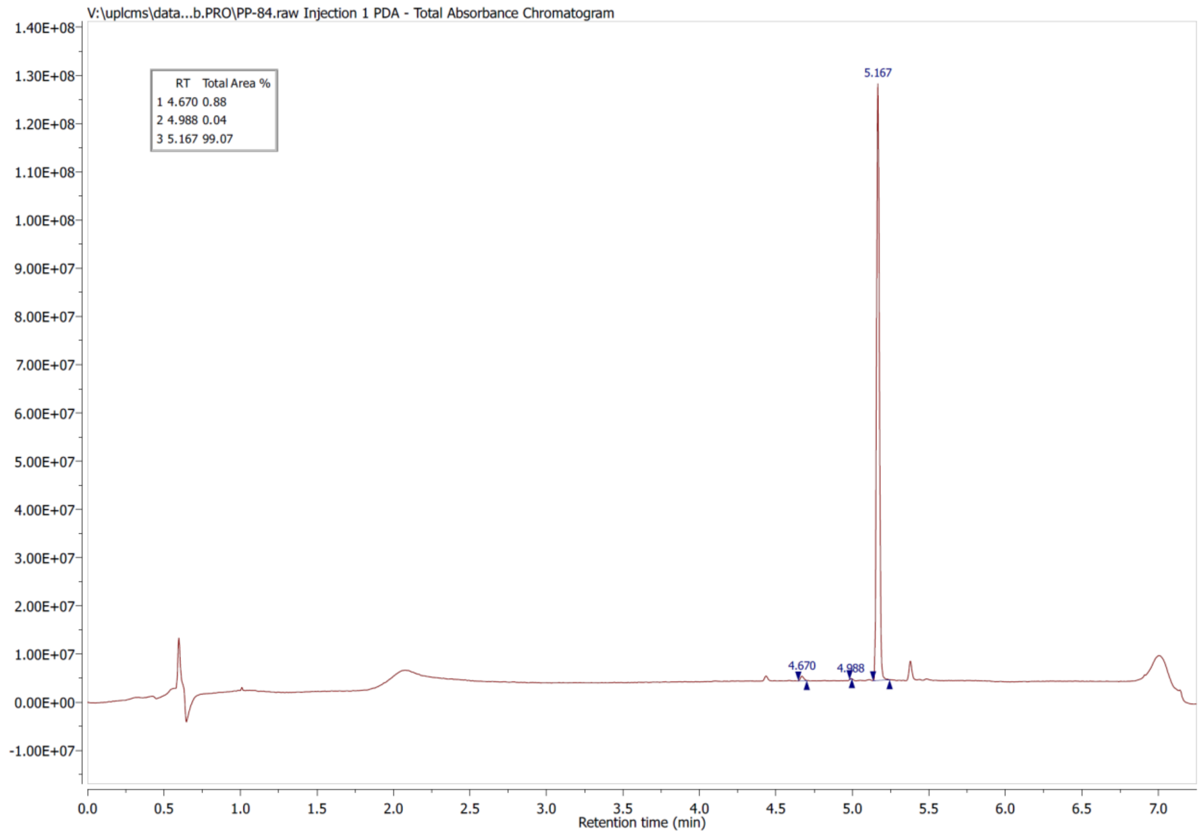

UPLC/UV-VIS chromatogram_Compound **6e**

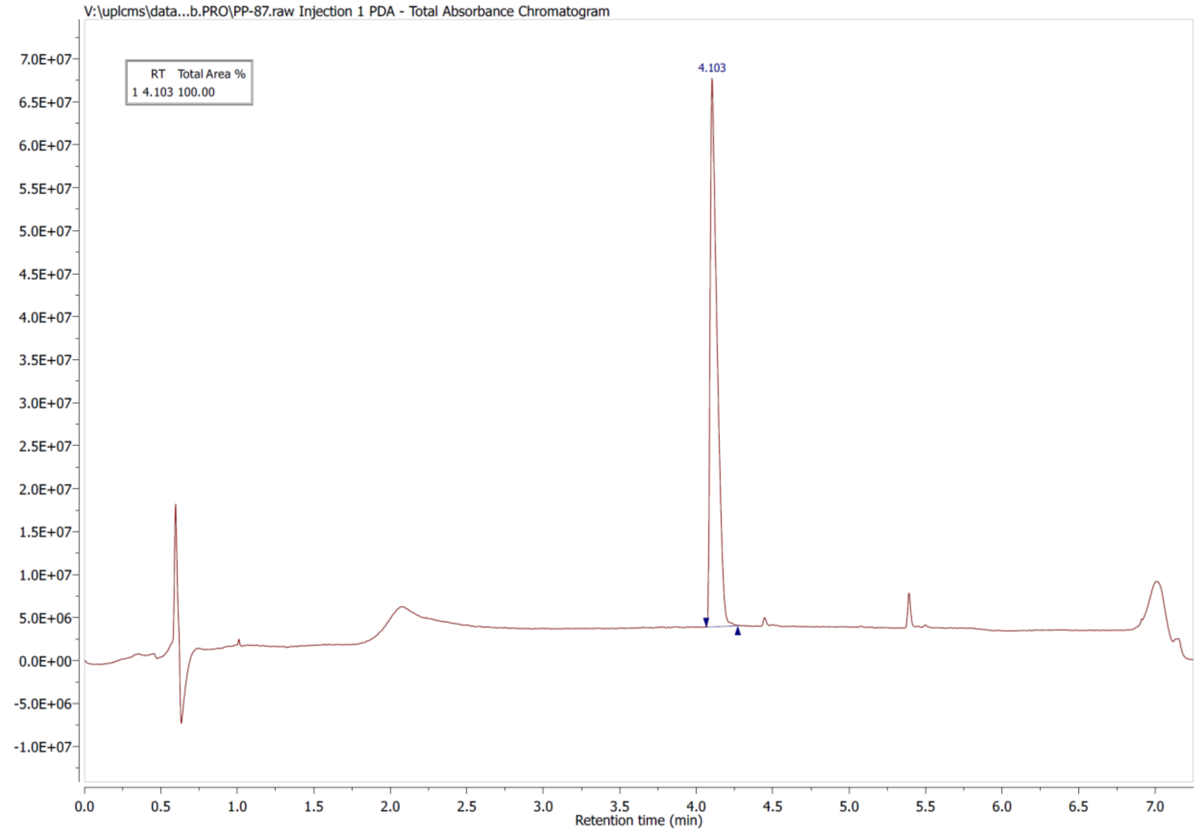

UPLC/UV-VIS chromatogram_Compound **7o**

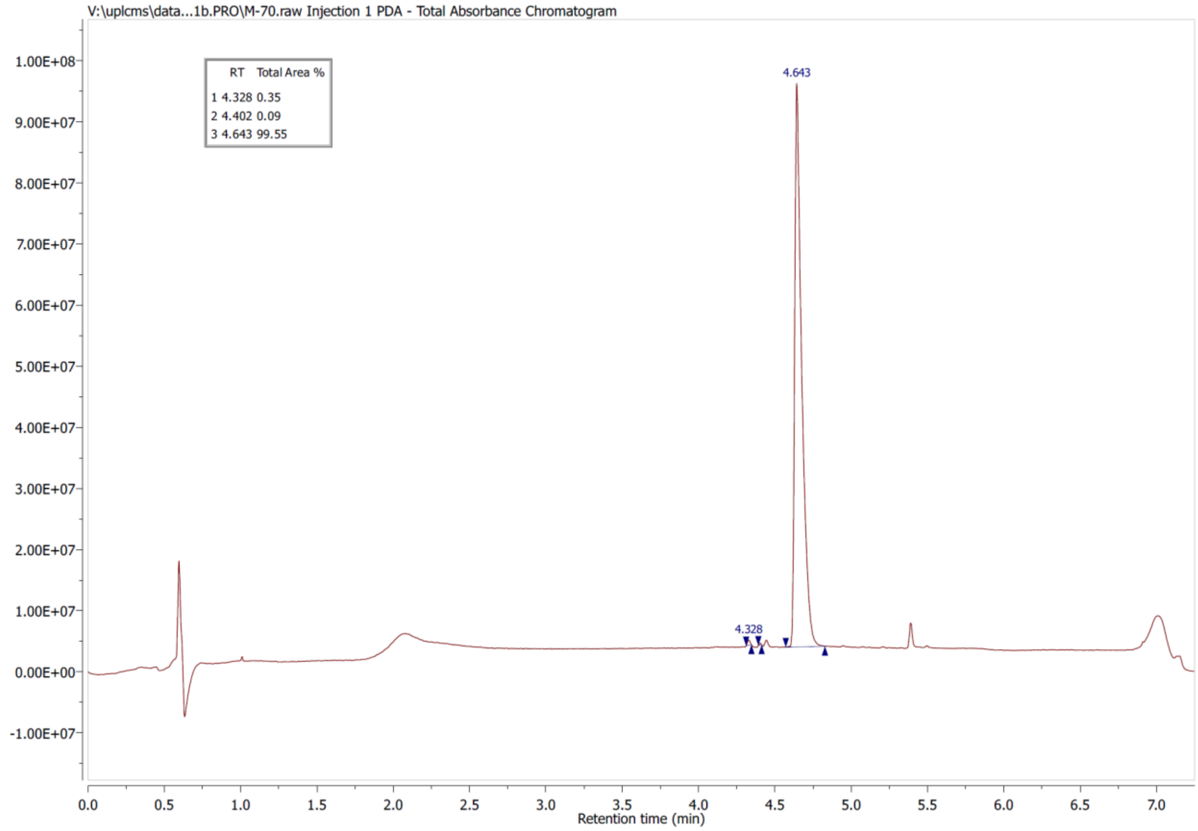

UPLC/UV-VIS chromatogram_Compound **8a**

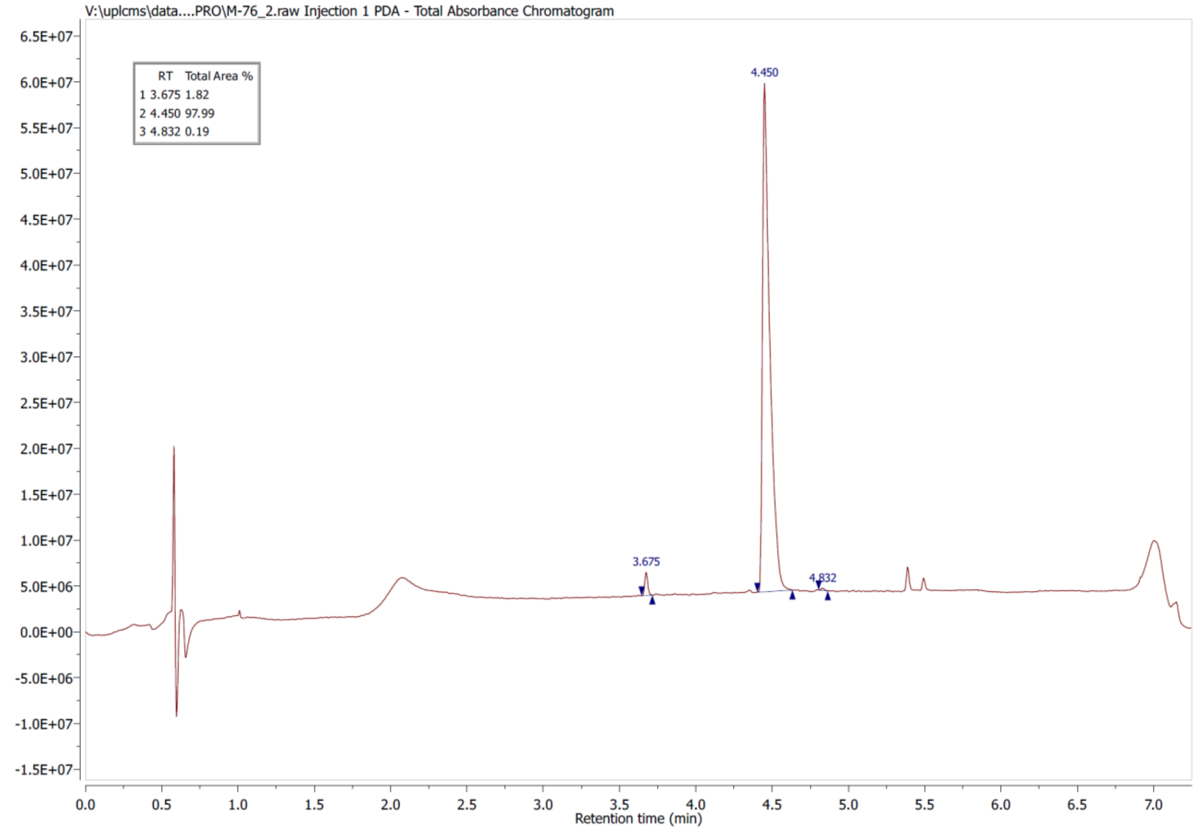

UPLC/UV-VIS chromatogram_Compound **8b**

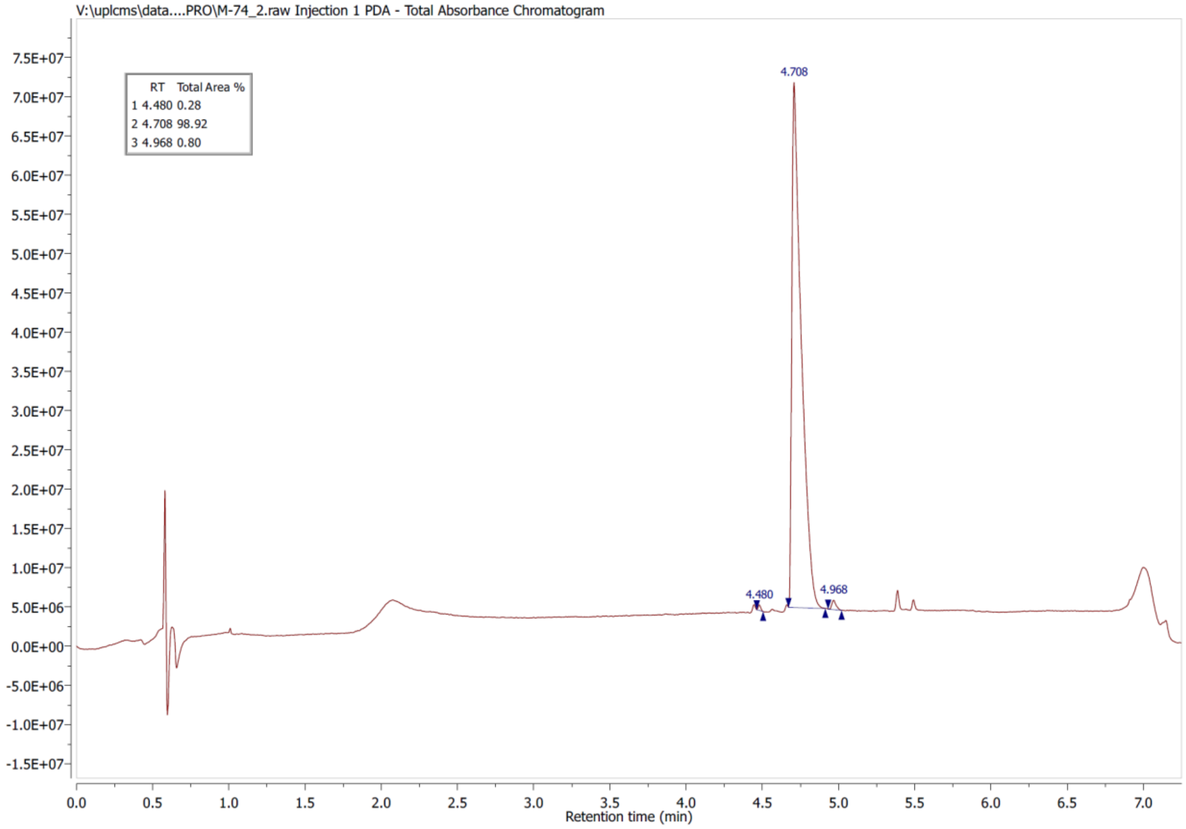

UPLC/UV-VIS chromatogram_Compound **9a**

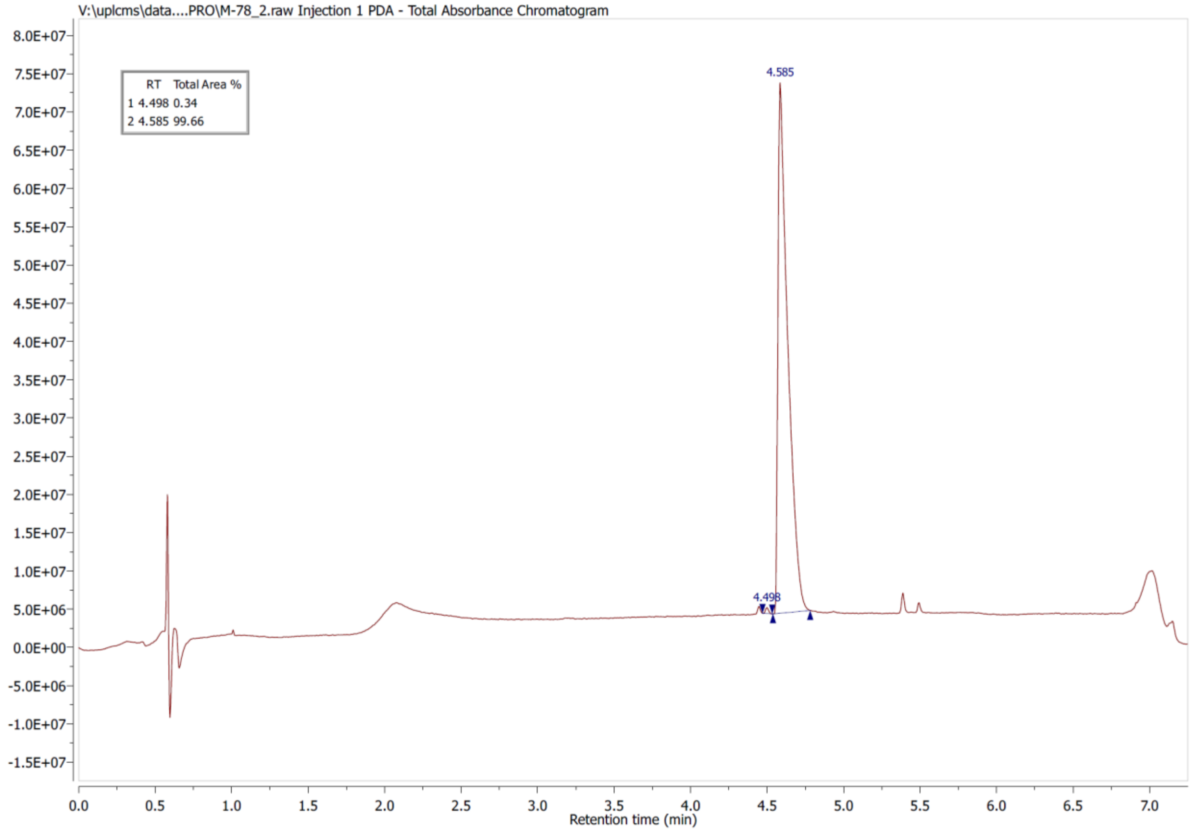

UPLC/UV-VIS chromatogram_Compound **9b**
